# Two glycosyltransferases involved in anthocyanin modification delineated by transcriptome independent component analysis in *Arabidopsis thaliana*

**DOI:** 10.1111/j.1365-313X.2011.04779.x

**Published:** 2011-10-14

**Authors:** Keiko Yonekura-Sakakibara, Atsushi Fukushima, Ryo Nakabayashi, Kousuke Hanada, Fumio Matsuda, Satoko Sugawara, Eri Inoue, Takashi Kuromori, Takuya Ito, Kazuo Shinozaki, Bunyapa Wangwattana, Mami Yamazaki, Kazuki Saito

**Affiliations:** 1 RIKEN Plant Science Center1-7-22, Suehiro-cho, Tsurumi-ku, Yokohama 230-0045, Japan; 2 Graduate School of Nanobiosciences, Yokohama City University1-7-29, Suehiro-cho, Tsurumi-ku, Yokohama 230-0045, Japan; 3 Graduate School of Pharmaceutical Sciences, Chiba University1-33 Yayoi-cho, Inage-ku, Chiba 263-8522, Japan; 4 CREST, Japan Science and Technology Agency4-1-8 Honcho, Kawaguchi, Saitama 332-0012, Japan; 5 Antibiotics LaboratoryRIKEN, 2-1 Hirosawa, Wako, Saitama 351-0198, Japan

**Keywords:** xylosyltransferase, glycosyltransferase, anthocyanin, flavonoid, *Arabidopsis thaliana*

## Abstract

To identify candidate genes involved in Arabidopsis flavonoid biosynthesis, we applied transcriptome coexpression analysis and independent component analyses with 1388 microarray data from publicly available databases. Two glycosyltransferases, UGT79B1 and UGT84A2 were found to cluster with anthocyanin biosynthetic genes. Anthocyanin was drastically reduced in *ugt79b1* knockout mutants. Recombinant UGT79B1 protein converted cyanidin 3-*O*-glucoside to cyanidin 3-*O*-xylosyl(1→2)glucoside. UGT79B1 recognized 3-*O*-glucosylated anthocyanidins/flavonols and uridine diphosphate (UDP)-xylose, but not 3,5-*O*-diglucosylated anthocyanidins, indicating that UGT79B1 encodes anthocyanin 3-*O*-glucoside: 2′′-*O*-xylosyltransferase. *UGT84A2* is known to encode sinapic acid: UDP-glucosyltransferase. In *ugt84a2* knockout mutants, a major sinapoylated anthocyanin was drastically reduced. A comparison of anthocyanin profiles in *ugt84a* knockout mutants indicated that UGT84A2 plays a major role in sinapoylation of anthocyanin, and that other UGT84As contribute the production of 1-*O*-sinapoylglucose to a lesser extent. These data suggest major routes from cyanidin 3-*O*-glucoside to the most highly modified cyanidin in the potential intricate anthocyanin modification pathways in Arabidopsis.

## Introduction

One of the goals of plant secondary metabolism research is to formulate a comprehensive understanding of gene functions in a particular synthetic pathway, including regulation and crosstalk with other metabolic processes and/or metabolites. However, the enzymes involved in secondary metabolism can be encoded by multigene families, making it difficult to determine their precise physiological functions (D’Auria and Gershenzon, 2005; Bowles *et al.*, 2006). Completion of several plant genome sequencing projects has made cataloging a finite number of genes possible, as well as the development of multiple databases and bioresources for transcriptomes, proteomes, metabolomes and phenomes (Yonekura-Sakakibara and Saito, 2009; Mochida and Shinozaki, 2010). These ‘omics platforms also provide the tools for genome-wide approaches based on sequence similarity, transcriptomics, and correlations between transcripts and metabolites in addition to the more traditional biochemical and reverse genetics approaches (Fridman and Pichersky, 2005; Moreno-Risueno *et al.*, 2010; Saito and Matsuda, 2010). These strategies facilitate an efficient narrowing-down of candidate genes involved in pathways of interest.

Flavonoids including the anthocyanins, flavonols, and flavones are some of the most intensely studied secondary metabolites with over 7000 known structures (Harborne and Baxter, 1999; Anderson and Markham, 2006). Many of them are important as flower pigments, UV-B protectants, signaling molecules between plants and microbes, and regulators of auxin transport (Dooner *et al.*, 1991; Dixon and Paiva, 1995). Biosynthetic pathways leading to the flavonoid aglycones have been well-studied, and the corresponding regulatory and synthesis genes have been characterized in various plants (Davies and Schwinn, 2006; Tanaka *et al.*, 2008). However, the pathways for sequential modification, such as glycosylation, acylation, and methylation, are still relatively unexplored, even although these modifications produce enormous chemical diversity and are essential for the stable accumulation of flavonoids.

*Arabidopsis thaliana* is one of the most studied plants for the molecular biology of flavonoid metabolism. The largest number of flavonoid-related genes including transcription factors have been identified from Arabidopsis because of the extensive ‘omics’-based information and bioresources available for this species. In Arabidopsis, flavonoid skeleton biosynthetic genes have been isolated on the basis of similarity or mutant phenotypes (Feinbaum and Ausubel, 1988; Shirley *et al.*, 1992; Pelletier and Shirley, 1996; Pelletier *et al.*, 1997; Kitamura *et al.*, 2004). The genes involved in modification of flavonols and anthocyanins, flavonol 3-*O*-rhamnosyltransferase, flavonol 7-*O*-glucosyltransferase and anthocyanin sinapoyltransferase, have also been identified by genome-wide methods based on similarity (Jones *et al.*, 2003; Fraser *et al.*, 2007). Genes involved in flavonoid biosynthesis are in general coordinately expressed. Two genes encoding flavonoid glycosyltransferases, flavonoid 3-*O*-glucosyltransferase and anthocyanin 5-*O*-glucosyltransferase, were identified by transcriptome analysis of *A.* *thaliana* overexpressing *PAP1*, a transcription factor for anthocyanin biosynthesis (Tohge *et al.*, 2005). Furthermore, two genes encoding flavonol glycosyltransferases, flavonol 7-*O*-rhamnosyltransferase and flavonol 3-*O*-arabinosyltransferase, were efficiently targeted from among over 100 candidate UGTs by transcriptome coexpression analyses using correlation coefficients which had been calculated based on publicly available transcriptome data (Yonekura-Sakakibara *et al.*, 2007, 2008). Functional identification of six kinds of flavonoid glycosyltransferases in one plant species allowed us to expand the search for substrate specificity, regiospecificity and evolutionary processes. However, even in this highly studied species, its flavonoid structures suggest that there are as yet identified genes encoding modification enzymes.

To identify more candidate genes involved in flavonoid biosynthesis, we used independent component analysis (ICA) in addition to transcriptome coexpression analyses using correlation coefficients. ICA, a form of unsupervised algorithm, has been used as an effective analytical tool for microarray gene expression data (Kong *et al.*, 2008). Originally ICA was developed as a method for multi-channel signal processing to separate mixed signals into their different sources. In the gene expression context, ICA has been applied to extract and characterize the informative features of biological signals from microarray data on the assumption that the level of gene expression is determined by a linear combination of some independent components corresponding to biological signals. Precise gene clustering and classification was achieved by ICA on yeast microarray data during sporulation (Hori *et al.*, 2001) and during the cell replication cycle (Liebermeister, 2002). ICA can be also used for screening genes involved in oncogenesis and in Alzheimer’s disease (Chiappetta *et al.*, 2004; Saidi *et al.*, 2004; Frigyesi *et al.*, 2006; Kong *et al.*, 2009). The genes involved in the biosynthesis of anthocyanins were categorized by ICA into two clusters for anthocyanin skeleton biosynthesis and modification. Two glycosyltransferases, UGT79B1 and UGT84A2, were predicted to be anthocyanin biosynthetic genes by both transcriptome coexpression analyses using correlation coefficients and ICA, and by ICA only, respectively. Analyses of anthocyanin profiles in knockout mutants and recombinant protein assays demonstrated that *UGT79B1* encodes anthocyanin 3-*O*-glucoside: 2′′-*O*-xylosyltransferase and that, of the four UGT84As, UGT84A2 plays the major role in sinapoylation of anthocyanins. When considered in the context of the previously known genes in anthocyanin biosynthetic pathway, we have now assembled a ‘roadmap’ for the major anthocyanin modification routes in Arabidopsis.

## Results

### Anthocyanin UGT candidates deduced by independent component analysis and transcriptome coexpression analysis

**Figure 1:**
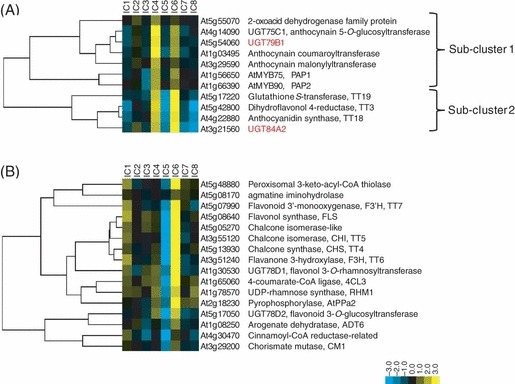
Major sub-clusters including anthocyanin and flavonol-related genes formed by hierarchical clustering of genes based on ICA. (A) A major sub-cluster for anthocyanin-related genes. The genes characterized in this study are shown in red. (B) A major sub-cluster for flavonol-related genes.

Previously, we conducted transcriptome coexpression analyses using correlation coefficients to identify all of the flavonoid-related genes and the relationship between flavonoid synthesis and other metabolic pathways. This examination of all Arabidopsis genes was performed using known 24 genes encoding flavonoid biosynthetic enzymes and transcription factors as query sequences (Yonekura-Sakakibara *et al.*, 2008). Twenty-four genes had two or more positive correlation (*r* > 0.525) with known flavonoid pathway-related genes. Over 100 genes also showed some correlation with one of the known flavonoid pathway-related genes. To shed new light on the transcriptome data and to identify previously undetected genes in the flavonoid biosynthetic pathway, ICA of a total of 1877 genes, including the flavonoid biosynthetic genes and all genes annotated in AraCyc, was performed on 1388 microarray data with ATTED-II (http://atted.jp/, ver.3) (Obayashi *et al.*, 2009) using the fastICA algorithm (Hyvärinen and Oja, 1997) (Figure S1, Data S1). A hierarchical cluster analysis of gene signature matrix *S* based on eight independent components (ICs) indicates that the genes involved in the biosynthesis of anthocyanins and flavonols form distinct clusters with a few exceptions. Anthocyanin biosynthetic genes form a cluster with two sub-clusters, one of which contains dihydroflavonol reductase (DFR), anthocyanidin synthase (ANS) and glutathione-*S*-transferase (GST), and is involved in anthocyanidin skeleton biosynthesis (sub-cluster 2, Figure 1a). The other contains anthocyanin 5-*O*-glucosyltransferase (At4g14090), anthocyanin 3-*O*-glucoside: 6′′-*O*-*p*-coumaroyltransferase (At1g03495), anthocyanin 5-*O*-glucoside: 6′′-*O*-malonyltransferase (At3g29590), and the anthocyanin-specific MYB transcription factors PAP1 (At1g56650) and PAP2 (At1g66390) (sub-cluster 1; Figure 1A). These data suggest that ICA can be used to classify genes into biologically meaningful clusters. In addition to known anthocyanin-related genes, UGT79B1 (At5g54060) and a 2-oxoacid dehydrogenase family protein (At5g55070) also belong to the anthocyanin modification sub-cluster. UGT84A2 (At3g21560) is part of the anthocyanin biosynthesis sub-cluster. Of these three candidate genes, UGT79B1 showed high correlation (Yonekura-Sakakibara *et al.*, 2008) and similar expression profile with known anthocyanin-specific biosynthetic genes (Solfanelli *et al.*, 2006; Kusano *et al.*, 2011). However, genes encoding 2-oxoacid dehydrogenase family protein and UGT84A2 did not correlate significantly with the anthocyanin-specific biosynthetic genes. By transcriptome coexpression analyses using correlation coefficients, *UGT84A2* had relatively higher correlation coefficients with flavonol-specific biosynthetic genes (At3g55120, chalcone isomerase, *r* = 0.521; At3g51240, flavanone 3-hydroxylase, *r* = 0.515; At1g06000, flavonol 7-*O*-rhamnosyltransferase, *r* = 0.488; At5g08640, flavonol synthase, *r* = 0.425; At5g13930, chalcone synthase, *r* = 0.424; ATTED-II all data ver. 3) than with anthocyanin-specific genes (At5g42800, DFR, *r* = 0.387; At5g17220, GST, *r* = 0.378; At4g22880, ANS, *r* = 0.369; ATTED-II all data ver. 3) although the slight but reproducible induction of UGT84A2 by *PAP1* over-expression suggests that *UGT84A2* may be more distantly regulated by *PAP1* (Tohge *et al.*, 2005).

Flavonol biosynthetic genes fall into two sub-clusters, but there is no clear separation between skeleton biosynthesis and modification (Figure 1B). Genes encoding peroxisomal 3-keto-acyl-CoA thiolase (At5g48880), pyrophosphorylase (At2g18230), arogenate dehydratase (At1g08250), cinnamoyl-CoA reductase like protein (At4g30470) and chorismate mutase (At3g29200) were found in the flavonoid cluster. Of these five candidate genes, only At5g48880 showed high correlation with known flavonoid-related genes (Yonekura-Sakakibara *et al.*, 2008). Thus, ICA identified additional candidate genes that are different from those tagged by transcriptome coexpression analyses based on simple correlation coefficients. We focused on the anthocyanin-related candidate genes, UGT79B1 and UGT84A2, because ICA based on eight ICs is apparently more suitable for analysis of the anthocyanin pathway.

### UGT79B1 belongs to the subfamily of UGTs catalyzing glycosylation at the sugar moiety of flavonoid glycosides

**Figure 2:**
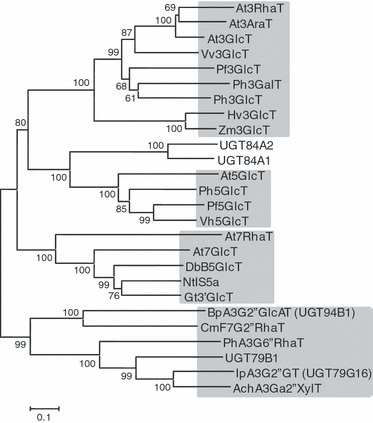
Non-rooted molecular phylogenetic tree of the flavonoid glycosyltransferases. The tree was constructed as described in Experimental procedures. The alignment used for this analysis is available in the supplementary material online (Data S2). Bar = 0.1 amino acid substitutions per site. The GenBank accession numbers for the sequences are shown in parentheses: At3RhaT (NM_102790); At3GlcT (NM_121711); At3AraT (NM_121709); Vv3GlcT (AF000371); Ph3GalT (AF316552); Ph3GlcT (AB027454); Pf3GlcT (AB002818); Hv3GlcT (X15694); Zm3GlcT (X13501); At5GlcT (NM_117485); Pf5GlcT (AB013596); Vh5GlcT (AB013598); Ph5GlcT (AB027455); At7GlcT (NM_129234); At7RhaT (NM_100480); DbB5GlcT (Y18871); NtIS5a (AF346431); Gt3′GlcT (AB076697); CmF7G2′′RhaT (AY048882); BpA3G2′′GlcAT (AB190262); AcA3Ga2′′XylT (FG404013); UGT79B1 (NM_124785); IpA3G2′′GlcT (AB192315); PhA3G2′′RhaT (Z25802); UGT84A2 (AY090952); UGT84A1 (BT002014). 3GlcT, flavonoid 3-*O*-glucosyltransferase; 3′GlcT, flavonoid 3′-*O*-glucosyltransferase; 3GalT, flavonoid 3-*O*-galactosyltransferase; 3RhaT, flavonol 3-*O*-rhamnosyltransferase; 5GlcT, flavonoid 5-*O*-glucosyltransferase; 7GlcT, flavonol 7-*O*-glucosyltransferase; NtIS5a, salicylate-induced glucosyltransferase. Abbreviations for species: Ac, *Actinidia chinensis;* At, *Arabidopsis thaliana;* Bp, *Bellis perennis;* Cm, *Citrus maxima;* Db, *Dorotheanthus bellidiformis;* Gt, *Gentiana triflora;* Hv, *Hordeum vulgare;* Ip, *Ipomoea purpurea;* Nt, *Nicotiana tabacum;* Pf, *Perilla frutescens;* Ph, *Petunia hybrida;* Vh, *Verbena hybrida;* Vv, *Vitis vinifera;* Zm, *Zea mays.*

The flavonoid UGT phylogenetic tree indicates that UGT79B1 belongs to a cluster in which UGTs transfer a glycosyl group to a sugar moiety of flavonoid glycosides (Figure 2). UGT79B1 has some amino acid sequence identity with IpA3G2′′GlcT (48%), AcA3Ga2′′XylT (48%), Ph3G2′′RhaT (37%), BpA3G2′′GlcAT (28%), and CmF7G2′′RhaT (27%), all of which are known to catalyze glycosyl transfer to a sugar moiety of flavonoid glycosides (GGT) (Bar-Peled *et al.*, 1991; Brugliera *et al.*, 1994; Kroon *et al.*, 1994; Morita *et al.*, 2005; Sawada *et al.*, 2005; Montefiori *et al.*, 2011). The high correlation coefficients with anthocyanin biosynthetic genes, the anthocyanin substituted patterns found in Arabidopsis (Bloor and Abrahams, 2002) and the primary sequence similarity of UGT79B1 to related genes suggest that UGT79B1 encodes anthocyanin 3-*O*-glucoside: 2′′-*O*-xylosyltransferase.

### Analysis of *ugt79b1* transposon mutants indicates that UGT79B1 encodes anthocyanin 3-*O*-glucoside: 2′′-*O*-xylosyltransferase

**Figure 3:**
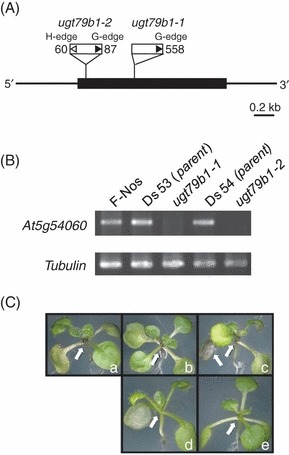
Ds transposon insertion mutants of UGT79B1. (A) Schematic representation of *UGT79B1* with two Ds transposon insertion mutations used in this work. The thick line indicates the coding region and the thinner line indicates the 5′- and 3′-untranslated regions. *UGT79B1* has no introns. White and black triangles show the H-edge and G-edge of the Ds transposon, respectively. Numbers indicate the position of the transposon insertion. (B) Reverse transcription polymerase chain reaction (RT-PCR) analysis of transcripts in wild-type (F-Nos), Ds parental lines (Ds53 and Ds54) and two independent homozygous mutant lines (*ugt79b1-1* and *ugt79b1-2*). (C) Phenotype of the wild-type, F-Nos (a); Ds53 (b); Ds54 (c); *ugt79b1-1* (d); and *ugt79b1-*2 (e). Plants were grown in 12% Suc-containing medium as described in Experimental procedures.

**Figure 4:**
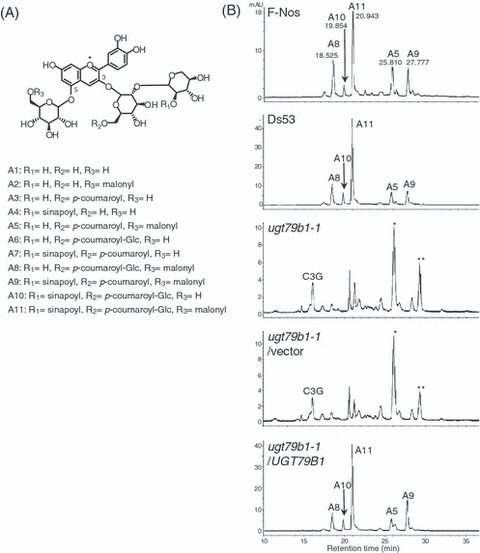
HPLC/PDA/MS analyses of the *ugt79b1* mutant lines. (A) Anthocyanin glycosides accumulated in *Arabidopsis thaliana.* (B) Anthocyanin composition of leaves of wild-type (F-Nos), Ds parental line (Ds 53), *ugt79b1*-deficient mutant (*ugt79b1-1*), *ugt79b1*-deficient mutant complemented with vector only (*ugt79b1-1*/vector) and UGT79B1 cDNA clone (*ugt79b1-1*/*UGT79B1*). C3G, cyanidin 3-*O*-glucoside; *a compound which has the *m/z* value correspond to cyanidin 3-*O*-(6′′-*O*-*p*-coumaroylglucoside)-5-*O*-(6′′-*O*-malonylglucoside) (*m/z* 843, RT 26.09 min); **, a compound which has the *m/z* value correspond to cyanidin 3-*O*-6′′-*O*-*p*-coumaroylglucoside (*m/z* 595, RT 29.26 min).

Homozygotes of two independent *ugt79b1* Arabidopsis transposon insertion lines (Kuromori *et al.*, 2004; Ito *et al.*, 2005), Ds53-4592-1 and Ds54-1263-1, were isolated and designated as *ugt79b1-1* and *ugt79b1-2*, respectively (Figure 3). The transposon was inserted into the exon of *UGT79B1* of both mutants but at the positions +557 (*ugt79b1-1*), and between +61 and +86 base pairs (*ugt79b1-2*) (Figure 3A). No transcripts of *UGT79B1* were detected by reverse-transcription polymerase chain reaction (RT-PCR) in homozygotes of either line (Figure 3B). Seedlings of wild-type and Ds parental lines of *ugt79b1-1* and *ugt79b1-2* accumulated anthocyanins when grown on 12% sucrose (Suc)-containing media. Homozygous insertion lines, *ugt79b1-1* and *ugt79b1-2*, grown on 12% Suc-containing media lacked the purple-coloration phenotype (Figure 3C). The anthocyanin profiles of wild-type, Ds parental lines, *ugt79b1-1* and *ugt79b1-2* were analyzed by high performance liquid chromatography (HPLC)/photodiode array (PDA)/electrospray ionization mass spectrometry (ESI-MS) (Figure 4B). The major anthocyanin A11 was detected in wild-type and Ds parental lines, and A5, A8, A9 and A10 were also detected as minor anthocyanins (Figure 4A). While A5, A8, A9, A10 and A11 were not detected in insertion lines, *ugt79b1-1* and *ugt79b1-2*, total anthocyanin content was reduced to ca. 33% of wild-type. Instead, a compound which has an *m/z* value corresponding with cyanidin 3-*O*-(6′′-*O*-*p*-coumaroylglucoside)-5-*O*-(6′′-*O*-malonylglucoside) (*m/z* 843, RT 26.09 min) was detected as a major peak in addition to cyanidin 3-*O*-glucoside (*m/z* 449, RT 16.07 min). A peak with an *m/z* value corresponding to cyanidin 3-*O*-6′′-*O*-*p*-coumaroylglucoside (*m/z* 595, RT 29.26 min) was also found. *ugt79b1-1* plants were transformed with UGT79B1 cDNA under the control of cauliflower mosaic virus (CaMV) 35S promoter to complement the *ugt79b1-1* mutation. Independent transgenic lines had essentially the same anthocyanin composition as wild-type and Ds parental plants.

### *In vitro* characterization of recombinant UGT79B1

**Figure 5:**
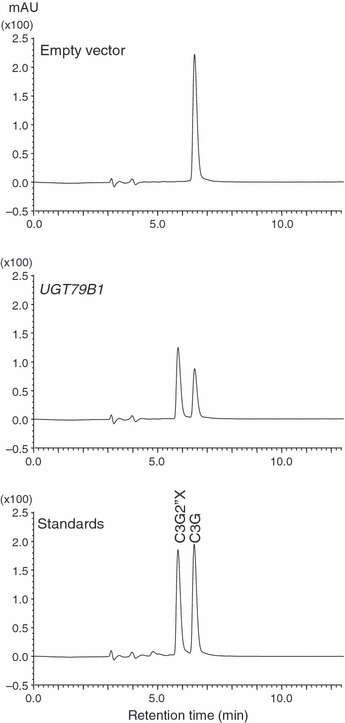
HPLC analyses of the reaction products of UGT79B1 recombinant protein. Elution profile of reaction products of His-tag protein (empty vector) and His-fused UGT79B1 protein (*UGT79B1*) and the standards (cyanidin 3-*O*-glucoside and cyanidin 3-*O*-xylosyl(1→2)glucoside). C3G, cyanidin 3-*O*-glucoside, C3G2′′X, cyanidin 3-*O*-xylosyl(1→2)glucoside.

Recombinant UGT79B1 protein was expressed in *Escherichia coli* as a His-tag fused protein and purified. The His-UGT79B1 protein catalyzed the conversion of cyanidin 3-*O*-glucoside to a single product, cyanidin 3-*O*-xylosyl(1→2)glucoside (Figure 5) as confirmed by retention time and MS/MS spectra. The His-tag alone as a negative control did not catalyze conversion to the 2′′-*O*-xyloside. Thus, UGT79B1 can be defined as an anthocyanin 3-*O*-glucoside: 2′′-*O*-xylosyltransferase.

The specificity of UGT79B1 as a sugar acceptor was also examined. UGT79B1 had significant activity with the 3-*O*-glucoside derivatives of anthocyanidins and flavonols (Table 1). Interestingly, UGT79B1 recognizes cyanidin/kaempferol 3-*O*-rhamnosyl(1→6)glucoside but not cyanidin 3,5-*O*-diglucoside or kaempferol 3-*O*-glucoside-7-*O*-rhamnoside although the latter compounds appear to have more free space around the glucosyl moiety at the C-3 position. UGT79B1 can utilize flavonol 3-*O*-glucosides as substrates, but no flavonol 3-*O*-xylosylglucosides were detected in Arabidopsis seedlings grown in 12% Suc-containing medium.

**Table 1:** Substrate specificity of UGT79B1 from *Arabidopsis thaliana*

Sugar acceptor^a^	Relative activity (%)
Cyanidin 3-*O*-glucoside	100.0 ± 27.5
Cyanidin 3-*O*-rhamnoside	ND
Cyanidin 3-*O*-rhamnosyl(1→6)glucoside	48.1 ± 10.7
Cyanidin 3,5-*O*-diglucoside	ND
Cyanidin 3-*O*-(6′′-*O*-*p*-coumaroylglucoside)-5-*O*-glucoside	ND
Cyanidin 3-*O*-(6′′-*O*-*p*-coumaroylglucoside)-5-*O*-(6′-*O*-malonylglucoside)	ND
Delphinidin 3-*O*-glucoside	28.3 ± 0.8
Pelargonidin 3-*O*-glucoside	48.1 ± 8.8
Kaempferol 3-*O*-glucoside	265.4 ± 34.0
Kaempferol 3-*O*-rhanoside	ND
Kaempferol 3-*O*-rhamnosyl(1→6)glucoside	47.3 ± 2.0
Kaempferol 3-*O*-glucoside-7-*O*-rhamnoside	ND
Quercetin 3-*O*-glucoside	121.6 ± 16.7

Sugar donor^b^	Relative activity (%)
UDP-xylose	100.0 ± 27.5
UDP-arabinose	ND
UDP-galactose	ND
UDP-glucose	ND
UDP-glucuronic acid	ND
UDP-rhamnose	ND

ND, not detected.

a The reactions were performed with UDP-xylose as the sugar donor.

b The reactions were performed with cyanidin 3-*O*-glucoside as the sugar acceptor.

The sugar donor specificity of UGT79B1 was examined with UDP-xylose, UDP-glucose, UDP-arabinose, UDP-glucose, UDP-rhamnose, UDP-galactose and UDP-glucuronic acid as donors and cyanidin 3-*O*-glucoside as acceptor (Table 1). No UGT activity was detected for UDP-sugars other than UDP-xylose, indicating that UGT79B1 is highly specific to UDP-xylose.

### Phylogenetic analyses of the UGTs from six genome-sequenced plants

**Figure 6:**
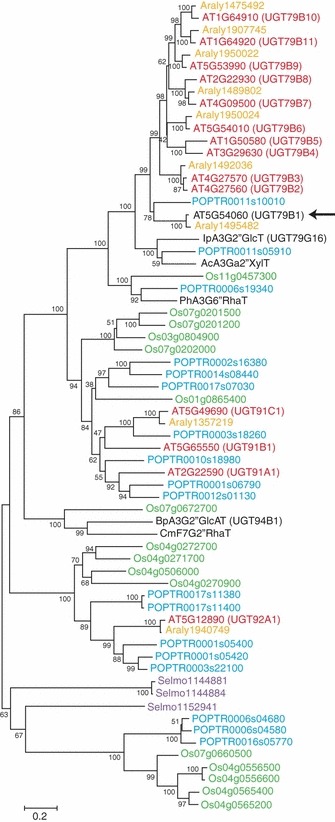
Phylogenetic tree of flavonoid glycosyltransferases in GGT subfamily and UGTs in the orthologous group OG8 from *Physcomitrella patens*, *Selaginella moellendorffii*, *Populus trichocarpa*, *Oryza sativa*, *Arabidopsis thaliana and A.* *lyrata.*The phylogenetic tree for UGTs was generated as described previously (Yonekura-Sakakibara and Hanada, 2011). Number indicates the percentage of replicate trees in which the associated taxa clustered together in the bootstrap test (1000 replicates). Some UGTs from the six plants in OG8 were omitted. A3G2′′GlcAT, anthocyanidin 3-*O*-glucoside 2′′-*O*-glucuronosyltransferase; A3G2′′GlcT, anthocyanidin 3-*O*-glucoside 2′′-*O*-glucosyltransferase; A3G6′′RhaT, anthocyanidin 3-*O*-glucoside 6′′-*O*-rhamnosyltransferase; A3Ga2′′XylT, anthocyanidin 3-*O*-galactoside 2′′-*O*-xylosyltransferase; F7G2′′RhaT, flavanone 7-*O*-glucoside 2′′-*O*- rhamnosyltransferase; Abbreviations for species: Ac, *Actinidia chinensis;* At, *Arabidopsis thaliana;* Bp, *Bellis perennis;* Cm, *Citrus maxima;* Ip, *Ipomoea purpurea;* Ph, *Petunia hybrida.*

To assess the origin of GGTs from a broader perspective, we conducted phylogenetic analyses of the UGTs from six genome-sequenced plants (*Physcomitrella patens*, *Selaginella moellendorffii*, *Populus trichocarpa*, *Oryza sativa*, *Arabidopsis thaliana and A.* *lyrata*) in addition to known flavonoid GGTs. Plant UGTs fell into 24 orthologous groups (OGs) that contained genes derived from a common ancestor of these six species (Yonekura-Sakakibara and Hanada, 2011). All known flavonoid GGTs belong to an orthologous group (OG8), suggesting that flavonoid GGTs are derived from a common ancestral gene (Figure 6). Unfortunately, the functions of other UGTs in this orthologous group have remained elusive. However, a furofuran lignan GGT (UGT94D1), which glucosylates at the 6′-hydroxyl group of the sugar moiety of (+)-sesaminol 2-*O*-glucoside, have been isolated from *Sesamum indicum* (Noguchi *et al.*, 2008). UGT94D1 cannot utilize flavonoid glycosides as substrates, but belongs to the same UGT94 family with BpA3G2′′GlcAT. This finding suggests that GGT function including a recognition mechanism for the hydroxyl group of the sugar moiety was established before the divergence of UGT94s, UGT91s and UGT79s and only then acquired the ability to specify substrates (sugar acceptors and sugar donors) and regiospecificity. Functional identification of other UGTs in OG8 will provide useful information for UGT evolution in terms of acquiring substrate specificity.

### UGT84A2 supplies sinapoylglucose for anthocyanin modification

By the hierarchical clustering of genes based on ICA, UGT84A2 was localized to sub-cluster 2, which is involved in anthocyanidin skeleton biosynthesis. It has been reported that UGT84A2 encodes UDP-glucose: sinapic acid glucosyltransferase required for the biosynthesis of 1-*O*-sinapoylglucose (Lim *et al.*, 2001; Sinlapadech *et al.*, 2007). 1-*O*-sinapoylglucose is utilized as an acyl donor by serine carboxypeptidase-like acyltransferases including malate sinapoyltransferase and choline sinapoyltransferase (Lehfeldt *et al.*, 2000; Shirley *et al.*, 2001). In ugt84a2 knockout mutants, sinapoylmalate content was slightly decreased and other sinapoylated/feruloyated compound contents were altered compared to wild-type (Sinlapadech *et al.*, 2007; Meissner *et al.*, 2008). Anthocyanin sinapoyltransferase (SAT, At2g23000) also belong to serine carboxypeptidase-like acyltransferases, suggesting that 1-*O*-sinapoylglucose produced by UGT84A2 may be a major source of sinapoyl groups to anthocyanin sinapoyltransferase. However, there is no direct evidence for a relationship between anthocyanin biosynthesis and UGT84A2 *in planta.*

### Analysis of *ugt84a* mutants supports the predominant involvement of UGT84A2 in anthocyanin acylation

**Figure 7:**
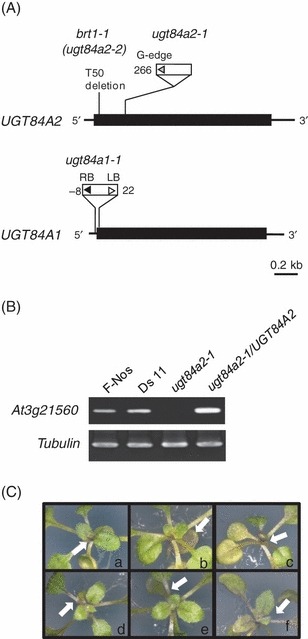
*ugt84a* mutants and characterization of *ugt84a* mutations on anthocyanin accumulation. (A) Schematic representation of *UGT84A2* with a Ds transposon insertion mutation and *UGT84A1* with a T-DNA insertion mutation used in this work. The thick line indicates the coding region and the thinner line indicates the 5′- and 3′-untranslated regions. *UGT84A2* and *UGT84A1* have no introns. Gray triangle shows the G-edges of the Ds transposon. Black and white triangles show right and left borders of T-DNA, respectively. Numbers indicate the position of the insertion. (B) RT-PCR analysis of transcripts in wild-type (F-Nos), Ds parental line (Ds11) and an independent homozygous mutant line (*ugt84a2-1)* and the *ugt84a2*-deficient mutant complemented with the UGT84A2 cDNA clone (*ugt84a2-1/*UGT84A2). (C) Phenotype of the wild-type, F-Nos (a); Ds 11 (b); *ugt84a2-1* (c); Col-0 (d); *ugt84a2-2* (e); and *ugt84a1-1* (f). Plants were grown in 12% Suc-containing medium as described in Experimental procedures.

A homozygote of an ecotype Nossen transposon insertion line, Ds11-5836-1, was isolated and designated as *ugt84a2-1.* The G-edge of the transposon was inserted into the exon of the mutant at position +266 (Figure 7A), creating a null mutation (Figure 7B). When grown on 12% Suc-containing medium, *ugt84a2-1* seedling pigment phenotype was the same as the Ds parental and wild-type lines (Figure 7C).

**Figure 8:**
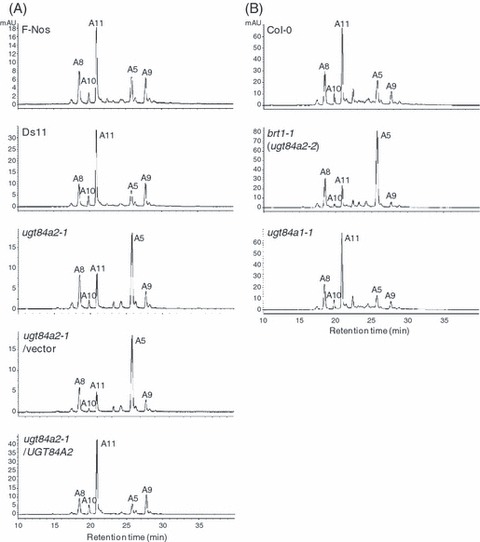
HPLC/PDA/MS analyses of the *ugt84a2* mutant lines. (A) Anthocyanin composition of leaves of wild-type (F-Nos), Ds parental line (Ds 11), *ugt84a2*-deficient mutant (*ugt84a2-1*), *ugt84a2*-deficient mutant complemented with vector only (*ugt84a2-1*/vector) and UGT84A2 cDNA clone (*ugt84a2-1*/*UGT84A2*). (B) Anthocyanin composition of leaves of wild-type (Col-0), *ugt84a2*-deficient mutant (*brt1-1*, *ugt84a2-2*) and *ugt84a1*-deficient mutant (*ugt84a1-1*).

The anthocyanins of wild-type, Ds parental line Ds11 and *ugt84a2-1* were analyzed by HPLC/PDA/ESI-MS (Figure 8). In wild-type and Ds parental lines, A11 was detected as a major anthocyanin (>50% of total anthocyanins), and A5, A8, A9 and A10 were detected as minor peaks (5–20% of total anthocyanins). In *ugt84a2-1*, A11 made up only about 20% of the total anthocyanins and A5 accounted for 40–50%, although there is no significant change in total anthocyanin contents. The A11 levels of *ugt84a2* knockout mutants were reduced to approximately 25% of wild-type. Compared with the impact on other sinapoylated compounds such as sinapoylmalate and sinapoylcholine (60–70% of wild-type) (Sinlapadech *et al.*, 2007; Meissner *et al.*, 2008), the effect on A11 content was more severe. Other sinapoylated anthocyanins (A9 and A10) were largely unaffected. To determine if the changes in anthocyanin composition can be ascribed to the *UGT84A2* mutation, *ugt84a2-1* plants were transformed with UGT84A2 cDNA under the control of CaMV 35S promoter. Independent complemented *UGT84A2* transgenic lines had substantially the same anthocyanin composition as wild-type and Ds parental plants (Figure 8A). These data indicate that 1-*O*-sinapoylglucose produced by UGT84A2 is a significant source of sinapoyl moieties for anthocyanins, and that the limited supply of 1-*O*-sinapoylglucose affects anthocyanin composition, reducing the content of sinapoylated anthocyanin, A11.

UGT84A2 and closely-related UGTs (UGT84A1 and UGT84A3) have significant UDP-glucose: sinapic acid glucosyltransferase activity, but the specific activities of both UGT84A1 and UGT84A3 for sinapic acid are nearly half that of UGT84A2 (Lim *et al.*, 2001). *UGT84A2* showed the highest correlation coefficients with *UGT84A1* (*r* = 0.403, ATTED-II, ver.3) among *UGT84A*s (*UGT84A3*, *r* = 0.077, *UGT84A4*, *r* = 0.144). To investigate the contribution of other UGT84As to anthocyanin composition, we also isolated a homozygous T-DNA insertion mutant of *UGT84A1* (GABI_765F10) designated as *ugt84a1-1* (Figure 7A). To compare phenotypes, we used another knockout mutant of *UGT84A2* with a Col-0 background, *brt1-1* (*ugt84a2-2*), because *ugt84a1-1* has a Col-0 background. When grown on 12% Suc-containing media, *ugt84a1-1* seedlings showed a purple-color phenotype that was indistinguishable from wild-type (Col-0) and *brt1-1* (Figure 7C). We analyzed the anthocyanin profiles of *ugt84a1-1* and *brt1-1* (*ugt84a2-2*) (Figure 8B). The *brt1-1* (*ugt84a2-2*) mutant in a Col-0 background had a similar anthocyanin profile to *ugt84a2-1.* However, no significant change of the anthocyanin profile in *ugt84a1-1* was observed compared with wild-type (Col-0). These data indicate that UGT84A2 is a major supplier of 1-*O*-sinapoylglucose for anthocyanin modification. The predominance of A11 in *ugt84a2* knockout mutants from different backgrounds suggests that other UGT84As also contribute the production of 1-*O*-sinapoylglucose, but to a much lesser extent.

## Discussion

### Flavonoid UGTs which glycosylate the sugar moiety attached to flavonoid aglycones

The functional identification of UGT79B1 allowed us to compare flavonoid GGTs that glycosylate the sugar moiety attached to flavonoid aglycones. Generally, flavonoid UGTs form a unique cluster based on their regiospecificity for sugar acceptors (*i.e.* the glycosylation position of sugar acceptors). Furthermore, in the case of UGT, which glycosylates at the C-3 position of flavonoids (3GT), the phylogenetic tree indicates that the function of 3GT was established before the divergence of monocots and dicots, and the specificity of sugar donors was afterward (Figure 2). However, no such systematic phylogenetic trace was found in the GGTs. IpA3G2′′GlcT (UGT79G16) is distant from Ph3G6′′RhaT although they are from the same order (*Solanales*). Anthocyanin 3-*O*-galactoside: 2′′-*O*-xylosyltransferase from kiwifruit (*Actinidia chinensis*, AcA3Ga2′′XylT), recognizes cyanidin 3-*O*-glucoside and UDP-xylose like UGT79B1 does. However, AcA3Ga2′′XylT had higher similarity with IpA3G2′′GlcT, which uses UDP-glucose as a sugar donor (63%), than with UGT79B1 (48%). Plant UGTs have a carboxyl-terminal consensus sequence of 44 amino acid residues termed the plant secondary product glycosyltransferase (PSPG) box (Mackenzie *et al.*, 1997; Paquette *et al.*, 2009). The PSPG box is thought to be involved in binding to the UDP moiety of the sugar nucleotide (Mackenzie *et al.*, 1997). The PSPG box of UGT79B1 showed higher sequence identity with that of IpA3G2′′GlcT (68%) than with that of AcA3Ga2′′XylT (59%), although both UGT79B1 and AcA3Ga2′′XylT recognize UDP-xylose.

Phylogenetic comparisons of flavonoid GGTs, including predicted common ancestral UGTs at nodes 7–10 suggest possible conserved amino acid residues involved in recognizing UDP-xylose and anthocyanin 3-*O*-glucosides (Figure S2). Ancestral sequences for four nodes (node 7 to node 10) were inferred by a maximum likelihood method (PAML: Phylogenetic Analysis by Maximum Likelihood, ver. 4.3). We assumed that the occurrence of enzymatic divergence was due to amino acid replacements, and ancestral GGT(s) obtained the ability to recognize UDP-xylose at the branch of node7 to node 8, and was preserved in the two branches (node 8 to node 9, and node 9 to AcA3Ga2′′XylT), but was lost at node 9 to Ip3G2′′GlcT. Following this simple assumption, amino acid residues involved in recognition of the C-2′′ position of anthocyanin 3-*O*-glucoside are expected to be conserved in all divergences except for two branches (node 10 to BpA3G2′′GlcAT and node 7 to PhA3G6′′RhaT). The multiple alignment of flavonoid GGTs shows that most amino acid residues conserved in flavonoid GGTs are common to other known flavonoid UGTs, and Met16 is clearly specific to flavonoid GGTs (Figure S2). In general, UGTs belong to the GT-B fold with two Rossmann-like domains (Coutinho *et al.*, 2003), and the crystal structures of plant UGTs (grape VvGT1, UGT71G1 and UGT85H2 from *Medicago truncatula*) have been determined (Shao *et al.*, 2005; Offen *et al.*, 2006; Li *et al.*, 2007). Protein modeling and site-directed mutagenesis of BpA3G2′′GlcAT suggest that N123 and D152 are key residues for recognition of cyanidin 3-*O*-glucoside (Osmani *et al.*, 2008). However, the residues are not conserved in other flavonoid GGTs. Crystallization of UGT79B1 would be required for precise determination of the amino acid residues involved in substrate recognition because of low sequence identity between plant UGTs.

### Anthocyanin modification pathway forms metabolic grids in Arabidopsis

**Figure 9:**
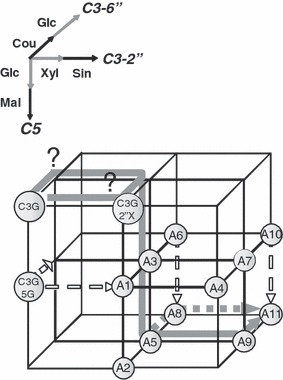
Possible metabolic route of anthocyanin modification pathway in Arabidopsis. The metabolic grid was constructed by expanding that of anthocyanin acylation in Fraser *et al.*, 2007;. Thin black/gray arrows indicate potential glucosylation (Glc), xylosylation (Xyl), coumaroylation (Cou), sinapoylation (Sin) and malonylation (Mal). Gray thick arrows indicate putative major anthocyanin modification routes. Open dotted arrows mean unlikely routes based on the corresponding enzymatic property. The structures of anthocyanins (A1–A11) are shown in Figure 5A. C3G; cyanidin3-*O*-glucosdie, C3G5G; cyanidin 3,5-*O*-diglucosides, C3G2′′X; cyanidin 3-*O*-xylosyl(1→2)glucoside.

In Arabidopsis, it has been estimated that the anthocyanin modification pathway forms a metabolic grid as proposed for *Perilla frutescens* and *Gentiana triflora* (Yamazaki *et al.*, 1999; Fukuchi-Mizutani *et al.*, 2003; Fraser *et al.*, 2007). Enzymatic characterization of anthocyanin xylosyltransferase and of the anthocyanin profile in *ugt84a2*, together with previous reports, provides a clue as to the likely major routes of anthocyanin modification (Figure 9).

UGT79B1 recognizes cyanidin 3-*O*-glucoside and cyanidin 3-*O*-rhamnosyl(1→6)glucoside as substrates, but not cyanidin 3,5-*O*-diglucoside. Other UGTs which catalyze glycosylation at the sugar moiety of flavonoid glycosides also show considerable activity toward anthocyanin 3-*O*-glucosides, but no or negligible activity toward anthocyanin 3,5-*O*-diglucosides (Morita *et al.*, 2005; Sawada *et al.*, 2005). Arabidopsis anthocyanin coumaroyltransferase, which transfers a coumaroyl moiety to the C-6′′ position of cyanidin 3-*O*-glucoside prefers cyanidin 3-*O*-glucoside, but has negligible activity for cyanidin 3,5-*O*-diglucosides (Luo *et al.*, 2007). These data imply that xylosylation and coumaroylation at C-2′′ and C-6′′ of cyanidin 3-*O*-glucoside, respectively, occur prior to glucosylation at the C-5 position. It has been reported that acylation with a coumaroyl moiety is effective for anthocyanin stability (Luo *et al.*, 2007). On the other hand, acylated anthocyanin 3-*O*-glucosides are unstable, but are the best substrates for anthocyanin 3-*O*-glucoside 2′′-*O*-xylosyltransferase in *Matthiola incana* R.Br (Teusch, 1986). Unfortunately, acylated anthocyanin 3-*O*-glucosides are not commercially available. The substantial anthocyanin reduction observed in *ugt79b1* mutants suggests that 2′′-*O*-xylosylation is crucial for stable accumulation of anthocyanin in Arabidopsis.

In *ugt84a2* knockout mutants, A5 accumulated as a major anthocyanin instead of A11. The content of sinapoylated anthocyanin A9 and A10, and the non-sinapoylated anthocyanin A8 showed no significant change. This finding suggests that anthocyanin A11 may be mainly produced from A5 via A9. Interestingly, in an *sng1-5* mutant that lacks anthocyanin sinapoyltransferase, A8 and A5 accumulated as major anthocyanins (Fraser *et al.*, 2007). The differences in accumulated anthocyanins in these mutants suggest that the affinity of A5 for anthocyanin sinapoyltransferase may be higher than for anthocyanin coumaroyltransferase. Anthocyanin sinapoyltransferase may hold A5 in *ugt84a2* mutants and thus inhibit further modification.

### Independent component analysis provides a different perspective on microarray data analysis by IC numbers

The hierarchical clustering of a gene signature matrix with a total of 1877 metabolism-related genes based on eight ICs formed clear clusters of genes involved in the biosynthesis of anthocyanins and flavonols, which are slightly different from those formed by transcriptome coexpression analysis. Among the algorithms with various numbers of ICs we applied, the closest anthocyanin/flavonol clusters were observed based on eight ICs. Fukushima *et al.* proposed that a small number of samples (∼20) are enough to find coexpression linkage (Fukushima *et al.*, 2008). Kinoshita and Obayashi examined principal component analysis (PCA) for identifying the major factors of gene expression correlation, and found the contribution of the first 10 principal components (PCs) to be enough to describe 80% of the variation within the 1388 samples of ATTED-II (Kinoshita and Obayashi, 2009).

Interestingly, the cluster that flavonoid 3-*O*-glucosyltransferase (Fd3GlcT; UGT78D3, At5g17050) belongs to changes depending on the number of ICs. For example, Fd3GlcT falls into the flavonol cluster based on 8 ICs, but into the anthocyanidin modification sub-cluster when based on 10 ICs (data not shown). Further, using only simple correlation coefficients, Fd3GlcT, which can recognize both flavonols and anthocyanidins as substrates, localizes to a flavonol gene cluster, but not to an anthocyanin group (Yonekura-Sakakibara *et al.*, 2007). In addition, UGT84A2 also plays an important role for other sinapoyltransferases, but no correlation with other sinapoyltransferase genes was found in this study. These data suggest that transcriptome analyses using ICA should specify optimum IC numbers for each metabolic pathway. Additionally, bi-functional genes may become apparent by fine adjustment of IC number. For example, eight ICs may be most suitable for analysis of anthocyanin pathway, as demonstrated by the dual role of flavonoid 3-*O*-glucosyltransferase. Recently, novel acyl-glucose-dependent anthocyanin glucosyltransferases, which belong to glycoside hydrolase family 1, but not UGT, were isolated from carnation (*Dianthus caryophyllus)* and delphinium (*Delphinium grandiflorum*) (Matsuba *et al.*, 2010). The application of ICA in various plant species thus might also be useful for finding ‘unexpected’ genes.

## Experimental Procedures

### Plant materials

*A.* *thaliana* accession Columbia-0 (Lehle Seeds), or accession Nossen (Fedoroff and Smith, 1993)] were used as wild-types in this study. The mutant *brt1-1* (*ugt84a2-2*) was described previously (Sinlapadech *et al.*, 2007). The Arabidopsis transposon-tagged lines Ds53-4592-1 and Ds54-1263-1 for UGT79B1 (*ugt79b1-1* and *ugt79b1-2*, respectively), and Ds11-5836-1 for UGT84A2 (*ugt84a2-1*) were obtained from RIKEN Bioresource Center. The T-DNA-insertion mutant *GABI_765F10* for UGT84A1 (*ugt84a1-1*) was obtained from the Arabidopsis Biological Resource Center. Homozygous knockout lines were screened by PCR using specific primers for *UGT79B1*, *UGT84A1*, *UGT84A2*, Ds transposon and T-DNA: UGT79B1f, UGT79B1r, UGT84A1f, UGT84A1r, UGT84A2f, UGT84A2r, Ds5-2a, Ds3-2a, o8409, o3144 (see Table S1). PCR products were sequenced to determine the exact insertion points.

For analyses of anthocyanin accumulation, plants were cultured on one-half-strength MS-agar medium containing 1% sucrose (Valvekens *et al.*, 1988) in a growth chamber at 22°C with 16 h/8 h light and dark cycles for 14 days with a light intensity of 40 μmol of photons m^−2^ s^−1^, then transferred on one-half-strength MS-agar medium containing 12% sucrose for 3 days with a light intensity of 80 μmol of photons m^−2^ s^−1^. Plants were harvested, immediately frozen with liquid nitrogen, and stored at −80°C until use. At least three biological replicates were used for anthocyanin analysis.

### Chemicals

Chemicals of the highest grade commercially available were used unless specifically noted. Flavonoid standards were purchased from Extrasynthese and AnalytiCon. UDP-β-l-arabinose and UDP-α-d-xylose were purchased from CarboSource Services (supported in part by National Science Foundation-Plant Cell Wall Biosynthesis Research Network grant 0090281).

### Independent component analyses

ICA is based on the assumption that a given gene expression level is determined by a linear combination of some independent components corresponding to biological signals. Assuming that an expression data matrix could be denoted as an *m* × *n* matrix *X* with rows and columns representing *m* genes and *n* samples, respectively, and could be considered to be a linear combination of ICs (i.e. *m* × *k* gene signature matrix *S*), we can describe *X* = *SA* where *A* denotes a latent mixing matrix (*k* × *n* latent vectors of the gene expression data) (Figure S1). Here we assumed *k* ICs. Rows of *S* (i.e. ICs) are statistically independent from each other in ICA. ICA was carried out using fastICA algorithm, which is based on a fixed point algorithm for seeking a maximum of non-Gaussian properties of the components (Hyvärinen and Oja, 1997), with statistical R package ‘fastICA’. Pre-processed expression data, ‘GeneExp_v3’ file, from ATTED-II website (http://atted.jp/) consisting of 1388 Affymetrix ATH1 GeneChips were used for the analyses. These data were originally from TAIR AtGenExpress and were normalized by robust multichip average (Irizarry *et al.*, 2003). For simplicity, we selected genes associated with AraCyc metabolic pathways using flat file, http://ftp://ftp.arabidopsis.org/Pathways/OLD/aracyc_dump.20091014. The array element mappings of Affymetrix probe set identifiers to AGI locus table from TAIR dated 29 July 2009 (affy_ATH1_ array_elements-2009-7-29.txt) was used. The resulting matrix size is 1388 samples × 1877 genes. After applying the fastICA algorithm with *k* components to be extracted (in this study, *k* = 8), the data were subjected to hierarchical cluster analysis (HCA) with the ICs using correlation (uncentered) and average linkage methods. The HCA was performed by Cluster 3.0 (de Hoon *et al.*, 2004) and was visualized by Java TreeView (http://jtreeview.sourceforge.net/).

### Phylogenetic analysis

UGT protein sequences were aligned by ClustalW implemented in mega4 (version 4.02; http://www.megasoftware.net/) (Tamura *et al.*, 2007). A phylogenetic tree was constructed with the aligned UGT protein sequences by MEGA4 using the neighbor-joining method (Saitou and Nei, 1987) with the following parameters: Poisson correction, complete deletion, and bootstrap (1000 replicates, random seed = 64238). The alignment data are available in the Supplementary material online (Data S2).

### Anthocyanin profiling by HPLC/PDA/ESI–MS

Anthocyanin extraction was carried out in triplicate as described previously (Tohge *et al.*, 2005). For anthocyanin profiling, Agilent HPLC 1100 series and Agilent single quadrupole LC-MS 6120 series (Agilent Technologies Inc., http://www.home.agilent.com/) were used with an Atlantis® T3 column (Φ4.6 mm × 150 mm, 5 μm, Waters) at a flow rate of 0.5 ml min^−1^ at 30°C. Anthocyanins were separated with solvent A (10% acetonitrile, 0.1% trifluoroacetic acid in water) and solvent B (90% acetonitrile, 0.1% trifluoroacetic acid in water) using an elution gradient (0 min, 0% B; 40 min, 40% B, 40.1 min, 100% B; 45 min 100% B; 45.1 min, 0% B; 50 min, 0% B). PDA was used for the detection of UV-visible absorption in the range of 200–600 nm. A mass analyzer was used for the detection of anthocyanin glycosides [M]^+^, and the peak of fragment ions in a positive ion scanning mode with the following setting: drying gas temperature, 350°C with drying gas flow of 12 L/min; capillary voltage, 4.0 kV; nebulizer pressure, 35 psig; fragmentor, 80 V; detection mode, scan (*m/z* 100–1400).

### Evaluation of T-DNA and transposon insertion mutants

For complementation tests, the full-length *UGT79B1* coding region was amplified by PCR using the primers UGT79B1-GWf and UGT79B1-GWf (Table S1). A full-length cDNA clone of *UGT84A2* (pda08060) was obtained from the RIKEN BioResource Center Arabidopsis full-length cDNA collection (Seki *et al.*, 1998, 2002). The full-length *UGT84A2* was amplified by PCR using the primers UGT84A2-GWf and UGT84A2-GWf (Table S1). Amplified fragments were cloned into the pENTR/D-TOPO vector (Invitrogen, http://www.invitrogen.com/) as an entry vector and sequenced to confirm the absence of PCR errors. pB2GW7 was used as a destination vector and the LR reactions for the binary vector pKYS390 for UGT79B1 and pKYS399 for UGT84A2 were catalyzed by the Gateway LR clonase enzyme mix (Invitrogen). Transformation into *Agrobacterium* and Arabidopsis, and the selection of transformants were carried out as described previously (Yonekura-Sakakibara *et al.*, 2007).

For analyses of anthocyanin accumulation, plants were cultured on one-half-strength MS-agar medium containing 1% sucrose (Valvekens *et al.*, 1988) in a growth chamber at 22°C with 16 h/8 h light and dark cycles for 14 days with a light intensity of 40 μmol of photons m^−2^ s^−1^, then transferred to one-half-strength MS-agar medium containing 12% sucrose for 3 days with a light intensity of 80 μmol of photons m^−2^ s^−1^. Plants were harvested, immediately frozen with liquid nitrogen, and stored at −80°C until use. At least three biological replicates were used for anthocyanin analysis.

### General molecular procedures

The molecular procedures used were as described previously (Yonekura-Sakakibara *et al.*, 2004) unless otherwise specified. RT-PCR was performed as described previously (Yonekura-Sakakibara *et al.*, 2004) with primers UGT79B1-RTf and UGT79B1-RTr for *UGT79B1*, UGT84A2-RTf and UGT84A2-RTr for *UGT84A2* and TUBf and TUBr for tubulin (GenBank™ Accession number AK117431) (Table S1).

### Production of recombinant UGT79B1 protein and glycosyltransferase assays

Full-length *UGT79B1* was amplified by PCR with the primers UGT79B1-IFf and UGT79B1-IFr to construct a protein expression vector (Table S1). The PCR product was cloned into pCFinf using an In-Fusion Advantage PCR cloning kit (Clontech, http://www.clontech.com/). The nucleotide sequence of the resultant plasmid, pKYS398, was confirmed as above. *Escherichia coli* strain KRX (Promega, http://www.promega.com/) was used as a host for expression of recombinant UGT79B1 protein. Transformed cells were grown at 37°C until A_600_ reached 0.5. After the addition of 20% (w/v) rhamnose to a final concentration of 0.1% (w/v), cells were cultured at 18°C for 24 h. The cells were collected, and the protein was purified as a His fusion according to the manufacturer’s instructions.

The standard enzyme assay reaction mixture (final volume, 50 μl) consisted of 50 mm HEPES-KOH, pH 7.5, 150 μm flavonoid substrates, and 500 μm UDP-sugar. For enzyme assays with anthocyanins as substrates, β-mercaptoethanol was added to a final concentration of 5 mm. The mixture was preincubated at 30°C for 2 min, and the reaction was started by the addition of enzyme. Reactions were stopped after 0, 4, 8, 12, or 30 min of incubation at 30°C by the addition of 50 μl ice-cold 0.5% (v/v) trifluoroacetic acid/MeOH for flavonols or 50 μl ice-cold 1.0% (v/v) HCl/MeOH for anthocyanidins and anthocyanins. Supernatants were recovered by centrifugation at 12 000 ***g*** for 3 min. Flavonoids in the resultant solution were analyzed using a Shimadzu HPLC system with a Unison UK-C18 column (2.0 × 150 mm, 3 μm; Imtakt corporation, http://www.imtaktusa.com/) at a flow rate of 0.2 ml/min at 35°C. Compounds were separated with a linear eluting gradient with solvent A (0.5% trifluoroacetic acid in water) and solvent B (0.5% trifluoroacetic acid in acetonitrile) set according to the following profile: 0 min, 20% B; 5 min, 20% B; 10 min, 22% B;10.1 min, 100% B; 15 min, 100% B; 15.1 min, 20% B; 20 min, 20% B. PDA was used for the detection of UV-visible absorption in the range of 200–600 nm.

## SUPPORTING INFORMATION

**Figure S1.** A schematic ICA model of gene expression data as a strategy for gene discovery.

**Figure S2.** Phylogenetic tree and multiple alignment of flavonoid UGTs catalyzing glycosyl transfer to a sugar moiety of flavonoid glycosides.

**Table S1.** Primers used in this study.

**Data S1.** Complete hierarchical clustering data of a gene signature matrix with 1877 metabolism-related genes based on 8 ICs. Java Treeview (http://jtreeview.sourceforge.net) is required for visualization.

**Data S2.** The alignment used for construction of the phylogenetic tree shown in Figure 2.

Please note: As a service to our authors and readers, this journal provides supporting information supplied by the authors. Such materials are peer-reviewed and may be re-organized for online delivery, but are not copy-edited or typeset. Technical support issues arising from supporting information (other than missing files) should be addressed to the authors.
