# Supplementary material for: Two glycosyltransferases involved in anthocyanin modification delineated by transcriptome independent component analysis in Arabidopsis thaliana
Source: Plant J. 2011 Oct 14;69(1):154–67. doi: 10.1111/j.1365-313X.2011.04779.x (PMC3507004; doi:10.1111/j.1365-313X.2011.04779.x)
Supplement: Supplementary file 3 [file tpj0069-0154-SD9.doc]

**Supplemental Table 1.** Primers used in this study

primer name sequence

UGT79B1f 5’- ACCAGGAGGTTACACTTGCCCGGTC-3’

UGT79B1r 5’-TTTGAGTAGGGAAGATTTAACTCT-3’

UGT84A2f 5’-CTTCTACCATCCAACAAGTC-3’

UGT84A2r 5’-CATTTTGTAGAGTGGTCCTAGC-3’

UGT84A1f 5’-TGAACCTAGAAGAAGGATGGAGAA -3’

UGT84A1r 5’-AAGCGAAATCTTGAGCTGAACGTG-3’

Ds5-2a 5’-TCCGTTCCGTTTTCGTTTTTTAC-3’

Ds3-2a 5’-CCGGATCGTATCGGTTTTCG-3’

o8409 5’-ATATTGACCATCATACTCATTGC-3’

o3144 5’-GTGGATTGATGTGATATCTCC-3’

UGT79B1-GWf 5’-CACCATGGGTGTTTTTATCG-3’

UGT79B1-GWr 5’- AACTCATGACTTCACAAGTTCA-3’

UGT84A2-GWf 5’-CACCATGGAGCTAGAATCTTCTCC -3’

UGT84A2-GWr 5’- GACTTTTTAAAAGCTTTTGATTGATCC-3’

UGT79B1-IFf 5’-CAGCGGCTCCTCGGGA ATGGGTGTTTTTGGATGCAATG -3’

UGT79B1-IFr 5’-TCAATCAATCATTAGTTATCATGACTTCACAAGTTCAATTAAA -3’

UGT79B1-RTf 5’-ATGGGTGTTTTTGGATCGAATG-3’

UGT79B1-RTr 5’-AACTCATGACTTCACAAGTTCA-3’

UGT84A2-RTf 5’-TGGAAAGGCTATCTCCGGTATG -3’

UGT84A2-RTr 5’- CATTTTGTAGAGTGGTCCTAGC-3’

TUBf 5’-CCTGATAACTTCGTCTTTGG-3’

TUBr 5’-GTGAACTCCATCTCGTTCA-3’
