# Supplementary material for: Two glycosyltransferases involved in anthocyanin modification delineated by transcriptome independent component analysis in Arabidopsis thaliana
Source: Plant J. 2011 Oct 14;69(1):154–67. doi: 10.1111/j.1365-313X.2011.04779.x (PMC3507004; doi:10.1111/j.1365-313X.2011.04779.x)
Supplement: Supplementary file 4 [file tpj0069-0154-SD2.doc]

**Supplemental Data Set 1.** Complete hierarchical clustering data of a gene signature matrix with 1877 metabolism-related genes based on 8 ICs. Java Treeview ([http://jtreeview.sourceforge.net](http://jtreeview.sourceforge.net/)) is required for visualization.

GENE NAME IC1 IC2 IC3 IC4 IC5 IC6 IC7 IC8

At2g26450 pectinesterase family protein -0.164575925 0.660529216 0.249294835 0.317317626 0.354977763 -0.372158623 4.509525356 -0.192162068

At2g26480 "UGT76D1 (UDP-GLUCOSYL TRANSFERASE 76D1); UDP-glycosyltransferase/ quercetin 7-O-glucosyltransferase/ transferase, transferring glycosyl groups" 0.169785106 0.813180191 0.020127835 0.515863628 0.12091481 -0.675680426 0.001021192 -0.086302627

At2g39770 EMB101_GMP1_SOZ1_VTC1__CYT1 (CYTOKINESIS DEFECTIVE 1); mannose-1-phosphate guanylyltransferase/ nucleotidyltransferase 0.203172006 0.318423703 0.366460323 0.427689097 -0.669859384 -0.837056693 0.765037 0.476767091

At2g23260 "UGT84B1 (UDP-glucosyl transferase 84B1); UDP-glycosyltransferase/ abscisic acid glucosyltransferase/ indole-3-acetate beta-glucosyltransferase/ quercetin 7-O-glucosyltransferase/ transferase, transferring glycosyl groups" -0.082379769 0.054072308 -0.021656963 -0.129471305 -0.193834044 0.233950956 -0.102882179 -1.403235365

At2g23250 "UGT84B2 (UDP-glucosyl transferase 84B2); UDP-glycosyltransferase/ abscisic acid glucosyltransferase/ transferase, transferring glycosyl groups" 0.050448111 0.297135441 0.310322702 0.378832256 0.122379551 -0.363652234 0.302475415 -0.297045895

At2g23210 "UDP-glycosyltransferase/ transferase, transferring glycosyl groups" 0.0643551 0.285252793 0.341739161 0.350778327 0.26393475 -0.301232186 0.180487002 -0.358297189

At2g23170 GH3.3; indole-3-acetic acid amido synthetase 4.158590463 -0.455279263 -1.912955345 1.740416843 -0.585548543 -2.240860412 -1.267084167 -0.195498138

At2g45290 "transketolase, putative" 0.019195692 1.787459441 -0.785571069 -0.268651271 -0.467118538 0.339590021 0.419781767 -1.458410065

At2g40840 DPE2 (DISPROPORTIONATING ENZYME 2); 4-alpha-glucanotransferase/ heteroglycan binding -0.081374239 -0.477665414 0.009037241 0.156314718 2.748570459 1.282609923 0.095176411 0.369026107

At2g40890 "CYP98A3 (cytochrome P450, family 98, subfamily A, polypeptide 3); monooxygenase/ p-coumarate 3-hydroxylase" 0.236543408 0.62879856 0.211052279 -0.811278932 -1.481081593 -0.174459719 -1.289694072 -0.787859941

At2g41510 ATCKX1__CKX1 (CYTOKININ OXIDASE/DEHYDROGENASE 1); cytokinin dehydrogenase 0.229053632 0.708718672 0.485356399 0.134221199 0.189729367 -0.209044554 -0.091093735 0.030799681

At2g41530 "AtSFGH__SFGH (S-FORMYLGLUTATHIONE HYDROLASE); S-formylglutathione hydrolase/ hydrolase, acting on ester bonds" -0.033505077 -0.162583287 0.087668117 -0.453970283 0.75267945 0.599103127 -0.93823364 0.045765584

At2g41680 NTRC (NADPH-DEPENDENT THIOREDOXIN REDUCTASE C); thioredoxin-disulfide reductase -0.618673997 -1.801866069 0.690504882 0.610367454 -0.578340958 0.263136691 -0.905355742 0.258464527

At2g47460 ATMYB12_PFG1__MYB12 (MYB DOMAIN PROTEIN 12); DNA binding / transcription activator/ transcription factor 1.714841218 0.879246856 0.130793895 -0.432607586 -0.672490268 0.955341992 -0.529985304 0.351416607

At2g45440 DHDPS2 (DIHYDRODIPICOLINATE SYNTHASE); catalytic/ dihydrodipicolinate synthase/ lyase 0.145775429 0.311348397 0.229988565 1.215406963 -0.117012249 0.878388481 -1.12659825 -0.715376286

At2g45220 pectinesterase family protein -0.367260077 0.781845533 -3.761787624 -2.293325663 -0.755159549 -0.102688783 -1.540917052 0.053546668

At2g47550 pectinesterase family protein 0.314530734 2.23462657 0.209870409 1.237597084 -1.11787805 -2.121421898 0.899752733 0.854466518

At2g33100 "CSLD1__ATCSLD1; cellulose synthase/ transferase, transferring glycosyl groups" -0.353583285 0.768379512 0.521968219 0.807661695 0.250990949 -0.616609763 3.782421907 -0.407184253

At2g33070 NSP2 (NITRILE SPECIFIER PROTEIN 2) -0.231019574 1.106603042 -0.142493064 0.185528114 0.581273217 -0.902809225 -0.987934216 -3.051234129

At2g33150 KAT2_PED1__PKT3 (PEROXISOMAL 3-KETOACYL-COA THIOLASE 3); acetyl-CoA C-acyltransferase 0.243058099 0.152445045 -1.899766996 -0.225154195 1.623712407 0.22356001 -0.047610268 -0.108391178

At2g33220 "FUNCTIONS IN: molecular_function unknown; INVOLVED IN: photorespiration; LOCATED IN: mitochondrion, mitochondrial membrane, plastid, respiratory chain complex I; EXPRESSED IN: 25 plant structures; EXPRESSED DURING: 15 growth stages; CONTAINS InterPro DOMA" -0.139243492 0.261428429 0.108484894 -0.273488746 0.471742298 0.157676294 0.092790792 0.133072981

At1g67740 YCF32__PSBY 0.135669998 -3.840538208 0.066143108 -0.614072121 -0.395675275 -0.323469794 -0.851748046 -0.086405462

At1g67730 YBR159; ketoreductase/ oxidoreductase 1.105584953 0.201941176 -0.177007808 -1.279031783 -0.482975201 1.241618938 -0.719173558 -0.39078433

At1g44575 PSBS__NPQ4 (NONPHOTOCHEMICAL QUENCHING); chlorophyll binding / xanthophyll binding 0.282091292 -3.107240249 0.172435109 -0.024231774 -0.936197215 0.501825312 -0.115046559 0.158658627

At4g25570 ACYB-2; carbon-monoxide oxygenase -0.573505082 -0.19041659 0.211911005 -2.080593497 0.118568678 -0.381665978 -0.550476649 1.251090999

At1g44446 ATCAO_CAO__CH1 (CHLORINA 1); chlorophyllide a oxygenase -0.381880642 -3.035552174 0.589474764 -1.96017983 -1.461736707 -1.417314384 -0.528321022 -1.396691438

At1g44350 ILL6; IAA-amino acid conjugate hydrolase/ metallopeptidase 1.070826079 -0.448696452 -0.047111009 1.325814333 -0.800667784 -3.489482037 0.682737965 -0.503723075

At4g16760 ATACX1__ACX1 (ACYL-COA OXIDASE 1); acyl-CoA oxidase 0.878159918 0.363359411 -1.656573912 1.238560456 -0.526572668 -0.738617131 -0.146222012 0.076155674

At4g15440 CYP74B2__HPL1 (HYDROPEROXIDE LYASE 1); electron carrier/ heme binding / iron ion binding / monooxygenase 1.848091341 -0.979173614 -0.71222554 4.02938965 0.208104092 2.319822624 0.916631144 1.794476852

At4g14680 APS3; sulfate adenylyltransferase (ATP) -1.036978441 -0.347297741 0.868476735 1.925192674 -1.077642081 -1.965980706 -0.439488615 -0.070137706

At4g15340 04C11__ATPEN1 (ARABIDOPSIS THALIANA PENTACYCLIC TRITERPENE SYNTHASE 1); arabidiol synthase -0.619479093 1.722495612 0.536224562 -1.201095856 0.186691881 -0.411108547 -0.161678896 0.656557227

At4g15210 AT-BETA-AMY_ATBETA-AMY_BMY1_RAM1__BAM5 (BETA-AMYLASE 5); beta-amylase 3.451585927 -1.830397169 -0.959724182 2.675868876 0.896404284 2.035845715 1.363291791 0.796997155

At4g15550 "IAGLU (INDOLE-3-ACETATE BETA-D-GLUCOSYLTRANSFERASE); UDP-glycosyltransferase/ transferase, transferring glycosyl groups" -0.724572033 -0.331864324 -2.103923284 -1.037484064 -1.604608401 0.283023552 0.389071477 0.743565311

At4g15560 DEF_DXPS2_DXS__CLA1 (CLOROPLASTOS ALTERADOS 1); 1-deoxy-D-xylulose-5-phosphate synthase 0.629360854 -1.671810063 0.313000921 -1.937385225 -0.232047441 -0.11221727 -0.869355655 0.847957318

At4g14210 PDE226__PDS3 (PHYTOENE DESATURASE 3); phytoene dehydrogenase -0.549764928 -1.471693528 -0.178746543 -0.439029969 -0.820235383 0.372192214 -0.05101246 -0.413338714

At4g14880 ATCYS-3A_CYTACS1__OASA1 (O-ACETYLSERINE (THIOL) LYASE (OAS-TL) ISOFORM A1); cysteine synthase -0.566108002 0.03831694 -0.096221218 -0.581352035 0.433116923 -0.245910562 -0.754094414 0.159126244

At4g14910 "imidazoleglycerol-phosphate dehydratase, putative" -0.468133412 -0.725761532 0.162221684 0.123098441 0.107169893 0.375526036 -0.412363428 -0.499755718

At4g17190 FPS2 (FARNESYL DIPHOSPHATE SYNTHASE 2); dimethylallyltranstransferase/ geranyltranstransferase 1.296647331 0.246956371 1.62391824 -0.895183005 0.569816893 0.848201053 -0.391967104 1.133299318

At4g16770 "iron ion binding / oxidoreductase/ oxidoreductase, acting on paired donors, with incorporation or reduction of molecular oxygen, 2-oxoglutarate as one donor, and incorporation of one atom each of oxygen into both donors" -0.379327125 0.144753896 -0.039685686 -0.213126518 1.105982799 0.031140098 -0.613410211 -0.104340456

At4g17260 "L-lactate dehydrogenase, putative" 1.454638903 0.778357798 0.832279125 1.718628781 0.687658063 -0.184963717 -0.874287263 0.428094217

At4g17090 BAM3_BMY8__CT-BMY (CHLOROPLAST BETA-AMYLASE); beta-amylase -1.506598655 -2.449450433 0.127743207 3.211993907 -0.680138867 0.961198081 0.221025439 0.493539273

At4g17770 "TPS5__ATTPS5; protein binding / transferase, transferring glycosyl groups / trehalose-phosphatase" -0.469733019 0.247692981 -0.11390197 -0.599479405 0.946303258 3.238316919 -0.908012723 0.520124681

At4g16690 "ATMES16__MES16 (METHYL ESTERASE 16); catalytic/ hydrolase, acting on ester bonds / methyl indole-3-acetate esterase/ methyl jasmonate esterase" 0.82896076 -1.617114592 -2.052322852 0.320740839 -1.408085599 0.759927847 0.238907065 -1.544819625

At4g15490 "UGT84A3; UDP-glycosyltransferase/ sinapate 1-glucosyltransferase/ transferase, transferring glycosyl groups" -0.053754686 -0.922456087 -2.347721131 1.296138166 1.542603125 1.247565787 0.613936141 -0.643427724

At4g13940 EMB1395_HOG1_SAHH1__MEE58 (MATERNAL EFFECT EMBRYO ARREST 58); adenosylhomocysteinase 0.712687045 0.430323329 0.238136563 -0.287441775 -0.465604458 0.261077765 -0.793084495 0.942330377

At4g14430 ATECI2_ECHIB_ECI2_PEC12__IBR10 (INDOLE-3-BUTYRIC ACID RESPONSE 10); catalytic/ dodecenoyl-CoA delta-isomerase 0.055192281 0.438864054 -0.666995603 0.080327649 0.384857779 -0.079813487 -0.138766675 -0.649607073

At4g16330 "oxidoreductase/ oxidoreductase, acting on paired donors, with incorporation or reduction of molecular oxygen, 2-oxoglutarate as one donor, and incorporation of one atom each of oxygen into both donors" -0.299678054 -0.135634663 -0.920304522 -1.234197749 0.912481031 0.211019636 -0.276272713 -0.281000971

At4g17480 palmitoyl protein thioesterase family protein 1.434903123 -0.050956029 0.482688564 -0.117225073 -0.48287776 0.404594565 0.244019107 -0.616577494

At4g16450 unknown protein 0.125281683 0.461318699 0.333680144 -0.496310044 0.333465371 0.622736419 -0.21492528 0.288296938

At4g17300 ATNS1_OVA8__NS1; asparagine-tRNA ligase -0.344289458 -0.478046183 0.780529807 0.786872589 0.070336042 -0.202147176 -0.083607451 -0.265513672

At4g17360 "formyltetrahydrofolate deformylase/ hydroxymethyl-, formyl- and related transferase/ methyltransferase" -2.236918872 -0.31461577 1.014819446 0.795832876 0.73955322 0.22155488 0.64038402 -0.223702901

At4g17470 palmitoyl protein thioesterase family protein -0.6833725 0.006452353 0.44377087 2.554536204 1.587103646 -0.276063817 -0.143129639 -0.432221103

At4g17483 palmitoyl protein thioesterase family protein 0.486408631 0.735391487 -0.025259561 -0.107872031 -0.084835198 -0.243575981 1.760959795 -0.792043767

At4g16700 PSD1 (phosphatidylserine decarboxylase 1); phosphatidylserine decarboxylase 0.515184297 0.352167881 0.829909409 0.949703332 0.2009949 -0.079722371 -0.328772031 -0.344118515

At4g16730 lyase/ magnesium ion binding 0.204611343 0.000598522 -0.563540411 0.169360519 -0.067510501 -0.287371024 0.486797167 -0.274000285

At4g16740 ATTPS03; (E)-beta-ocimene synthase/ myrcene synthase 0.231842571 -0.255945859 -1.134085577 0.372230298 -0.045518975 0.020366762 0.748096743 0.091615983

At4g16800 "enoyl-CoA hydratase, putative" 0.706628045 0.062418904 0.695317311 -0.365839379 0.84821368 0.743667748 -0.717485312 -0.779235033

At4g16820 lipase class 3 family protein 0.540867136 0.232909905 0.00468654 1.769151296 -0.100404338 -1.499472882 0.088629796 -0.386829421

At4g15980 pectinesterase family protein -0.048511192 0.803645637 0.651853399 0.616866752 0.349184668 -0.54102488 3.771943272 -0.265771701

At4g16130 ATISA1_ISA1__ARA1 (ARABINOSE KINASE); ATP binding / L-arabinokinase/ galactokinase 0.546401009 -0.540524374 -1.339214947 -1.392911271 0.545855295 0.364627349 1.261906388 -0.204352195

At4g16210 E-COAH-2__ECHIA (ENOYL-COA HYDRATASE/ISOMERASE A); catalytic -0.064702003 0.47989806 -0.968608526 0.011895714 0.786576867 0.210256348 -0.149466684 -0.855047459

At4g16310 LDL3 (LSD1-LIKE3); amine oxidase/ electron carrier/ oxidoreductase 0.601201868 0.118857608 0.080229185 0.27495546 0.234041321 -0.014428526 -0.208578923 -0.062550483

At4g15130 catalytic/ choline-phosphate cytidylyltransferase/ nucleotidyltransferase 0.336755376 0.543234789 0.526862018 2.317256794 0.046032843 -0.90064666 0.760103269 -0.350348436

At4g15260 UDP-glucoronosyl/UDP-glucosyl transferase family protein 0.072232362 -0.276889392 -1.386785063 0.494927246 0.188404835 -1.425109875 0.231041372 0.448915222

At4g15270 glucosyltransferase-related -0.213344227 0.496151076 0.126898739 0.389672122 0.250017305 -0.511556042 -0.138767819 -0.187232038

At4g15280 "UGT71B5 (UDP-GLUCOSYL TRANSFERASE 71B5); UDP-glycosyltransferase/ quercetin 3-O-glucosyltransferase/ transferase, transferring glycosyl groups" 0.13057187 0.353488855 0.047392907 0.723933326 0.189768779 -0.631900104 0.100769768 -0.405926994

At4g15290 "ATCSLB5_CSLB05__ATCSLB05; cellulose synthase/ transferase/ transferase, transferring glycosyl groups" -0.689565109 1.202662513 0.350218129 -0.812772283 -0.04149483 -0.033575739 0.143601383 0.17859723

At4g15320 "ATCSLB6_CSLB06__ATCSLB06 (CELLULOSE SYNTHASE LIKE B6); cellulose synthase/ transferase/ transferase, transferring glycosyl groups" 0.009021035 0.21993203 0.114309157 0.254506856 0.18379571 -0.220757905 0.238811802 -0.309182648

At4g15370 PEN2__BARS1 (BARUOL SYNTHASE 1); baruol synthase/ catalytic -0.469903706 0.509462011 0.629410889 0.179733766 0.181331089 0.047645092 0.237630899 -0.321014274

At4g15480 "UGT84A1; UDP-glycosyltransferase/ sinapate 1-glucosyltransferase/ transferase, transferring glycosyl groups" 2.077599742 0.929532385 0.352002403 0.747546158 -1.288680849 1.366735087 -0.851234968 0.36583424

At4g15500 "UGT84A4; UDP-glycosyltransferase/ sinapate 1-glucosyltransferase/ transferase, transferring glycosyl groups / transferase, transferring hexosyl groups" -1.116018608 1.016214564 0.40762972 -0.131258205 0.285749015 0.066929317 0.059666122 0.151826694

At4g14940 ATAO1 (ARABIDOPSIS THALIANA AMINE OXIDASE 1); amine oxidase/ copper ion binding -0.683508525 0.914079597 0.609270193 -0.985604984 -0.05145912 -0.547911315 -0.229849226 0.622284993

At4g14440 ATECI3_ECI3__HCD1 (3-HYDROXYACYL-COA DEHYDRATASE 1); carnitine racemase/ catalytic/ dodecenoyl-CoA delta-isomerase 0.374861297 -0.505881508 0.850971038 -1.00520997 -1.024490119 1.228623741 -0.757945525 -0.805603338

At4g14070 AAE15 (acyl-activating enzyme 15); long-chain-fatty-acid-[acyl-carrier-protein] ligase -0.729118369 -1.087632674 -0.52433763 0.224606138 1.202884282 0.33894726 0.658249483 -0.01272106

At4g14090 UDP-glucoronosyl/UDP-glucosyl transferase family protein -1.082065654 0.274041276 -0.530117021 3.044495543 -0.695714885 1.807007828 -0.955127212 -0.226307044

At1g56600 "AtGolS2 (Arabidopsis thaliana galactinol synthase 2); transferase, transferring glycosyl groups / transferase, transferring hexosyl groups" -0.632878496 -0.280743338 -2.615236597 2.017543045 0.2619793 -0.334449022 -0.995582848 -3.082324434

At1g56650 ATMYB75_MYB75_SIAA1__PAP1 (PRODUCTION OF ANTHOCYANIN PIGMENT 1); DNA binding / transcription factor -0.551527422 0.062145369 -1.725947148 1.830313589 -0.469234697 1.593926168 0.083267762 -0.264474899

At1g25350 OVA9 (ovule abortion 9); glutamine-tRNA ligase 0.610454146 0.280375839 0.245929586 0.039546197 -0.42079017 0.462079877 -0.938220935 -0.379831678

At1g24735 O-methyltransferase -0.707359383 0.605256436 -0.162459023 0.118525487 0.118194927 0.099080295 -0.397330015 -3.380078844

At1g56710 glycoside hydrolase family 28 protein / polygalacturonase (pectinase) family protein -0.30124851 0.12955609 0.114893282 0.357674694 0.755579726 0.517319607 0.42989721 -0.650458959

At1g56560 "beta-fructofuranosidase, putative / invertase, putative / saccharase, putative / beta-fructosidase, putative" -0.112291439 0.129914786 -0.538439742 0.237517134 0.043700046 0.453856167 0.415765155 -0.227525753

At5g04230 PAL3 (PHENYL ALANINE AMMONIA-LYASE 3); phenylalanine ammonia-lyase -0.791392741 -0.688193483 1.303755462 0.756910771 0.298479977 -0.664633438 -0.326168452 -0.555835637

At5g04180 ATACA3__ACA3 (ALPHA CARBONIC ANHYDRASE 3); carbonate dehydratase/ zinc ion binding -0.218284375 0.354273198 -0.207473062 -0.053840723 0.383675677 -0.280357485 5.435042927 0.100318271

At5g04140 GLS1_GLUS__GLU1 (GLUTAMATE SYNTHASE 1); glutamate synthase (ferredoxin) -1.483164803 -3.344852192 0.046636621 0.661221912 -0.576792033 0.126904641 -0.120178865 0.853013159

At5g04360 ATPU1_PU1__ATLDA (LIMIT DEXTRINASE); alpha-amylase/ limit dextrinase/ pullulanase -0.970322872 -0.888554059 0.209559875 0.889812988 2.762218752 0.389980786 0.479735313 -1.047237708

At1g73370 ATSUS6__SUS6 (SUCROSE SYNTHASE 6); UDP-glycosyltransferase/ sucrose synthase -0.219831213 0.551185871 0.59486616 -0.549958525 -0.079360086 -0.292241165 0.080922277 -0.31618061

At1g73480 "hydrolase, alpha/beta fold family protein" 0.097206336 -0.681570178 -0.487206343 0.772875549 -0.635094733 -3.484578202 1.432616523 -1.176306941

At1g44170 ALDH4__ALDH3H1 (ALDEHYDE DEHYDROGENASE 3H1); 3-chloroallyl aldehyde dehydrogenase/ aldehyde dehydrogenase (NAD) 0.766219589 0.526851245 -1.049783309 -0.991046778 0.354237398 0.985780396 0.012184882 0.342460701

At1g35190 "oxidoreductase, 2OG-Fe(II) oxygenase family protein" -1.616572769 0.832464477 -2.250804072 -1.055238579 0.05860332 -0.230798478 1.39130128 -1.678706761

At1g32180 "CSLD6__ATCSLD6; cellulose synthase/ transferase, transferring glycosyl groups" 0.203604837 0.258531106 0.205807449 0.203895592 0.144630736 -0.198839968 0.788233435 -0.174379407

At1g32200 ACT1__ATS1; glycerol-3-phosphate O-acyltransferase 0.143173609 -1.421344502 0.927807388 -0.122152497 -0.144072948 -0.590886439 -0.572047636 -0.57175387

At1g58440 SQE1__XF1; squalene monooxygenase 0.069147136 0.220868815 0.86625094 -0.799514331 -0.332194133 -0.554180445 -0.183945981 -0.249430017

At1g26100 cytochrome B561 family protein 0.410819716 0.409460883 1.250189054 -0.25893859 0.550789904 0.647364193 -0.42266437 0.12647211

At1g48850 EMB1144 (embryo defective 1144); chorismate synthase 0.53699615 0.67603257 -0.084686064 0.659259147 -0.923940576 0.341733549 -0.299284869 0.646546278

At1g58080 HISN1A__ATATP-PRT1 (ATP PHOSPHORIBOSYL TRANSFERASE 1); ATP phosphoribosyltransferase -1.147940264 -0.036645246 0.917289096 0.304898967 -0.563483445 -0.056147427 -0.652709753 0.310795144

At1g26190 phosphoribulokinase/uridine kinase family protein 1.347024531 0.524910665 0.945865083 1.003427409 0.644429263 0.402565982 -0.29832296 -0.409732954

At5g09420 "MTOM64__ATTOC64-V (ARABIDOPSIS THALIANA TRANSLOCON AT THE OUTER MEMBRANE OF CHLOROPLASTS 64-V); amidase/ binding / carbon-nitrogen ligase, with glutamine as amido-N-donor" 1.055325372 0.798813014 0.367164889 1.571366247 -0.294872499 -0.299452721 -0.94171802 -0.137389054

At5g09430 hydrolase -0.124907265 0.863722573 0.59727862 0.800826664 0.361318073 -0.754264207 2.775643686 -0.040403816

At5g09300 "2-oxoisovalerate dehydrogenase, putative / 3-methyl-2-oxobutanoate dehydrogenase, putative / branched-chain alpha-keto acid dehydrogenase E1 alpha subunit, putative" 1.824887054 0.387863636 0.507576985 -0.069617071 0.393936794 1.129955844 -0.110472086 -1.034985857

At5g11110 KNS2_SPS1__ATSPS2F (SUCROSE PHOSPHATE SYNTHASE 2F); sucrose-phosphate synthase -0.321434668 0.997864086 -2.082239713 1.211107466 0.025800402 0.564603306 1.226587308 0.720151111

At5g24760 "alcohol dehydrogenase, putative" 1.261841769 0.815283031 0.365240722 -0.073158474 -1.026054902 0.09307638 -1.155249337 0.512929818

At5g19850 "hydrolase, alpha/beta fold family protein" -1.187165957 -1.008380079 -0.775812901 0.627367514 -1.523192493 0.59035709 -0.224689682 -0.829070198

At5g19530 ACL5 (ACAULIS 5); spermine synthase/ thermospermine synthase 0.296380022 0.170875332 1.323413392 -3.052631074 0.469381925 -0.325941479 -1.342229884 -0.145419242

At5g19550 AAT2__ASP2 (ASPARTATE AMINOTRANSFERASE 2); L-aspartate:2-oxoglutarate aminotransferase -0.066759543 0.772804318 -1.087401935 0.827767362 0.120576665 0.458866994 -1.122033365 -0.349743514

At5g19730 pectinesterase family protein 0.951317765 -0.081028586 0.996774036 -0.520705578 -1.669150894 1.370718371 0.64050561 -0.357830602

At5g13110 G6PD2 (GLUCOSE-6-PHOSPHATE DEHYDROGENASE 2); glucose-6-phosphate dehydrogenase -0.497427734 0.950463177 -0.61121234 0.479999294 -0.772658461 0.538718827 -0.536480806 -0.193902066

At5g13050 5-FCL (5-FORMYLTETRAHYDROFOLATE CYCLOLIGASE); 5-formyltetrahydrofolate cyclo-ligase 0.110264002 0.176435132 -0.200379814 -0.107367043 0.696346674 0.495845096 -0.232890073 0.351795397

At5g20830 ASUS1_atsus1__SUS1 (SUCROSE SYNTHASE 1); UDP-glycosyltransferase/ sucrose synthase 0.78233388 0.262788798 -2.861036918 1.130858826 0.516078115 1.019444347 0.840467345 0.541574498

At5g08415 lipoic acid synthase family protein 0.24517826 -0.144558312 0.800130817 -0.770149166 0.154091771 0.617157422 0.20939641 -0.358658247

At5g21100 "L-ascorbate oxidase, putative" 0.178278099 -1.483565298 0.108417781 -0.031881678 -0.339821119 1.017256314 0.758231092 1.003819968

At5g21060 homoserine dehydrogenase family protein 0.145235477 -0.224325099 0.771007531 0.242923687 -0.348079255 0.625563745 -0.286700793 0.658140946

At5g21105 L-ascorbate oxidase/ copper ion binding / oxidoreductase 0.622370572 0.083351208 -0.134768781 -0.497659501 -0.820587746 -0.664919724 -0.364608099 1.324372126

At5g08280 HEMC (HYDROXYMETHYLBILANE SYNTHASE); hydroxymethylbilane synthase -0.14187026 -1.367908291 1.169548477 0.217742568 1.182771766 -0.24421667 -0.68479315 -0.011078654

At5g08300 "succinyl-CoA ligase (GDP-forming) alpha-chain, mitochondrial, putative / succinyl-CoA synthetase, alpha chain, putative / SCS-alpha, putative" 0.300389537 0.715442112 -0.351252218 0.934602801 0.032140807 0.603102262 -0.678641859 1.26025523

At5g08370 "AtAGAL2 (Arabidopsis thaliana ALPHA-GALACTOSIDASE 2); alpha-galactosidase/ catalytic/ hydrolase, hydrolyzing O-glycosyl compounds" -0.872261505 -0.630790983 0.354426078 -0.34141772 0.817137967 0.957146716 0.437420826 2.119723107

At5g19470 atnudt24 (Arabidopsis thaliana Nudix hydrolase homolog 24); hydrolase -0.207846998 -0.151229708 -0.182400129 1.248940496 0.470380673 0.06342799 0.185058517 0.301566804

At5g19440 "cinnamyl-alcohol dehydrogenase, putative (CAD)" 1.224174761 0.819815935 -1.342835697 1.125949894 0.726537147 0.711323491 -2.587164737 0.212550265

At5g28840 "GME (GDP-D-MANNOSE 3',5'-EPIMERASE); GDP-mannose 3,5-epimerase/ NAD or NADH binding / catalytic" 0.327846825 -0.819303305 0.117562325 -0.143186329 -0.441127562 0.492060672 -0.556717272 0.034052955

At5g08380 "AtAGAL1 (Arabidopsis thaliana ALPHA-GALACTOSIDASE 1); alpha-galactosidase/ catalytic/ hydrolase, hydrolyzing O-glycosyl compounds" 0.681691504 -0.699614109 -1.388176795 0.737558137 3.129185329 2.326410524 -1.577621166 0.843202774

At5g20410 "ATMGD2__MGD2; 1,2-diacylglycerol 3-beta-galactosyltransferase/ UDP-galactosyltransferase/ transferase, transferring glycosyl groups" 0.015952269 0.3228367 0.068438835 0.214263841 0.002638195 -0.044288843 3.792537549 0.337099222

At5g20280 "ATSPS1F (sucrose phosphate synthase 1F); sucrose-phosphate synthase/ transferase, transferring glycosyl groups" -0.286307633 -0.937248643 -1.783671237 -0.220266275 1.084126362 1.261036281 0.495181646 0.446030906

At5g20550 "oxidoreductase, 2OG-Fe(II) oxygenase family protein" -0.778798799 1.912105945 0.438819633 -1.02095217 -0.147101499 0.079861424 -0.398834118 0.742269233

At5g20400 "oxidoreductase, 2OG-Fe(II) oxygenase family protein" -1.152330856 1.570233876 -0.645666118 -0.263868008 -0.056320429 -0.725262753 -1.474153404 1.395080013

At5g20250 "DIN10 (DARK INDUCIBLE 10); hydrolase, hydrolyzing O-glycosyl compounds" 0.404853614 -0.682395561 0.572057156 -3.761686494 1.189464703 -4.349161901 -0.612285013 0.724455971

At5g20990 CHL6_CNX_CNX1_SIR4__B73; molybdenum ion binding -0.376549319 0.327239664 -0.000728873 0.304267676 0.473565053 -0.232797615 -0.732606443 0.225304245

At5g20960 AO1_ATAO_ATAO1__AAO1 (ARABIDOPSIS ALDEHYDE OXIDASE 1); aldehyde oxidase/ indole-3-acetaldehyde oxidase -0.969645386 2.042729955 -1.824559353 -0.398378527 0.019130219 -0.520016703 -2.226439615 -0.543751568

At5g20040 ATIPT9; ATP binding / tRNA isopentenyltransferase -0.142934063 0.01077908 1.235401174 0.436106731 0.377707434 0.090186847 -0.414802532 -0.355670233

At5g28360 "ACS3 (1-AMINOCYCLOPROPANE-1-CARBOXYLATE SYNTHASE LIKE PSEUDOGENE); 1-aminocyclopropane-1-carboxylate synthase/ catalytic/ pyridoxal phosphate binding / transferase, transferring nitrogenous groups" -0.117501745 0.292774337 0.526977023 0.612301605 0.119340323 -0.248029075 0.329086601 -0.637780286

At5g20860 pectinesterase family protein -0.1614584 0.591760802 0.210695197 -0.279404373 -0.120404811 -0.019323125 -0.581730483 -2.513354654

At5g20980 ATMS3 (methionine synthase 3); 5-methyltetrahydropteroyltriglutamate-homocysteine S-methyltransferase/ methionine synthase -0.498533223 0.51244907 1.206613794 0.464795789 0.023913449 0.211946129 0.467119946 -0.043161943

At4g37000 ATRCCR__ACD2 (ACCELERATED CELL DEATH 2); red chlorophyll catabolite reductase -0.585077972 -1.280600069 -0.29481809 -0.311258471 0.352435493 0.625987657 -0.226234166 -0.834546719

At4g36810 GGPS1 (GERANYLGERANYL PYROPHOSPHATE SYNTHASE 1); farnesyltranstransferase -0.288085923 -1.019640519 0.189391696 -0.104136913 -0.050070208 0.925398394 -0.487561871 0.371054988

At4g36530 "hydrolase, alpha/beta fold family protein" -1.095063306 -1.437240711 -0.267326477 -0.289288176 -0.078712149 0.416111201 0.031522256 -1.141012608

At4g36480 EMB2779_FBR11_LCB1__ATLCB1 (LONG-CHAIN BASE1); protein binding / serine C-palmitoyltransferase 0.613221779 0.337820757 0.379068406 0.19699174 -0.14328112 -0.68562585 -0.39198954 -0.148897312

At4g36380 "ROT3 (ROTUNDIFOLIA 3); oxidoreductase, acting on paired donors, with incorporation or reduction of molecular oxygen, NADH or NADPH as one donor, and incorporation of one atom of oxygen / oxygen binding / steroid hydroxylase" 0.249288119 0.704579724 -0.778153593 -2.473268341 -0.620013749 -0.061642233 0.445524985 -0.99679778

At4g36890 "IRX14 (irregular xylem 14); transferase, transferring glycosyl groups / xylosyltransferase" 0.037482339 0.151464369 -0.1400632 -1.043337699 -0.129004343 0.434292943 -0.222171697 0.377778536

At4g37070 "PLA IVA_PLP1__patatin, putative" -0.671311064 2.181081199 -0.058207943 -2.133261672 -0.372001558 -0.329117584 -0.754819939 1.203852176

At4g36770 "UDP-glycosyltransferase/ transferase, transferring glycosyl groups" 0.268036249 -0.144477675 0.258314969 0.039176355 -0.086713122 -0.156217112 1.144127217 0.073706841

At1g31860 HISN2__AT-IE; phosphoribosyl-AMP cyclohydrolase/ phosphoribosyl-ATP diphosphatase 0.615749102 0.296285875 1.777916785 0.330715957 -0.563596008 0.423553891 -1.084370816 -0.087209184

At1g31800 LUT5__CYP97A3 (CYTOCHROME P450-TYPE MONOOXYGENASE 97A3); carotene beta-ring hydroxylase/ oxygen binding -0.57581093 -1.683708085 0.428792675 0.344112105 -0.172480375 0.734228564 0.054502844 0.994346657

At4g37150 "ATMES9__MES9 (METHYL ESTERASE 9); hydrolase, acting on ester bonds / methyl indole-3-acetate esterase/ methyl jasmonate esterase/ methyl salicylate esterase" -0.869649353 0.423517893 -2.21545724 -1.669244664 -1.78370558 0.435744781 -0.103079953 0.779574962

At4g36940 NAPRT1 (NICOTINATE PHOSPHORIBOSYLTRANSFERASE 1); nicotinate phosphoribosyltransferase -0.166363045 0.286120832 0.21391657 0.077415319 1.028951319 1.086859626 0.12141877 0.950264624

At3g51840 ATG6_ATSCX__ACX4 (ACYL-COA OXIDASE 4); acyl-CoA oxidase/ oxidoreductase 0.418625938 -0.266183694 -1.071774334 -2.10605663 0.730541475 -1.14880726 0.197364154 0.452117723

At3g51820 ATG4_CHLG__G4; chlorophyll synthetase -0.679970257 -1.756907745 0.911405551 0.401842418 -0.445527549 0.626097403 0.029452222 0.286515426

At1g16540 ATABA3_LOS5_SIR3__ABA3 (ABA DEFICIENT 3); Mo-molybdopterin cofactor sulfurase/ selenocysteine lyase 0.67066847 0.398937393 -1.743201806 0.402529041 0.356345135 0.119030978 -0.190788351 -0.129281169

At3g43600 AAO2 (ALDEHYDE OXIDASE 2); aldehyde oxidase -2.064452984 1.103359174 1.116530094 -0.381295637 -0.602629773 0.32872547 -1.082408224 -0.030623767

At3g44830 lecithin:cholesterol acyltransferase family protein / LACT family protein -0.003767689 0.203906239 0.099451222 0.402090281 0.164242339 -0.575460483 -0.345365553 -2.411646369

At3g44880 LLS1_PAO__ACD1 (ACCELERATED CELL DEATH 1); iron-sulfur cluster binding / pheophorbide a oxygenase 0.180149137 -2.000798738 -2.717876954 0.86938799 0.845043682 0.131346037 -0.226434034 -1.462845551

At3g56700 "FAR6 (FATTY ACID REDUCTASE 6); fatty acyl-CoA reductase (alcohol-forming)/ oxidoreductase, acting on the CH-CH group of donors, NAD or NADP as acceptor" -0.095160449 0.268077675 0.252849171 0.128083814 0.305451599 -0.387616621 0.236010102 -0.356783768

At1g16570 glycosyl transferase family 1 protein 0.386327891 0.357808044 0.239284707 -0.38386207 0.678480386 0.04874063 0.055663305 0.265179137

At1g57750 4.529893989 -1.774088096 -0.450723627 -1.961295614 -0.268432444 0.598732014 0.293834917 1.566296169

At1g58180 ATBCA6_BCA6__carbonic anhydrase family protein / carbonate dehydratase family protein -0.795365367 -1.191093398 -2.486489427 -2.368223554 0.819980093 -1.856613035 -0.363311817 0.850770752

At5g17530 phosphoglucosamine mutase family protein -0.382345658 -0.023361704 0.08232801 -0.127217908 0.103148998 0.634291207 -0.341076571 0.235531023

At5g17030 "UGT78D3 (UDP-GLUCOSYL TRANSFERASE 78D3); UDP-glycosyltransferase/ flavonol 3-O-arabinosyltransferase/ quercetin 3-O-glucosyltransferase/ transferase, transferring glycosyl groups" 1.371040637 0.150747713 0.307378809 0.673692947 0.165603261 0.096228043 0.208038901 0.064789878

At5g17420 ATCESA7_CESA7_MUR10__IRX3 (IRREGULAR XYLEM 3); cellulose synthase -0.901560436 0.361479545 0.049746919 -2.022434669 -0.298335227 0.028980197 0.992968974 0.54994784

At5g16760 "inositol 1,3,4-trisphosphate 5/6-kinase" 0.485413806 0.616262806 -0.079228245 0.46517027 -0.301325187 0.138006416 -0.00666742 -0.845686371

At5g16710 DHAR3 (dehydroascorbate reductase 1); glutathione dehydrogenase (ascorbate) -0.849825113 -1.400762943 0.371647342 -0.03718112 0.411561773 0.091507034 -0.451495751 -0.080017227

At5g16910 "ATCSLD2__CSLD2 (CELLULOSE-SYNTHASE LIKE D2); cellulose synthase/ transferase, transferring glycosyl groups" 0.567441804 0.578042823 -0.398583731 0.234960141 -0.710299527 -1.592330918 -0.951394836 -0.046722053

At5g17040 UDP-glucoronosyl/UDP-glucosyl transferase family protein -0.085726613 0.366906638 0.391369212 0.663879527 -0.260529349 0.106590724 0.087179656 -0.572927348

At5g17050 "UGT78D2 (UDP-GLUCOSYL TRANSFERASE 78D2); UDP-glycosyltransferase/ anthocyanidin 3-O-glucosyltransferase/ quercetin 3-O-glucosyltransferase/ transferase, transferring glycosyl groups" -0.328321758 0.135854733 -0.691641687 1.267499301 -2.99507449 2.3161275 -1.130745186 -0.704557001

At5g15950 adenosylmethionine decarboxylase family protein -1.022238527 -0.43824702 -0.112028193 2.122941785 -3.891464303 1.281813198 1.766288252 -1.678484173

At5g16230 "acyl-(acyl-carrier-protein) desaturase, putative / stearoyl-ACP desaturase, putative" -0.426516935 0.978967288 -0.040650749 0.208447747 0.27401289 0.412841752 -0.461869226 -2.383624348

At5g16240 "acyl-(acyl-carrier-protein) desaturase, putative / stearoyl-ACP desaturase, putative" 0.263768667 -0.460523153 0.623109953 -1.175199169 -0.221154118 0.28774279 1.480892849 -1.082839762

At5g16120 "hydrolase, alpha/beta fold family protein" 0.774095341 -0.342323284 -0.871942308 -1.09223971 0.539608092 -0.761222482 -0.209527371 0.077015766

At5g16715 EMB2247 (embryo defective 2247); ATP binding / aminoacyl-tRNA ligase/ nucleotide binding / valine-tRNA ligase -0.333940247 -0.682305474 1.339461066 0.962541353 -0.041833432 0.428987507 -0.1087309 -0.126759831

At5g15490 "UDP-glucose 6-dehydrogenase, putative" -0.306981934 1.094314419 0.216393028 0.082544925 -0.575463428 0.681191963 0.543164914 -0.259169277

At5g15770 AtGNA1 (Arabidopsis thaliana glucose-6-phosphate acetyltransferase 1); N-acetyltransferase/ glucosamine 6-phosphate N-acetyltransferase -0.367562884 0.469995963 0.634609369 -0.443295593 1.258483935 1.366056604 0.133280203 0.231788666

At5g15470 "GAUT14 (Galacturonosyltransferase 14); polygalacturonate 4-alpha-galacturonosyltransferase/ transferase, transferring glycosyl groups / transferase, transferring hexosyl groups" -0.629061529 0.845036666 0.348678039 0.477256013 -0.732010923 -0.003481068 1.644883195 0.233091919

At5g14950 ATGMII__GMII (GOLGI ALPHA-MANNOSIDASE II); alpha-mannosidase 0.015312366 0.491252188 -0.070959309 0.28029482 0.594424574 -0.027929168 -0.016596943 0.086056346

At5g14860 "transferase, transferring glycosyl groups" 0.033496049 0.292021359 0.098728835 0.396108046 0.042284265 -0.125508548 0.380008188 -0.611978616

At5g14800 AT-P5C1_AT-P5R_EMB2772__P5CR (PYRROLINE-5- CARBOXYLATE (P5C) REDUCTASE); pyrroline-5-carboxylate reductase 0.752418928 0.699700822 0.4391538 0.415386958 -0.018012833 0.373810514 -0.528745216 0.705804589

At5g14740 BETA CA2_CA18__CA2 (CARBONIC ANHYDRASE 2); carbonate dehydratase/ zinc ion binding -3.18880297 -3.88333259 0.876304028 1.133001997 -0.135776072 -1.731674499 -0.024719179 0.444194094

At5g14760 AO (L-ASPARTATE OXIDASE); L-aspartate oxidase/ electron carrier/ oxidoreductase -0.606924835 -0.076854564 0.24041136 2.429259256 -4.912617028 0.961688518 1.307276975 0.654967953

At5g14850 "mannosyltransferase, putative" 0.478072163 0.232484881 0.335103823 0.333318293 0.392550753 0.129594995 -0.112179413 -0.317308014

At5g35360 CAC2; acetyl-CoA carboxylase/ biotin carboxylase 0.610435285 -0.057036953 1.433010087 -1.045505557 -0.01838116 0.109894916 -0.317307476 -0.182484768

At1g48860 "3-phosphoshikimate 1-carboxyvinyltransferase, putative / 5-enolpyruvylshikimate-3-phosphate, putative / EPSP synthase, putative" 0.773196923 0.350187907 0.063349392 -0.040811578 -1.153600497 0.090772875 -1.202971938 0.597068035

At5g35170 adenylate kinase family protein -0.202053504 -2.185601559 0.362773811 -0.266856524 0.938826051 -0.311978806 0.268877567 0.570895043

At5g28030 "cysteine synthase, putative / O-acetylserine (thiol)-lyase, putative / O-acetylserine sulfhydrylase, putative" -0.259517118 -0.684708222 -0.481864747 0.365267719 -0.178263625 1.008407231 0.359045436 0.084581944

At5g28020 ATCYSD2__CYSD2 (CYSTEINE SYNTHASE D2); catalytic/ cysteine synthase/ pyridoxal phosphate binding -1.212991229 -0.481039059 -0.31885473 -1.726261814 -0.298008195 0.029036417 -0.793095079 1.208703276

At5g27870 pectinesterase family protein -0.354261096 0.76205018 0.373878912 0.460107914 0.339431396 -0.288560853 4.470643644 -0.16888701

At5g27530 glycoside hydrolase family 28 protein / polygalacturonase (pectinase) family protein -0.075261042 0.17342385 0.275800845 0.378494223 0.371730553 -0.401533452 0.242009058 -0.387177283

At5g27450 MVK__MK (MEVALONATE KINASE); mevalonate kinase 0.642895174 0.869386438 1.428351545 -0.636150086 0.308544244 -0.254747184 0.291514958 0.234058412

At5g27470 seryl-tRNA synthetase / serine--tRNA ligase 0.783120892 0.232078513 0.462811787 0.030122047 -0.280236026 0.232903325 -0.988119201 0.276776215

At5g27380 GSHB__GSH2 (GLUTATHIONE SYNTHETASE 2); glutathione synthase 0.583784441 0.059820376 -0.222528298 0.316508898 1.826311053 -0.825647289 -1.174193818 1.317932276

At5g27600 LACS7 (LONG-CHAIN ACYL-COA SYNTHETASE 7); long-chain-fatty-acid-CoA ligase/ protein binding 0.524501298 0.572383055 -2.378575508 0.611077834 0.487993629 0.471471671 -0.543519172 -0.132163026

At5g26780 SHM2 (SERINE HYDROXYMETHYLTRANSFERASE 2); catalytic/ glycine hydroxymethyltransferase/ pyridoxal phosphate binding -0.327914044 0.741152255 0.716578282 0.185836951 0.416003711 1.128161275 -0.348247314 -0.057250519

At5g26830 threonyl-tRNA synthetase / threonine--tRNA ligase (THRRS) 0.979282151 0.196961314 0.307625044 0.143011238 -0.085106787 0.658216066 -1.046859759 0.308903604

At5g26310 "UGT72E3; UDP-glycosyltransferase/ coniferyl-alcohol glucosyltransferase/ transferase, transferring glycosyl groups" -0.724119221 1.255083283 0.010396237 -1.29769359 0.005990822 -0.215566856 0.728016529 0.467918733

At5g25900 ATKO1_CYP701A3__GA3 (GA REQUIRING 3); ent-kaurene oxidase/ oxygen binding 1.137771101 -0.393934771 -0.828013529 -0.956161579 -0.111299453 0.133005539 -0.678879849 -1.361282271

At5g26030 FC1 (ferrochelatase 1); ferrochelatase 0.196953581 0.513004387 -0.395234028 1.561741898 -1.329968635 -1.658111788 -0.024214808 0.735611922

At5g25980 "BGLU37__TGG2 (GLUCOSIDE GLUCOHYDROLASE 2); hydrolase, hydrolyzing O-glycosyl compounds / thioglucosidase" 1.670177235 -3.83637956 0.057886095 1.303882681 0.855709825 -0.707994927 -0.018689969 0.101697174

At5g25370 PLDALPHA3 (PHOSPHLIPASE D ALPHA 3); phospholipase D -0.213615769 0.328807733 0.51889809 0.144616955 0.128927571 -0.500269003 0.289685666 -0.497702677

At5g67520 "adenylylsulfate kinase, putative" 0.004130213 0.518902089 -0.213790013 -0.137911457 -0.01904525 -0.493023841 1.719311526 -1.063803338

At5g67590 FRO1 (FROSTBITE1); NADH dehydrogenase (ubiquinone) -0.109927881 0.280154248 -0.024840558 -0.063154196 0.439373295 0.087253242 -0.060027984 0.423506856

At5g67050 lipase class 3 family protein 0.107378364 0.074496151 0.27918705 0.360810931 0.223245067 -0.419214317 0.234810986 -0.195279018

At5g67030 ATABA1_ATZEP_IBS3_LOS6_NPQ2_ZEP__ABA1 (ABA DEFICIENT 1); zeaxanthin epoxidase -0.518338795 -2.699077586 -1.287821368 -2.033985668 -2.035248156 -0.84766579 -0.220116718 -0.709979725

At5g66690 "UGT72E2; UDP-glycosyltransferase/ coniferyl-alcohol glucosyltransferase/ transferase, transferring glycosyl groups" -0.902123414 2.197292725 -0.029261271 -1.480746331 -0.270311839 0.037860665 -0.528766911 0.595677272

At5g66760 SDH1-1; ATP binding / succinate dehydrogenase 0.341570349 0.853747809 -1.44007534 0.513149651 -0.751277482 0.954010331 0.385569432 0.557865582

At5g66920 sks17 (SKU5 Similar 17); copper ion binding / oxidoreductase -1.198907634 0.852486589 2.264622989 -1.493469337 -0.983215167 -0.615286833 -0.731643492 0.858510818

At5g66570 MSP-1_OE33_OEE1_OEE33_PSBO-1__PSBO1 (PS II OXYGEN-EVOLVING COMPLEX 1); oxygen evolving/ poly(U) binding 0.256294722 -3.337058049 -0.147758934 -0.839287895 -0.134316346 -0.406484895 -0.593290774 0.160117543

At5g66510 GAMMA CA3 (GAMMA CARBONIC ANHYDRASE 3); carbonate dehydratase 0.124753914 0.510291151 0.557129927 -0.016353589 0.115615591 0.413614354 0.017628581 1.143583959

At5g66280 "GMD1 (GDP-D-MANNOSE 4,6-DEHYDRATASE 1); GDP-mannose 4,6-dehydratase/ binding / catalytic/ coenzyme binding" -0.883956175 2.036007227 0.934029212 -1.102170061 -0.036704941 0.205649952 0.405805537 0.64500352

At5g66040 STR16 (SULFURTRANSFERASE PROTEIN 16) -2.562932582 0.028298789 -0.536958988 -1.50788465 0.47724794 -0.395113831 -1.612409267 0.062893837

At5g65940 CHY1 (BETA-HYDROXYISOBUTYRYL-COA HYDROLASE 1); 3-hydroxyisobutyryl-CoA hydrolase 0.284473442 -0.242101658 -0.255424282 -0.513231958 0.703423459 0.287619126 0.038269589 0.56647571

At5g66220 chalcone isomerase -0.007648781 0.277517252 0.48756602 0.280570989 0.246897968 -0.566970771 0.38202426 -0.65043109

At5g66170 FUNCTIONS IN: molecular_function unknown; INVOLVED IN: aging; LOCATED IN: cellular_component unknown; EXPRESSED IN: 22 plant structures; EXPRESSED DURING: 11 growth stages; CONTAINS InterPro DOMAIN/s: Rhodanese-like (InterPro:IPR001763); BEST Arabidopsis 0.982001334 0.871339668 -2.131271213 -3.458433128 -0.87183846 0.076929701 -0.069375566 -0.596135502

At5g66120 "3-dehydroquinate synthase, putative" -0.114792333 -0.111528138 -0.99700323 -0.165715795 -0.420695615 0.544689771 -0.790459897 0.208201469

At5g65780 ATBCAT-5; branched-chain-amino-acid transaminase/ catalytic 0.21947507 0.170650424 0.063708913 -0.435191059 0.677341012 0.854368084 -0.709810203 -0.111042022

At5g65800 CIN5_ETO2__ACS5 (ACC SYNTHASE 5); 1-aminocyclopropane-1-carboxylate synthase 0.05500558 0.315299589 0.337336445 0.094630135 0.326504076 -0.508326414 0.075198929 -0.354342273

At5g65685 soluble glycogen synthase-related -0.205248407 -0.142003908 0.570235875 0.080140805 0.453344048 -0.167335427 0.446403795 -0.309532294

At5g65720 ATNFS1_ATNIFS1_NIFS1__NFS1; ATP binding / cysteine desulfurase/ transaminase 0.268086692 0.230513667 -0.37289477 0.201349778 -0.558626265 0.524166779 -0.637138198 0.249738208

At5g65550 UDP-glucoronosyl/UDP-glucosyl transferase family protein -0.126724535 0.470640705 0.093942282 0.565954635 0.627358024 -0.60287898 -0.257251944 -3.00714976

At5g65110 ATACX2__ACX2 (ACYL-COA OXIDASE 2); acyl-CoA oxidase 0.04800932 0.322558455 -2.131797066 -0.586722642 0.664955601 -0.789530018 0.219710215 0.050641964

At5g64860 DPE1 (DISPROPORTIONATING ENZYME); 4-alpha-glucanotransferase/ catalytic/ cation binding 0.165638709 -0.468265953 0.831320358 0.288030585 5.432677251 0.680590091 -0.035005217 0.716602081

At5g65010 ASN2 (ASPARAGINE SYNTHETASE 2); asparagine synthase (glutamine-hydrolyzing) -2.074831366 -0.487223297 2.228220245 0.595587146 -0.117244049 0.429040739 -0.605417723 0.601392909

At5g65140 "trehalose-6-phosphate phosphatase, putative" 1.295684931 0.417955505 -0.901719653 1.171524056 -1.71128598 -0.16606261 -0.008844963 -1.774579897

At5g64640 pectinesterase family protein 0.345723232 0.423578168 1.364377898 1.500878677 0.845460392 0.08908973 1.016963908 -1.061543885

At5g64740 "E112_IXR2_PRC1__CESA6 (CELLULOSE SYNTHASE 6); cellulose synthase/ transferase, transferring glycosyl groups" -0.185754214 -0.011311572 0.737256567 -0.677523986 -0.527055126 -0.502850607 -0.585942119 -0.019889165

At5g64210 AOX2; alternative oxidase 0.210807695 0.256207712 0.086976757 0.654566118 0.425186386 -0.670158568 0.007988784 -1.295373483

At5g64300 "ATGCH; 3,4-dihydroxy-2-butanone-4-phosphate synthase/ GTP cyclohydrolase II" -0.156216447 -0.111776652 -0.102211102 1.327040317 -0.318896178 -0.900566362 -0.538168804 0.650680313

At5g64370 BETA-UP (beta-ureidopropionase); beta-ureidopropionase -0.126423507 0.432756185 -2.202895031 -1.105004547 1.254328851 0.965132912 -0.258752238 0.250965486

At5g64380 "fructose-1,6-bisphosphatase family protein" -0.141858848 -1.350131314 0.962661547 -0.371019315 1.015542363 -0.500088394 -0.243826552 -0.829653301

At5g63890 ATHDH (HISTIDINOL DEHYDROGENASE); histidinol dehydrogenase -0.382873621 -0.082303172 0.710758629 0.473883793 0.2551775 0.291452809 -0.447256243 0.144682306

At5g64050 ATERS_OVA3__ERS (GLUTAMATE TRNA SYNTHETASE); glutamate-tRNA ligase -0.163872298 -0.466117237 0.57588172 0.445282128 0.482897855 -0.172255665 -0.422814815 -0.16995554

At5g64040 PSAN; calmodulin binding 0.147256146 -4.017593193 0.154371291 -1.650048582 -0.156942066 -0.545681192 -0.787475586 0.798743851

At5g63510 GAMMA CAL1 (GAMMA CARBONIC ANHYDRASE LIKE 1); carbonate dehydratase 0.323910396 0.417346406 0.639365307 -0.310946752 0.253594125 0.319178828 -0.143798841 0.35034571

At5g63600 FLS5 (FLAVONOL SYNTHASE 5); flavonol synthase -1.06127742 3.004807673 0.198494871 -2.267155514 -0.281965396 -0.780335678 -0.981927236 1.425562845

At5g63680 "pyruvate kinase, putative" 0.634002553 0.640157598 -0.910286081 0.174767022 -0.098432863 0.459127922 -0.661321433 1.572483111

At5g63590 FLS3 (FLAVONOL SYNTHASE 3); flavonol synthase -0.713027672 1.806431585 0.125046754 -1.175026602 -0.17306482 0.00606852 -0.673623456 -0.488471637

At5g63580 FLS2 (FLAVONOL SYNTHASE 2); flavonol synthase 0.777244227 -0.365082507 -0.658681069 -1.054874209 -0.494241059 0.366577323 0.446402923 0.521731018

At5g63560 transferase family protein -0.680626016 1.960021666 -0.198675832 -1.129598974 -0.285442085 -0.373550587 -1.176312175 -2.09292194

At5g63310 NDPK IA_NDPK IA IA_NDPK1A__NDPK2 (NUCLEOSIDE DIPHOSPHATE KINASE 2); ATP binding / nucleoside diphosphate kinase/ protein binding 0.061697968 -1.328511673 1.919710391 0.412602204 0.360213784 -0.113792107 -0.547681052 0.889128912

At5g63400 "ADK1 (ADENYLATE KINASE 1); ATP binding / adenylate kinase/ nucleobase, nucleoside, nucleotide kinase" 0.713532594 0.695293335 0.711965652 0.111712551 0.098843836 0.277377854 -0.454246498 0.406930975

At5g63570 "GSA1 (GLUTAMATE-1-SEMIALDEHYDE-2,1-AMINOMUTASE); glutamate-1-semialdehyde 2,1-aminomutase" -0.303718215 -0.393026979 0.839407407 0.965619639 0.479470086 -0.035358355 -0.863089619 0.080272193

At5g62790 PDE129__DXR (1-DEOXY-D-XYLULOSE 5-PHOSPHATE REDUCTOISOMERASE); 1-deoxy-D-xylulose-5-phosphate reductoisomerase -0.237358544 -1.469904309 0.191138779 -0.612894061 -0.079441253 0.419227908 -0.605286201 -0.335950091

At5g62575 unknown protein 0.243197283 0.548643281 0.044139033 -0.170595911 0.710249206 0.361317381 0.082838627 -0.367489309

At5g61760 IPK2B__ATIPK2BETA; inositol or phosphatidylinositol kinase/ inositol trisphosphate 6-kinase 0.42949616 0.407193927 -0.492894548 0.313144795 -0.044367648 0.612544343 -0.214931524 -0.185156482

At5g61410 emb2728__RPE; catalytic/ ribulose-phosphate 3-epimerase -0.619848535 -1.666843629 0.15449941 0.435282852 0.218421828 -0.176979243 0.465771391 -0.825373911

At5g61580 PFK4 (PHOSPHOFRUCTOKINASE 4); 6-phosphofructokinase 0.139469059 -0.177766422 -0.534617227 -0.358691514 1.1061872 0.090401004 -0.018119531 0.46322392

At5g60620 phospholipid/glycerol acyltransferase family protein -0.05445086 0.339252343 -0.389529975 -0.5190821 0.785875258 0.183038548 -0.081678576 0.125740583

At5g60340 maoC-like dehydratase domain-containing protein -0.049511483 0.326617703 0.480635245 0.552535053 0.503123277 -0.096055693 0.076372332 -0.336890467

At5g60600 "CLB4_CSB3_GCPE_ISPG__HDS (4-HYDROXY-3-METHYLBUT-2-ENYL DIPHOSPHATE SYNTHASE); 4 iron, 4 sulfur cluster binding / 4-hydroxy-3-methylbut-2-en-1-yl diphosphate synthase" -0.548720806 -1.765566375 -0.678455671 -0.012573274 0.193440176 0.104146764 -0.237927784 -0.116936332

At5g60540 ATPDX2_EMB2407__PDX2 (PYRIDOXINE BIOSYNTHESIS 2); glutaminase/ glutaminyl-tRNA synthase (glutamine-hydrolyzing)/ protein heterodimerization -0.474408027 0.451556775 -0.616363188 0.781974321 2.07618918 0.747234281 -0.203642819 0.347865477

At5g59750 "riboflavin biosynthesis protein, putative" -0.923693217 -1.545386159 0.143175732 -0.208735442 -0.493562903 -0.086265623 0.419598264 0.111147776

At5g59290 ATUXS3__UXS3 (UDP-GLUCURONIC ACID DECARBOXYLASE 3); UDP-glucuronate decarboxylase/ catalytic -0.26028804 0.47388103 0.237515768 -1.464244449 0.17339257 0.126782212 0.738949151 1.270213893

At5g59580 UGT76E1 (UDP-GLUCOSYL TRANSFERASE 76E1); UDP-glycosyltransferase/ quercetin 3-O-glucosyltransferase/ quercetin 7-O-glucosyltransferase 0.271960293 0.1111536 0.444684203 0.596468446 -0.353827062 -0.84181729 0.285031977 -0.303791419

At5g59590 UGT76E2 (UDP-GLUCOSYL TRANSFERASE 76E2); UDP-glycosyltransferase/ quercetin 3-O-glucosyltransferase/ quercetin 7-O-glucosyltransferase -0.186954889 0.187948811 0.044463273 -0.390600715 -0.124125787 -0.011716478 -0.04357544 -1.984847613

At5g59440 ATTMPK.1_ATTMPK.2__ZEU1 (ZEUS1); ATP binding / thymidylate kinase 1.226908763 0.27054985 -0.007996472 -0.761678805 1.00573894 -0.438403348 -1.283641412 0.051511524

At5g58860 -1.055048099 2.416575909 0.139801881 -1.420837251 -0.650707906 0.17282826 -1.251284215 -1.306604348

At5g58660 "oxidoreductase, 2OG-Fe(II) oxygenase family protein" 0.224961144 0.485093447 -0.294823057 -0.661237197 0.194556564 -0.214086568 -0.602499786 -2.496621817

At5g58690 phosphoinositide-specific phospholipase C family protein -0.53303689 1.094494471 -0.507649332 0.817556818 -0.346754591 -0.51200223 1.0857338 0.515944149

At5g58700 phosphoinositide-specific phospholipase C family protein 0.737319631 1.565341328 0.627569079 0.997988317 0.82735734 0.017722404 1.197532709 0.58733719

At5g58600 PMR5 (POWDERY MILDEW RESISTANT 5) 1.432188407 -0.013965272 0.865228762 0.596003505 2.70473675 0.488745454 -0.772219399 0.475883289

At5g58730 pfkB-type carbohydrate kinase family protein 0.056256309 0.87854373 -0.875869851 -0.194356314 -0.732317955 -0.291413907 0.634636347 -1.321727885

At5g58670 ATPLC_ATPLC1__PLC1 (PHOSPHOLIPASE C 1); phospholipase C -1.722701744 0.41592691 0.490894504 1.342865942 1.586151673 -2.248658809 -0.416924051 0.409248568

At5g58310 "ATMES18__MES18 (METHYL ESTERASE 18); hydrolase/ hydrolase, acting on ester bonds / methyl indole-3-acetate esterase" -2.848384331 -1.31238602 0.666773433 1.168518353 0.332996626 2.55284357 2.108746574 -0.212958638

At5g57890 "anthranilate synthase beta subunit, putative" 0.819665331 0.476356932 -0.645399035 1.933651427 -0.574349594 -1.632778803 -0.785707152 1.407264308

At5g57815 "cytochrome c oxidase subunit 6b, putative" -0.220417102 0.034305801 -0.622013633 -0.220716595 1.044550755 0.399388861 0.539286833 -0.230122563

At5g57800 FLP1_WAX2_YRE__CER3 (ECERIFERUM 3); binding / catalytic/ iron ion binding / oxidoreductase 2.194239417 -1.968638562 -0.470776339 -1.467137142 -0.908872598 0.387179446 0.127735162 -0.379116163

At5g57850 aminotransferase class IV family protein -0.145290639 -0.353557922 0.233374522 0.533820385 1.044747767 0.495506017 -0.015555882 0.232015554

At5g57440 GPP2__GS1; catalytic/ hydrolase -0.02492922 0.14470283 1.524912847 -0.562052873 0.868108555 0.108327312 0.011007227 0.506310796

At5g57590 BIO1 (biotin auxotroph 1); adenosylmethionine-8-amino-7-oxononanoate transaminase 1.201248388 0.284355394 1.302949351 -0.082142891 -0.386436051 0.341281805 -0.700628248 0.087635437

At5g57655 xylose isomerase family protein 1.567859943 -0.376854855 -1.262970993 -2.565006351 2.245027967 -1.200099032 0.367449142 0.305890937

At5g57030 LUT2 (LUTEIN DEFICIENT 2); lycopene epsilon cyclase 0.138815107 -1.4739071 0.57213573 0.211672923 0.03414097 0.389622853 0.924324287 0.624165017

At5g57190 PSD2 (phosphatidylserine decarboxylase 2); phosphatidylserine decarboxylase -0.227237878 0.729751023 0.011316468 1.592309996 -0.503596254 -1.459082324 1.138765896 0.184324423

At5g57220 CYP81F2; electron carrier/ heme binding / iron ion binding / monooxygenase/ oxygen binding -0.320326846 0.589883486 -0.415014067 1.56791717 -1.505268503 -5.497429685 -2.421385922 1.589994467

At5g56970 ATCKX3__CKX3 (CYTOKININ OXIDASE 3); amine oxidase/ cytokinin dehydrogenase 1.13244444 0.123845559 -0.329034941 0.27270824 -0.270088189 0.196696797 1.088333512 0.151309778

At5g56720 "malate dehydrogenase, cytosolic, putative" -0.30073343 0.406286477 0.589330041 -0.047278384 0.215078606 -0.523765775 0.217965768 0.171167308

At5g56760 SAT-52_SAT5__ATSERAT1;1 (ARABIDOPSIS THALIANA SERINE ACETYLTRANSFERASE 1;1); serine O-acetyltransferase -0.304442364 0.816088629 0.229071748 -0.275017454 -0.289981286 -1.030283669 0.279358319 0.383805058

At5g56630 PFK7 (PHOSPHOFRUCTOKINASE 7); 6-phosphofructokinase 0.019627683 1.088663319 -0.670111242 0.603790874 1.283604754 1.174174684 0.081370573 1.327628236

At5g56650 ILL1; IAA-amino acid conjugate hydrolase/ metallopeptidase -0.338702649 0.300191091 -0.553935807 -0.821771971 0.253406479 1.097439224 -0.700150527 0.427117378

At5g56350 "pyruvate kinase, putative" 0.491490135 0.757183519 -1.440715282 0.863803736 0.061125663 0.573349092 -0.849382799 1.49802706

At5g56330 ATACA8__ACA8 (ALPHA CARBONIC ANHYDRASE 8); carbonate dehydratase/ zinc ion binding -0.0545157 0.248821334 0.327852904 0.255427034 0.255656358 -0.376236193 0.198344256 -0.471660714

At5g55700 BMY6__BAM4 (BETA-AMYLASE 4); beta-amylase/ catalytic/ cation binding 0.96357591 -0.130092629 -0.329103725 -0.188127732 0.807826908 -0.980590146 0.800488607 -0.237262477

At5g56080 ATNAS2__NAS2 (NICOTIANAMINE SYNTHASE 2); nicotianamine synthase -1.045658829 1.74326736 0.517630196 -0.033703993 -0.69125636 0.114116331 -0.141272873 0.058605849

At5g55810 0.109082968 0.086819729 -0.122783711 -0.102156679 0.518927777 -0.158339618 -0.019926013 0.070280471

At5g55590 QRT1 (QUARTET 1); pectinesterase -0.092517875 0.456127859 0.280909919 -0.139848505 -0.027997786 -0.137482523 0.137223349 -0.465327234

At5g55070 2-oxoacid dehydrogenase family protein -0.042461512 0.668826545 -0.306840581 1.439898357 -0.290822376 0.544783621 -0.186721514 -0.253162748

At5g55120 VTC5 (VITAMIN C DEFECTIVE 5); GDP-D-glucose phosphorylase/ galactose-1-phosphate guanylyltransferase (GDP)/ quercetin 4'-O-glucosyltransferase 1.680462176 0.173282042 0.436549938 1.216054691 -1.315497374 -0.875821845 -1.217242038 -0.295887226

At5g55250 IAMT1 (IAA CARBOXYLMETHYLTRANSFERASE 1); S-adenosylmethionine-dependent methyltransferase/ indole acetic acid carboxyl methyltransferase 1.797984777 -0.071891077 0.43392759 -1.075101555 -0.957496334 0.455110079 -0.394836618 -0.777892949

At5g54690 "IRX8_LGT6__GAUT12 (GALACTURONOSYLTRANSFERASE 12); polygalacturonate 4-alpha-galacturonosyltransferase/ transferase, transferring glycosyl groups / transferase, transferring hexosyl groups" -0.660108881 0.387997323 -0.241697916 -1.883596627 -0.257583774 0.128186316 1.093425269 0.54781807

At5g54770 THI4_TZ__THI1; protein homodimerization 0.23850559 -2.093019091 -0.502957257 0.431547424 0.320577792 0.78194195 -0.538210925 -0.861833579

At5g54960 PDC2 (pyruvate decarboxylase-2); carboxy-lyase/ catalytic/ magnesium ion binding / pyruvate decarboxylase/ thiamin pyrophosphate binding -0.394202499 0.630424587 -2.378297226 1.10977657 4.457728386 0.718584722 -1.021962645 -0.393584189

At5g54510 GH3.6__DFL1 (DWARF IN LIGHT 1); indole-3-acetic acid amido synthetase 2.035954458 -0.098319905 -0.359373153 -1.129486186 -0.036373552 -2.012416709 -1.142512108 2.277899647

At5g54060 "UF3GT (udp-glucose:flavonoid 3-O-glucosyltransferase); transferase, transferring glycosyl groups" -1.084325656 0.395011937 -0.515288842 2.771517823 -0.746087523 1.679489656 -0.448074815 -0.590033189

At5g54140 ILL3; IAA-amino acid conjugate hydrolase/ metallopeptidase 0.220143525 0.688695013 -0.485201208 -0.194362205 0.15847129 0.2995009 -0.19389414 -0.017718395

At5g54080 "HGO (HOMOGENTISATE 1,2-DIOXYGENASE); homogentisate 1,2-dioxygenase" 0.486374391 -0.591061606 -3.019474098 -2.613918398 1.271855547 -1.128653548 -0.118906594 -0.398221494

At5g54190 PORA; oxidoreductase/ protochlorophyllide reductase 0.067117872 -0.711651414 0.891453055 -0.826086828 2.057936553 -1.500168115 -0.042708635 -0.253255618

At5g54160 OMT1__ATOMT1 (O-METHYLTRANSFERASE 1); caffeate O-methyltransferase/ myricetin 3'-O-methyltransferase/ quercetin 3-O-methyltransferase 1.11818324 -0.136121928 -0.473922267 -1.970639432 -1.984472405 -0.360543704 -0.957707831 0.464524819

At5g53970 "aminotransferase, putative" 0.021619774 -1.23549657 -2.504424062 -0.189345067 -2.128490912 -0.059826555 -0.290384205 -1.408090254

At5g53990 glycosyltransferase family protein -0.081420311 1.046848143 -0.102422771 -0.17224931 0.164360646 -0.569886884 -0.422121535 -0.208492868

At5g54000 "oxidoreductase, 2OG-Fe(II) oxygenase family protein" -0.071244305 0.497551313 0.117154921 0.673215765 0.49563259 -0.708836989 -0.070587785 -2.784206036

At5g54010 glycosyltransferase family protein 0.320418412 0.522521241 0.62871324 0.717820902 0.190088985 -0.158238724 1.653549118 -0.245150807

At5g53120 SPMS__SPDS3 (SPERMIDINE SYNTHASE 3); spermidine synthase/ spermine synthase 1.246728987 -0.156280772 -2.100058586 -0.056491003 -1.11382335 1.699484091 -0.090939367 0.061495517

At5g53370 ATPMEPCRF__PMEPCRF (PECTIN METHYLESTERASE PCR FRAGMENT F); pectinesterase -3.115148402 -0.484569233 -1.017000651 -0.759287226 -1.20941354 0.723760118 -0.528310852 1.167877505

At5g53460 GLT1; glutamate synthase (NADH) 0.011540188 0.879359595 0.163137905 -0.557062525 1.153159808 0.099911332 -0.732770826 0.532648527

At5g52920 PKP1_PKP2__PKP-BETA1 (PLASTIDIC PYRUVATE KINASE BETA SUBUNIT 1); pyruvate kinase 0.813892594 -0.230280836 1.122199014 -1.164749959 -0.411976304 0.259274768 -0.977940851 -0.689362196

At5g52840 NADH-ubiquinone oxidoreductase-related 0.060244469 0.332384421 0.378544296 -0.177658378 0.271440966 0.572253819 -0.265651464 0.327013337

At5g52560 ATUSP (ARABIDOPSIS THALIANA UDP-SUGAR PYROPHOSPHORYLASE); UTP-monosaccharide-1-phosphate uridylyltransferase/ UTP:arabinose-1-phosphate uridylyltransferase/ UTP:galactose-1-phosphate uridylyltransferase/ UTP:glucose-1-phosphate uridylyltransferase/ UTP:xy 0.184321298 0.344256597 0.351731002 -0.097289632 0.035507161 0.187512799 0.810002872 0.047065843

At5g52570 B2_CHY2__BETA-OHASE 2 (BETA-CAROTENE HYDROXYLASE 2); carotene beta-ring hydroxylase -0.218304896 -1.647003213 0.056037343 0.642974937 -5.500781435 -2.768361295 -0.253468686 -2.053382466

At5g51810 AT2353_ATGA20OX2__GA20OX2 (GIBBERELLIN 20 OXIDASE 2); gibberellin 20-oxidase 0.996295063 0.09173688 0.535969334 -0.608503723 -0.3683046 0.033406403 0.921417483 -1.266707219

At5g51820 ATPGMP_PGM1_STF1__PGM (PHOSPHOGLUCOMUTASE); phosphoglucomutase -0.933962699 -1.156957585 0.841071322 0.944842853 2.236507621 0.682092933 -0.268355899 -0.455555067

At5g51830 pfkB-type carbohydrate kinase family protein 1.701252216 0.97216183 -1.46296245 1.590271669 0.746828237 -0.461953221 -1.777382294 -1.215171132

At5g51970 "sorbitol dehydrogenase, putative / L-iditol 2-dehydrogenase, putative" -1.614932109 -0.212684609 -0.324398246 -1.046988668 1.166611257 -0.25628556 -0.024800968 -0.414926925

At5g51460 ATTPPA; trehalose-phosphatase -0.943106378 0.262628062 2.536903793 1.994770531 -1.856156722 -1.813396997 1.401551175 0.322458397

At5g51490 pectinesterase family protein -0.727432405 0.690369283 0.276332923 -0.69160544 -0.38904395 0.449867012 -0.4619468 -3.859822181

At5g51500 pectinesterase family protein -0.360706326 1.1510686 0.364964447 -0.295613813 0.125120313 -0.273356849 -0.013028066 -0.783929204

At5g51690 ACS12; 1-aminocyclopropane-1-carboxylate synthase/ L-aspartate:2-oxoglutarate aminotransferase/ aromatic-amino-acid:2-oxoglutarate aminotransferase 0.107732066 0.262938281 0.110658844 0.299154104 0.639236462 0.025579208 -0.104877252 -0.056511852

At5g51310 gibberellin 20-oxidase-related -0.730318832 0.750987339 0.531325791 -0.12142794 0.172705844 0.418842857 0.173241955 -0.212791028

At5g51290 ceramide kinase-related -0.236040917 0.31398525 -0.116317019 0.52284166 0.593357487 -0.490835793 0.527385672 -0.574555868

At5g50950 "fumarate hydratase, putative / fumarase, putative" -0.422892995 0.028952349 0.323986645 0.149285646 0.752025993 -0.395282218 0.890767353 2.357908809

At5g50850 MAB1 (MACCI-BOU); catalytic/ pyruvate dehydrogenase (acetyl-transferring) -0.120554472 0.594912838 0.035139889 0.117132659 0.112262943 0.308637222 0.04980998 0.631320388

At5g51100 FSD2 (FE SUPEROXIDE DISMUTASE 2); superoxide dismutase 0.339460358 -0.744898138 1.344022677 1.280889439 0.744784094 0.417070491 -0.565225205 -0.127876853

At5g50370 "adenylate kinase, putative" 0.653532083 0.588601502 1.022466443 -0.252208018 -0.718827845 0.635066703 -0.28664079 -0.243028672

At5g50160 ATFRO8__FRO8 (FERRIC REDUCTION OXIDASE 8); ferric-chelate reductase/ oxidoreductase -0.996807469 -1.573536184 -1.165500356 0.443692795 2.320209378 1.104996959 0.190665952 0.455487132

At5g50210 "OLD5_SUFE3__QS (QUINOLINATE SYNTHASE); 4 iron, 4 sulfur cluster binding / enzyme activator/ protein homodimerization/ quinolinate synthetase A" -1.582476831 0.576963874 0.265878466 1.209886732 0.133899268 -0.649185902 -0.118733898 -0.108088527

At5g49970 PDX3__ATPPOX (A. THALIANA PYRIDOXIN (PYRODOXAMINE) 5'-PHOSPHATE OXIDASE); pyridoxamine-phosphate oxidase -0.455707775 -0.853907834 -0.191641043 -0.550074858 -0.104726341 0.537156696 0.602531105 0.721654762

At5g49690 UDP-glucoronosyl/UDP-glucosyl transferase family protein 0.243732689 0.205121997 -0.55516301 0.320852729 0.478757598 -0.825077808 -0.274625464 -1.192669193

At5g49720 "DEC_IRX2_KOR_KOR1_RSW2__ATGH9A1 (ARABIDOPSIS THALIANA GLYCOSYL HYDROLASE 9A1); cellulase/ hydrolase, hydrolyzing O-glycosyl compounds" -0.157179898 -0.381586201 -0.145797945 -1.771208129 -0.193102283 -0.244841019 -0.882863636 0.762555811

At5g49810 MMT; S-adenosylmethionine-dependent methyltransferase -0.490417462 0.446386778 -0.259641271 0.029317409 -0.279266718 1.156332305 0.573245294 1.481470183

At5g49650 "xylulose kinase, putative" 0.480925979 -0.217837963 -0.311443588 -0.75199637 1.206183069 0.046050209 0.319803305 0.202501835

At5g49180 pectinesterase family protein 1.136170971 -0.719218261 -0.382087669 -1.561032684 -0.912421822 1.220206101 1.199553914 -2.527419263

At5g49330 AtMYB111 (myb domain protein 111); DNA binding / transcription factor 1.751137391 -0.499096665 0.554755251 1.190502742 -0.66750714 1.551726395 0.402110457 0.140747716

At5g49460 ACLB-2 (ATP CITRATE LYASE SUBUNIT B 2); ATP citrate synthase 1.276872016 0.238519413 1.076170116 -1.704550951 -0.475134676 0.191493098 0.24273173 0.383507956

At5g48880 KAT5_PKT1__PKT2 (PEROXISOMAL 3-KETO-ACYL-COA THIOLASE 2); acetyl-CoA C-acyltransferase/ catalytic 1.491821429 -0.682260674 -0.208900606 0.073852853 -1.71259114 2.983416347 0.540047073 0.291994063

At5g49030 OVA2 (ovule abortion 2); ATP binding / aminoacyl-tRNA ligase/ isoleucine-tRNA ligase/ nucleotide binding -0.478771412 -0.681509056 1.409311246 0.668199729 -0.043274251 0.513075656 -0.31436924 -0.00358

At5g48930 HCT (HYDROXYCINNAMOYL-COA SHIKIMATE/QUINATE HYDROXYCINNAMOYL TRANSFERASE); quinate O-hydroxycinnamoyltransferase/ shikimate O-hydroxycinnamoyltransferase/ transferase -0.789141605 0.060384145 -0.190427605 -0.964125173 -1.356324653 -0.381450935 -0.507207106 -0.348154023

At5g49190 "ATSUS2_SSA__SUS2 (SUCROSE SYNTHASE 2); UDP-glycosyltransferase/ sucrose synthase/ transferase, transferring glycosyl groups" -0.205440376 0.16734505 0.04931878 -0.183242157 -0.195710823 0.692821141 -0.751585908 -4.589343666

At5g48840 ATPTS_PTS__PANC (ARABIDOPSIS HOMOLOG OF BACTERIAL PANC); pantoate-beta-alanine ligase/ protein homodimerization -0.103596568 -0.062541502 0.374191175 0.126340398 0.578611824 0.699500588 -0.238589909 0.243464571

At5g48300 APS1__ADG1 (ADP GLUCOSE PYROPHOSPHORYLASE 1); glucose-1-phosphate adenylyltransferase -0.112068965 -1.23827614 0.384027312 1.080578912 0.13224153 0.999399218 -0.074290589 0.44647757

At5g48220 "indole-3-glycerol phosphate synthase, putative" -0.647776483 -1.364949552 0.823975816 0.35584278 0.162435561 -0.457516656 -0.050852928 -1.025189509

At5g48230 EMB1276__ACAT2 (ACETOACETYL-COA THIOLASE 2); acetyl-CoA C-acetyltransferase/ catalytic 1.103409492 0.705030239 0.693787917 -1.000162303 0.12203847 0.757725384 -0.188505842 0.566260876

At5g48370 thioesterase family protein 0.588518502 0.104209546 -0.915534868 0.499247744 1.553194653 0.504770019 0.476865874 0.687308716

At5g48180 NSP5 (NITRILE SPECIFIER PROTEIN 5) 0.80387742 -0.256888043 -2.675266744 -0.130524611 -0.804285871 0.14859611 -0.130793085 -1.096958432

At5g47770 FPS1 (FARNESYL DIPHOSPHATE SYNTHASE 1); dimethylallyltranstransferase/ geranyltranstransferase 0.531537474 0.491880594 1.493252524 -0.810721319 0.508645893 -0.479361058 0.19363594 2.351452695

At5g47780 "GAUT4 (Galacturonosyltransferase 4); polygalacturonate 4-alpha-galacturonosyltransferase/ transferase, transferring glycosyl groups" 0.47071535 -0.428897257 0.613648159 -1.430246167 0.096002248 -0.195581815 -0.304212891 0.250455325

At5g47810 PFK2 (PHOSPHOFRUCTOKINASE 2); 6-phosphofructokinase 0.158992944 0.275633163 -0.706145389 -0.363582168 0.34786049 -0.152121891 -0.405222044 -2.641713311

At5g47990 THAD_THAD1__CYP705A5; oxygen binding / thalian-diol desaturase -1.457482553 2.996815547 0.463009184 -2.467413721 -0.552095294 0.284216941 -0.691679397 1.510076764

At5g48000 CYP708 A2_THAH_THAH1__CYP708A2; oxygen binding / thalianol hydroxylase -1.64480041 3.058907246 0.504805015 -1.942152089 -0.534244028 0.261284881 -0.672727158 1.351127921

At5g48010 THAS1 (THALIANOL SYNTHASE 1); catalytic/ thalianol synthase -1.100051992 2.545696834 0.217788741 -1.557984313 -0.553316818 0.143439608 -0.868490773 1.198326847

At5g48100 LAC15__TT10 (TRANSPARENT TESTA 10); copper ion binding / laccase -0.362118609 0.570433317 0.048702723 -0.390967857 -0.102268681 0.31133229 0.192394431 -5.619481563

At5g47840 "AMK2 (Adenosine monophosphate kinase); ATP binding / adenylate kinase/ nucleobase, nucleoside, nucleotide kinase/ nucleotide kinase/ phosphotransferase, phosphate group as acceptor" -0.748643217 -0.92362663 0.777315956 0.001653409 0.170593525 -0.275870089 0.492181135 0.198914043

At5g47720 "acetyl-CoA C-acyltransferase, putative / 3-ketoacyl-CoA thiolase, putative" 0.521675529 0.326159105 -0.392081433 -2.003332458 0.064835835 0.193515333 -0.650036637 -0.329205949

At5g47760 ATPK5_PGLP2__ATPGLP2 (ARABIDOPSIS THALIANA 2-PHOSPHOGLYCOLATE PHOSPHATASE 2); phosphoglycolate phosphatase/ protein serine/threonine kinase 0.067111436 0.136362488 -0.077444019 -0.127106127 0.662427096 0.448684289 0.502240339 1.597172242

At5g47340 palmitoyl protein thioesterase family protein -0.071392693 0.131568708 0.262445398 0.157323338 0.150436438 -0.237587592 0.140867229 -0.403533816

At5g47350 palmitoyl protein thioesterase family protein 0.326910097 -0.132807357 0.060756504 -0.658226134 -0.44868294 0.128377486 0.758224067 -1.261797925

At5g47435 "formyltetrahydrofolate deformylase, putative" -0.962981911 0.185811225 0.91796712 0.278601376 0.759225783 -0.226986679 0.755403614 0.688058343

At5g47500 pectinesterase family protein 3.617948927 -0.809698596 2.182940811 0.193054709 1.068504517 0.419615286 -0.572777485 0.930773609

At5g47330 palmitoyl protein thioesterase family protein -0.450777433 -0.406806034 -0.475601797 -0.095349168 0.562952041 1.033064712 0.337277152 -2.372610256

At5g46180 delta-OAT; ornithine-oxo-acid transaminase 0.095869316 -0.061372872 -2.711031171 -0.282306456 0.512526818 0.110297581 0.221013876 -0.253611797

At5g46290 KAS I (3-KETOACYL-ACYL CARRIER PROTEIN SYNTHASE I); catalytic/ fatty-acid synthase 0.152014404 0.13648898 1.69551854 -0.351048273 -0.309064299 0.617711882 -0.631669624 -0.53533421

At5g45930 CHL I2_CHLI-2__CHLI2 (MAGNESIUM CHELATASE I2); ATPase/ magnesium chelatase -0.343989384 -2.192061651 1.957851324 0.312205638 0.723951536 -0.20851708 0.164588513 0.250447574

At5g45940 atnudt11 (Arabidopsis thaliana Nudix hydrolase homolog 11); hydrolase 0.213978774 -0.207420843 -0.296855513 -2.260772799 -0.068960306 0.638988107 1.00795967 1.693413563

At5g45340 CYP707A3; (+)-abscisic acid 8'-hydroxylase/ oxygen binding -1.158000891 -0.457354687 0.502076365 -0.23713423 -2.149623743 -4.353230364 0.027587044 0.492320032

At5g45300 BAM8__BMY2 (BETA-AMYLASE 2); beta-amylase/ catalytic/ cation binding 0.508653973 0.18048068 0.665202917 0.230524572 0.170573169 0.125704573 0.079232752 -0.665837194

At5g44630 terpene synthase/cyclase family protein 5.309691193 -0.576191946 -0.020600486 0.056141936 -0.042882237 0.68892216 0.719953452 1.276984587

At5g44030 "IRX5_NWS2__CESA4 (CELLULOSE SYNTHASE A4); cellulose synthase/ transferase, transferring glycosyl groups" -1.025841269 0.159238291 0.033238319 -2.507572159 -0.421071559 0.303316569 1.353166009 1.176011146

At5g43940 ADH2_GSNOR__HOT5 (sensitive to hot temperatures 5); S-(hydroxymethyl)glutathione dehydrogenase/ S-nitrosoglutathione reductase 0.529619042 -0.066354474 -0.481690772 -0.129261174 0.791125521 0.669749689 -1.085497534 0.347060313

At5g43860 ATCLH2; chlorophyllase -0.191945084 -0.049041743 -0.373202128 0.045651587 0.609754195 0.116683539 0.075999209 -1.588895397

At5g43780 APS4; sulfate adenylyltransferase (ATP) -1.359466151 0.174614454 -2.33076128 -2.102923302 0.497668829 0.407317466 -0.048225801 1.163743373

At5g43280 "DCI1__ATDCI1 (DELTA(3,5),DELTA(2,4)-DIENOYL-COA ISOMERASE 1); delta3,5-delta2,4-dienoyl-CoA isomerase/ enoyl-CoA hydratase" 0.270582979 0.059706349 -0.856414148 -0.536318129 0.7475522 0.334457048 -0.051267839 0.24272999

At5g43330 "malate dehydrogenase, cytosolic, putative" -0.345273696 0.463002036 0.194000863 -0.50569398 -0.120258726 0.821916921 1.364179573 0.590208749

At5g42740 "glucose-6-phosphate isomerase, cytosolic (PGIC)" -0.317384727 0.335109702 0.17507513 0.655134285 -0.279165528 0.440474192 0.118055489 -0.755931664

At5g42810 ATIPK1 (Inositol-pentakisphosphate 2-kinase 1); inositol pentakisphosphate 2-kinase/ inositol tetrakisphosphate 2-kinase -0.010361415 -0.290375927 -0.350773265 -0.51421862 -1.192424476 -0.109984175 -0.732964055 -1.026083795

At5g42600 MRN1 (MARNERAL SYNTHASE); catalytic/ marneral synthase -0.70942421 1.288334386 0.494228452 -0.968649118 0.095359123 -0.614406133 -0.322500972 0.131236246

At5g42650 CYP74A__AOS (ALLENE OXIDE SYNTHASE); allene oxide synthase/ hydro-lyase/ oxygen binding -2.061685894 -0.135433794 0.386785266 2.822573625 0.811720768 -2.111926119 -0.825839813 0.302245185

At5g42800 M318_TT3__DFR (DIHYDROFLAVONOL 4-REDUCTASE); dihydrokaempferol 4-reductase -2.025675286 -0.139634461 -0.594069251 2.58767458 -2.67192712 3.108127468 -0.988940034 -3.067515554

At5g42250 "alcohol dehydrogenase, putative" -2.036837381 0.240222932 -0.361609274 -2.918508891 0.382446645 -1.778964272 -0.159160139 1.002945827

At5g41670 6-phosphogluconate dehydrogenase family protein -0.286558576 1.184156415 0.951252837 0.350667968 0.352222473 1.240491564 -0.827204939 0.922825715

At5g41040 transferase family protein -1.206798886 1.940873937 -0.470213066 -1.959259878 -0.92076562 0.959970513 -1.616599602 -3.267931028

At5g41480 ATDFA_DFA__GLA1 (GLOBULAR ARREST1); dihydrofolate synthase 0.230676316 -0.032306954 0.213938452 -0.098902169 0.578549187 0.26656064 -0.173569792 -0.057608926

At5g40870 AtUK/UPRT1 (Uridine kinase/Uracil phosphoribosyltransferase 1); ATP binding / kinase/ uracil phosphoribosyltransferase/ uridine kinase 1.243203393 -0.054145961 0.65365134 -0.614445285 0.673610963 0.235589518 -0.531530876 0.173289246

At5g40850 UPM1 (UROPHORPHYRIN METHYLASE 1); uroporphyrin-III C-methyltransferase -1.705483753 0.559258345 1.154509618 0.642618023 0.245073158 -0.034980315 -0.867155977 0.56488282

At5g40650 SDH2-2; electron carrier/ succinate dehydrogenase 0.249251878 0.618563117 -0.179675431 0.098360704 -0.373918039 -0.016597614 0.401830002 0.919758839

At5g40760 G6PD6 (GLUCOSE-6-PHOSPHATE DEHYDROGENASE 6); glucose-6-phosphate dehydrogenase 1.728130165 0.743101241 -1.568856181 -0.214690654 -0.256256835 0.761480085 -0.64352796 0.583241603

At5g40280 ATFTB_WIG__ERA1 (ENHANCED RESPONSE TO ABA 1); farnesyltranstransferase/ protein farnesyltransferase 0.469454334 0.066321021 -0.168948562 -0.269733725 0.496886306 0.209257184 -0.722452498 -0.032835581

At5g40390 "SIP1 (seed imbibition 1-like); galactinol-sucrose galactosyltransferase/ hydrolase, hydrolyzing O-glycosyl compounds" -1.019659406 1.179204717 0.362245415 0.976367879 -1.165186756 -0.251438635 -0.732387535 -0.056417196

At5g39320 "UDP-glucose 6-dehydrogenase, putative" 0.595918685 1.467343795 2.354287155 -0.853779918 -0.168087447 -0.161971072 0.594207466 1.947751186

At5g38970 ATBR6OX_CYP85A1__BR6OX1 (BRASSINOSTEROID-6-OXIDASE 1); monooxygenase/ oxygen binding 0.023079191 0.218776469 0.280429236 -0.398233433 0.23016541 0.012000639 0.296799744 -0.267385126

At5g38630 ACYB-1; carbon-monoxide oxygenase -0.005414979 0.624583195 0.165715857 -0.568048409 0.64766043 0.314765341 -0.340967136 0.223010238

At5g38520 "hydrolase, alpha/beta fold family protein" -0.809115634 -2.602991961 0.788644243 -0.311891498 -0.62842268 0.324487328 -0.159457398 -0.445914891

At5g38710 "proline oxidase, putative / osmotic stress-responsive proline dehydrogenase, putative" -0.08517471 0.4378253 -2.728130234 0.652398187 -1.3332566 -2.354314833 0.374142606 1.795186602

At5g38830 tRNA synthetase class I (C) family protein 0.422163816 0.503407936 -0.246831239 0.417558674 -0.447076875 0.323153396 -0.365471946 -0.317397756

At5g38120 4-coumarate--CoA ligase family protein / 4-coumaroyl-CoA synthase family protein 1.023507532 -0.167091383 0.405644065 0.452323739 -0.174565976 -0.757103151 1.634294551 0.384342034

At5g38450 CYP735A1; electron carrier/ heme binding / iron ion binding / monooxygenase/ oxygen binding -0.220761572 0.788207846 0.61942318 0.177417246 0.302854272 -0.467964321 0.827060235 -0.50853203

At5g38040 UDP-glucoronosyl/UDP-glucosyl transferase family protein 0.459466658 0.241543478 0.035441682 0.385293548 0.126738114 0.019138249 -0.194665345 -0.50221759

At5g37600 GLN1;1__ATGSR1; copper ion binding / glutamate-ammonia ligase 0.435979931 0.501344679 -2.567314235 -0.447656897 1.553392948 0.829562988 -0.934702074 0.818170798

At5g37830 OXP1 (OXOPROLINASE 1); 5-oxoprolinase (ATP-hydrolyzing)/ hydrolase 0.176884018 0.210001742 -0.363079251 -0.534519886 0.729962326 0.593785775 -0.492058723 0.689080533

At5g37850 SOS4 (SALT OVERLY SENSITIVE 4); kinase/ pyridoxal kinase -0.327202774 -0.13033478 0.12650792 0.193770796 -0.096953104 -0.292513789 -0.626461285 0.799219709

At5g37950 "transferase, transferring hexosyl groups" -0.833595092 1.063010748 0.160239343 -1.236654583 -0.198781145 -0.072760032 -0.334777369 0.760194898

At5g37300 WSD1; diacylglycerol O-acyltransferase/ long-chain-alcohol O-fatty-acyltransferase 3.821430129 -1.141030957 -0.344696595 0.113300228 -0.720937829 1.920984753 0.238932329 1.084523683

At5g37510 "EMB1467 (embryo defective 1467); NADH dehydrogenase (ubiquinone)/ NADH dehydrogenase/ electron carrier/ iron-sulfur cluster binding / oxidoreductase/ oxidoreductase, acting on NADH or NADPH" 0.154558023 0.470791561 -0.276080364 0.078735993 0.093719605 0.696905517 -0.112822367 0.470568648

At5g37180 ATSUS5__SUS5; UDP-glycosyltransferase/ sucrose synthase -0.368460714 0.580691835 0.408507956 -0.173828645 0.341509366 -0.283009827 0.296788808 -0.505068246

At5g36880 "acetyl-CoA synthetase, putative / acetate-CoA ligase, putative" -0.123368142 0.867580002 0.135241329 0.724656586 0.37831452 -0.586170459 0.178341735 0.967761848

At5g36700 ATPGLP1__PGLP1 (2-PHOSPHOGLYCOLATE PHOSPHATASE 1); phosphoglycolate phosphatase -0.581521676 -3.517690592 0.370881893 0.692427833 -0.169924611 0.257113505 0.502972308 0.863397251

At5g36140 CYP716A2; electron carrier/ heme binding / iron ion binding / monooxygenase -0.489982105 0.983976072 0.319043191 -0.392524901 0.023807272 -0.194731363 0.052040628 -0.142419582

At5g36150 ATPEN3 (putative pentacyclic triterpene synthase 3); catalytic/ lupeol synthase 0.274338322 0.354130894 0.389872096 0.026884976 -0.030362233 -0.139935356 0.864242144 -0.140648396

At5g35790 G6PD1 (GLUCOSE-6-PHOSPHATE DEHYDROGENASE 1); glucose-6-phosphate dehydrogenase 0.277291706 -2.874234217 0.64734426 -1.288797588 0.694024473 -0.01797695 -0.622565924 -0.109671688

At5g35550 ATMYB123_ATTT2_MYB123__TT2 (TRANSPARENT TESTA 2); DNA binding / transcription factor -0.014373402 0.02343903 0.219916329 -0.075314385 -0.110764328 0.059586487 0.459664439 -0.898213241

At5g35630 ATGSL1_GLN2__GS2 (GLUTAMINE SYNTHETASE 2); glutamate-ammonia ligase -0.553767475 -1.932942577 0.262916778 -0.386280612 -0.149654153 0.321907492 -0.360407239 2.854766761

At5g24410 glucosamine/galactosamine-6-phosphate isomerase-related -0.619517192 1.507334053 0.46084874 -1.155619209 0.411575576 0.007181958 -0.278047878 -1.243944272

At5g24420 glucosamine/galactosamine-6-phosphate isomerase-related 4.28363612 -1.81839226 2.148126764 1.418574189 -1.78458438 -2.180997573 1.236206894 0.916915536

At5g24520 URM23__TTG1 (TRANSPARENT TESTA GLABRA 1); DNA binding / nucleotide binding / protein binding 0.518898448 -0.674473935 -0.233852354 -0.851992699 0.174758455 -0.302305713 -0.774460709 0.524724027

At5g24530 "DMR6 (DOWNY MILDEW RESISTANT 6); oxidoreductase/ oxidoreductase, acting on paired donors, with incorporation or reduction of molecular oxygen, 2-oxoglutarate as one donor, and incorporation of one atom each of oxygen into both donors" -1.141255116 -0.357029834 -2.579819045 -0.069701587 -0.168370621 0.243473592 -0.691194339 0.97361404

At5g23960 TPS21 (TERPENE SYNTHASE 21); (-)-E-beta-caryophyllene synthase/ alpha-humulene synthase 0.873732111 -0.175995954 -0.12668488 -0.316713058 0.080753565 -0.142233243 0.809874559 0.310873764

At5g24140 SQP2; FAD binding / oxidoreductase/ squalene monooxygenase -0.630431952 1.133372156 0.265730924 -0.497379039 -0.491175858 -0.301947558 -0.073484689 0.176486108

At5g24150 SQP1; squalene monooxygenase -1.78001069 -2.765803512 -1.339047008 -0.472984622 -1.933359944 1.526123107 2.311010201 0.886996872

At5g24160 SQE6 (SQUALENE MONOXYGENASE 6); FAD binding / oxidoreductase/ squalene monooxygenase -1.737798783 -0.836841485 -2.170206798 0.532583596 0.984105929 -0.176335028 -0.70052625 0.29803298

At5g23670 LCB2; protein binding / serine C-palmitoyltransferase 0.226742713 0.542197656 0.745909375 -0.317434025 0.958632755 -0.490583733 0.881100735 0.243871486

At5g23790 "AtGolS5 (Arabidopsis thaliana galactinol synthase 5); transferase, transferring glycosyl groups / transferase, transferring hexosyl groups" 0.043740297 0.263399131 0.224001653 0.298382601 0.290282985 -0.298547653 0.284649946 -1.116464269

At5g23310 FSD3 (FE SUPEROXIDE DISMUTASE 3); superoxide dismutase 0.402502244 -0.567030189 1.518546055 0.987007746 0.637726498 1.236765931 -0.62767863 -0.323763427

At5g23250 "succinyl-CoA ligase (GDP-forming) alpha-chain, mitochondrial, putative / succinyl-CoA synthetase, alpha chain, putative / SCS-alpha, putative" 0.808571765 0.613101292 0.769040697 0.356506073 0.330916386 0.839138651 -0.274098561 0.389107669

At5g23300 PYRD (pyrimidine d); dihydroorotate dehydrogenase 0.5634384 0.94474124 1.262436179 0.982617533 0.217210622 0.681522248 -0.795475822 -0.713148927

At5g23220 NIC3 (NICOTINAMIDASE 3); catalytic/ nicotinamidase -0.365820257 1.568797269 0.006903476 -0.627212125 -0.270684737 -0.25638297 -0.446382071 0.434452105

At5g23230 NIC2 (NICOTINAMIDASE 2); catalytic/ nicotinamidase 0.025662279 0.228360093 -0.898017374 -0.501795277 0.894910203 -0.370951727 0.297300822 -1.773030152

At5g23260 ABS_AGL32__TT16 (TRANSPARENT TESTA16); transcription factor 0.612930829 -0.093534301 0.265378803 -0.205337573 0.001854021 -0.084576072 0.450634486 -0.561761434

At5g23010 IMS3__MAM1 (METHYLTHIOALKYLMALATE SYNTHASE 1); 2-isopropylmalate synthase/ methylthioalkylmalate synthase -6.137926235 -0.467307438 0.653133318 -0.23951429 1.116545897 0.638456832 -0.040026108 0.199926072

At5g23020 MAM-L_MAM3__IMS2 (2-ISOPROPYLMALATE SYNTHASE 2); 2-isopropylmalate synthase/ methylthioalkylmalate synthase -4.339532398 2.479574441 0.355020392 -1.279648374 -0.335091066 1.980763684 -0.079138158 0.508290949

At5g23050 AAE17 (ACYL-ACTIVATING ENZYME 17); catalytic/ ligase -0.111388296 0.538940688 -1.10166237 -0.304726209 2.079804887 0.260243851 0.03688423 -0.028787922

At5g22500 "FAR1 (FATTY ACID REDUCTASE 1); fatty acyl-CoA reductase (alcohol-forming)/ oxidoreductase, acting on the CH-CH group of donors" 3.648250243 0.100854307 -0.264150696 -0.772344029 0.054608483 -1.302334513 -1.422769435 -0.407423141

At5g22510 "beta-fructofuranosidase, putative / invertase, putative / saccharase, putative / beta-fructosidase, putative" -0.370974223 -0.91004213 -0.755367923 -0.226082748 -0.237333078 0.022550167 -0.250779517 0.352598508

At5g22630 ADT5 (arogenate dehydratase 5); arogenate dehydratase/ prephenate dehydratase 0.778613414 0.908987215 -1.196414264 -0.263609174 -1.946338858 -1.035979246 -0.661956303 0.537248065

At5g19220 ADG2__APL1 (ADP GLUCOSE PYROPHOSPHORYLASE LARGE SUBUNIT 1); glucose-1-phosphate adenylyltransferase -0.756629787 -2.921154025 0.92865057 0.06390292 -0.43521856 -0.49476895 -0.790310282 0.073684761

At5g22420 "FAR7 (FATTY ACID REDUCTASE 7); binding / catalytic/ oxidoreductase, acting on the CH-CH group of donors" 0.139381053 0.232009433 0.111724214 0.191434255 0.090958761 -0.243441152 0.445226163 -0.310372072

At5g22300 NIT4 (NITRILASE 4); 3-cyanoalanine hydratase/ cyanoalanine nitrilase/ indole-3-acetonitrile nitrilase/ nitrilase/ nitrile hydratase 0.026805456 -1.521643793 -3.031970283 0.452198568 -1.644979424 0.888759002 0.407520327 0.071409741

At5g18930 SAMDC4__BUD2 (BUSHY AND DWARF 2); adenosylmethionine decarboxylase 0.564701912 0.017941554 0.637312861 -0.457456483 -0.053518783 -0.393930989 -0.294458749 -0.370211336

At5g18990 pectinesterase family protein -0.030352016 0.263474397 0.483084232 0.325067076 0.384293728 -0.517813872 0.51114974 -0.529482608

At5g19040 "ATIPT5__IPT5; ATP binding / tRNA isopentenyltransferase/ transferase, transferring alkyl or aryl (other than methyl) groups" -0.463215386 0.930740065 0.417575682 -0.504150177 0.184651484 -0.480522466 -0.018101715 -0.085416704

At5g18640 lipase class 3 family protein -0.148224806 0.027422605 -1.232279696 -1.305405749 2.204873082 -0.137806749 -0.033147079 -0.255930032

At5g18660 "PCB2 (PALE-GREEN AND CHLOROPHYLL B REDUCED 2); 3,8-divinyl protochlorophyllide a 8-vinyl reductase" 0.025679464 -2.407439429 2.423358307 0.871592295 0.228791953 0.312882128 -0.435757074 0.017403195

At5g18630 lipase class 3 family protein -0.205387614 -0.649297265 -0.817435205 -2.154223856 -0.177515851 -2.116339683 0.090236346 -0.22863745

At5g17990 pat1__TRP1 (tryptophan biosynthesis 1); anthranilate phosphoribosyltransferase 0.019094751 -0.19696342 -0.564272306 2.211349282 -0.170130038 -0.392670289 -1.887413548 1.682083861

At5g18070 "DRT101 (DNA-DAMAGE-REPAIR/TOLERATION 101); intramolecular transferase, phosphotransferases / magnesium ion binding" 0.719715295 0.529054928 0.146749702 0.03474944 -0.183404099 0.185256846 -0.403316916 0.060745249

At5g18100 CSD3 (copper/zinc superoxide dismutase 3); superoxide dismutase -0.08606737 -0.46364521 0.379013608 -0.253365997 0.851697128 -0.355792816 -0.351643853 -0.75030423

At5g18200 UTP:galactose-1-phosphate uridylyltransferase/ ribose-5-phosphate adenylyltransferase 0.016947863 0.40284785 -0.276960739 0.127074341 0.18219384 0.458618087 0.229590292 -0.486440329

At5g18170 GDH1 (GLUTAMATE DEHYDROGENASE 1); ATP binding / glutamate dehydrogenase [NAD(P)+]/ oxidoreductase 0.422660104 0.057533316 0.130780415 -1.576145226 1.185999391 -1.783846024 -1.782295229 0.514243248

At5g17310 "UTP--glucose-1-phosphate uridylyltransferase, putative / UDP-glucose pyrophosphorylase, putative / UGPase, putative" -1.189660015 -0.278555388 0.44587268 0.199294715 -1.110037793 0.319231166 1.079943256 -2.442308405

At5g17200 glycoside hydrolase family 28 protein / polygalacturonase (pectinase) family protein 0.332468917 0.146331603 0.232745814 0.18127802 0.2511336 -0.231052751 0.150197851 -0.377064808

At5g17220 GST26_TT19__ATGSTF12 (ARABIDOPSIS THALIANA GLUTATHIONE S-TRANSFERASE PHI 12); glutathione transferase -0.598142267 -0.493423897 -1.07813466 2.318802827 -2.491925192 3.947481182 -0.391472264 -1.160319905

At5g17330 GAD1__GAD; calmodulin binding / glutamate decarboxylase -1.504896163 2.237620304 -1.476292478 -2.345312251 -1.264438091 -0.769606866 -0.993978233 2.715395807

At5g17230 PSY__phytoene synthase (PSY) / geranylgeranyl-diphosphate geranylgeranyl transferase -0.722110832 -2.188017474 -0.280357626 -0.430773164 -1.089202001 0.619729346 0.647750143 0.775281737

At5g16570 GLN1;4; glutamate-ammonia ligase -1.1583472 -0.04997023 -1.253458429 -0.420016905 0.683110411 1.031284346 0.105661904 0.427482306

At5g16580 -0.298583335 0.211137725 0.395661463 0.449714568 0.189066921 -0.382629482 0.333297553 -0.530793845

At5g16440 IPP1 (ISOPENTENYL DIPHOSPHATE ISOMERASE 1); isopentenyl-diphosphate delta-isomerase 0.915526588 0.106316631 -0.102857159 -1.082147655 0.792655548 0.095626483 0.647895187 1.371563288

At5g16390 BCCP_BCCP-1_BCCP1_CAC1-A_CAC1A__CAC1 (CHLOROPLASTIC ACETYLCOENZYME A CARBOXYLASE 1); acetyl-CoA carboxylase/ biotin binding 0.26298703 -0.430319911 1.622165955 -0.90065837 -0.383552702 -0.142252175 -0.254059121 0.128872581

At5g14700 cinnamoyl-CoA reductase-related 2.094414767 -0.20246714 -0.576131846 2.445945085 -1.436957743 -0.849404726 -0.122688915 0.918842992

At5g13930 ATCHS_CHS__TT4 (TRANSPARENT TESTA 4); naringenin-chalcone synthase 0.663785484 -0.351719037 -0.256341615 0.44830489 -5.265188493 4.645353129 -0.339209918 0.379713476

At5g14220 hemg2_MEE61__HEMG2; electron carrier/ oxidoreductase/ protoporphyrinogen oxidase 0.369197264 -0.219811522 0.44243123 -0.155723422 0.733072922 0.022986851 -0.844728568 -0.011824738

At5g13630 CCH_CCH1_CHLH__GUN5 (GENOMES UNCOUPLED 5); magnesium chelatase 0.40532852 -3.879122264 0.343153473 -1.891967569 -1.798371223 0.113216605 -0.842428795 0.864030103

At5g13700 APAO_PAO1__ATPAO1 (ARABIDOPSIS THALIANA POLYAMINE OXIDASE 1); FAD binding / polyamine oxidase 0.148663079 0.704644361 0.429824078 1.009498781 -0.015150574 -1.274784756 0.931879041 -1.364053896

At5g13640 ATPDAT; phosphatidylcholine-sterol O-acyltransferase -0.40664349 -0.034218208 -0.333671651 -0.526142288 -0.182156361 0.043222878 0.920904405 1.072218296

At5g12890 UDP-glucoronosyl/UDP-glucosyl transferase family protein -0.763070549 -0.217790344 -0.898955789 1.346712768 0.574376597 -0.711288648 0.989100859 0.888012427

At5g13280 AK1__AK-LYS1 (ASPARTATE KINASE 1); aspartate kinase 0.236512992 0.406595414 0.85702433 0.78428685 -0.563700376 0.096519076 -0.616960005 0.239214454

At5g12200 dihydropyrimidinase / DHPase / dihydropyrimidine amidohydrolase / hydantoinase (PYD2) -0.358310898 0.398721723 -1.31761737 -0.850359176 1.543541539 0.653403816 -0.539011115 1.259737531

At5g11770 "NADH-ubiquinone oxidoreductase 20 kDa subunit, mitochondrial" -0.285529124 0.425347994 -0.455099367 -0.265306095 0.063687227 0.207566409 0.045670548 0.084239474

At5g11650 "hydrolase, alpha/beta fold family protein" 0.050174811 0.197131494 -0.487215232 0.589562198 -3.331076065 -2.085515011 0.038532276 -0.895239359

At5g11720 alpha-glucosidase 1 (AGLU1) -0.151835993 -0.414901682 -0.097392045 -1.160679498 1.050514616 0.026100335 0.026772359 1.25233059

At5g11380 DXPS3 (1-deoxy-D-xylulose 5-phosphate synthase 3); 1-deoxy-D-xylulose-5-phosphate synthase 0.288628128 0.015286916 0.376935327 0.322842538 -0.048110803 0.351220115 0.304840066 -0.475035549

At5g11520 YLS4__ASP3 (ASPARTATE AMINOTRANSFERASE 3); L-aspartate:2-oxoglutarate aminotransferase 0.106831241 0.557537454 -1.505826647 0.393384057 0.87628278 -0.09286673 0.364899906 0.177675237

At5g10920 "argininosuccinate lyase, putative / arginosuccinase, putative" 0.328127532 0.213847191 1.423915696 0.509212555 -0.509635684 0.472608499 -0.534398571 -0.155359299

At5g10870 ATCM2 (chorismate mutase 2); chorismate mutase -0.016963847 0.416100507 -0.107732112 -0.252626617 0.915994908 0.528560912 -0.376557783 0.093498531

At5g11160 APT5 (Adenine phosphoribosyltransferase 5); adenine phosphoribosyltransferase 2.421513781 -0.119246385 2.023444475 0.540061476 -1.422387146 -0.116250613 -1.576375609 -1.057149152

At5g10480 PEP__PAS2 (PASTICCINO 2); enoyl-CoA hydratase/ protein tyrosine phosphatase 1.303127721 -0.206568634 0.60173738 -1.300908761 -0.286501007 0.916274415 -0.534955098 0.111492152

At5g10100 "trehalose-6-phosphate phosphatase, putative" 0.547618057 0.729146321 0.083481104 -0.042840507 -0.150759608 -0.41442902 -0.322847075 -0.278422767

At5g10170 ATMIPS3__MIPS3 (MYO-INOSITOL-1-PHOSTPATE SYNTHASE 3); binding / catalytic/ inositol-3-phosphate synthase -0.616909265 1.149840286 1.935966331 0.245375632 0.249560136 -0.152369871 -1.022244417 0.813326914

At5g10240 ASN3 (ASPARAGINE SYNTHETASE 3); asparagine synthase (glutamine-hydrolyzing) 0.480694621 0.701183657 1.554312313 0.396477701 -0.662207706 0.666560111 -0.200574278 0.213119559

At5g09760 pectinesterase family protein 0.350865556 0.007951787 1.187020464 -1.214667928 -0.964906476 0.756976304 0.018338958 -0.346682299

At5g09650 AtPPa6 (Arabidopsis thaliana pyrophosphorylase 6); inorganic diphosphatase/ pyrophosphatase -0.533332102 -0.306728763 1.727635905 0.439165884 -0.323491872 0.221553178 -0.532348528 2.00345951

At5g09660 PMDH2 (peroxisomal NAD-malate dehydrogenase 2); malate dehydrogenase -1.042472439 -3.838418502 -0.101136899 0.354511629 -0.235119334 -0.185544685 0.369058955 1.199704717

At5g09640 SNG2__SCPL19; serine-type carboxypeptidase/ sinapoyltransferase -0.124626602 0.201332637 -0.183744697 -0.421166824 -0.293386152 0.341272567 -0.841960916 -4.406687037

At5g09870 "CESA5 (CELLULOSE SYNTHASE 5); cellulose synthase/ transferase, transferring glycosyl groups" -0.528036895 -0.528605594 0.842931841 0.454564706 -1.190481165 0.738712414 -0.475696574 -0.189583311

At5g08570 "pyruvate kinase, putative" -0.067932236 0.214245047 -0.118150024 -0.200725696 0.347852479 1.284725654 -0.77007366 -0.637767372

At5g08640 FLS (FLAVONOL SYNTHASE); flavonol synthase 1.888449032 -0.00671075 0.746301594 0.383245312 -4.304668208 4.55852776 -0.360109935 0.390720555

At5g08530 "CI51 (51 kDa subunit of complex I); 4 iron, 4 sulfur cluster binding / FMN binding / NAD or NADH binding / NADH dehydrogenase (ubiquinone)/ oxidoreductase, acting on NADH or NADPH" 0.093335341 0.086105708 -0.07026927 -0.117602769 -0.000800081 0.404723446 -0.043991108 0.533584295

At5g08100 L-asparaginase / L-asparagine amidohydrolase 0.825950743 -0.015005135 -0.325622398 -0.689244777 1.357728386 0.790093492 -0.698445903 0.580852725

At5g07990 CYP75B1_D501__TT7 (TRANSPARENT TESTA 7); flavonoid 3'-monooxygenase/ oxygen binding 1.553550164 -0.076934881 -0.595150033 -0.3903209 -2.261782047 2.865684395 0.353816009 -1.318774808

At5g08170 ATAIH__EMB1873 (EMBRYO DEFECTIVE 1873); agmatine deiminase 0.361933289 0.009484067 -0.373210504 0.05144288 -0.391553458 0.844600047 0.416206377 0.211521646

At5g07440 GDH2 (GLUTAMATE DEHYDROGENASE 2); ATP binding / glutamate dehydrogenase [NAD(P)+]/ glutamate dehydrogenase/ oxidoreductase 0.781169778 1.228554983 -0.581276108 -0.946135244 0.455849419 -3.960689602 0.018000166 1.527702834

At5g07410 pectinesterase family protein 1.27968978 -0.150211928 -0.796894653 -0.788170443 -0.504485557 0.660201139 7.418493544 0.777435547

At5g07370 ATIPK2A__IPK2a (INOSITOL POLYPHOSPHATE KINASE 2 ALPHA); inositol or phosphatidylinositol kinase/ inositol trisphosphate 6-kinase -0.807074391 -0.036897792 -0.241360175 0.079445776 0.516623123 0.288501959 -0.37097529 -0.113954322

At5g07420 pectinesterase family protein 0.026473501 0.665573074 0.375910505 0.286974933 0.184391483 -0.113957041 5.229562247 0.114859967

At5g07200 ATGA20OX3_GA20OX3__YAP169; gibberellin 20-oxidase -0.287796176 0.109573915 -0.146409811 -0.705157895 -0.583741954 0.869362717 -0.669887135 -4.70294074

At5g07430 pectinesterase family protein 0.780683043 0.046881482 -0.668449154 -0.6152885 -0.487347385 0.439278079 6.828647754 0.350967125

At5g07480 "oxidoreductase, 2OG-Fe(II) oxygenase family protein" -0.088753907 0.234342014 0.382756872 0.326634816 0.163180605 -0.354312136 0.293570075 -0.709005376

At5g07010 ST2A (SULFOTRANSFERASE 2A); hydroxyjasmonate sulfotransferase/ sulfotransferase 0.857799172 0.592152603 -3.03962353 0.144676752 0.93313191 -1.220245886 -0.495775941 -1.356280193

At5g06580 FAD linked oxidase family protein -0.422420331 -0.332281135 -0.063900457 0.08937325 0.440864112 -0.119390557 -0.313007788 -0.259181664

At5g05730 AMT1_TRP5_WEI2__ASA1 (ANTHRANILATE SYNTHASE ALPHA SUBUNIT 1); anthranilate synthase 0.733715467 0.031000771 -0.857297092 1.414317259 -0.037075213 -2.114994671 -1.793793383 1.834319787

At5g05880 UDP-glucoronosyl/UDP-glucosyl transferase family protein -0.233475679 1.019313547 0.23077556 -0.755303638 0.100030186 -0.612268508 -0.401038014 -0.19982422

At5g05900 UDP-glucoronosyl/UDP-glucosyl transferase family protein -0.240675705 0.863566332 0.500750299 0.25613221 0.172387969 -0.136203633 0.091729099 -0.079022971

At5g05870 "UGT76C1 (UDP-glucosyl transferase 76C1); UDP-glycosyltransferase/ cis-zeatin O-beta-D-glucosyltransferase/ cytokinin 7-beta-glucosyltransferase/ cytokinin 9-beta-glucosyltransferase/ trans-zeatin O-beta-D-glucosyltransferase/ transferase, transferring gly" -1.177915567 -0.131945441 -0.134095139 -0.06495483 0.513382485 -0.922836371 -1.298288057 -2.203336292

At5g05890 UDP-glucoronosyl/UDP-glucosyl transferase family protein -1.170824557 0.805808416 1.119058844 -0.930047623 1.265222832 -0.426708274 -0.039441895 0.037639252

At5g05690 CBB3_CYP90_CYP90A_CYP90A1_DWF3__CPD (CONSTITUTIVE PHOTOMORPHOGENIC DWARF); electron carrier/ heme binding / iron ion binding / monooxygenase/ oxygen binding 0.326055073 -1.563350621 -0.406911045 -1.523525756 1.294025159 -0.68249522 -0.493358966 0.015407836

At5g05860 "UGT76C2; UDP-glycosyltransferase/ cis-zeatin O-beta-D-glucosyltransferase/ cytokinin 7-beta-glucosyltransferase/ cytokinin 9-beta-glucosyltransferase/ trans-zeatin O-beta-D-glucosyltransferase/ transferase, transferring glycosyl groups" -0.525195543 0.474043218 1.314539499 -0.335199179 -1.048838383 -0.844388441 -1.0719786 0.064632306

At5g05980 DFB__ATDFB (A. THALIANA DHFS-FPGS HOMOLOG B); tetrahydrofolylpolyglutamate synthase -0.090909277 0.388709497 -0.074780511 -0.843451172 -0.095203786 0.414673146 -0.562464497 -0.030944245

At5g05260 "CYP79A2 (CYTOCHROME P450 79A2); oxidoreductase, acting on paired donors, with incorporation or reduction of molecular oxygen, NADH or NADPH as one donor, and incorporation of one atom of oxygen / oxygen binding" 0.033757745 0.238200291 0.314495707 0.297647322 0.246872665 -0.343780304 0.334630059 -0.681058955

At5g05600 "oxidoreductase, 2OG-Fe(II) oxygenase family protein" -0.320906709 0.090801127 -1.972248212 0.676430777 -1.029032917 -3.808047622 0.645060684 0.599219955

At5g05270 chalcone-flavanone isomerase family protein 0.960154485 0.110567809 0.658759453 0.350460208 -4.136751016 3.943093251 -0.592774793 -0.163132981

At5g04960 pectinesterase family protein -1.371947699 2.162192449 0.799093256 -2.175983276 -0.255011247 0.558679473 -0.022603015 0.793278489

At5g04970 "pectinesterase, putative" -0.777963163 1.255995533 0.619129748 -0.834712906 0.420996887 -0.081600585 0.242223602 0.352963033

At5g05170 "ATCESA3_ATH-B_CESA3_IXR1__CEV1 (CONSTITUTIVE EXPRESSION OF VSP 1); cellulose synthase/ transferase, transferring glycosyl groups" -0.11509142 -0.08264732 0.427147377 -1.235825219 0.13097454 -0.24535394 -0.961035521 0.159641412

At5g04950 ATNAS1__NAS1 (NICOTIANAMINE SYNTHASE 1); nicotianamine synthase -4.670604062 1.418275066 0.219233195 -1.055385037 -1.846290144 1.509738774 -0.900126575 1.408479097

At5g04620 ATBIOF (BIOTIN F); 8-amino-7-oxononanoate synthase/ transaminase 0.76860035 0.344028156 1.185760091 0.077016212 -0.054265014 0.514877433 0.13866181 -0.720092022

At5g04490 VTE5 (vitamin E pathway gene5); phosphatidate cytidylyltransferase/ phytol kinase -0.47265888 -1.99014169 -0.493976467 -1.000420066 0.309516874 -1.114354701 -0.547077591 0.019555703

At5g04590 SIR; sulfite reductase (ferredoxin)/ sulfite reductase -1.001689068 0.265757721 0.273047322 0.425973924 -0.607243116 -0.081942727 -1.131420387 -0.096017634

At5g03860 MLS (MALATE SYNTHASE); malate synthase -0.131518275 0.72659023 -0.052083205 -0.363047212 0.243694143 -0.407799984 -1.049203727 -4.416566356

At5g04040 SDP1 (SUGAR-DEPENDENT1); triacylglycerol lipase -0.099645775 -0.164067813 -0.494069459 -1.033970821 0.51156901 -2.321055295 0.1742954 -0.996088479

At5g03300 ADK2 (ADENOSINE KINASE 2); adenosine kinase/ copper ion binding / kinase 0.035985448 0.773454035 1.673631285 0.095474263 -0.244346575 0.95739695 -0.592209585 1.167718531

At5g03650 "SBE2.2 (starch branching enzyme 2.2); 1,4-alpha-glucan branching enzyme" -0.040357408 -1.0953085 0.709140926 -0.090133834 -0.15622544 0.636360965 0.325406892 0.326357634

At5g03630 ATMDAR2; monodehydroascorbate reductase (NADH) -0.207591942 1.099786529 -0.935428487 0.807666781 0.008075227 -0.223359657 -0.487478767 2.223162118

At5g03690 "fructose-bisphosphate aldolase, putative" -0.168035167 0.381790752 0.546402838 0.644491893 0.135805177 -0.946637076 2.341371551 -0.04066052

At5g03290 "isocitrate dehydrogenase, putative / NAD+ isocitrate dehydrogenase, putative" -0.110816377 0.869616554 -0.71022947 0.825994734 0.380305895 0.40717446 -0.116057146 0.946469215

At5g03490 UDP-glucoronosyl/UDP-glucosyl transferase family protein -0.597823717 -0.216645358 -1.32447932 0.508231729 -0.078236169 -0.987891972 -0.299571115 -0.280935027

At5g01320 "pyruvate decarboxylase, putative" -0.787033237 1.171388116 0.301451323 0.074390895 0.566535069 -0.181045807 0.045289023 0.406662594

At3g63410 "VTE3__APG1 (ALBINO OR PALE GREEN MUTANT 1); 2-methyl-6-phytyl-1,4-benzoquinone methyltransferase/ S-adenosylmethionine-dependent methyltransferase/ methyltransferase" -0.018374615 -1.595415534 0.910025493 -0.191120096 -0.28889118 0.550056698 -0.841020418 -0.143811583

At5g01220 "SQD2 (sulfoquinovosyldiacylglycerol 2); UDP-glycosyltransferase/ UDP-sulfoquinovose:DAG sulfoquinovosyltransferase/ transferase, transferring glycosyl groups" -1.80469542 -0.454904198 -0.246969933 -0.260094091 0.59432569 0.348501919 -0.26157589 1.161936142

At3g63520 ATCCD1_ATNCED1_NCED1__CCD1 (CAROTENOID CLEAVAGE DIOXYGENASE 1); 9-cis-epoxycarotenoid dioxygenase -1.005524181 -1.25506362 -1.019323488 -1.009607682 -0.289033432 0.371166001 0.061973499 -0.166381984

At3g63110 "IPT3__ATIPT3 (ARABIDOPSIS THALIANA ISOPENTENYLTRANSFERASE 3); ATP binding / tRNA isopentenyltransferase/ transferase, transferring alkyl or aryl (other than methyl) groups" -2.564960191 0.709920029 1.012429873 0.084248442 0.315559928 0.130008941 -0.015356859 0.106249872

At3g63250 ATHMT-2_HMT-2__HMT2 (HOMOCYSTEINE METHYLTRANSFERASE 2); homocysteine S-methyltransferase 1.108797971 0.3554035 0.590151446 -0.521357441 0.403056331 0.442232946 -0.624829473 0.113900997

At3g63440 ATCKX6_ATCKX7__CKX6 (CYTOKININ OXIDASE/DEHYDROGENASE 6); cytokinin dehydrogenase 1.474338899 0.22179718 1.451005925 0.874316222 -0.44993654 -1.405330909 -0.788908158 0.190451122

At3g62720 "ATXT1_XXT1__XT1 (XYLOSYLTRANSFERASE 1); UDP-xylosyltransferase/ transferase/ transferase, transferring glycosyl groups / xyloglucan 6-xylosyltransferase" 0.628744297 -0.007341083 1.769724915 0.481893415 -1.201543216 -3.730173104 0.233448382 -0.427491299

At3g62410 CP12-2; protein binding -2.582167754 -3.999781215 -1.48135319 0.497798647 0.470131219 0.859195537 0.698649289 1.564254461

At3g62610 PFG2__ATMYB11 (MYB DOMAIN PROTEIN 11); DNA binding / transcription factor 0.981491584 -0.149574062 0.075489655 0.23968569 -0.471085133 0.446325092 0.154509868 -0.56251525

At3g62740 "BGLU7 (BETA GLUCOSIDASE 7); catalytic/ cation binding / hydrolase, hydrolyzing O-glycosyl compounds" -0.109743272 0.903436418 -0.010900092 -0.632689958 -0.329650882 -0.323469371 -0.530086468 0.293356304

At3g62750 -1.48064915 -1.257770386 0.575379651 0.283116216 2.978132672 -0.362899799 -0.192088466 -0.726016397

At2g47650 UXS4 (UDP-XYLOSE SYNTHASE 4); UDP-glucuronate decarboxylase/ catalytic 0.434683316 1.1219663 -0.065473414 0.04880962 0.106248984 0.083707584 -0.185328497 0.333068889

At3g62170 VGDH2 (Vanguard 1 homolog 2); enzyme inhibitor/ pectinesterase 0.893683148 0.136555168 -0.264230639 -0.333218541 -0.309770636 0.210018661 6.389541832 0.499294439

At3g62000 O-methyltransferase family 3 protein 0.397241855 -0.288414784 0.85981057 0.260545078 0.058308828 -0.084729378 -0.688762872 0.735729533

At3g61880 CYP78A9 (CYTOCHROME P450 78A9); monooxygenase/ oxygen binding -0.768479572 0.136624542 -0.700858366 -0.740438264 0.234299267 0.178291602 -0.046860243 -3.308014468

At3g61130 "LGT1__GAUT1 (GALACTURONOSYLTRANSFERASE 1); polygalacturonate 4-alpha-galacturonosyltransferase/ transferase, transferring glycosyl groups" -0.048381097 0.326928988 0.936965116 0.113109151 0.32129155 0.300709316 0.588367389 -0.032072995

At3g61530 PANB2; 3-methyl-2-oxobutanoate hydroxymethyltransferase 0.231607856 0.315857121 0.050128758 0.408751251 0.551662807 0.037660187 -0.366710488 -0.441429133

At3g61440 ARATH;BSAS3;1_ATCYSC1__CYSC1 (CYSTEINE SYNTHASE C1); L-3-cyanoalanine synthase/ cysteine synthase -0.143436961 -0.677857481 -0.661863361 -1.100228941 -0.681763962 -0.243268913 -1.454365392 1.485800601

At3g61580 delta-8 sphingolipid desaturase (SLD1) 0.041722233 -0.330829762 1.532025858 -0.832807599 0.412712879 -1.56703838 1.403759327 0.74081549

At3g60620 phosphatidate cytidylyltransferase family protein -0.262691416 -0.343183346 0.857389433 0.410715724 0.412944605 0.542541664 -0.159892824 -0.375329887

At3g60730 pectinesterase family protein 0.053935037 0.421884308 -0.008020473 0.254804354 0.332767684 -0.276520431 -0.757746601 -4.701801479

At3g60880 dihydrodipicolinate synthase 1 (DHDPS1) (DHDPS) (DHPS1) 0.179485159 0.348775712 0.461200636 0.400733196 0.217708338 0.098766247 -0.644740266 -0.547653495

At3g60750 "transketolase, putative" -0.718449315 -0.908084277 0.272971757 -0.108420217 0.093428609 0.84327798 -0.414080616 0.911933827

At3g60290 "oxidoreductase/ oxidoreductase, acting on paired donors, with incorporation or reduction of molecular oxygen, 2-oxoglutarate as one donor, and incorporation of one atom each of oxygen into both donors" -0.318749969 -1.079903015 1.236824906 -0.909745914 0.952653143 -1.871078316 0.512154224 -2.525292576

At3g60510 enoyl-CoA hydratase/isomerase family protein 0.788895701 -0.139253688 0.662303892 -0.502665551 0.85408422 -0.165688892 0.217997027 0.345911912

At3g60180 "uridylate kinase, putative / uridine monophosphate kinase, putative / UMP kinase, putative" -0.473887727 -0.120100868 -1.409208067 -0.250062692 1.0049996 0.125510889 0.04053122 -0.13100752

At3g60100 "CSY5 (citrate synthase 5); citrate (SI)-synthase/ transferase, transferring acyl groups, acyl groups converted into alkyl on transfer" -0.204016045 0.705222077 0.37269114 0.633128056 0.345097591 -0.14910654 1.513011773 -0.401246663

At3g59380 ATFTA_PFT/PGGT-IALPHA_PLP__FTA (FARNESYLTRANSFERASE A); farnesyltranstransferase/ protein heterodimerization/ protein prenyltransferase 0.453987509 0.301091892 0.148076313 -0.288675019 0.291089129 0.105529956 -0.075569994 0.109926854

At3g59760 ATCS-C__OASC (O-ACETYLSERINE (THIOL) LYASE ISOFORM C); ATP binding / cysteine synthase -0.020308813 0.828765739 0.884782637 0.699781763 0.058182212 0.395304253 -0.784565766 1.705831035

At3g59480 pfkB-type carbohydrate kinase family protein 0.477528603 0.553670226 0.59679987 -0.858264069 0.309783101 -0.896676756 0.829757834 -0.363815281

At3g59030 ATTT12__TT12 (TRANSPARENT TESTA 12); antiporter/ solute:hydrogen antiporter/ transmembrane transporter/ transporter 0.013548538 0.09721318 0.510180806 0.148880693 0.122545912 -0.27892 0.496589942 -0.431362669

At3g59050 ATPAO3 (Polyamine oxidase 3); polyamine oxidase -0.211495307 -0.055494516 -0.30491141 0.881930684 -0.444325223 -0.039474899 0.46940887 0.638414873

At3g59010 pectinesterase family protein -1.582213572 -0.930771629 -0.179346511 -0.25647608 1.051102334 0.926801681 0.53750433 -2.24445355

At3g58740 "CSY1 (CITRATE SYNTHASE 1); citrate (SI)-synthase/ transferase, transferring acyl groups, acyl groups converted into alkyl on transfer" -0.259642504 0.136111591 0.083767324 -0.21379886 -0.128583593 0.382581042 -0.277373706 -2.997662257

At3g58750 CSY2 (citrate synthase 2); citrate (SI)-synthase 0.510620893 0.429204896 -1.119917096 -0.219785427 1.637801078 -0.232206357 0.97362705 0.129713598

At3g58790 "GAUT15 (Galacturonosyltransferase 15); polygalacturonate 4-alpha-galacturonosyltransferase/ transferase, transferring glycosyl groups / transferase, transferring hexosyl groups" 0.096492386 -0.281571226 0.445027111 0.262871153 -0.162972851 -1.038765136 -0.421402741 -1.447368352

At3g58140 phenylalanyl-tRNA synthetase class IIc family protein -0.705094175 -0.886673627 0.705473504 0.072961817 0.477363972 -0.270470137 -0.142545413 0.34791987

At3g58180 PBS lyase HEAT-like repeat-containing protein 0.157007878 0.277696618 0.036281861 0.443443762 -0.081729687 0.055644245 -0.633281149 0.33692979

At3g57650 LPAT2; 1-acylglycerol-3-phosphate O-acyltransferase 0.766355102 0.393979923 0.146500002 -0.391340831 0.203435227 0.515565691 -0.231860242 -0.047692368

At3g57610 ADSS (ADENYLOSUCCINATE SYNTHASE); adenylosuccinate synthase 0.125195087 -0.141494102 0.851140985 0.279535634 -0.357114726 0.608103624 -0.936904255 0.548489112

At3g57785 unknown protein -0.371855746 0.264188668 0.210573995 0.195712184 0.621872944 0.691276179 0.273731034 0.154376751

At3g57260 "BG2_PR-2_PR2__BGL2 (BETA-1,3-GLUCANASE 2); cellulase/ glucan 1,3-beta-glucosidase/ hydrolase, hydrolyzing O-glycosyl compounds" -2.729101125 -1.662708818 -3.397619106 3.956791823 -0.325651747 0.391631989 -0.772447141 1.616596645

At3g57510 ADPG1; polygalacturonase 0.038346764 0.066422254 -0.813620025 -0.276130443 -0.129807286 0.548304791 1.786558989 -0.761872196

At3g57560 aspartate/glutamate/uridylate kinase family protein 0.306783072 -0.125412697 1.270282291 0.37978476 0.255376703 -0.099585718 -0.674066589 -0.28713464

At3g57520 "AtSIP2 (Arabidopsis thaliana seed imbibition 2); hydrolase, hydrolyzing O-glycosyl compounds" 1.586626806 -0.064513971 -0.797062732 0.938350111 -0.399604662 -1.731648753 -1.091970747 1.317894188

At3g57550 GK-2__AGK2 (GUANYLATE KINASE); guanylate kinase 1.031864652 0.69725085 0.121540675 1.413286289 -1.224153415 -1.948891366 0.135135018 0.490034406

At3g56940 ACSF_CHL27__CRD1 (COPPER RESPONSE DEFECT 1); DNA binding / magnesium-protoporphyrin IX monomethyl ester (oxidative) cyclase 0.375464537 -3.261966706 0.368347193 -1.179738804 -0.807659847 -0.240102865 -1.174925219 0.127100187

At3g57050 CBL (cystathionine beta-lyase); cystathionine beta-lyase -0.313444313 0.006827821 0.234990571 -0.367980311 0.583441323 0.682948637 -0.962398873 0.283031806

At3g57240 "BG3 (BETA-1,3-GLUCANASE 3); cellulase/ hydrolase, hydrolyzing O-glycosyl compounds" -1.464912285 -0.147749771 -0.567130696 1.106434091 1.432436957 0.905098774 -0.423013353 -0.948899529

At3g55870 "anthranilate synthase, alpha subunit, putative" 0.248131269 0.095406996 -0.522990428 -0.504247465 0.333950418 -0.482078422 2.27370959 0.254143706

At3g56300 tRNA synthetase class I (C) family protein 0.569079345 0.094802868 0.730924662 0.448883905 -0.55120044 0.024138087 -0.032313829 -0.292511786

At3g56310 "alpha-galactosidase, putative / melibiase, putative / alpha-D-galactoside galactohydrolase, putative" 0.256781433 0.066867567 -1.830255878 -1.221132312 0.396275908 0.533685085 -0.938223104 0.054196346

At3g56350 "superoxide dismutase (Mn), putative / manganese superoxide dismutase, putative" -0.014215941 0.432862306 -0.329824598 0.159927041 0.405440493 -0.32892442 -0.572822558 -5.083810085

At3g55710 UDP-glucoronosyl/UDP-glucosyl transferase family protein 1.221131521 -1.110670584 0.528211802 -0.213529959 -0.671530157 0.672878881 0.86024274 0.270873992

At3g55630 ATDFD (A. THALIANA DHFS-FPGS HOMOLOG D); tetrahydrofolylpolyglutamate synthase -1.546883563 -2.036531128 0.321967054 0.988629233 1.195579855 0.279696536 1.741062829 0.807806713

At3g55700 UDP-glucoronosyl/UDP-glucosyl transferase family protein 0.397622055 0.226308275 -0.212673258 0.334977528 0.128526268 0.099985793 0.408683817 0.080688868

At3g55800 SBPASE (sedoheptulose-bisphosphatase); phosphoric ester hydrolase/ sedoheptulose-bisphosphatase -0.401589696 -3.69772779 0.377263374 0.574550908 -0.844015029 0.385006171 -0.112300493 -0.512891093

At3g55940 "phosphoinositide-specific phospholipase C, putative" -0.611605259 0.822295263 0.539602275 0.735302761 -0.23248652 -0.639939578 1.038196637 -0.239502222

At3g55970 "oxidoreductase, 2OG-Fe(II) oxygenase family protein" 1.025750806 -0.441904494 -2.631976127 1.585120015 -0.375837126 -2.045115718 0.566798618 1.004496355

At3g55610 P5CS2 (DELTA 1-PYRROLINE-5-CARBOXYLATE SYNTHASE 2); catalytic/ glutamate 5-kinase/ oxidoreductase -0.086680304 -0.49769997 -1.295314846 -0.457491935 -0.338495729 0.05748284 0.451081162 -0.264531772

At3g55650 "pyruvate kinase, putative" 0.100549693 0.145278201 0.172180953 0.163227887 0.264450506 -0.327419795 0.009705662 -0.304676785

At3g55590 "GDP-mannose pyrophosphorylase, putative" -0.123960786 0.397173648 0.557704896 0.569583347 0.189472633 -0.382989886 0.872356856 -0.429319859

At3g55360 ATTSC13_ECR_TSC13__CER10; 3-oxo-5-alpha-steroid 4-dehydrogenase/ fatty acid elongase/ trans-2-enoyl-CoA reductase (NADPH) 0.762231857 -0.272383141 0.798434623 -1.243604183 0.045300581 0.932652391 -0.272177234 0.388610872

At3g55440 ATCTIMC__TPI (TRIOSEPHOSPHATE ISOMERASE); triose-phosphate isomerase -0.254396403 0.209388575 0.050203921 -0.170798316 0.067763171 0.576000516 -0.33844494 0.759672898

At3g55400 OVA1 (OVULE ABORTION 1); ATP binding / aminoacyl-tRNA ligase/ methionine-tRNA ligase/ nucleotide binding 0.011841896 -0.138594189 1.735043397 1.85296132 -0.02913527 -0.027351054 0.640605063 0.80616824

At3g55030 PGPS2 (phosphatidylglycerolphosphate synthase 2); CDP-alcohol phosphatidyltransferase/ CDP-diacylglycerol-glycerol-3-phosphate 3-phosphatidyltransferase 0.205655538 0.345137312 0.501654157 0.216130693 0.274422092 0.178541182 0.322542212 -0.020824056

At3g55120 A11_CFI__TT5 (TRANSPARENT TESTA 5); chalcone isomerase 1.137331556 0.103849183 -0.239289331 0.059085726 -2.855114713 3.268455787 -0.951846406 0.085176753

At3g55010 ATPURM__PUR5; ATP binding / phosphoribosylformylglycinamidine cyclo-ligase 0.580406562 0.566904612 1.462477815 0.90235407 -0.339584189 1.066490014 -1.080917198 0.776249186

At3g54640 TRP3__TSA1 (TRYPTOPHAN SYNTHASE ALPHA CHAIN); tryptophan synthase 0.483402215 0.833907422 -0.683758187 1.688694979 -0.163170012 -0.550688148 -1.710735511 2.013669719

At3g54660 ATGR2_EMB2360__GR (GLUTATHIONE REDUCTASE); ATP binding / glutathione-disulfide reductase -0.687513547 -0.790227971 0.293204309 0.119431311 -0.884847216 -0.18665621 -0.376180217 -0.836910501

At3g54270 sucrose-phosphatase 3 (SPP3) 0.457996234 0.469948192 0.336675172 0.041222101 -0.07440792 -0.042409355 -0.218718468 -0.641507936

At3g54250 "mevalonate diphosphate decarboxylase, putative" 1.375485204 0.577261246 1.018310979 -0.484999232 -0.082463682 0.88841739 0.432022953 0.534273769

At3g54050 "fructose-1,6-bisphosphatase, putative / D-fructose-1,6-bisphosphate 1-phosphohydrolase, putative / FBPase, putative" -1.335530588 -4.002511122 0.090651899 -0.095336872 0.010826344 0.162261915 0.384311423 0.390264187

At3g53900 "uracil phosphoribosyltransferase, putative / UMP pyrophosphorylase, putative / UPRTase, putative" 0.239645692 -0.288408883 2.383223491 0.539154427 0.326612518 0.205398808 -0.456832357 0.092629266

At3g54090 pfkB-type carbohydrate kinase family protein 0.560899786 -1.116191239 1.553097522 1.090820578 0.878978923 0.405393147 -1.096405764 -0.12098805

At3g53520 ATUXS1__UXS1 (UDP-GLUCURONIC ACID DECARBOXYLASE 1); UDP-glucuronate decarboxylase/ catalytic 0.558941777 0.37983168 -0.018613435 -0.377223673 -0.005664516 0.37332449 0.213045793 0.017008009

At3g53580 diaminopimelate epimerase family protein 0.321130983 0.325115492 0.99134784 0.399336417 0.023285784 0.072972281 -0.42503663 -0.039285023

At3g53620 AtPPa4 (Arabidopsis thaliana pyrophosphorylase 4); inorganic diphosphatase 0.536117852 0.201106625 0.569454617 -0.496462667 -1.333461847 -1.04577459 -0.587680659 -0.331852537

At3g53130 CYP97C1__LUT1 (LUTEIN DEFICIENT 1); epsilon hydroxylase/ oxygen binding 0.074556663 -1.617881612 0.963423455 0.57711648 0.244459231 0.017122535 -0.109554184 -0.28787276

At3g53150 "UGT73D1 (UDP-glucosyl transferase 73D1); UDP-glycosyltransferase/ transferase, transferring hexosyl groups" 0.131617078 0.901953066 -1.325264247 1.106488455 0.31213219 -0.527465437 -0.727882802 0.627457933

At3g53160 "UGT73C7 (UDP-glucosyl transferase 73C7); UDP-glycosyltransferase/ transferase, transferring glycosyl groups" 0.757985834 0.148948281 -0.61779988 1.113992437 0.128752874 -0.90179809 -0.377013063 -0.190954415

At3g53260 PAL2; phenylalanine ammonia-lyase 0.396592488 -0.104379292 0.166436864 -0.986381187 -3.608098479 -1.086225208 -1.4208186 0.445192543

At3g52780 ATPAP20__PAP20; acid phosphatase/ protein serine/threonine phosphatase 0.364831329 -0.080097146 -1.709351331 -0.6094357 -0.430794128 0.490027886 0.668259398 -2.837872106

At3g52820 ATPAP22__PAP22 (PURPLE ACID PHOSPHATASE 22); acid phosphatase/ protein serine/threonine phosphatase 0.709265259 0.19929622 -0.586677486 -0.289134739 -0.074637213 -0.208564356 0.510126469 -0.121254167

At3g52720 ATACA1__ACA1 (ALPHA CARBONIC ANHYDRASE 1); carbonate dehydratase/ zinc ion binding -3.785978869 -1.435221063 1.402316304 0.298125077 -2.279561469 -0.030102467 1.903803953 -0.345307604

At3g52930 "fructose-bisphosphate aldolase, putative" 0.526240941 0.807155537 0.25232585 -0.29695758 -0.428045792 0.74752827 -0.583782444 1.135356285

At3g52880 "ATMDAR1__monodehydroascorbate reductase, putative" -0.143423525 -0.31879206 -0.6688942 -0.270168858 -0.400961597 0.614049332 0.171926926 1.183045761

At3g51520 diacylglycerol acyltransferase family -0.578959868 0.125904695 -0.228481006 -1.510979712 0.620982209 0.576340431 -0.28570878 0.115407394

At3g51160 "GMD2_MUR_1__MUR1 (MURUS 1); GDP-mannose 4,6-dehydratase" -0.057590337 0.913614673 0.521328872 0.332803839 -0.88434919 -0.659252936 -0.10581802 0.830769941

At3g51240 F3'H_TT6__F3H (FLAVANONE 3-HYDROXYLASE); naringenin 3-dioxygenase 1.753164289 -0.294960637 -0.418486751 0.508159774 -5.890576799 4.514951475 -0.933575521 -0.392373879

At3g50820 OEC33_PSBO-2__PSBO2 (PHOTOSYSTEM II SUBUNIT O-2); oxygen evolving/ poly(U) binding -1.379362625 -3.275536878 0.1890418 -1.033765452 -0.556306596 -0.372324491 -0.247849344 -0.023752976

At3g50790 "late embryogenesis abundant protein, putative / LEA protein, putative" -0.120267625 -1.019810651 0.87804768 0.120989532 -0.315005392 -0.434252163 0.37307433 -0.431078114

At3g50740 "UGT72E1 (UDP-glucosyl transferase 72E1); UDP-glycosyltransferase/ coniferyl-alcohol glucosyltransferase/ transferase, transferring glycosyl groups" -2.887084974 1.594803332 -0.275063196 -0.832908974 2.03671288 -1.474049694 -2.286561484 -0.459702782

At3g50660 CLM_CYP90B1_SNP2__DWF4 (DWARF 4); steroid 22-alpha hydroxylase -0.144343461 0.383079595 0.28275097 -0.412101276 1.010230572 0.184872136 0.376555014 0.121835778

At3g50210 "2-oxoacid-dependent oxidase, putative" 0.37970064 0.441614966 -1.434635732 -0.988165154 2.742000131 0.824411642 -0.561778497 0.561052184

At3g49220 pectinesterase family protein 0.815061435 -1.299564938 0.550131342 -0.442359551 -0.455117582 -0.973379436 0.343890431 2.318079038

At3g49620 DIN11 (DARK INDUCIBLE 11); iron ion binding / oxidoreductase 0.750719567 0.450256111 -1.274267522 3.245974236 0.900167832 -4.506097717 -2.361346163 1.260648077

At3g49630 "2-oxoacid-dependent oxidase, putative" 0.017019768 0.415906031 0.27443198 0.110605445 0.485828662 -0.367362194 0.231466056 -0.143047818

At3g49680 ATBCAT-3__BCAT3 (BRANCHED-CHAIN AMINOTRANSFERASE 3); branched-chain-amino-acid transaminase/ catalytic -0.349074957 -0.166963268 1.289971805 0.215380267 -0.063317278 0.855482089 -0.834890786 0.473429387

At3g49700 ETO3__ACS9 (1-AMINOCYCLOPROPANE-1-CARBOXYLATE SYNTHASE 9); 1-aminocyclopropane-1-carboxylate synthase -0.070788567 0.375688287 0.472097238 0.427608449 0.103745956 -0.617670781 0.163318756 -0.515117419

At3g48720 transferase family protein -3.472178308 -1.486707341 1.368902614 0.100956804 1.274006471 -0.627832853 0.122780721 -0.291391097

At3g48730 "GSA2 (glutamate-1-semialdehyde 2,1-aminomutase 2); catalytic/ glutamate-1-semialdehyde 2,1-aminomutase/ pyridoxal phosphate binding / transaminase" -0.005274538 -1.749404837 1.640935549 0.412292387 0.715642858 0.151147325 -0.648935175 0.353725111

At3g48560 AHAS_ALS_IMR1_TZP5__CSR1 (CHLORSULFURON/IMIDAZOLINONE RESISTANT 1); acetolactate synthase/ pyruvate decarboxylase -0.198319568 -0.264628633 0.94375116 0.34343912 0.112038912 -0.426319906 0.106224106 -0.456810349

At3g48680 GAMMA CAL2 (GAMMA CARBONIC ANHYDRASE-LIKE 2); transferase 0.241167603 0.47862449 -0.14571673 -0.672590951 0.776214387 0.001917347 -0.389397647 0.118348858

At3g48790 "serine C-palmitoyltransferase, putative" 0.55105023 0.848169803 -0.56990545 0.705890684 -0.115830588 -1.564809865 1.157192802 0.007234288

At3g48610 phosphoesterase family protein -0.056259043 -0.798649188 1.207415998 -0.033188637 -0.946668535 0.52414274 -0.720898705 0.234460335

At3g48170 ALDH10A9; 3-chloroallyl aldehyde dehydrogenase/ oxidoreductase 0.026891377 0.372610035 -0.757492492 -0.423921146 1.080352129 0.547248202 -0.195688551 0.538651434

At3g48420 haloacid dehalogenase-like hydrolase family protein -1.228367783 -3.046746647 0.969294465 0.78078289 0.205390874 -0.224861567 0.432699174 0.41862796

At3g48000 ALDH2A__ALDH2B4 (ALDEHYDE DEHYDROGENASE 2B4); 3-chloroallyl aldehyde dehydrogenase/ ATP binding / aldehyde dehydrogenase (NAD) -0.149858072 -0.519209846 -1.511590503 -0.859920361 0.29732119 0.211541196 -1.574352854 -0.38582178

At3g47930 "ATGLDH (L-GALACTONO-1,4-LACTONE DEHYDOROGENASE); L-gulono-1,4-lactone dehydrogenase/ galactonolactone dehydrogenase" 0.109393843 0.376516788 0.721013729 0.654002089 0.407096925 0.232461316 -0.257952323 -0.631230661

At3g47833 unknown protein -0.378680723 0.537110115 -0.407760535 0.017248198 0.315518955 0.97896645 0.153998237 0.806404466

At3g47520 "MDH (MALATE DEHYDROGENASE); L-malate dehydrogenase/ binding / catalytic/ malate dehydrogenase/ oxidoreductase/ oxidoreductase, acting on the CH-OH group of donors, NAD or NADP as acceptor" 0.01513637 0.382387384 0.622242006 0.365502855 -0.399287024 0.583600161 -0.797751276 -0.201790515

At3g47340 AT-ASN1_DIN6__ASN1 (GLUTAMINE-DEPENDENT ASPARAGINE SYNTHASE 1); asparagine synthase (glutamine-hydrolyzing) 3.405286174 -1.26940562 0.220191198 -3.304871563 0.080965914 -8.753991619 0.532057479 -0.27109148

At3g47400 pectinesterase family protein -0.316744216 0.777133297 0.623982033 -0.335474225 0.360955025 -0.133874422 0.374266279 0.195052282

At3g46940 deoxyuridine 5'-triphosphate nucleotidohydrolase family 0.847976098 0.86577735 2.423878545 1.130414827 -0.232883738 1.182905884 -0.873011073 0.710575032

At3g47220 phosphoinositide-specific phospholipase C family protein -0.000118669 -0.287006358 0.727927604 -0.094307478 -0.068899572 0.014835922 0.558377668 0.942746857

At3g46970 "ATPHS2__PHS2 (ALPHA-GLUCAN PHOSPHORYLASE 2); phosphorylase/ transferase, transferring glycosyl groups" -0.202428281 -1.172533159 -0.374783742 1.270328096 5.359174738 1.718907259 0.161679391 0.85906921

At3g46680 UDP-glucoronosyl/UDP-glucosyl transferase family protein 0.072464038 0.285827562 0.092297806 0.475671722 0.172159962 -0.395038651 0.259675688 -0.530901776

At3g46670 "UGT76E11 (UDP-GLUCOSYL TRANSFERASE 76E11); UDP-glycosyltransferase/ quercetin 3-O-glucosyltransferase/ quercetin 7-O-glucosyltransferase/ transferase, transferring glycosyl groups" -0.07119126 -0.850904094 -1.382769177 2.034952938 0.580165666 1.094018553 0.274839855 -1.178683138

At3g46690 UDP-glucoronosyl/UDP-glucosyl transferase family protein 0.057057515 0.732620416 -0.278542671 -0.149076473 0.04150843 -0.906101526 -0.514488181 0.281176398

At3g46660 "UGT76E12 (UDP-GLUCOSYL TRANSFERASE 76E12); UDP-glycosyltransferase/ quercetin 3-O-glucosyltransferase/ quercetin 7-O-glucosyltransferase/ transferase, transferring glycosyl groups" 1.077033998 0.340562818 -2.640508016 2.182605584 -0.585868137 -0.82383837 0.415584374 -0.866768044

At3g46700 "UDP-glycosyltransferase/ transferase, transferring glycosyl groups" -0.424115696 1.479231083 -0.047414849 -0.741815386 -0.491682908 -0.666268686 -0.409486823 0.480205732

At3g46720 UDP-glucoronosyl/UDP-glucosyl transferase family protein -0.60856736 1.221726058 0.320814133 -0.519244471 -0.012143312 0.169262454 -0.036069894 0.311807276

At3g46100 ATHRS1 (HISTIDYL-TRNA SYNTHETASE 1); histidine-tRNA ligase 0.079454538 -0.74566134 0.777349016 0.440283195 -0.15881084 -0.229373017 -0.342314619 0.515292271

At3g46480 "iron ion binding / oxidoreductase/ oxidoreductase, acting on paired donors, with incorporation or reduction of molecular oxygen, 2-oxoglutarate as one donor, and incorporation of one atom each of oxygen into both donors" -0.088124064 0.196181866 0.196688139 0.250086178 0.23243453 -0.414271194 0.260406971 -0.578602846

At3g46440 UXS5; UDP-glucuronate decarboxylase/ catalytic 0.155231781 1.257087957 -1.049037264 -0.269215871 2.652539992 1.323491315 0.732744612 0.630291736

At3g46490 "iron ion binding / oxidoreductase/ oxidoreductase, acting on paired donors, with incorporation or reduction of molecular oxygen, 2-oxoglutarate as one donor, and incorporation of one atom each of oxygen into both donors" -2.011303823 -0.074640755 1.679533922 0.107428218 0.528761324 -0.583896833 0.125051159 -0.396448115

At3g46500 "oxidoreductase, 2OG-Fe(II) oxygenase family protein" -0.176488976 0.905989305 0.132150907 -0.383728016 0.268275011 -0.456825955 -0.210387313 0.11469005

At3g45940 "alpha-xylosidase, putative" 0.771841223 -0.207212819 -0.005787577 -0.124518802 0.16054554 -0.1045023 0.482736279 0.134299927

At3g45300 ATIVD__IVD (ISOVALERYL-COA-DEHYDROGENASE); ATP binding / isovaleryl-CoA dehydrogenase 0.822651976 -0.152375222 -2.46497929 -2.468989359 1.390568533 -1.60505809 -0.068407613 0.801142589

At3g45070 sulfotransferase family protein -1.347088105 2.423306369 0.564426849 -2.101074758 -0.50873431 0.810994665 -0.455672738 1.19598884

At3g45130 LAS1; lanosterol synthase -0.400289259 0.230980526 -0.228773198 -0.282358258 -0.076423168 -0.116912869 0.199774963 -0.79451307

At3g45140 ATLOX2__LOX2 (LIPOXYGENASE 2); lipoxygenase 0.788714192 -3.289197452 -1.143730333 4.446649415 1.941786925 1.176226433 1.734246182 2.447413035

At3g44540 "FAR4 (FATTY ACID REDUCTASE 4); binding / catalytic/ oxidoreductase, acting on the CH-CH group of donors" -0.638254077 1.901262852 -0.695285651 -1.787942494 -0.526740705 -0.081330822 -0.625069197 -1.417022505

At3g44550 "FAR5 (FATTY ACID REDUCTASE 5); binding / catalytic/ oxidoreductase, acting on the CH-CH group of donors" -0.218828266 1.447957895 -0.507284319 -0.556231173 -0.662451413 -0.591072985 -0.321646351 -0.075020223

At3g44560 "FAR8 (FATTY ACID REDUCTASE 8); fatty acyl-CoA reductase (alcohol-forming)/ oxidoreductase, acting on the CH-CH group of donors" 0.174288433 0.258180826 0.188187274 0.203346832 0.110400463 -0.288407909 0.187181561 -0.315391279

At3g44720 ADT4 (arogenate dehydratase 4); arogenate dehydratase/ prephenate dehydratase 0.669615695 0.374975678 -0.690011683 1.607672001 -2.712506906 -1.169363075 -1.878380925 0.576469715

At3g44740 ATP binding / aminoacyl-tRNA ligase/ glycine-tRNA ligase/ nucleotide binding 0.432264272 0.306684512 0.778881219 0.427145735 0.036198347 0.243538991 -0.18933513 -0.293438182

At3g44320 NIT3 (NITRILASE 3); indole-3-acetonitrile nitrilase/ indole-3-acetonitrile nitrile hydratase/ nitrilase -0.987754281 1.762240439 -0.632336482 0.20861692 -0.696973968 -0.166872729 -0.684257946 0.472203729

At3g44300 NIT2 (nitrilase 2); indole-3-acetonitrile nitrilase/ indole-3-acetonitrile nitrile hydratase/ nitrilase -0.190581653 -1.192779511 -1.704694909 -0.567391573 -0.678017576 0.623401731 -1.114549137 0.796506678

At3g43860 "AtGH9A4 (Arabidopsis thaliana Glycosyl Hydrolase 9A4); catalytic/ hydrolase, hydrolyzing O-glycosyl compounds" -0.220163045 0.620829585 -0.042417896 0.191455662 0.267791308 -0.207130208 5.290945647 -0.25807251

At3g43270 pectinesterase family protein -0.572208824 -0.350551986 -1.675266294 -0.590225453 0.757984438 0.254101682 1.114979163 0.088008509

At3g43190 "ATSUS4__SUS4; UDP-glycosyltransferase/ sucrose synthase/ transferase, transferring glycosyl groups" -0.448802139 1.111393395 0.219819464 -0.626149117 -0.110512863 -0.083392727 -0.270911554 -0.281132855

At3g42850 "galactokinase, putative" 0.344382414 0.290206352 0.53399498 0.36019022 0.020035838 -0.010015973 2.009943275 -0.601167492

At4g39955 "hydrolase, alpha/beta fold family protein" 0.21121379 0.473880106 -1.839140798 -0.560247558 -0.390529227 0.259359372 -0.720254453 0.677157985

At4g39950 CYP79B2; electron carrier/ heme binding / iron ion binding / monooxygenase/ oxygen binding -1.206496354 1.463848389 -0.901834211 1.703138591 -0.630925783 -0.911041897 -1.958126841 2.711545209

At4g39980 DHS1 (3-DEOXY-D-ARABINO-HEPTULOSONATE 7-PHOSPHATE SYNTHASE 1); 3-deoxy-7-phosphoheptulonate synthase 0.636285498 0.66278279 0.593278132 1.166722059 0.034548889 -0.482756698 -1.541880171 0.002884365

At4g39770 "trehalose-6-phosphate phosphatase, putative" -1.927978506 0.327649556 1.450257979 -0.995254235 -0.37748949 -0.791264302 0.406382753 0.042007901

At4g39830 "L-ascorbate oxidase, putative" -0.254635296 0.178668971 -0.839646873 2.619380713 0.029814943 -1.508392571 -0.466871649 0.415943006

At4g39800 ATMIPS1_MI-1-P SYNTHASE__MIPS1 (MYO-INOSITOL-1-PHOSTPATE SYNTHASE 1); inositol-3-phosphate synthase -0.932667965 -0.871498005 0.424241459 0.65027167 -1.607500686 1.624178641 0.526872221 -1.988644267

At4g39940 "AKN2 (APS-kinase 2); ATP binding / adenylylsulfate kinase/ kinase/ transferase, transferring phosphorus-containing groups" -1.869990218 0.286312597 0.168913966 0.626467276 -0.433551627 -0.497585778 -1.243554158 2.556000627

At4g39970 haloacid dehalogenase-like hydrolase family protein -1.073196745 -2.025536133 1.102185841 0.381188161 0.454176687 -0.591164485 0.721945989 0.564932223

At4g39350 "ATH-A__CESA2 (CELLULOSE SYNTHASE A2); cellulose synthase/ transferase, transferring glycosyl groups" -1.056596645 0.078669576 1.216873885 -1.27177792 -0.471177147 -0.543306245 -0.589590409 -0.758726482

At4g39210 APL3; glucose-1-phosphate adenylyltransferase -0.174305888 -0.636804997 -1.663245847 1.54652997 -0.275307748 2.782945816 1.341596503 0.74978571

At4g39490 CYP96A10; electron carrier/ heme binding / iron ion binding / monooxygenase 0.282160436 0.019163085 -0.001372754 -0.037921515 0.11943904 -0.268934965 0.657511812 -0.419060735

At4g39540 shikimate kinase family protein -0.151196008 -0.343285984 -0.146395559 -0.061668649 -0.921800247 0.279679502 -0.870305232 0.590640011

At4g39640 GGT1 (GAMMA-GLUTAMYL TRANSPEPTIDASE 1); gamma-glutamyltransferase/ glutathione gamma-glutamylcysteinyltransferase -1.340920527 -0.556505594 0.049919191 1.175311513 -0.473461489 -3.336704454 -0.347302549 -0.320732356

At4g39650 GGT2 (GAMMA-GLUTAMYL TRANSPEPTIDASE 2); gamma-glutamyltransferase/ glutathione gamma-glutamylcysteinyltransferase -0.114567759 0.208092317 0.258114551 0.227215603 0.234087054 -0.303406552 0.48371487 -0.820203901

At4g39000 "AtGH9B17 (Arabidopsis thaliana glycosyl hydrolase 9B17); catalytic/ hydrolase, hydrolyzing O-glycosyl compounds" 0.055998021 0.291476463 0.43870517 0.322296584 0.164611041 -0.312842806 1.023046076 -0.358983808

At4g38970 "fructose-bisphosphate aldolase, putative" -0.059705474 -3.695075478 0.16156914 0.169485205 -1.172972999 0.503296148 -0.628297635 0.5882068

At4g39010 "AtGH9B18 (Arabidopsis thaliana glycosyl hydrolase 9B18); catalytic/ hydrolase, hydrolyzing O-glycosyl compounds" 1.080565869 -0.204358903 0.294934065 -0.15260187 -0.322465609 0.354468348 0.819938425 -0.351720413

At4g39120 "IMPL2 (MYO-INOSITOL MONOPHOSPHATASE LIKE 2); 3'(2'),5'-bisphosphate nucleotidase/ L-galactose-1-phosphate phosphatase/ inositol or phosphatidylinositol phosphatase/ inositol-1(or 4)-monophosphatase" -0.093811711 0.287575636 1.195935036 0.309709283 0.837437412 0.619141223 -0.195782936 0.634434628

At4g39230 "isoflavone reductase, putative" 1.731951785 1.10032334 0.446498254 1.978016968 -0.928094109 -0.767297715 0.251968604 0.548477417

At4g39280 "phenylalanyl-tRNA synthetase, putative / phenylalanine--tRNA ligase, putative" 0.274561787 0.212847638 0.4695561 0.328032212 -0.081791186 0.742894137 -0.719395234 0.090402473

At4g39330 CAD9 (CINNAMYL ALCOHOL DEHYDROGENASE 9); binding / catalytic/ oxidoreductase/ zinc ion binding 2.647587912 -2.28725309 1.342652504 -0.231536821 -0.439713257 1.598519572 0.38209296 0.91887343

At4g38620 ATMYB4__MYB4; DNA binding / transcription factor 0.681418144 0.289930068 -0.322251838 -0.382703016 -1.459098208 1.448307581 -0.387842351 0.394835293

At4g38880 ATASE3 (GLN PHOSPHORIBOSYL PYROPHOSPHATE AMIDOTRANSFERASE 3); amidophosphoribosyltransferase -0.326222475 0.22048378 0.578479303 0.438311796 0.146450015 -0.455174919 0.387092145 -0.593068186

At4g38800 ATMTN1; catalytic/ methylthioadenosine nucleosidase -0.176959639 0.448084301 -0.131233406 -0.676273082 0.503255648 -0.02936472 0.065653149 0.778275354

At4g37980 ELI3-1 (ELICITOR-ACTIVATED GENE 3-1); binding / catalytic/ oxidoreductase/ zinc ion binding -0.153494473 -2.146249592 -1.451826327 1.640005781 0.319655798 0.35905841 1.971400691 1.038441284

At4g37990 ATCAD8_CAD-B2__ELI3-2 (ELICITOR-ACTIVATED GENE 3-2); aryl-alcohol dehydrogenase/ mannitol dehydrogenase 0.351154325 0.128072414 -2.681920095 1.885487248 0.451237897 0.127658468 2.102606683 0.845475904

At4g38420 sks9 (SKU5 Similar 9); copper ion binding / oxidoreductase -1.440815125 -0.841928582 1.512666387 1.132574065 -1.346989113 -3.015272022 0.129331996 -0.20596165

At4g38530 ATPLC1 (ARABIDOPSIS THALIANA PHOSPHOLIPASE C 1); phospholipase C -0.639084771 0.504459008 0.26802332 0.154776053 0.26621077 -0.545152129 0.225581105 -0.16635299

At4g37930 SHMT1_STM__SHM1 (SERINE TRANSHYDROXYMETHYLTRANSFERASE 1); glycine hydroxymethyltransferase/ poly(U) binding -0.956620816 -2.514050075 0.099530041 -0.006026308 -0.438200135 0.441923119 0.860994926 2.268921186

At4g37970 CAD6 (CINNAMYL ALCOHOL DEHYDROGENASE 6); binding / catalytic/ oxidoreductase/ zinc ion binding -0.795756842 0.729589238 -0.101358542 0.135030341 0.282096627 0.023348736 -0.093348658 0.152442844

At4g38190 "CSLD4__ATCSLD4; cellulose synthase/ transferase, transferring glycosyl groups" -0.455648772 0.693798635 0.422564331 0.663521627 0.458774682 -0.547657491 3.03807003 -0.16341391

At4g38270 "GAUT3 (Galacturonosyltransferase 3); polygalacturonate 4-alpha-galacturonosyltransferase/ transferase, transferring glycosyl groups" 0.470761004 0.30459504 -0.43835845 -0.185601561 0.570289501 0.242960822 -0.085375204 0.196124822

At4g37760 SQE3 (squalene epoxidase 3); squalene monooxygenase -0.438039213 -1.280325427 -0.582510881 -0.466030012 -2.222035417 0.270503882 1.521530035 -1.067443014

At4g37870 PEPCK__PCK1 (PHOSPHOENOLPYRUVATE CARBOXYKINASE 1); ATP binding / phosphoenolpyruvate carboxykinase (ATP)/ phosphoenolpyruvate carboxykinase/ purine nucleotide binding 1.877262364 0.294119381 -0.234682644 -0.944516374 1.148631335 -0.287063009 -0.637826767 0.036929976

At4g37670 GCN5-related N-acetyltransferase (GNAT) family protein / amino acid kinase family protein 0.157458648 -0.058694168 0.239977467 1.355057444 0.542542252 -0.677644197 -0.713222655 0.224069867

At4g37770 ACS8; 1-aminocyclopropane-1-carboxylate synthase 0.062920883 -0.140913424 0.596373926 1.062240075 -0.301777995 -2.555930169 0.164175073 -0.446574521

At4g37840 HKL3 (HEXOKINASE-LIKE 3); ATP binding / fructokinase/ glucokinase/ hexokinase -0.122190596 0.547846864 0.593927723 0.738430937 0.340473435 -0.31363688 1.50157121 -0.2425708

At4g37830 cytochrome c oxidase-related 0.026733243 0.4035831 -0.137428571 -0.528766872 0.063304761 0.100527763 -0.022468151 0.432573459

At4g36250 ALDH3F1 (Aldehyde Dehydrogenase 3F1); 3-chloroallyl aldehyde dehydrogenase/ aldehyde dehydrogenase (NAD) 1.001869187 -1.396790182 0.702523524 -0.1055478 0.284961808 0.452351581 0.774107337 1.165067207

At4g36220 CYP84A1__FAH1 (FERULIC ACID 5-HYDROXYLASE 1); ferulate 5-hydroxylase/ monooxygenase -0.949504371 -0.434345938 -0.356503702 0.314514053 -0.819646064 0.188041374 -2.230005285 -1.723529723

At4g35790 PLDDELTA__ATPLDDELTA; phospholipase D 0.528968991 0.830510244 -0.794935832 0.198326182 2.167081578 -0.078581902 0.284894179 -0.475547066

At4g35830 "aconitate hydratase, cytoplasmic / citrate hydro-lyase / aconitase (ACO)" -0.046646213 0.297359888 -0.31501167 -0.633077675 0.962215645 -0.602457244 -0.367116618 -0.165261968

At4g35650 "isocitrate dehydrogenase, putative / NAD+ isocitrate dehydrogenase, putative" -0.195078238 0.542158695 0.632156629 0.375297165 0.20975482 -0.284576101 1.890417678 -0.582151444

At4g35670 glycoside hydrolase family 28 protein / polygalacturonase (pectinase) family protein 0.027268154 0.799349977 0.747897325 0.759033579 0.227428301 -0.12676691 2.498153791 -0.058461844

At4g35630 PSAT; O-phospho-L-serine:2-oxoglutarate aminotransferase 1.037104986 1.093394323 -0.876952105 1.441327664 0.831079543 0.486852207 -1.606908352 0.993836756

At4g35090 CAT2 (CATALASE 2); catalase -2.140457841 -1.194973395 -0.643310483 -1.047549104 -2.108696067 0.107068703 0.214354976 -0.479179559

At4g35260 "IDH1 (ISOCITRATE DEHYDROGENASE 1); isocitrate dehydrogenase (NAD+)/ oxidoreductase, acting on the CH-OH group of donors, NAD or NADP as acceptor" -0.206228439 0.677856928 -0.471516743 0.486013696 0.226789629 0.803723759 -0.260781228 0.960912388

At4g34710 SPE2__ADC2 (ARGININE DECARBOXYLASE 2); arginine decarboxylase 1.414375907 0.588924821 -0.840872645 2.157912937 -1.050863628 -1.789340034 -0.516152794 0.208741342

At4g34640 ERG9__SQS1 (SQUALENE SYNTHASE 1); farnesyl-diphosphate farnesyltransferase 0.365317482 0.516266693 0.437451821 -0.525414057 -0.14169545 0.351694115 0.148775325 1.267473917

At4g34840 ATMTN2; methylthioadenosine nucleosidase 0.278886799 0.469107585 0.578217756 0.282909435 0.309287285 -0.150103796 0.390247046 0.378726815

At4g34890 ATXDH1__XDH1 (XANTHINE DEHYDROGENASE 1); xanthine dehydrogenase 0.298480597 0.091843885 -1.893378843 -0.447314137 1.572848999 0.068631913 -0.497320452 -0.928440161

At4g34850 chalcone and stilbene synthase family protein 0.989632738 -0.155777506 0.353029885 0.179080893 -0.107976608 -0.069213737 0.341495783 -0.343640833

At4g35000 APX3 (ASCORBATE PEROXIDASE 3); L-ascorbate peroxidase -0.106893746 -0.580333564 -0.043943659 -0.089773435 0.177480266 0.269001248 0.039184876 0.788398818

At4g34860 "beta-fructofuranosidase, putative / invertase, putative / saccharase, putative / beta-fructosidase, putative" 1.717650897 0.64530105 -0.846973441 0.109042138 -0.185453184 0.853912697 -0.280763862 -0.401160313

At4g34350 CLB6_ISPH__HDR (4-HYDROXY-3-METHYLBUT-2-ENYL DIPHOSPHATE REDUCTASE); 4-hydroxy-3-methylbut-2-en-1-yl diphosphate reductase/ 4-hydroxy-3-methylbut-2-en-1-yl diphosphate synthase -0.079105342 -1.631998813 -0.682010565 -1.852022825 -1.94846671 -1.208059426 -0.617760825 -0.040117222

At4g34510 "KCS17 (3-KETOACYL-COA SYNTHASE 17); acyltransferase/ catalytic/ transferase, transferring acyl groups other than amino-acyl groups" 0.559455268 0.455193012 0.157086761 -0.137713547 0.20767751 0.378609255 1.026216044 -2.060187033

At4g34520 FAE1__KCS18; acyltransferase/ fatty acid elongase -0.148977427 0.014327112 -0.171005536 -0.766922589 -0.3559357 0.581200348 -0.907973033 -5.061128016

At4g34740 ATPURF2_CIA1__ATASE2 (GLN PHOSPHORIBOSYL PYROPHOSPHATE AMIDOTRANSFERASE 2); amidophosphoribosyltransferase -0.164412122 0.002938224 1.221392501 1.122994215 0.018650726 0.246812798 -1.081211178 -1.678721031

At4g34650 SQS2 (SQUALENE SYNTHASE 2); farnesyl-diphosphate farnesyltransferase -0.362441373 -0.281299814 0.273107851 0.452229301 -0.81777249 -0.270575072 0.301945805 -0.154623983

At4g34135 UGT73B2 (UDP-GLUCOSYLTRANSFERASE 73B2); UDP-glucosyltransferase/ UDP-glycosyltransferase/ flavonol 3-O-glucosyltransferase/ quercetin 7-O-glucosyltransferase -2.018919888 1.598432663 -4.230978224 2.088474638 -0.154568518 -0.278243245 1.50801362 3.55242648

At4g34050 "caffeoyl-CoA 3-O-methyltransferase, putative" -0.037266997 0.376827454 -0.729506394 -1.189690475 -1.06922467 0.906430736 -0.962333894 0.40059542

At4g34230 CAD-5_CAD5__ATCAD5 (CINNAMYL ALCOHOL DEHYDROGENASE 5); cinnamyl-alcohol dehydrogenase 0.753930827 0.304834301 -3.166723467 -0.641002731 -1.284461606 0.258781383 -0.417681557 1.515730379

At4g34030 MCCB (3-METHYLCROTONYL-COA CARBOXYLASE); biotin carboxylase/ methylcrotonoyl-CoA carboxylase 0.766387932 -0.559162316 -0.455119555 -1.829150483 1.437954164 -0.828399696 -0.632403296 0.4423663

At4g34138 UGT73B1 (UDP-glucosyl transferase 73B1); UDP-glycosyltransferase/ abscisic acid glucosyltransferase/ quercetin 3-O-glucosyltransferase/ quercetin 7-O-glucosyltransferase -0.675331002 -0.264356665 -1.271625607 -2.898208352 0.524289735 -1.068921998 0.102811108 1.054208029

At4g34250 "KCS16 (3-KETOACYL-COA SYNTHASE 16); acyltransferase/ catalytic/ transferase, transferring acyl groups other than amino-acyl groups" -0.278222153 -0.568153064 1.286320297 -0.757620603 1.028781173 -1.126131477 -0.896030907 -3.808096545

At4g33760 "tRNA synthetase class II (D, K and N) family protein" 0.184245763 -0.275931354 1.172552767 0.879235793 0.001365754 0.055893076 -0.440739835 -0.15809551

At4g33770 "inositol 1,3,4-trisphosphate 5/6-kinase family protein" -0.385394976 0.525093869 0.572736056 -0.098263648 -0.925406139 -0.50590038 0.069115416 0.207911202

At4g33580 ATBCA5_BCA5__carbonic anhydrase family protein / carbonate dehydratase family protein -0.301655422 0.11463744 0.521704948 -1.09620795 -0.412404966 0.227098831 0.547236758 0.02807146

At4g33670 L-galactose dehydrogenase (L-GalDH) 0.28985715 -0.5270538 0.000827091 -0.406425661 2.087921927 -0.173739709 -0.629240387 -1.30551213

At4g33680 "AGD2 (ABERRANT GROWTH AND DEATH 2); L,L-diaminopimelate aminotransferase/ transaminase" 0.300469428 -0.212195463 1.107825597 0.083492532 0.111236763 0.716508182 -0.602645414 0.13762748

At4g33790 "FAR3_G7__CER4 (ECERIFERUM 4); fatty acyl-CoA reductase (alcohol-forming)/ oxidoreductase, acting on the CH-CH group of donors" 1.961767819 -0.849834417 -0.375114873 -2.394412975 -0.518066081 0.680822029 1.149814238 1.394051591

At4g33510 DHS2 (3-deoxy-d-arabino-heptulosonate 7-phosphate synthase); 3-deoxy-7-phosphoheptulonate synthase -0.181478879 -0.785691146 -0.745606504 0.372670854 1.512811367 0.266438038 -0.449440757 -0.277375992

At4g33230 pectinesterase family protein -0.457223038 0.819508569 0.678333421 0.850205969 0.393929003 -0.563596637 2.597091661 -0.528324819

At4g33220 enzyme inhibitor/ pectinesterase 0.790653018 -2.136209552 1.083252347 -1.040131335 -0.631560944 -0.323607988 0.682514173 0.582680123

At4g33150 LKR_LKR/SDH_SDH__lysine-ketoglutarate reductase/saccharopine dehydrogenase bifunctional enzyme 1.277930583 0.036252437 -4.353901077 -2.881234486 0.342148219 -0.135972648 1.404576273 0.125527619

At4g33030 SQD1; UDPsulfoquinovose synthase/ sulfotransferase -0.176764299 -0.876502268 -0.019237083 0.137375825 0.538349092 0.436823695 -0.609621379 0.197777962

At4g33010 AtGLDP1 (Arabidopsis thaliana glycine decarboxylase P-protein 1); catalytic/ glycine dehydrogenase (decarboxylating)/ pyridoxal phosphate binding -1.279091067 -2.059016598 0.085802299 0.411627015 -1.401248671 0.939525973 0.305743432 1.211659631

At4g32770 ATSDX1__VTE1 (VITAMIN E DEFICIENT 1); tocopherol cyclase 0.391554285 -1.539614317 -0.679472857 -0.434024701 -2.694802432 0.556905166 0.339049316 -2.362872875

At4g32840 PFK6 (PHOSPHOFRUCTOKINASE 6); 6-phosphofructokinase 1.236738007 0.232008652 1.380147658 -0.215543194 0.204992845 0.191307885 -0.5365324 -0.377271243

At4g33070 "pyruvate decarboxylase, putative" -0.176672462 1.424437153 -0.062429643 0.331195179 -0.48141479 0.802784611 0.116235171 -1.807384899

At4g32410 "RSW1__CESA1 (CELLULOSE SYNTHASE 1); cellulose synthase/ transferase, transferring glycosyl groups" -0.411858537 -0.094000345 0.332606414 -1.14068731 0.16773332 -0.136996756 -0.708221328 0.231511228

At4g32520 SHM3 (SERINE HYDROXYMETHYLTRANSFERASE 3); catalytic/ glycine hydroxymethyltransferase/ pyridoxal phosphate binding 0.443701053 0.091421169 0.488545142 0.668957421 0.121654045 0.590029264 -0.69933432 0.315695173

At4g32540 YUC1 (YUCCA 1); FAD binding / NADP or NADPH binding / flavin-containing monooxygenase/ oxidoreductase 0.934636362 0.320630488 0.553377686 0.757899062 0.334541872 -0.328296614 -0.314912103 -0.535482373

At4g32180 ATPANK2 (PANTOTHENATE KINASE 2); pantothenate kinase 0.181340807 0.234765349 -0.767037448 -0.477813729 0.739954908 0.479668089 -0.076395639 0.867505922

At4g32210 SDH3-2; succinate dehydrogenase -0.067973794 0.53035018 -0.476193255 -0.02692388 0.026340636 0.441154943 -0.028158232 -0.051741236

At4g32360 NADP adrenodoxin-like ferredoxin reductase 0.656357518 0.061024502 -0.112476185 0.181403299 0.832475281 0.459256361 -0.385805992 0.500688465

At4g31990 AAT3_ATAAT1__ASP5 (ASPARTATE AMINOTRANSFERASE 5); L-aspartate:2-oxoglutarate aminotransferase -0.894710588 -0.195419364 -0.297540683 -0.231394904 0.044878387 1.060398821 -0.046210541 0.003650493

At4g31780 "MGDA__MGD1 (MONOGALACTOSYL DIACYLGLYCEROL SYNTHASE 1); 1,2-diacylglycerol 3-beta-galactosyltransferase/ UDP-galactosyltransferase/ UDP-glycosyltransferase/ transferase, transferring glycosyl groups" 0.056271918 -1.030967427 0.619627773 1.872127533 -0.355815451 -1.483743347 0.14700434 0.323059793

At4g31810 enoyl-CoA hydratase/isomerase family protein 1.240064622 1.18474812 0.891893085 1.025115018 -0.113400962 0.802556647 -0.843733218 0.186772855

At4g31870 ATGPX7 (glutathione peroxidase 7); glutathione peroxidase -1.017701073 -1.796379117 -0.607116156 -0.015824123 -4.548552519 -0.98000642 0.695923542 -0.740816032

At4g31500 "ATR4_RED1_RNT1_SUR2__CYP83B1 (CYTOCHROME P450 MONOOXYGENASE 83B1); oxidoreductase, acting on paired donors, with incorporation or reduction of molecular oxygen, NADH or NADPH as one donor, and incorporation of one atom of oxygen / oxygen binding" -1.166317212 0.718999051 -0.408581341 1.170426033 0.487552926 -0.836973414 -1.737289981 3.532456813

At4g30950 FADC_SFD4__FAD6 (FATTY ACID DESATURASE 6); omega-6 fatty acid desaturase -0.032751256 -1.644655473 0.623309848 -0.418383316 -0.193761636 -0.246497579 -0.182529198 -0.227565522

At4g31180 "aspartyl-tRNA synthetase, putative / aspartate--tRNA ligase, putative" 0.690467706 0.368046988 -0.192197356 -0.068873924 0.332941005 0.48484878 -0.962387815 0.116226712

At4g30570 "GDP-mannose pyrophosphorylase, putative" 0.479157517 0.028311352 0.049644756 0.075855946 0.327360025 -0.274653635 0.067347915 -0.347341807

At4g30580 EMB1995__ATS2; 1-acylglycerol-3-phosphate O-acyltransferase/ acyltransferase -0.403846453 -0.567839302 0.864261084 -0.228058529 0.649184007 -0.676264486 -0.089145274 -0.476097114

At4g30440 GAE1 (UDP-D-GLUCURONATE 4-EPIMERASE 1); UDP-glucuronate 4-epimerase/ catalytic -1.173674444 -0.245631049 0.753661832 -0.321030624 -0.548189993 -1.535679315 1.079560281 -0.579066114

At4g30470 cinnamoyl-CoA reductase-related 0.871129369 -0.417912851 -1.655015723 -0.250424427 -3.11210318 0.457233993 -0.242537339 -1.130282897

At4g30210 AR2__ATR2 (ARABIDOPSIS P450 REDUCTASE 2); NADPH-hemoprotein reductase -0.039022184 -0.057152254 -0.252188077 0.94630534 -1.806505908 -2.0324309 -0.045490593 0.552053075

At4g30000 "dihydropterin pyrophosphokinase, putative / dihydropteroate synthase, putative / DHPS, putative" 0.303103362 0.079272917 0.674556003 0.012817048 0.155595089 0.543083043 -0.325798559 -0.040382142

At4g29570 "cytidine deaminase, putative / cytidine aminohydrolase, putative" 0.01452968 -0.097536959 -0.28210989 -0.029785137 -0.213045897 0.213516881 0.279306117 -1.427658605

At4g29580 "cytidine deaminase, putative / cytidine aminohydrolase, putative" -0.114189124 0.215415163 0.284977787 0.239371917 0.169728326 -0.04765928 0.301769331 -0.91812414

At4g29600 "cytidine deaminase, putative / cytidine aminohydrolase, putative" 0.136577997 0.129947235 0.183886663 0.153691092 0.154357429 -0.206419209 0.04111149 -0.350034148

At4g29610 "cytidine deaminase, putative / cytidine aminohydrolase, putative" -0.123945673 0.018728257 1.215657713 0.839985259 0.840439488 -1.175016385 0.577602997 -0.485400504

At4g29620 "cytidine deaminase, putative / cytidine aminohydrolase, putative" -0.162458639 0.141494526 0.216444556 -0.000374391 0.002817697 -0.135582593 0.375535236 -0.677842849

At4g29630 "cytidine deaminase, putative / cytidine aminohydrolase, putative" -0.023592766 0.222419293 0.287735534 0.211145518 0.155509842 -0.463977723 0.264180985 -0.660367358

At4g29640 "cytidine deaminase, putative / cytidine aminohydrolase, putative" -0.081020308 0.175600969 0.318383818 0.290659525 0.003927532 -0.20949885 0.292457078 -1.105641571

At4g29650 cytidine deaminase 4 (CDA4) (desH) / cytidine aminohydrolase -0.136988089 0.162455375 0.267082545 0.113866946 0.132808609 -0.280895126 0.343973793 -0.944883567

At4g29720 ATPAO5 (Polyamine oxidase 5); amine oxidase/ electron carrier/ oxidoreductase 0.083019809 0.266202035 1.257580898 -0.71563444 -0.511789247 0.29128838 -0.807354981 -0.13696236

At4g29740 ATCKX4__CKX4 (CYTOKININ OXIDASE 4); amine oxidase/ cytokinin dehydrogenase -1.848083377 1.143022809 1.142709599 1.294302073 -0.434156787 -1.658014469 -0.197206728 0.28380593

At4g29840 TS__MTO2 (METHIONINE OVER-ACCUMULATOR 2); threonine synthase -0.028058561 0.633418776 0.626177452 1.237804054 1.165149465 0.550668882 -0.98945324 -1.387335516

At4g29130 ATHXK1_GIN2__HXK1 (HEXOKINASE 1); ATP binding / fructokinase/ glucokinase/ hexokinase -0.239694038 0.588490084 -0.197145783 0.34963645 -0.497295203 1.148591247 1.355924356 1.187794477

At4g29210 GGT3__GGT4 (GAMMA-GLUTAMYL TRANSPEPTIDASE 4); gamma-glutamyltransferase/ glutathione gamma-glutamylcysteinyltransferase 0.505453398 0.551916401 -1.548602336 0.408784998 1.33877143 0.853110262 -0.456635696 2.041090594

At4g29220 PFK1 (PHOSPHOFRUCTOKINASE 1); 6-phosphofructokinase -0.908761005 -0.177402827 -0.217502657 -0.0660834 0.500379677 0.428292731 0.355040608 1.351368562

At4g28750 PSAE-1 (PSA E1 KNOCKOUT); catalytic 0.108232251 -3.895170541 -0.30247573 -0.233466077 -0.205138772 -0.308452697 -0.730524209 0.046141039

At4g29010 AIM1 (ABNORMAL INFLORESCENCE MERISTEM); enoyl-CoA hydratase 0.086049981 -0.111705686 -1.437545283 -0.611670531 1.686801716 0.248631273 0.347681415 -0.026361808

At4g28680 "tyrosine decarboxylase, putative" 2.884658614 -0.09037533 1.193938579 0.623400162 -0.29593545 0.239543923 1.930446892 0.823710899

At4g28410 aminotransferase-related -0.465791938 1.016433593 0.479153674 -0.377131752 0.342409748 -0.371431884 0.118080793 -0.079204175

At4g27820 "BGLU9 (BETA GLUCOSIDASE 9); catalytic/ cation binding / hydrolase, hydrolyzing O-glycosyl compounds" -0.612232129 -1.164074293 -0.734177989 1.083744575 0.767560476 0.927847206 0.140396916 0.440004907

At4g27830 "BGLU10 (BETA GLUCOSIDASE 10); catalytic/ cation binding / hydrolase, hydrolyzing O-glycosyl compounds" 0.027086114 -0.71156462 -3.500719951 0.109351494 0.091000478 0.76273859 -0.274307996 0.451600365

At4g28090 sks10 (SKU5 Similar 10); copper ion binding / oxidoreductase 0.057969423 0.213223697 0.808816216 0.407990537 -0.107694575 -0.701980665 0.952220961 -0.723583833

At4g27600 pfkB-type carbohydrate kinase family protein -0.465387702 -0.980849705 0.962760003 0.507826855 0.407384943 -0.337552054 0.523904517 -0.176342489

At4g27440 PORB (PROTOCHLOROPHYLLIDE OXIDOREDUCTASE B); oxidoreductase/ protochlorophyllide reductase 0.521039121 -2.504382139 1.282180963 -0.731284553 5.348176731 -1.154168507 -0.677379244 0.102238815

At4g27550 "TPS4__ATTPS4; alpha,alpha-trehalose-phosphate synthase (UDP-forming)/ transferase, transferring glycosyl groups" -0.087002696 0.166766453 0.273909544 0.001623912 -0.159938832 -0.025743287 0.319421341 -1.005426719

At4g27570 glycosyltransferase family protein 1.3622641 0.726274819 0.518632384 3.763886352 0.187619317 1.291419354 -0.966926972 1.653670383

At4g27070 TSB2 (TRYPTOPHAN SYNTHASE BETA-SUBUNIT 2); tryptophan synthase -0.387602388 0.934786477 -0.353138866 2.221554028 1.668148475 -1.202629173 -0.288723337 0.001714626

At4g27250 dihydroflavonol 4-reductase family / dihydrokaempferol 4-reductase family 0.486197901 0.393564241 0.247842635 0.210839891 0.094595744 0.171824813 -0.355258049 -2.479275218

At4g27260 GH3.5__WES1; indole-3-acetic acid amido synthetase 2.336094935 -0.0862732 -1.303215579 -1.757367643 -0.143006369 -1.99765242 -0.99938566 0.266601022

At4g26900 HISN4__AT-HF (HIS HF); imidazoleglycerol-phosphate synthase 0.244451478 0.00755611 1.012650566 0.274900533 -0.340759273 0.457183511 -0.790662216 0.218220369

At4g26850 VTC2 (vitamin c defective 2); GDP-D-glucose phosphorylase/ GDP-galactose:glucose-1-phosphate guanyltransferase/ GDP-galactose:mannose-1-phosphate guanyltransferase/ GDP-galactose:myoinositol-1-phosphate guanyltransferase/ galactose-1-phosphate guanylyltra -0.642031616 -1.377936811 -1.077336185 0.240775979 -2.595277289 2.047474349 0.594656004 -0.63515264

At4g26770 "phosphatidate cytidylyltransferase/ transferase, transferring phosphorus-containing groups" -0.569760765 0.923214058 0.750982676 0.194300657 0.147535029 -0.434567347 1.433854002 -0.922735029

At4g26870 "aspartyl-tRNA synthetase, putative / aspartate--tRNA ligase, putative" 1.165017849 0.053465629 0.596622682 -0.264603728 -0.050305653 0.575941519 -1.055171666 0.084210446

At4g26910 2-oxoacid dehydrogenase family protein -0.238761011 0.991419647 -0.816215056 1.251386939 0.729276602 -0.078688097 -0.187525003 1.38164955

At4g26970 aconitate hydratase/ copper ion binding -0.595798207 0.920543106 -0.782935808 0.482538679 0.119671932 0.181739524 0.023704321 1.537242253

At4g26520 "fructose-bisphosphate aldolase, cytoplasmic" 0.299004801 -1.255643917 1.20142694 0.092642992 0.875909955 0.477008272 0.348103066 -0.326047907

At4g26510 "ATP binding / kinase/ phosphotransferase, alcohol group as acceptor / uracil phosphoribosyltransferase" -0.008448827 -0.204302685 0.755374933 -0.030787966 0.982136278 0.229095245 0.38113667 0.362820288

At4g26530 "fructose-bisphosphate aldolase, putative" -2.408132705 -3.237050079 1.098521421 -0.825470128 1.760528254 -0.39701449 0.988421609 -0.276210304

At4g26220 "caffeoyl-CoA 3-O-methyltransferase, putative" -0.984830572 1.354551774 -0.057739991 -2.389923489 -0.640737745 0.429283334 0.028886785 -1.68470551

At4g26270 PFK3 (PHOSPHOFRUCTOKINASE 3); 6-phosphofructokinase 1.772054918 0.522013148 -1.477838575 2.113338224 2.503944364 0.772444745 0.375203161 0.358334018

At4g26200 ACS7; 1-aminocyclopropane-1-carboxylate synthase 0.11986233 0.371854406 -0.62862666 0.546101437 -1.085499988 -1.907559027 -1.311569277 -3.652095401

At4g26250 "AtGolS6 (Arabidopsis thaliana galactinol synthase 6); transferase, transferring glycosyl groups / transferase, transferring hexosyl groups" 0.123941723 0.206367565 0.177636926 0.320723746 0.178937308 -0.259119991 0.123906412 -0.403528724

At4g26260 MIOX4; inositol oxygenase 3.016870705 0.282614908 0.232156876 -0.454634863 0.203219593 -1.121464029 3.524030097 0.638605905

At4g26300 emb1027 (embryo defective 1027); ATP binding / aminoacyl-tRNA ligase/ arginine-tRNA ligase/ nucleotide binding 0.358923294 0.246871068 0.838499537 0.188951712 -0.16429258 0.436201255 -0.802095698 -0.086000593

At4g26390 "pyruvate kinase, putative" -0.281054046 0.587970938 0.690935046 0.834185367 0.181760047 -0.213600056 1.716233509 -0.264879401

At4g25700 B1_chy1__BETA-OHASE 1 (BETA-HYDROXYLASE 1); carotene beta-ring hydroxylase -0.126227392 -1.488188763 -1.036560866 0.240753191 -1.95181983 0.421128739 0.76250922 -0.798692268

At4g25970 PSD3 (phosphatidylserine decarboxylase 3); phosphatidylserine decarboxylase 0.428952632 0.157138721 -0.535089697 -0.664661061 2.544223963 0.154716339 -0.476092262 0.986608656

At4g25840 GPP1 (glycerol-3-phosphatase 1); catalytic/ hydrolase 0.217434974 0.543539337 -0.09595643 -0.106835838 0.17663287 0.216462974 0.132744726 0.611204722

At4g25280 adenylate kinase family protein 0.129799285 0.12118691 0.271316456 0.029162125 -0.326672969 -0.05552045 0.065359885 -0.664878665

At4g25300 "oxidoreductase, 2OG-Fe(II) oxygenase family protein" -0.789053707 -0.022136636 -0.713107592 -0.326210551 -0.867425679 0.363739392 -0.29432396 0.137924518

At4g25420 AT2301_ATGA20OX1_GA5__GA20OX1; gibberellin 20-oxidase -0.06004981 -0.140210244 0.641681796 -0.407950276 -0.132427779 -0.289962602 0.760432987 -0.588061484

At4g25100 FSD1 (FE SUPEROXIDE DISMUTASE 1); copper ion binding / superoxide dismutase -3.83389926 -0.249217967 -1.064271885 -1.80314778 -1.250637625 -0.013461524 -1.673320115 -0.795936225

At4g25000 ATAMY1__AMY1 (ALPHA-AMYLASE-LIKE); alpha-amylase -0.326724745 -0.643090982 -2.61322735 -0.462318565 0.066331511 0.865847624 0.791131756 -0.694996014

At4g25080 CHLM (magnesium-protoporphyrin IX methyltransferase); magnesium protoporphyrin IX methyltransferase -0.005461511 -2.315372281 1.351473115 -1.335711784 0.091158115 -0.469179421 -0.630121539 0.482566144

At4g24650 ATIPT4; adenylate dimethylallyltransferase -0.017866953 0.163240954 0.212683243 0.259387918 0.187564085 -0.277634894 0.07323368 -0.382279908

At4g24620 PGI1 (PHOSPHOGLUCOSE ISOMERASE 1); glucose-6-phosphate isomerase -0.476635797 0.419383612 0.913591596 -0.316139867 -0.359555254 0.788933833 0.238240016 0.633748155

At4g24260 "KOR3__ATGH9A3 (ARABIDOPSIS THALIANA GLYCOSYL HYDROLASE 9A3); catalytic/ hydrolase, hydrolyzing O-glycosyl compounds" 0.5647095 -0.043154564 0.238849669 -0.302466092 -0.305991479 0.252732178 0.82458449 -0.612635251

At4g23990 "CSLG3__ATCSLG3; cellulose synthase/ transferase/ transferase, transferring glycosyl groups" 1.101274217 -0.564150791 -0.810472543 1.876250703 0.476745679 1.151037667 -0.166084757 -0.960976346

At4g24010 "CSLG1__ATCSLG1; cellulose synthase/ transferase/ transferase, transferring glycosyl groups" -0.409499158 -0.048473262 -0.195310471 0.233149772 0.226786995 0.080724545 0.789362751 -0.323894349

At4g23920 UGE2 (UDP-D-glucose/UDP-D-galactose 4-epimerase 2); UDP-glucose 4-epimerase/ protein dimerization 0.650566789 0.120144777 -0.523315549 1.332241204 0.426739879 -0.361800054 -0.011909126 -0.475872559

At4g24000 "CSLG2__ATCSLG2; cellulose synthase/ transferase/ transferase, transferring glycosyl groups" 2.409885039 -1.120001367 -4.42249178 0.125047853 -0.453064889 1.42711552 1.336480034 0.840376483

At4g23900 nucleoside diphosphate kinase 4 (NDK4) -0.600235812 -0.085062362 0.222565395 -0.034085368 -0.021585091 0.461696169 2.251105042 0.994848891

At4g23850 long-chain-fatty-acid--CoA ligase / long-chain acyl-CoA synthetase -0.080516921 -0.191567026 -0.888219324 -0.558952235 -0.602682424 -0.498937879 -1.008262364 0.675080498

At4g24040 "ATTRE1__TRE1 (TREHALASE 1); alpha,alpha-trehalase/ trehalase" 1.494465694 -0.81851612 -1.982217022 -0.311015358 0.557505412 -0.606765066 0.761964394 -1.751454515

At4g24160 "hydrolase, alpha/beta fold family protein" -0.249501414 0.868030703 -0.184337497 1.191503561 -1.763407677 -2.5434626 -0.03176387 0.227888099

At4g23590 aminotransferase class I and II family protein -0.35861446 0.83201691 0.213604596 0.146496955 0.400001698 -0.390539574 0.072603468 -0.227961177

At4g23660 AtPPT1 (Arabidopsis thaliana polyprenyltransferase 1); 4-hydroxybenzoate nonaprenyltransferase 0.776646372 0.127557787 -0.112335868 -0.16035869 0.143174981 -0.024284195 0.445156977 -0.05737289

At4g23600 JR2__CORI3 (CORONATINE INDUCED 1); cystathionine beta-lyase/ transaminase -0.75465685 -1.310508818 -2.07584285 5.59756681 3.026019101 1.564386419 -0.38129038 0.391876378

At4g22930 "DHOASE__PYR4 (PYRIMIDIN 4); dihydroorotase/ hydrolase/ hydrolase, acting on carbon-nitrogen (but not peptide) bonds, in cyclic amides" -0.675517646 0.496611324 0.930344127 0.284981468 0.025525717 0.243499031 0.110317602 0.327122967

At4g23100 ATECS1_CAD2_PAD2_RML1__GSH1 (GLUTAMATE-CYSTEINE LIGASE); glutamate-cysteine ligase -0.29890655 0.112575906 -0.763041721 -0.123745759 0.457501 -0.260027842 -0.776435338 0.373111404

At4g22870 "leucoanthocyanidin dioxygenase, putative / anthocyanidin synthase, putative" -1.433602664 -0.397441841 -0.252895902 2.35132017 -2.279802249 2.784438725 -1.12009605 -2.371040322

At4g22590 "trehalose-6-phosphate phosphatase, putative" -0.213128318 1.181719891 -0.123593936 1.289933874 -1.046790517 -1.348264576 1.610714263 -0.203716347

At4g22570 APT3 (ADENINE PHOSPHORIBOSYL TRANSFERASE 3); adenine phosphoribosyltransferase -0.037681701 -1.817179646 0.136400762 -1.93434043 -2.503715808 0.729622621 0.732421808 2.396973915

At4g22100 "BGLU3 (BETA GLUCOSIDASE 2); catalytic/ cation binding / hydrolase, hydrolyzing O-glycosyl compounds" -0.159610089 0.450777039 0.187810412 0.656720627 0.404529571 -0.474676386 -0.254979106 -2.953497446

At4g21990 ATAPR3_PRH-26_PRH26__APR3 (APS REDUCTASE 3); adenylyl-sulfate reductase -1.50904674 0.93281147 0.322658014 2.326780441 -1.634966969 -0.709954269 -0.027282001 0.458289406

At4g22110 "alcohol dehydrogenase, putative" -0.691512672 0.840346141 0.407352857 -0.831319908 -0.139586874 0.321834174 -0.21379277 -0.329345301

At4g22340 "phosphatidate cytidylyltransferase, putative / CDP-diglyceride synthetase, putative" 0.03039181 0.009615699 -0.5189195 -0.2979385 -0.258158157 0.158589367 -0.196614222 -0.553779945

At4g22010 sks4 (SKU5 Similar 4); copper ion binding / oxidoreductase 1.183830551 0.353366045 2.981626884 -0.533111188 -0.535890289 -0.587260704 -0.062015649 0.165345885

At4g21690 ATGA3OX3__GA3OX3 (GIBBERELLIN 3-OXIDASE 3); iron ion binding / oxidoreductase -0.003419187 0.414733358 0.357053302 0.344678219 0.027159833 -0.16767283 0.71374058 -0.689916867

At4g21280 "PSBQ_PSBQ-1_PSBQA__oxygen-evolving enhancer protein 3, chloroplast, putative (PSBQ1) (PSBQ)" -0.074770817 -3.285317464 0.821998527 0.077332575 -0.151629951 -0.098683298 -0.20780724 0.824780452

At4g21470 ATFMN/FHY (riboflavin kinase/FMN hydrolase); FMN adenylyltransferase/ riboflavin kinase 0.439986047 0.039231069 -1.118734739 0.170911442 -0.427935989 0.40417039 -0.259946806 0.14174896

At4g20990 ATACA4__ACA4 (ALPHA CARBONIC ANHYDRASE 4); carbonate dehydratase/ zinc ion binding 0.08982544 0.197228912 0.23632157 0.454907389 0.0851354 -0.462901623 0.099162735 -0.475713113

At4g20960 cytidine/deoxycytidylate deaminase family protein 0.088130329 -0.681640034 0.96868438 0.587670405 0.300711773 0.21208431 -0.578486609 -0.434147895

At4g20870 FAH2 (FATTY ACID HYDROXYLASE 2); catalytic/ fatty acid alpha-hydroxylase -0.14413655 0.197394086 1.965004804 -0.915079174 -0.122053882 -0.90412901 2.19589014 1.063031006

At4g21090 adrenodoxin-like ferredoxin 1 0.38118954 0.264621186 -0.088817963 0.666520609 -0.035578016 0.126345696 -0.574536605 -0.137378631

At4g21200 ATGA2OX8__GA2OX8 (GIBBERELLIN 2-OXIDASE 8); gibberellin 2-beta-dioxygenase 0.610296117 0.262643972 0.668219951 0.501327279 -0.694437468 -1.234249589 0.095431436 -0.194178891

At4g20150 unknown protein 0.179865893 0.222786794 0.039612079 -0.622976543 0.14756336 0.345709357 -0.290681783 0.429604142

At4g20460 NAD-dependent epimerase/dehydratase family protein 0.035984369 1.962901872 0.068349339 0.287951317 -0.822187571 0.080326974 1.789036552 -0.546033125

At4g20320 CTP synthase/ catalytic 3.805656073 0.584197832 -1.037516326 -0.495078488 -0.066673394 1.448822205 0.222372762 0.809385175

At4g20050 QRT3 (QUARTET 3); polygalacturonase 1.078923099 -0.020692597 0.049850761 -0.766681819 -0.471475647 0.758454898 1.748356225 -0.358187665

At4g20070 ATAAH (Arabidopsis thaliana Allantoate Amidohydrolase); allantoate deiminase/ metallopeptidase -1.088549949 -0.041559493 0.123504736 -1.185267057 -1.327854103 -1.13131503 1.066301324 -0.227169645

At4g19680 IRT2; iron ion transmembrane transporter/ zinc ion transmembrane transporter -0.874893843 1.414758704 0.376448252 -1.183856193 -0.248683862 0.258727262 0.043216584 0.523557312

At4g19710 "AK-HSDH_AK-HSDH II__bifunctional aspartate kinase/homoserine dehydrogenase, putative / AK-HSDH, putative" -0.169948208 -0.275671284 1.351053091 0.420426949 -0.913470319 0.228312023 -0.82988863 -0.530616364

At4g19860 lecithin:cholesterol acyltransferase family protein / LACT family protein -0.25798151 0.17890232 -1.345944072 -1.491610323 1.517487479 -0.498268783 -0.075102997 -0.548586252

At4g19690 IRT1 (iron-regulated transporter 1); cadmium ion transmembrane transporter/ copper uptake transmembrane transporter/ iron ion transmembrane transporter/ manganese ion transmembrane transporter/ zinc ion transmembrane transporter -1.454691095 2.458206809 1.295961436 -1.875162894 -1.302982693 1.29748888 1.842184328 1.473445753

At4g19230 CYP707A1; (+)-abscisic acid 8'-hydroxylase/ oxygen binding 1.055770479 0.366803136 -0.658790308 0.202643434 -0.433559142 -1.07041064 0.215489654 -0.604601344

At4g19010 4-coumarate--CoA ligase family protein / 4-coumaroyl-CoA synthase family protein 0.307269347 0.359088503 -0.004463033 0.719384879 0.253942116 -0.384993199 -0.199222988 -0.171546213

At4g18780 "ATCESA8_CESA8_LEW2__IRX1 (IRREGULAR XYLEM 1); cellulose synthase/ transferase, transferring glycosyl groups" -0.767888567 0.190496341 0.219068968 -2.166865782 -0.252354634 0.095038587 0.984139088 0.849891316

At4g18480 CH-42_CH42_CHL11_CHLI-1__CHLI1; ATPase/ magnesium chelatase -0.074892557 -2.721927283 1.209599739 -0.456184397 -0.496224033 0.292651223 -0.579516523 0.629545087

At4g18360 "(S)-2-hydroxy-acid oxidase, peroxisomal, putative / glycolate oxidase, putative / short chain alpha-hydroxy acid oxidase, putative" 0.181669202 2.189564892 -1.47994776 -0.199940708 -0.650659288 0.63590533 -0.789249649 0.270537052

At4g18550 lipase class 3 family protein -0.101652133 0.696406043 -0.639226336 -1.739231765 -0.326695535 -0.129758333 1.041251277 0.448057344

At4g18180 glycoside hydrolase family 28 protein / polygalacturonase (pectinase) family protein -0.144495298 0.228056151 0.411725238 0.394894819 0.386205826 -0.452801947 0.445746588 -0.455617586

At4g18240 "SSIV__ATSS4; transferase, transferring glycosyl groups" -1.02935103 -0.512737057 -0.292709172 0.617710079 3.933239696 0.761376543 0.089088606 -0.760775175

At4g18350 ATNCED2__NCED2 (NINE-CIS-EPOXYCAROTENOID DIOXYGENASE 2); 9-cis-epoxycarotenoid dioxygenase -0.05107989 0.244286301 -0.320870351 0.212992929 0.260339757 -0.595758186 1.249240939 -0.710311326

At4g13770 "REF2__CYP83A1 (CYTOCHROME P450 83A1); oxidoreductase, acting on paired donors, with incorporation or reduction of molecular oxygen, NADH or NADPH as one donor, and incorporation of one atom of oxygen / oxygen binding" -4.435120334 -0.323831171 0.774095247 -0.569315529 0.31023987 1.916888683 0.026656969 0.511893955

At4g13510 ATAMT1_ATAMT1;1__AMT1;1 (AMMONIUM TRANSPORTER 1;1); ammonium transmembrane transporter -1.027237518 0.351530803 -0.659527815 1.06862473 0.90320489 -0.078218041 -0.599234099 1.641122155

At4g13780 "methionine--tRNA ligase, putative / methionyl-tRNA synthetase, putative / MetRS, putative" 0.799217353 0.391469224 0.024304259 -0.216997661 0.054574132 0.187745011 -0.670379037 0.339424862

At4g13890 EDA37_SHM5__EDA36 (EMBRYO SAC DEVELOPMENT ARREST 37); catalytic/ glycine hydroxymethyltransferase/ pyridoxal phosphate binding -0.033747843 0.784033321 1.299075131 -0.181617564 -1.286766173 0.957774058 0.235434786 1.310923053

At4g13430 "IIL1 (ISOPROPYL MALATE ISOMERASE LARGE SUBUNIT 1); 4 iron, 4 sulfur cluster binding / hydro-lyase/ lyase" -0.589487212 0.197266638 0.259787265 -0.250163493 -0.119295474 0.738263603 -0.706371491 0.635507755

At4g13360 catalytic -0.394482983 0.076015959 -0.672848355 -0.168989632 0.896077026 0.584092738 -0.074940874 -0.132784201

At4g12800 PSAL (photosystem I subunit L) 0.728207942 -4.077613328 -0.35332543 -0.367141626 -0.064887759 -0.532230189 -0.933361075 0.032710629

At4g12430 "trehalose-6-phosphate phosphatase, putative" 0.731210865 0.348363316 -0.09857045 1.65957037 0.152340542 0.057458169 2.295308596 -0.202599802

At4g12250 GAE5 (UDP-D-GLUCURONATE 4-EPIMERASE 5); UDP-glucuronate 4-epimerase/ catalytic 0.727384533 0.295777907 -0.908585945 -0.110045148 -0.143964365 0.174778161 -0.376449948 -1.083828854

At4g12440 "APT4__adenine phosphoribosyltransferase, putative" -0.39632684 0.438386522 0.325082831 -0.157106219 -0.156524342 0.012364105 -0.023920858 0.235504887

At4g11820 EMB2778_HMGS__MVA1; acetyl-CoA C-acetyltransferase/ hydroxymethylglutaryl-CoA synthase 1.474405347 0.696708456 1.118123949 -0.857980018 -0.120826907 -0.155535861 0.151669102 -0.232154386

At4g11850 MEE54__PLDGAMMA1; phospholipase D -0.27535414 0.873855681 -0.729877818 1.304975297 0.738058705 -0.835454882 -0.708487188 0.91420179

At4g11570 haloacid dehalogenase-like hydrolase family protein -0.872446956 -1.232847082 -1.492934024 -0.175085619 -0.476894817 -0.436036276 0.36641382 -1.000156167

At4g11640 ATSR (ARABIDOPSIS THALIANA SERINE RACEMASE); serine racemase 0.673916238 -0.196313942 0.624206294 0.089115467 0.369447475 -0.292445679 -0.273072328 -0.629425045

At4g11600 LSC803_PHGPX__ATGPX6 (GLUTATHIONE PEROXIDASE 6); glutathione peroxidase -0.532009627 0.885509567 -1.88167084 0.450514923 2.666545023 0.949958871 0.218391066 -0.40202392

At4g11830 PLDGAMMA2; phospholipase D 0.235026335 -0.017330235 0.208303788 -0.962658934 0.011329426 -0.52590169 -0.247666969 0.313918691

At4g11840 PLDGAMMA3; phospholipase D -0.08843686 0.214851995 -0.80050277 0.809121104 0.530845912 0.359275928 -0.347964378 0.322700725

At4g11280 ACS6 (1-AMINOCYCLOPROPANE-1-CARBOXYLIC ACID (ACC) SYNTHASE 6); 1-aminocyclopropane-1-carboxylate synthase 0.355010869 0.180933629 -0.370013274 2.332509232 -2.971298004 -3.820173784 0.040406847 1.029561105

At4g10960 UGE5 (UDP-D-glucose/UDP-D-galactose 4-epimerase 5); UDP-glucose 4-epimerase/ protein dimerization -0.371826978 0.723417696 -3.05422383 1.056360015 -0.235881269 -0.075302519 0.987747968 -0.495731156

At4g11010 NDPK3 (NUCLEOSIDE DIPHOSPHATE KINASE 3); ATP binding / nucleoside diphosphate kinase -0.329090112 0.446741714 0.472598904 -0.088208942 0.145678959 0.985160672 0.136222326 1.037846535

At4g11030 "long-chain-fatty-acid--CoA ligase, putative / long-chain acyl-CoA synthetase, putative" -0.007017029 0.665393214 0.701065244 0.685576547 0.196405065 -0.210219521 2.317334656 -0.089811781

At4g11050 "AtGH9C3 (Arabidopsis thaliana glycosyl hydrolase 9C3); carbohydrate binding / catalytic/ hydrolase, hydrolyzing O-glycosyl compounds" -0.050712104 0.712441321 0.718674773 0.062387185 0.129189041 -0.314635918 0.385232805 -0.247127164

At4g10490 "oxidoreductase, 2OG-Fe(II) oxygenase family protein" -0.045206075 0.03902437 -0.090610884 -0.405523762 -0.086714571 0.149648226 -0.333459997 -2.680441176

At4g10500 "oxidoreductase, 2OG-Fe(II) oxygenase family protein" -1.851983685 0.387968454 -2.248299589 2.159511312 0.059973744 0.098220004 -0.723474045 0.260544826

At4g09760 "choline kinase, putative" -1.935759407 -1.178946015 -1.908205713 -1.794863096 1.565202801 -0.511670338 -0.53812432 -1.017323036

At4g10120 "ATSPS4F; transferase, transferring glycosyl groups" -3.785338375 -2.592086726 -1.862852372 0.224716538 -1.531446305 2.269503043 2.296707477 -0.477453486

At4g10100 SIR5__CNX7; catalytic -0.10309956 -0.069907941 -0.624526884 -0.547187831 -0.01042263 0.020319426 -0.691959628 0.071697996

At4g10320 0.693070563 0.300440102 0.319057489 0.294719114 -0.250422511 0.572026065 -0.697294259 0.159683605

At4g09500 glycosyltransferase family protein 1.087285365 -0.285112785 -0.342165316 0.264030196 0.91009596 0.899871368 -0.34560329 -0.292768663

At4g09510 "beta-fructofuranosidase, putative / invertase, putative / saccharase, putative / beta-fructosidase, putative" 1.793637242 0.17840579 1.060516031 0.595276332 -0.061859198 0.407843808 -0.49308298 0.035650549

At4g09740 "AtGH9B14 (Arabidopsis thaliana glycosyl hydrolase 9B14); catalytic/ hydrolase, hydrolyzing O-glycosyl compounds" 0.282333961 0.232536152 -0.040822418 -0.173687994 0.139860838 -0.18003706 1.52147091 -0.354528859

At4g09820 TT8 (TRANSPARENT TESTA 8); DNA binding / transcription factor -0.22216904 -0.138859158 -0.323954294 -0.251151214 -0.47473116 0.888820171 -0.2746331 -2.019198742

At4g08870 "arginase, putative" -0.96147409 -1.535461923 -0.815357938 1.118582049 -1.669228722 -0.212108891 -0.20344244 0.349081382

At4g09320 NDPK1; ATP binding / nucleoside diphosphate kinase 0.724657012 0.170043641 0.337327194 -0.143320983 -0.397636773 0.638141402 -0.883157617 0.466287194

At4g08790 "nitrilase, putative" 0.630433395 1.96E-05 0.189087586 0.281039007 0.675870051 0.658073135 -0.552777579 0.202771871

At4g08040 ACS11; 1-aminocyclopropane-1-carboxylate synthase -0.372184722 -0.128115998 0.698974502 -0.038942084 -0.79642457 -2.572611575 -0.283937222 -0.131764469

At4g05450 adrenodoxin-like ferredoxin 2 0.09870072 0.466624935 0.684907556 0.765851952 -0.026450732 0.78893346 0.106459059 0.495004078

At4g05180 PSII-Q__PSBQ-2; calcium ion binding 0.074613012 -3.88134121 -0.277819831 -0.794331013 0.04944133 -0.291403354 -0.984824831 -0.341129794

At4g04930 DES-1-LIKE; oxidoreductase/ sphingolipid delta-4 desaturase -0.045681803 0.634945945 0.240056306 0.257183218 0.106615177 -0.182795474 5.096876697 0.26593178

At4g04610 ATAPR1_PRH19__APR1 (APS REDUCTASE 1); adenylyl-sulfate reductase -2.063396203 0.887847065 0.263434417 2.06053381 -1.558842985 -0.121139323 0.226027797 0.523077947

At4g04870 CLS (CARDIOLIPIN SYNTHASE); cardiolipin synthase/ phosphatidyltransferase 0.211670639 0.52754071 0.124820803 0.548986956 0.006517659 0.615088453 -0.474643706 -1.536749046

At4g04955 ATALN (Arabidopsis allantoinase); allantoinase/ hydrolase 0.758757352 -0.219089485 -0.083652646 -2.288910247 -0.146600475 -0.411613963 0.405343964 0.255911522

At4g04350 EMB2369 (EMBRYO DEFECTIVE 2369); ATP binding / aminoacyl-tRNA ligase/ leucine-tRNA ligase/ nucleotide binding -0.192465251 -1.064598942 0.868493806 0.521088858 0.236688593 0.388930204 -0.171204021 -0.132948572

At4g03930 enzyme inhibitor/ pectinesterase 0.020253534 0.08776898 0.222384226 0.022573783 0.092998732 -0.160814788 0.24127011 -0.458303108

At4g04040 MEE51 (maternal effect embryo arrest 51); diphosphate-fructose-6-phosphate 1-phosphotransferase -1.259341061 -0.778302014 -0.342049086 -1.117804363 1.035674602 -0.517024443 -0.290509341 0.861217754

At4g03205 "hemf2__coproporphyrinogen III oxidase, putative / coproporphyrinogenase, putative / coprogen oxidase, putative" 1.337925297 -0.256665047 0.793656495 0.185959088 1.096124647 -0.225105998 -0.355536195 0.118726396

At4g03060 -1.794607415 -0.500048381 0.51107237 0.484102334 0.792978317 1.487133342 0.647580886 0.113847571

At4g03070 "AOP1.1__AOP1; oxidoreductase, acting on paired donors, with incorporation or reduction of molecular oxygen, 2-oxoglutarate as one donor, and incorporation of one atom each of oxygen into both donors" 0.050376253 -0.702849066 -0.094122997 -0.810175415 -0.038931122 1.468777714 0.812172109 0.251733398

At4g02580 "NADH-ubiquinone oxidoreductase 24 kDa subunit, putative" 0.377150103 0.25788683 0.42154676 -0.188431498 -0.085807617 0.286142825 0.035799261 0.715433637

At4g02770 PSAD-1 (photosystem I subunit D-1) 0.116500315 -3.776374911 -0.036780349 -0.897954594 0.094742254 -0.191239927 -0.966200736 0.186289814

At4g02780 ABC33_ATCPS1__GA1 (GA REQUIRING 1); ent-copalyl diphosphate synthase/ magnesium ion binding 0.152948187 0.169423417 0.329516851 0.232708774 0.134044302 -0.215782423 0.128862716 -0.574024294

At4g03050 AOP3; iron ion binding / oxidoreductase 0.217769507 -0.023924148 -0.316758938 -0.578305736 -0.399225206 0.787035119 -0.441838673 -3.974617303

At4g02610 "tryptophan synthase, alpha subunit, putative" 0.72987995 0.028066123 0.184215189 -0.608353064 1.029864207 0.958894697 -0.666955654 0.810747278

At4g02290 "AtGH9B13 (Arabidopsis thaliana glycosyl hydrolase 9B13); catalytic/ hydrolase, hydrolyzing O-glycosyl compounds" 3.585853986 1.01040785 1.577590525 -1.696886443 -0.07369042 0.705120125 0.786190701 2.483968216

At4g02300 pectinesterase family protein -0.049777567 0.356819124 0.37710387 0.421821637 0.287007522 -0.443512899 0.507237188 -0.584549256

At4g02280 "ATSUS3__SUS3 (sucrose synthase 3); UDP-glycosyltransferase/ sucrose synthase/ transferase, transferring glycosyl groups" 0.107912269 0.317653089 -3.368440635 0.231922988 -0.10403948 0.522517384 -0.450406687 -2.592411834

At4g02320 pectinesterase family protein -0.097719362 0.298832087 0.376523605 0.327399031 0.368027911 -0.440764003 0.457797771 -0.402308962

At4g02330 ATPMEPCRB; pectinesterase -0.576879393 0.209164851 1.340371956 2.025264153 -1.444067481 -2.76207281 -1.905446507 1.665125872

At4g02120 "CTP synthase, putative / UTP--ammonia ligase, putative" -0.299242102 -0.236146584 -0.313153137 -0.220033686 0.333451924 0.235549713 0.229683659 1.699494484

At4g01690 HEMG1__PPOX; protoporphyrinogen oxidase -0.580683082 -0.825192224 0.895429965 -0.104188444 0.191998103 -0.323618669 -0.188642744 -0.155225098

At4g01950 ATGPAT3__GPAT3 (GLYCEROL-3-PHOSPHATE ACYLTRANSFERASE 3); acyltransferase -2.282438323 -0.073535161 1.430771332 1.334579111 -0.877707948 -2.992166283 -0.037975612 -0.055783785

At4g01970 "AtSTS (Arabidopsis thaliana stachyose synthase); galactinol-raffinose galactosyltransferase/ hydrolase, hydrolyzing O-glycosyl compounds" -0.155569837 0.479998747 0.163203982 -0.407104694 0.161046168 0.020199287 -0.337480254 -3.318174252

At4g01850 MAT2__SAM-2 (S-ADENOSYLMETHIONINE SYNTHETASE 2); methionine adenosyltransferase 0.738246915 0.291381255 -0.078274161 -1.065057925 -0.571003293 -0.671088844 -0.433479708 0.960125639

At4g01480 AtPPa5 (Arabidopsis thaliana pyrophosphorylase 5); inorganic diphosphatase/ pyrophosphatase -2.522923376 1.112617375 -0.937733233 -0.913065179 -0.667914796 1.131165047 2.366252761 1.817293452

At4g01070 "UGT72B1__GT72B1; UDP-glucosyltransferase/ UDP-glycosyltransferase/ transferase, transferring glycosyl groups" -1.092542397 0.322196251 -1.803735082 0.018237483 -1.008309262 1.734521265 0.442813022 0.99523304

At4g00240 PLDBETA2; phospholipase D -0.259251184 0.785741135 0.228266293 0.617687609 0.491811946 -0.516372189 0.743499353 -0.02721231

At4g00490 BMY9__BAM2 (BETA-AMYLASE 2); beta-amylase -0.01382973 -0.902759051 0.235063977 1.115343086 -0.428375425 1.535599084 0.070885571 0.829659137

At4g00520 acyl-CoA thioesterase family protein -0.234532498 0.097929288 -0.047115412 0.372790084 0.534962781 0.449566107 -0.187192819 0.357866523

At4g00550 "DGD2; UDP-galactosyltransferase/ UDP-glycosyltransferase/ digalactosyldiacylglycerol synthase/ transferase, transferring glycosyl groups" -0.046378258 0.643494092 -0.684340389 0.858470133 0.390085849 0.22431928 0.381322522 0.097308419

At4g00590 asparaginase 2 family protein 0.206680473 0.365959126 0.298701804 0.180508867 0.479308248 -0.396740185 0.208045197 -0.040356271

At4g00600 "tetrahydrofolate dehydrogenase/cyclohydrolase, putative" 1.036309096 0.589757395 0.981590291 0.991807963 -0.211324218 0.796181825 -1.571837597 -0.234132477

At4g00360 -3.377415329 -1.38156698 0.099364491 -1.24118336 -0.31201481 -1.323884594 -1.141760066 -1.86765289

At4g00400 GPAT8 (glycerol-3-phosphate acyltransferase 8); acyltransferase/ glycerol-3-phosphate O-acyltransferase 0.7957896 -1.122444104 2.348727037 -0.441330292 0.748474057 -1.034293723 -0.510034623 1.081583078

At4g00110 GAE3 (UDP-D-GLUCURONATE 4-EPIMERASE 3); UDP-glucuronate 4-epimerase/ catalytic -0.185396018 0.981775867 0.82622291 0.273894255 0.388060881 -0.07988669 1.489985298 0.148542647

At4g00190 pectinesterase family protein 0.061978874 0.192441996 0.306145614 0.202989513 0.300041409 -0.329737333 0.204733597 -0.41157387

At1g32060 PRK (PHOSPHORIBULOKINASE); ATP binding / phosphoribulokinase/ protein binding -0.421221352 -4.054565182 -0.16763404 -0.114453599 -0.175142934 0.289661537 -0.280159212 0.054468432

At1g25410 ATIPT6; ATP binding / adenylate dimethylallyltransferase/ tRNA isopentenyltransferase -0.134907731 0.090218071 0.061346087 -0.488700819 -0.20138906 0.224057328 -0.222109071 -2.761232276

At1g19940 "AtGH9B5 (Arabidopsis thaliana Glycosyl Hydrolase 9B5); catalytic/ hydrolase, hydrolyzing O-glycosyl compounds" -0.757961844 0.165574479 0.785623628 -0.62169484 -0.302158431 0.188004312 2.322058048 -0.539108423

At1g16780 "vacuolar-type H+-translocating inorganic pyrophosphatase, putative" 0.966768501 -0.258222958 0.972976491 -0.364289327 0.449687248 1.171194522 -0.018998418 0.45014357

At1g18590 SOT17 (SULFOTRANSFERASE 17); desulfoglucosinolate sulfotransferase/ sulfotransferase -2.525686755 0.358042325 -0.164106904 0.329987421 -0.340231868 0.51012899 -0.944476655 1.765958286

At1g18640 PSP (3-PHOSPHOSERINE PHOSPHATASE); phosphoserine phosphatase 0.485947258 -0.048670136 0.63892308 -0.601552354 0.651439683 0.259497022 -0.441754544 1.258305708

At1g18580 GAUT11 (Galacturonosyltransferase 11); polygalacturonate 4-alpha-galacturonosyltransferase -0.086328782 0.369960532 0.477969987 0.042681391 0.278548728 -0.068114784 -0.017055474 0.252282046

At1g19920 ASA1__APS2; sulfate adenylyltransferase (ATP) -0.337562021 -0.099518886 1.062649581 0.493192951 -0.846512329 1.413197688 0.099227227 0.851062587

At1g19670 ATHCOR1_CORI1__ATCLH1 (ARABIDOPSIS THALIANA CORONATINE-INDUCED PROTEIN 1); chlorophyllase 0.927887371 -2.073430501 0.020762068 3.075408963 0.457045056 -0.081409239 -0.062134883 1.401833617

At4g10260 pfkB-type carbohydrate kinase family protein 0.672075405 0.530517042 0.781277623 0.751845265 0.256520535 -0.166897503 2.248016526 -0.308527598

At2g40490 HEME2; uroporphyrinogen decarboxylase -0.180006654 -1.663208003 1.496652436 -0.385059729 0.353138561 -0.525730867 -0.610912289 0.185957869

At5g34930 arogenate dehydrogenase 0.460223649 0.375152262 -0.723870003 0.113788073 -0.186292002 -0.348282563 -0.236514379 0.216608333

At1g67070 PMI2__DIN9 (DARK INDUCIBLE 9); mannose-6-phosphate isomerase -0.021199941 0.118968077 -0.404757297 0.387361449 -1.27014765 -1.086058231 1.119278295 -0.817680489

At1g17960 "threonyl-tRNA synthetase, putative / threonine--tRNA ligase, putative" 0.139307333 0.549981305 -0.042294626 -0.392377979 -0.059613637 -0.352519518 -0.01451278 0.054633649

At1g17890 GER2; binding / catalytic/ coenzyme binding -0.181118048 0.92062707 0.437935096 0.359314434 0.495355292 0.122092211 -0.116367745 0.159390441

At1g66960 "lupeol synthase, putative / 2,3-oxidosqualene-triterpenoid cyclase, putative" -0.420567358 -0.061102534 -0.36941043 -0.115208302 0.291842124 -0.046863884 0.13505176 -0.107805684

At1g22210 "trehalose-6-phosphate phosphatase, putative" 0.165245672 0.212012369 0.145275209 0.185455795 0.211270667 -0.303866841 0.174146058 -0.317903783

At1g22170 phosphoglycerate/bisphosphoglycerate mutase family protein 0.782520552 0.574373522 0.633903067 -1.150662005 -0.316215916 1.142313186 0.006907253 -0.713022139

At1g12640 membrane bound O-acyl transferase (MBOAT) family protein -0.074507888 0.395402022 -1.094609386 -0.194065858 -0.2484516 0.068048399 0.795917742 -0.364251637

At1g22360 "AtUGT85A2 (UDP-glucosyl transferase 85A2); UDP-glycosyltransferase/ glucuronosyltransferase/ transferase, transferring glycosyl groups" -1.080698334 0.681765152 -0.036031664 0.26525583 0.673254856 -2.322826256 -1.592388244 1.298246217

At1g22370 "AtUGT85A5 (UDP-glucosyl transferase 85A5); glucuronosyltransferase/ transferase, transferring glycosyl groups" -0.72850871 -0.581201497 -1.483481344 0.524930688 3.214402278 -0.240985612 -0.065677773 -0.674704237

At1g22020 SHM6 (serine hydroxymethyltransferase 6); catalytic/ glycine hydroxymethyltransferase/ pyridoxal phosphate binding 0.12003392 0.305278289 -0.270577487 0.207444462 0.140915155 -0.2119342 0.318834046 -0.287227223

At1g22340 "AtUGT85A7 (UDP-glucosyl transferase 85A7); UDP-glycosyltransferase/ glucuronosyltransferase/ transferase, transferring glycosyl groups" -0.409787033 0.305032803 0.218887694 0.655794752 0.443366231 -0.656597818 -0.084940718 -0.784670005

At1g29870 "tRNA synthetase class II (G, H, P and S) family protein" -0.024216391 0.171812718 0.318211171 0.263125577 0.182269458 -0.214387827 0.04921257 -0.511992201

At1g29880 glycyl-tRNA synthetase / glycine--tRNA ligase 0.162607366 0.117560329 0.599112051 0.75393046 -0.465171536 0.562457412 -0.682438773 -0.022311622

At1g29900 CARB (CARBAMOYL PHOSPHATE SYNTHETASE B); ATP binding / carbamoyl-phosphate synthase/ catalytic 0.085636488 0.368948299 0.677368708 0.436694661 0.170550364 0.915358306 -0.92527804 -0.112772387

At1g58290 HEMA1; glutamyl-tRNA reductase -0.309880749 -1.889279311 1.408856705 -1.599789358 -2.135079457 -1.800741349 0.302674427 0.338760708

At1g07250 "UGT71C4 (UDP-GLUCOSYL TRANSFERASE 71C4); quercetin 3-O-glucosyltransferase/ quercetin 7-O-glucosyltransferase/ transferase, transferring glycosyl groups" -0.488973785 -0.542218866 -0.646920157 -1.040535721 -0.111879894 0.298100297 0.072284679 0.105085596

At1g07230 "hydrolase, acting on ester bonds" 0.558589715 -0.347382453 -1.086349789 -1.162193571 1.06532966 0.175652956 -0.012003528 -0.140558103

At1g07260 "UGT71C3 (UDP-GLUCOSYL TRANSFERASE 71C3); UDP-glycosyltransferase/ quercetin 3-O-glucosyltransferase/ transferase, transferring glycosyl groups" 0.799165525 1.770439745 0.295396145 0.216493993 0.137625933 -0.070864458 -1.637271267 -0.526120428

At1g07240 "UGT71C5 (UDP-GLUCOSYL TRANSFERASE 71C5); UDP-glycosyltransferase/ quercetin 3-O-glucosyltransferase/ transferase, transferring glycosyl groups" -0.641622404 0.617572116 -0.258413125 0.380987944 1.012407711 -1.706325504 -0.863716942 0.510367466

At1g13700 glucosamine/galactosamine-6-phosphate isomerase family protein 0.12494899 -0.834377554 0.190361295 -2.39251212 0.192784942 -2.351231118 0.253807022 -0.246363068

At1g16980 "TPS2__ATTPS2; alpha,alpha-trehalose-phosphate synthase (UDP-forming)/ transferase, transferring glycosyl groups" -0.059325133 0.21999516 0.229989204 -0.032620529 0.009599553 0.040208054 0.131162683 -1.231501912

At1g18130 tRNA synthetase-related / tRNA ligase-related -0.102846144 0.254408908 0.313907312 0.377818049 0.269074184 -0.463483227 0.125685433 -0.367192186

At1g13560 "AAPT1 (AMINOALCOHOLPHOSPHOTRANSFERASE 1); phosphatidyltransferase/ phosphotransferase, for other substituted phosphate groups" 0.33903354 0.12391854 0.65005113 0.23399728 -0.088529601 0.203101571 -0.387245372 0.145634092

At1g55090 carbon-nitrogen hydrolase family protein 0.133672278 -0.150475798 -0.393530108 -0.007689176 0.33266649 0.060182592 -0.306941568 -0.556458902

At3g08510 ATPLC2 (PHOSPHOLIPASE C 2); phospholipase C 0.226748388 -0.347123563 -0.209369863 -1.130891565 2.436312524 -0.442665214 -0.023294663 0.124509872

At1g30120 PDH-E1 BETA (PYRUVATE DEHYDROGENASE E1 BETA); pyruvate dehydrogenase (acetyl-transferring) 0.648432355 -0.407224587 0.476994754 -1.441094657 -0.912867453 0.420844485 -0.048617512 0.10541474

At1g51780 ILL5; IAA-amino acid conjugate hydrolase/ metallopeptidase 0.796735517 0.003321303 -2.025441814 2.007860756 -1.412443751 -1.928596151 -0.408318133 0.657114856

At1g51680 4CL.1_AT4CL1__4CL1 (4-COUMARATE:COA LIGASE 1); 4-coumarate-CoA ligase -1.161479491 -0.063273654 -1.114150283 -1.262030324 -2.113219412 0.014630769 -0.13797867 0.559679997

At1g30100 ATNCED5__NCED5 (NINE-CIS-EPOXYCAROTENOID DIOXYGENASE 5); 9-cis-epoxycarotenoid dioxygenase 0.341893359 0.094573211 -0.132819624 0.597266713 0.264534418 -0.344278127 0.105034482 -1.401371298

At1g50960 ATGA2OX7__GA2OX7 (GIBBERELLIN 2-OXIDASE 7); gibberellin 2-beta-dioxygenase 0.021340533 0.276108099 0.258040352 0.285515182 0.19967966 -0.286685827 0.243340658 -0.411696699

At1g56190 "phosphoglycerate kinase, putative" -1.474557858 -0.909435767 0.60147225 0.018311569 -0.035305003 1.186529512 0.265932117 0.517277555

At3g11340 UDP-glucoronosyl/UDP-glucosyl transferase family protein 0.285939102 1.865364527 -3.26944226 0.107345659 0.581018364 0.411829636 -1.298935755 2.195317482

At3g12290 "tetrahydrofolate dehydrogenase/cyclohydrolase, putative" 0.332008775 -0.162244745 -0.02782265 -0.648555914 1.479130714 0.170610104 -0.140314486 1.149379305

At3g12260 complex 1 family protein / LVR family protein 0.133323887 0.276716661 0.054207586 -0.325564105 0.414697533 0.511862622 -0.140687142 0.19296109

At3g12120 FAD2 (FATTY ACID DESATURASE 2); delta12-fatty acid dehydrogenase/ omega-6 fatty acid desaturase 0.50053367 0.160521731 0.184303082 -1.10347925 0.990987213 0.219148907 0.063341806 -0.320633531

At1g30370 lipase class 3 family protein 0.05103726 1.483759645 0.387208011 1.344367126 -0.986795961 -2.699062014 -0.557590874 0.267695916

At1g30380 PSAK (photosystem I subunit K) 0.41583353 -3.38964519 -0.126721294 -0.911005632 -0.417271068 -0.037852765 -0.872810942 0.428652478

At1g35910 "trehalose-6-phosphate phosphatase, putative" 1.243627402 0.228646994 -1.545702895 0.980583679 -1.020623272 -0.376795976 0.894218453 0.582137043

At1g55020 LOX1; lipoxygenase 0.517629035 0.661854823 -1.964719165 -1.271271949 -1.353267142 0.865495613 -0.571075157 -0.097989362

At3g02350 "GAUT9 (Galacturonosyltransferase 9); polygalacturonate 4-alpha-galacturonosyltransferase/ transferase, transferring glycosyl groups / transferase, transferring hexosyl groups" -0.62458768 0.191797744 0.583477064 -0.402885663 -0.397757476 -0.294549853 0.026489441 0.408042928

At3g02360 6-phosphogluconate dehydrogenase family protein 0.421131511 0.718272132 -0.948414647 0.013613136 -0.510461361 0.482031538 -0.076112336 0.596139958

At1g66520 "pde194 (pigment defective 194); catalytic/ formyltetrahydrofolate deformylase/ hydroxymethyl-, formyl- and related transferase" 0.165213909 0.07799927 1.065346208 0.994203337 0.22746597 -0.07063069 -0.146411386 -0.294863365

At1g66530 "arginyl-tRNA synthetase, putative / arginine--tRNA ligase, putative" 1.220052325 0.297419606 0.465940505 -0.002448244 -0.130541787 0.997096206 -0.774269024 0.686354604

At1g66800 cinnamyl-alcohol dehydrogenase family / CAD family -0.990234775 2.365534304 0.579695394 -1.330365919 -0.096600857 -0.291547166 -0.059756181 0.770105544

At3g11170 FADD__FAD7 (FATTY ACID DESATURASE 7); omega-3 fatty acid desaturase -0.603830148 -1.673663796 1.523679897 1.398175057 -0.993779293 -0.60852743 0.23963832 0.447005135

At3g11180 "oxidoreductase, 2OG-Fe(II) oxygenase family protein" -0.513025259 0.401508988 0.183486389 -0.229401384 -0.35203604 0.321942834 0.796446653 -3.656459113

At3g10920 MEE33__MSD1 (MANGANESE SUPEROXIDE DISMUTASE 1); metal ion binding / superoxide dismutase 0.03098632 0.257536182 -0.15267425 0.000725789 0.360591386 0.402386757 -0.37945848 0.628053351

At1g75290 "oxidoreductase, acting on NADH or NADPH" 0.001743667 0.439321762 -0.381336169 -0.113190329 -0.017238154 -0.011938889 1.457014572 0.034692793

At1g75270 DHAR2 (DEHYDROASCORBATE REDUCTASE 2); glutathione binding / glutathione dehydrogenase (ascorbate) 0.013874698 1.138217754 -0.469045323 0.630996314 -0.564303961 0.408220189 0.012249856 0.942872522

At1g36180 ACC2 (ACETYL-COA CARBOXYLASE 2); acetyl-CoA carboxylase 0.92532851 0.373760908 0.539419811 0.715400007 0.271710477 0.440843034 -0.047655779 -0.018868084

At4g18440 "adenylosuccinate lyase, putative / adenylosuccinase, putative" -1.005515413 -0.239238613 1.393202436 1.916691789 1.17726179 -0.186465592 -0.54543915 0.089226463

At1g66030 CYP96A14P; electron carrier/ heme binding / iron ion binding / monooxygenase -0.164875081 0.355054975 0.36596063 0.110193238 0.13449303 -0.335371922 0.198472035 -0.282985549

At1g66200 ATGSR2; copper ion binding / glutamate-ammonia ligase -1.449502553 -0.120845278 -0.937198736 -1.912756401 -1.217299566 1.071299324 0.107112762 1.189446305

At3g31415 terpene synthase/cyclase family protein -0.702602391 1.621141538 0.507590157 -0.838624982 0.030066758 -0.125052556 -0.068406709 0.273774131

At3g14790 ATRHM3__RHM3 (RHAMNOSE BIOSYNTHESIS 3); UDP-L-rhamnose synthase/ catalytic 0.2125328 0.747440692 1.128083311 0.396738859 -0.52836915 0.349386472 0.78289727 0.351948945

At3g30180 CYP85A2__BR6OX2 (BRASSINOSTEROID-6-OXIDASE 2); monooxygenase/ oxygen binding 0.263341701 -0.308602419 -0.930184009 -0.484858294 1.404567466 1.011054288 0.459721916 0.144143239

At3g22250 UDP-glucoronosyl/UDP-glucosyl transferase family protein 0.033166609 0.358515771 0.345320999 0.361432433 0.346489481 -0.698480331 0.238331349 -0.35522874

At3g13610 "oxidoreductase, 2OG-Fe(II) oxygenase family protein" -0.541583743 2.381979923 -1.497706444 -0.449079754 -0.864393201 0.288348995 -0.194978904 2.559190585

At3g18850 LPAT5; acyltransferase 3.015217082 -1.230930857 0.65975833 -1.048015927 0.796103931 0.449832797 1.71335213 1.058653461

At3g11980 "FAR2__MS2 (MALE STERILITY 2); fatty acyl-CoA reductase (alcohol-forming)/ oxidoreductase, acting on the CH-CH group of donors, NAD or NADP as acceptor" 1.338131955 -0.282792268 0.229440195 -0.092108935 -0.300949101 0.076441739 0.45756087 0.060485068

At3g52340 ATSPP2__SPP2 (SUCROSE-6F-PHOSPHATE PHOSPHOHYDROLASE 2); catalytic/ magnesium ion binding / phosphatase/ sucrose-phosphatase -0.352148573 -1.080097703 -0.627344646 0.157456125 -2.182518757 -0.174612956 -0.519194258 -1.393037197

At3g32040 "geranylgeranyl pyrophosphate synthase, putative / GGPP synthetase, putative / farnesyltranstransferase, putative" -0.587260655 1.106297208 0.471847106 -0.33647603 0.19806255 0.029459941 0.109795697 0.073898147

At3g25660 "glutamyl-tRNA(Gln) amidotransferase, putative" -0.414847339 -0.812710822 1.64303456 0.526665299 0.239576825 -0.129712813 0.015856932 -0.183960541

At3g29430 "geranylgeranyl pyrophosphate synthase, putative / GGPP synthetase, putative / farnesyltranstransferase, putative" -0.08161648 0.837882249 0.493373829 -0.112190167 0.371845901 -0.337790315 -0.025603038 0.121161577

At3g29360 "UDP-glucose 6-dehydrogenase, putative" -0.181585486 0.695383916 0.902197779 0.920486454 -0.348253269 -0.51234128 0.168521276 0.682233259

At3g22200 GABA-T_HER1__POP2 (POLLEN-PISTIL INCOMPATIBILITY 2); 4-aminobutyrate transaminase/ 4-aminobutyrate:pyruvate transaminase 0.655937115 0.514918126 -1.556544788 -0.689658584 2.049476414 -0.008291822 -0.063422928 1.567382565

At3g13682 LDL2 (LSD1-LIKE2); amine oxidase/ electron carrier/ oxidoreductase 0.348290206 0.319380913 0.546368965 0.319621749 0.153798245 -0.076175283 0.037031194 -0.487008464

At3g13790 "ATCWINV1__ATBFRUCT1; beta-fructofuranosidase/ hydrolase, hydrolyzing O-glycosyl compounds" -1.732051571 1.242397392 -1.370151581 0.119844675 -1.52271917 -1.586101973 -2.802188464 2.265362731

At3g21420 "oxidoreductase, 2OG-Fe(II) oxygenase family protein" 1.078376382 0.418483413 -0.626757135 -0.410091353 -0.462181527 0.183118988 0.640723075 0.116617599

At3g22890 APS1 (ATP SULFURYLASE 1); sulfate adenylyltransferase (ATP) -1.427619956 -0.466395681 -0.454357794 0.239214921 -1.102626073 -0.992167329 -1.093656197 0.336270513

At3g22960 PKP1__PKP-ALPHA; pyruvate kinase 0.452040227 -0.168247295 0.866872867 -1.190318207 0.617388751 0.149051198 -0.711532068 -0.288510301

At3g27980 pectinesterase family protein 0.153458988 0.157643126 0.150451719 0.057701696 0.133600309 -0.197540032 0.032956275 -0.249441012

At3g23920 BMY7_TR-BAMY__BAM1 (BETA-AMYLASE 1); beta-amylase -0.052205582 -0.413870001 -1.939766374 0.583535388 -2.291873093 -0.774365576 0.979808949 -1.683853439

At3g23820 GAE6 (UDP-D-GLUCURONATE 4-EPIMERASE 6); UDP-glucuronate 4-epimerase/ catalytic -1.023315113 -0.107227106 0.650170463 -0.483301346 0.00700896 0.064950073 0.377351057 1.081197513

At3g26380 glycosyl hydrolase family protein 27 / alpha-galactosidase family protein / melibiase family protein 0.799126837 -0.429067678 -0.75990672 -0.074641421 0.537419511 0.962496531 -0.524773893 -1.000668452

At3g19000 "oxidoreductase, 2OG-Fe(II) oxygenase family protein" -1.337933693 -0.414837358 -0.285470689 -1.297621577 -0.173973389 1.196251678 -0.248972044 -0.86356832

At3g24090 glutamine-fructose-6-phosphate transaminase (isomerizing)/ sugar binding / transaminase 0.813895373 1.066139079 0.115461314 2.291433201 0.267812873 -0.147550165 -0.253034915 0.151617713

At3g19010 "oxidoreductase, 2OG-Fe(II) oxygenase family protein" -1.33986131 0.177608212 -1.106319961 1.252723009 0.360487038 -2.250674028 0.729017446 2.207686038

At3g29590 AT5MAT; O-malonyltransferase/ transferase -0.501128807 0.416994818 0.007812023 2.041190936 -0.271920083 0.68763453 -0.343237331 -0.450134707

At3g22460 OASA2 (O-ACETYLSERINE (THIOL) LYASE (OAS-TL) ISOFORM A1); catalytic/ cysteine synthase/ pyridoxal phosphate binding -0.726247636 0.124080515 -1.883768426 -1.000254256 0.843943776 -0.731895581 -0.148228752 0.601397846

At3g29630 glycosyltransferase family protein -0.363039441 0.86595924 -0.130473637 -0.754556616 -0.184996671 0.23548986 -0.296702359 -0.856932246

At3g13390 sks11 (SKU5 Similar 11); copper ion binding / oxidoreductase 0.294840105 0.170285226 -0.102289533 -0.201491711 -0.145252829 0.019891595 6.064313678 0.404376737

At3g13490 ATKRS-2__OVA5 (OVULE ABORTION 5); ATP binding / aminoacyl-tRNA ligase/ lysine-tRNA ligase/ nucleic acid binding / nucleotide binding 0.001623202 -0.57773774 0.425818438 0.542963186 0.379446462 0.342253977 -0.221992388 0.022956765

At3g13450 DIN4 (DARK INDUCIBLE 4); 3-methyl-2-oxobutanoate dehydrogenase (2-methylpropanoyl-transferring)/ catalytic 1.676545474 -1.187357374 -1.019997634 -3.089029846 1.37464159 -1.498017897 0.224567428 1.430024216

At3g13400 sks13 (SKU5 Similar 13); copper ion binding / oxidoreductase 1.193018182 -0.28937399 -0.852135369 -0.962446401 -0.598516735 0.651503652 7.153482453 0.187332291

At3g21070 ATNADK-1__NADK1 (NAD KINASE 1); NAD+ kinase/ NADH kinase/ calmodulin binding 0.341484794 0.55242712 0.047613519 1.088319683 -0.81096974 -1.504372574 -0.39974676 -0.002993944

At3g21110 ATPURC_PURC__PUR7 (PURIN 7); phosphoribosylaminoimidazolesuccinocarboxamide synthase 0.862235967 0.142982414 1.025640664 0.683104466 -1.004009704 0.773723361 -0.793306018 0.118123323

At3g21055 PSBTN (photosystem II subunit T) 0.118469001 -4.028421955 0.520800556 -0.367960025 -0.028632861 -0.221099172 -0.987783842 -0.694137627

At3g25830 "ATTPS-CIN (terpene synthase-like sequence-1,8-cineole); (E)-beta-ocimene synthase/ myrcene synthase" -1.523600221 2.762543742 -0.190702813 -2.683136291 -1.226794447 -0.040870825 -1.091261433 -0.355197299

At3g14150 "(S)-2-hydroxy-acid oxidase, peroxisomal, putative / glycolate oxidase, putative / short chain alpha-hydroxy acid oxidase, putative" 0.703771031 -0.068448024 -0.67276348 -0.419025668 1.353069267 0.133327038 -1.195819236 -0.938843449

At3g19710 BCAT4 (BRANCHED-CHAIN AMINOTRANSFERASE4); catalytic/ methionine-oxo-acid transaminase -6.140598655 0.208356251 0.657210524 -0.570867541 0.188001215 0.587243452 -0.420295325 0.157894162

At3g19270 CYP707A4; (+)-abscisic acid 8'-hydroxylase/ oxygen binding 1.12555647 -0.084163209 -0.183718823 0.626442823 -0.527810284 -0.417982958 0.125690131 0.033943523

At3g19160 PGA22__ATIPT8 (ATP/ADP ISOPENTENYLTRANSFERASES); adenylate dimethylallyltransferase -0.148039288 0.196108847 0.422824302 0.31757948 0.120813362 -0.295224331 0.334262842 -0.390936271

At3g15290 "3-hydroxybutyryl-CoA dehydrogenase, putative" 0.078741833 0.331713922 -0.269560607 -0.048509054 0.867701315 -0.509861679 0.60937338 -0.604034832

At3g18220 phosphatidic acid phosphatase family protein / PAP2 family protein -0.347290725 0.591709625 0.377427522 0.564916931 0.270857397 -0.399226096 3.723487003 -0.297296317

At3g20160 "geranylgeranyl pyrophosphate synthase, putative / GGPP synthetase, putative / farnesyltranstransferase, putative" -0.24271855 0.553169843 0.304775603 -0.226899405 0.065075227 -0.154634807 0.01312936 -0.053152794

At3g20040 HKL2__ATHXK4; ATP binding / fructokinase/ glucokinase/ hexokinase 0.447651658 0.838834129 0.614186823 0.53685744 -0.164456957 -0.135019004 0.797917856 0.253436261

At3g27300 G6PD5 (glucose-6-phosphate dehydrogenase 5); glucose-6-phosphate dehydrogenase -0.779874562 0.988249522 -1.169805487 0.013911839 2.79128538 1.323503962 -0.013655808 0.265712257

At3g24360 enoyl-CoA hydratase/isomerase family protein 0.907804863 0.171214947 0.189872508 -0.271860061 0.011036061 0.288337688 0.734293518 0.34892214

At3g23810 SAHH2 (S-ADENOSYL-L-HOMOCYSTEINE (SAH) HYDROLASE 2); adenosylhomocysteinase/ binding / catalytic -0.621673497 0.161225568 1.509700423 -0.174360456 -1.404174251 0.948770358 1.029158617 0.876354301

At3g27190 "uracil phosphoribosyltransferase, putative / UMP pyrophosphorylase, putative / UPRTase, putative" 0.673670603 1.483229296 0.08652301 -1.495777143 -0.333462818 0.243514467 -0.782278821 0.166728224

At3g13110 SAT-1_SAT-A_SAT-M_SAT3__ATSERAT2;2 (SERINE ACETYLTRANSFERASE 2;2); serine O-acetyltransferase -0.936353481 0.469906058 -0.26782787 0.954109108 -1.248635648 -0.152444199 0.022672627 1.572338244

At3g16520 UGT88A1__UDP-glucoronosyl/UDP-glucosyl transferase family protein -0.018091625 -2.310194566 -0.451879594 -1.422436307 -0.265375537 -0.200465961 -0.802569451 0.140918792

At3g15020 "malate dehydrogenase (NAD), mitochondrial, putative" -0.222144503 1.1197179 0.243911401 0.699087516 -0.118847694 1.576308979 1.169701084 0.012735193

At3g14930 HEME1; uroporphyrinogen decarboxylase -0.426047609 -1.414269206 1.637605789 -0.040134414 1.127714241 -0.680448579 -0.49704412 -0.368155598

At3g27820 ATMDAR4__MDAR4 (MONODEHYDROASCORBATE REDUCTASE 4); monodehydroascorbate reductase (NADH) -1.001354806 -0.684433966 -1.336815835 -0.675693243 0.106086257 1.084240281 -0.000765348 0.893696964

At3g24220 ATNCED6__NCED6 (NINE-CIS-EPOXYCAROTENOID DIOXYGENASE 6); 9-cis-epoxycarotenoid dioxygenase -0.196626197 0.582478371 0.363142455 0.370147014 0.069524353 -0.022989919 0.710864165 -3.52022714

At3g24130 pectinesterase family protein 0.151183749 0.345754343 0.334754695 0.238503591 0.263374913 -0.379067789 0.427947494 -0.330349066

At3g24170 ATGR1 (glutathione-disulfide reductase); FAD binding / NADP or NADPH binding / glutathione-disulfide reductase/ oxidoreductase -0.04238218 0.020917319 -1.577492365 0.19108724 -2.971962885 0.867854836 0.146333613 0.699806955

At3g14440 ATNCED3_SIS7_STO1__NCED3 (NINE-CIS-EPOXYCAROTENOID DIOXYGENASE 3); 9-cis-epoxycarotenoid dioxygenase 0.53858139 -0.03790569 -2.19075825 1.976869305 -0.441600277 -1.589477306 0.16259213 0.012444943

At3g30775 AT-POX_ATPDH_ATPOX_PRO1_PRODH__ERD5 (EARLY RESPONSIVE TO DEHYDRATION 5); proline dehydrogenase 2.859181153 -0.060269899 0.540010858 -2.284457834 -2.114956149 -3.899528955 0.016727475 -0.510501316

At3g25810 "myrcene/ocimene synthase, putative" -0.142321435 0.336216861 -0.121164751 -0.026511964 0.116163535 -0.273249604 1.066737979 -0.656129404

At3g25820 "ATTPS-CIN (terpene synthase-like sequence-1,8-cineole); (E)-beta-ocimene synthase/ myrcene synthase" -0.967662315 1.710687696 0.013873484 -1.737768624 -0.960447036 0.318126955 -0.555113645 0.927694763

At3g25760 ERD12__AOC1 (ALLENE OXIDE CYCLASE 1); allene-oxide cyclase -3.478845533 -1.758150925 -0.297786176 5.065999767 2.719329268 -2.383924732 -1.312545714 1.525612371

At3g25780 AOC3 (ALLENE OXIDE CYCLASE 3); allene-oxide cyclase -0.07476072 1.372082632 0.49361652 2.949782541 -2.025691004 -3.640259883 -1.090886963 1.094912132

At3g20440 BE1_EMB2729__alpha-amylase/ catalytic/ cation binding 0.652683325 0.239781411 1.092825309 0.985712873 0.903060209 0.14310979 -0.432851667 -0.121850306

At3g20330 "aspartate carabmoyltransferase, chloroplast / aspartate transcarbamylase / ATCase (PYRB)" 0.302801271 0.375762917 0.45511531 1.228709557 0.167235771 0.838555153 -0.843077401 0.380066732

At3g12780 PGK1 (PHOSPHOGLYCERATE KINASE 1); phosphoglycerate kinase -0.808028643 -1.986363118 0.212925901 0.058885007 0.298788097 0.313802883 -0.260237658 0.170517919

At3g12670 emb2742 (embryo defective 2742); CTP synthase/ catalytic 0.495787205 0.470730569 0.581632394 0.391380816 -0.213618124 0.755744778 -1.612316231 -1.896711478

At3g27380 SDH2-1; electron carrier/ succinate dehydrogenase 0.271172009 0.711946604 -0.811345562 0.310747306 -0.424249103 0.617616724 0.392357493 0.822214689

At3g27440 "uracil phosphoribosyltransferase, putative / UMP pyrophosphorylase, putative / UPRTase, putative" -0.06304979 0.84844314 0.520022311 0.876929435 0.509615275 -0.487404348 1.747572445 -1.79738979

At3g29200 ATCM1__CM1 (CHORISMATE MUTASE 1); L-ascorbate peroxidase/ chorismate mutase 0.315131418 0.056318261 -0.380896621 -0.16089856 -1.995306325 0.883688589 -0.438420313 -0.561094594

At3g26650 GAPA-1__GAPA (GLYCERALDEHYDE 3-PHOSPHATE DEHYDROGENASE A SUBUNIT); glyceraldehyde-3-phosphate dehydrogenase/ protein binding 0.726346025 -3.410731008 -0.612268451 -0.376680888 -0.493913526 0.361693086 -0.955820256 0.338139679

At3g27060 TSO2 (TSO meaning 'ugly' in Chinese); oxidoreductase/ ribonucleoside-diphosphate reductase/ transition metal ion binding 0.673387749 0.82878101 2.117139602 1.411738069 0.136731495 1.20504385 -0.014922138 0.945556148

At3g25110 AtFaTA (Arabidopsis FatA acyl-ACP thioesterase); acyl carrier/ acyl-[acyl-carrier-protein] hydrolase 0.669285703 1.062431826 0.856984494 -0.744190223 -0.060431331 1.1454022 -0.55470468 -1.037027596

At3g25140 "GAUT8__QUA1 (QUASIMODO 1); polygalacturonate 4-alpha-galacturonosyltransferase/ transferase, transferring glycosyl groups / transferase, transferring hexosyl groups" 0.33094123 0.25051203 0.764047591 -0.200511154 -0.00243439 0.122627696 -0.181134211 -0.080206511

At3g17720 pyridoxal-dependent decarboxylase family protein 0.140975486 0.828593411 0.82211075 0.942926041 0.204120045 -0.229487717 2.666830979 -0.130755135

At3g16910 ACN1__AAE7 (ACYL-ACTIVATING ENZYME 7); AMP binding / acetate-CoA ligase -1.4526964 -0.114878601 -1.143028747 -0.079396145 -0.623264976 1.374540616 -0.218023963 -0.065572349

At3g17060 pectinesterase family protein -0.292653773 0.486214175 0.097398151 0.026237537 0.273692985 -0.308509583 5.080030894 -0.075905293

At3g18030 HAL3_HAL3A__ATHAL3A; FMN binding / phosphopantothenoylcysteine decarboxylase 1.202001123 0.259698792 0.296217602 -0.286916228 0.440064646 -0.055181046 -1.059057398 -1.144684923

At3g25585 "AAPT2 (AMINOALCOHOLPHOSPHOTRANSFERASE); phosphatidyltransferase/ phosphotransferase, for other substituted phosphate groups" -0.51031252 0.920425378 -0.421621799 -0.046179467 -1.774017969 0.582334915 0.430284725 -0.357511255

At3g21790 UDP-glucoronosyl/UDP-glucosyl transferase family protein -0.857642371 -1.041476049 -1.732512972 0.632473361 0.45296078 0.114292229 -0.378922011 -0.993547476

At3g21800 "UGT71B8 (UDP-GLUCOSYL TRANSFERASE 71B8); UDP-glycosyltransferase/ quercetin 3-O-glucosyltransferase/ quercetin 4'-O-glucosyltransferase/ transferase, transferring glycosyl groups" 0.029177236 0.219471371 0.095791563 0.070689033 0.170730429 -0.197687086 -0.175496073 -1.142041045

At3g21720 ICL (ISOCITRATE LYASE); catalytic/ isocitrate lyase -0.00063683 0.406010316 -0.264202046 -0.393006041 0.007950496 -0.422672451 -1.050915359 -4.654789072

At3g21750 "UGT71B1 (UDP-GLUCOSYL TRANSFERASE 71B1); UDP-glycosyltransferase/ quercetin 3-O-glucosyltransferase/ transferase, transferring glycosyl groups" 0.851822348 -0.867595556 -1.673441972 1.571843187 0.16754198 2.640457574 0.119598344 0.432212622

At3g21780 "UGT71B6 (UDP-glucosyl transferase 71B6); UDP-glycosyltransferase/ abscisic acid glucosyltransferase/ transferase, transferring glycosyl groups" 0.04121978 0.016472339 -0.982254317 1.342527851 -0.090864036 -1.096483816 -0.414338068 0.166789202

At3g21760 UDP-glucoronosyl/UDP-glucosyl transferase family protein -0.199545635 -1.149741893 -0.637094332 0.804755332 0.407423156 1.875484937 0.164080434 -1.441160729

At3g25570 adenosylmethionine decarboxylase family protein -1.278987847 -0.28167284 -0.673623857 0.118424358 -0.374292332 -0.231582592 0.958614815 0.957352916

At3g19450 CAD_CAD-C_CAD4__ATCAD4; cinnamyl-alcohol dehydrogenase -1.486140887 0.198288943 0.803291039 -1.739282164 -3.202229297 1.373560567 -1.183807501 0.552788836

At3g19480 "D-3-phosphoglycerate dehydrogenase, putative / 3-PGDH, putative" -2.042085996 -2.176968211 0.819510347 0.074709021 -0.718849563 -0.014543085 0.504387786 -0.018964725

At3g21230 4CL5 (4-coumarate:CoA ligase 5); 4-coumarate-CoA ligase -0.920833786 0.790688499 -1.350961507 0.032826568 -2.628102456 -0.779171491 0.265511931 -0.306478986

At3g21240 AT4CL2__4CL2 (4-COUMARATE:COA LIGASE 2); 4-coumarate-CoA ligase -0.121588516 1.349927853 -0.616266916 -0.840608661 -1.03388778 0.706863136 -0.393913736 -0.140987513

At3g25960 "pyruvate kinase, putative" 0.039839997 0.218346581 0.212246114 0.237081522 0.278133106 -0.39312419 0.140225216 -0.377843668

At3g25900 ATHMT-1__HMT-1; homocysteine S-methyltransferase 0.508135431 1.736720621 1.63377007 1.506457315 -0.665704142 -2.302474656 1.14672566 0.439170063

At3g25860 PLE2__LTA2; dihydrolipoyllysine-residue acetyltransferase -0.298207843 -0.073510823 1.905773745 -1.033763849 -0.194307876 0.515333419 -0.540903641 0.028999775

At3g23630 "ATIPT7; ATP binding / tRNA isopentenyltransferase/ transferase, transferring alkyl or aryl (other than methyl) groups" -0.455641085 0.788534711 0.137681382 0.16728964 0.118771555 -0.329081238 0.116505586 -0.091621136

At3g23580 RNR2A (RIBONUCLEOTIDE REDUCTASE 2A); ribonucleoside-diphosphate reductase 0.528999332 -0.268854142 0.129460397 0.968217862 -0.451155988 0.589206404 -0.189466459 -0.61893547

At3g23640 "HGL1 (heteroglycan glucosidase 1); hydrolase, hydrolyzing O-glycosyl compounds" 0.592036532 -0.198118114 -0.292062967 -1.567602162 0.631947126 0.308427283 -0.966787354 -0.399356592

At3g14530 "geranylgeranyl pyrophosphate synthase, putative / GGPP synthetase, putative / farnesyltranstransferase, putative" 0.003260861 0.916025942 0.746802934 -0.629993238 -0.146848359 0.119735069 0.180086962 0.270123164

At3g24503 ALDH1A_REF1__ALDH2C4; 3-chloroallyl aldehyde dehydrogenase/ aldehyde dehydrogenase (NAD)/ coniferyl-aldehyde dehydrogenase -2.267979431 0.791832813 -0.995503129 0.489254559 0.655966492 0.277118042 -1.736613802 -0.225158726

At3g17820 GLN1.3_GLN1;3__ATGSKB6; copper ion binding / glutamate-ammonia ligase 1.74236879 0.84816861 -1.050200309 -0.249547509 0.545425613 0.886448725 -0.033068802 0.119234035

At3g17810 dihydroorotate dehydrogenase family protein / dihydroorotate oxidase family protein 0.180567602 0.384928045 -2.723550542 -0.952920471 -0.217207472 -0.05171665 -0.260606396 -0.217987799

At3g21560 UGT84A2; UDP-glycosyltransferase/ sinapate 1-glucosyltransferase -0.896255888 0.740665822 -0.712713696 2.179982344 -1.945738916 2.957220757 -2.074096649 -2.68333092

At3g21500 DXPS1__1-deoxy-D-xylulose-5-phosphate synthase 0.226055752 -0.121811223 -1.423939794 0.322291682 -0.344500166 0.031605063 0.38614534 0.199068288

At3g29090 pectinesterase family protein 0.549796633 0.035541561 -0.31572111 0.162866787 0.579086001 0.535349713 -0.785023037 -0.912525957

At3g13930 "dihydrolipoamide S-acetyltransferase, putative" -0.575796213 0.683250092 0.338073828 0.317353828 -0.06629008 0.778631065 0.296082632 0.971419592

At3g18000 NMT1_PEAMT__XPL1 (XIPOTL 1); methyltransferase/ phosphoethanolamine N-methyltransferase 1.627149402 0.688424554 1.898999253 0.090196889 2.006650491 1.505187369 -0.353786184 1.638845961

At3g15730 PLDALPHA1 (PHOSPHOLIPASE D ALPHA 1); phospholipase D 0.424395413 0.120721419 -0.502897259 -0.381515575 0.524447948 0.158800756 -0.902995371 0.382936683

At3g27620 AOX1C; alternative oxidase 0.117485155 0.343706426 0.361781096 0.726558497 0.269395933 -0.158428449 0.680632473 -0.251343794

At3g27740 CARA (CARBAMOYL PHOSPHATE SYNTHETASE A); carbamoyl-phosphate synthase (glutamine-hydrolyzing)/ carbamoyl-phosphate synthase/ catalytic 0.688205767 0.197508274 0.914091299 0.583108024 -0.308441167 0.653665206 -1.121280976 0.497920758

At3g15850 ADS3_FADB_JB67__FAD5 (FATTY ACID DESATURASE 5); 16:0 monogalactosyldiacylglycerol desaturase/ oxidoreductase -0.889361962 -2.024718767 1.487409637 -0.252341163 -0.805989653 -0.185604985 0.154603136 -0.84022925

At3g15720 glycoside hydrolase family 28 protein / polygalacturonase (pectinase) family protein 1.12832438 0.230578262 0.623371733 0.120918468 1.265649936 1.722322358 -0.591480661 0.977318856

At3g15870 oxidoreductase 0.17502773 0.202117102 0.393990549 0.265598083 0.410447565 -0.292083807 0.415961561 -0.379781252

At3g15640 cytochrome c oxidase family protein 0.148191372 0.687502811 0.493494062 0.269082681 0.232029355 0.399925438 0.120385679 0.385859964

At3g26830 CYP71B15__PAD3 (PHYTOALEXIN DEFICIENT 3); dihydrocamalexic acid decarboxylase/ monooxygenase/ oxygen binding -0.117068971 1.072070293 -3.904336423 4.269366801 -0.318776529 -0.886046078 -2.249709322 2.152887213

At3g16140 PSAH-1 (photosystem I subunit H-1) 0.208311304 -3.367851997 0.114133249 -0.431268319 0.012453421 -0.589669878 -0.534877136 -0.239811703

At3g22740 HMT3; homocysteine S-methyltransferase -1.385672642 0.010442651 -0.974641266 0.364340427 0.880004072 -0.905612041 2.343446984 -1.15350444

At3g16150 "L-asparaginase, putative / L-asparagine amidohydrolase, putative" 2.652886321 0.243627069 0.294952898 -0.597976812 -2.49671259 -0.739547049 1.402402084 1.291716964

At3g14415 "(S)-2-hydroxy-acid oxidase, peroxisomal, putative / glycolate oxidase, putative / short chain alpha-hydroxy acid oxidase, putative" -1.359950446 -2.797699788 -0.093637476 0.165240216 -0.407062728 0.219687932 -0.221794272 0.946648641

At3g14300 ATPMEPCRC; pectinesterase 0.04439692 0.312148973 0.274025056 0.426230237 0.287521844 -0.373087055 0.258590673 -0.369442429

At3g14390 "diaminopimelate decarboxylase, putative / DAP carboxylase, putative" 0.311505801 0.012330378 1.159772152 -0.021124256 -0.262921328 0.65555574 -1.043935539 0.802321294

At3g14310 ATPME3; pectinesterase -1.069383447 0.589718583 0.895088077 -0.125646216 3.340438664 0.159098101 -0.600456039 2.825542438

At3g14360 lipase class 3 family protein 0.093339523 0.942729642 -0.510879287 -0.037770186 0.513535741 0.775889613 -0.386038001 -2.961917473

At3g17390 MAT4_SAMS3__MTO3 (METHIONINE OVER-ACCUMULATOR 3); methionine adenosyltransferase -0.247060793 0.163218134 1.127273644 -0.123361423 -0.855242415 -0.270662411 -0.881344319 0.896573486

At3g16785 PLD ZETA 1_PLDZ1_PLDZETA1__PLDP1 (PHOSPHOLIPASE D P1); phospholipase D -0.016873527 0.282363983 -0.649874628 0.265976312 0.475690524 -0.448191711 0.137442544 -0.101132408

At3g17240 mtLPD2 (LIPOAMIDE DEHYDROGENASE 2); ATP binding / dihydrolipoyl dehydrogenase -0.200033744 0.924995047 -0.660622368 1.082550682 0.256102446 0.441366192 -0.347522889 0.183858363

At3g22400 "LOX5; electron carrier/ iron ion binding / lipoxygenase/ metal ion binding / oxidoreductase, acting on single donors with incorporation of molecular oxygen, incorporation of two atoms of oxygen" -0.368790983 0.725320013 0.155586456 -0.483982349 -0.061519911 -0.539621487 -0.857058976 -2.398724279

At3g22360 AOX1B; alternative oxidase -0.066707621 0.660693503 0.349910944 0.977823872 0.250014104 -0.55896489 1.599000725 -0.240326193

At3g22370 ATAOX1A__AOX1A (ALTERNATIVE OXIDASE 1A); alternative oxidase -0.927884028 -0.023907224 -2.656798104 1.233825698 -0.135406913 -1.074771269 0.194162039 1.401071852

At3g02610 acyl-[acyl-carrier-protein] desaturase/ oxidoreductase/ transition metal ion binding 0.168942577 0.890932572 0.062379794 -0.451326409 0.328538873 -0.007799579 -0.708311252 -2.237874933

At3g02600 ATLPP3__LPP3 (LIPID PHOSPHATE PHOSPHATASE 3); phosphatidate phosphatase 0.887952398 -0.122353703 0.279126262 -0.405798702 0.298535839 0.256923629 -0.290032258 -0.184146232

At3g02570 PMI1__MEE31 (MATERNAL EFFECT EMBRYO ARREST 31); mannose-6-phosphate isomerase -0.469006806 0.047820576 0.839342151 1.489480999 0.831944623 -0.264444889 0.721487395 -0.688191738

At3g02630 "acyl-(acyl-carrier-protein) desaturase, putative / stearoyl-ACP desaturase, putative" 0.263842173 0.002694349 0.009972893 -1.393597536 1.830177828 1.368966008 -0.889444415 -0.1877505

At3g02470 SAMDC (S-ADENOSYLMETHIONINE DECARBOXYLASE); adenosylmethionine decarboxylase 0.236446921 0.033917867 -0.224790927 -0.631067885 -1.223291437 -0.112767879 0.085666505 -0.057271091

At3g06500 "beta-fructofuranosidase, putative / invertase, putative / saccharase, putative / beta-fructosidase, putative" 0.837810997 -0.259939434 -1.046750589 0.850647921 0.717729741 -2.724494002 -0.167663791 0.50399149

At3g06650 ACLB-1; ATP citrate synthase 0.926272111 -0.124653789 1.033038389 -0.92530438 -0.682704853 0.371877443 -0.410256915 0.564931929

At3g06580 GAL1__GALK; ATP binding / galactokinase 0.379761623 0.151935974 -0.350431069 -0.322243355 1.106993473 0.423523541 0.074658416 0.343643832

At3g06830 pectinesterase family protein -0.251631174 0.814877492 0.621459091 0.574600269 0.41645719 -0.534762256 3.939394945 -0.01410564

At3g06810 IBR3 (IBA-RESPONSE 3); acyl-CoA dehydrogenase/ oxidoreductase 0.068459605 0.340153451 -1.727093239 0.3783877 0.398324076 0.22695757 0.419664117 -0.539899253

At3g06850 DIN3_LTA1__BCE2; acetyltransferase/ alpha-ketoacid dehydrogenase/ dihydrolipoamide branched chain acyltransferase 1.781203805 -0.454266386 -0.561050265 -2.338523262 0.540616544 -1.300059041 -0.311020829 0.330303231

At3g06860 ATMFP2__MFP2 (MULTIFUNCTIONAL PROTEIN 2); 3-hydroxyacyl-CoA dehydrogenase/ enoyl-CoA hydratase 0.706858226 0.602070393 -1.761746709 0.231844408 0.779586126 1.026381245 0.042744933 -0.230425285

At3g05970 LACS6 (long-chain acyl-CoA synthetase 6); long-chain-fatty-acid-CoA ligase -0.502928256 0.485786836 -1.314450226 0.507214326 1.865706667 0.88645602 0.231312431 0.383512308

At3g04120 GAPC-1__GAPC1 (GLYCERALDEHYDE-3-PHOSPHATE DEHYDROGENASE C SUBUNIT 1); glyceraldehyde-3-phosphate dehydrogenase (phosphorylating)/ glyceraldehyde-3-phosphate dehydrogenase 0.180356659 0.452909948 -0.142969742 -0.251861443 -0.65405219 0.522003165 -0.272413395 1.331881205

At3g04390 xanthine dehydrogenase family protein 0.074491754 0.169646246 -0.060447935 0.181587451 0.529204334 -0.144929169 0.020091873 -0.329334872

At3g04520 THA2 (Threonine Aldolase 2); threonine aldolase 0.417342674 -0.345133212 -1.216612405 0.268965691 0.152215429 0.685021442 -0.328919665 0.403974213

At3g02760 ATP binding / aminoacyl-tRNA ligase/ histidine-tRNA ligase/ nucleotide binding 0.985105366 0.330290478 0.684873569 0.186568272 -0.167910322 0.452050322 -1.201705187 0.292190559

At3g02875 ILR1 (IAA-LEUCINE RESISTANT 1); IAA-Leu conjugate hydrolase/ IAA-Phe conjugate hydrolase/ metallopeptidase 1.622014514 0.038796691 -1.996263652 -0.207943837 -0.213214387 0.09003707 -0.053612887 -0.922586511

At3g02870 "VTC4; 3'(2'),5'-bisphosphate nucleotidase/ L-galactose-1-phosphate phosphatase/ inositol or phosphatidylinositol phosphatase/ inositol-1(or 4)-monophosphatase" -0.085348947 -0.516253774 1.120035482 -0.766811594 -1.285662478 0.619486167 0.160245011 1.016533024

At3g02780 IDI2_IPIAT1__IPP2 (ISOPENTENYL PYROPHOSPHATE:DIMETHYLALLYL PYROPHOSPHATE ISOMERASE 2); isopentenyl-diphosphate delta-isomerase -0.251128896 0.560128369 -0.161313233 -0.825642723 -0.100759318 0.023256746 -0.343730799 0.110921992

At3g09820 ADK1 (adenosine kinase 1); adenosine kinase/ copper ion binding 0.099060294 0.599562976 1.533291161 -0.106265053 -0.271579576 0.716207246 0.21597314 1.076603768

At3g08590 "2,3-biphosphoglycerate-independent phosphoglycerate mutase, putative / phosphoglyceromutase, putative" 0.025092032 0.253690085 -0.788126155 0.85299027 -0.845661628 1.419144525 -0.088126517 0.917091572

At3g08610 unknown protein -0.116361045 0.28023214 0.146166934 -0.181240195 0.273235854 0.349448082 -0.017205093 0.510535212

At3g09640 APX1B__APX2 (ASCORBATE PEROXIDASE 2); L-ascorbate peroxidase 0.45295529 -0.017146253 -0.438474309 0.463123302 0.595511312 -0.892905139 0.134267431 -0.960983613

At3g05820 beta-fructofuranosidase/ catalytic 0.005154058 0.595309812 0.612655294 0.862072804 0.300688295 -0.757432734 2.635876413 -0.424408449

At3g10720 "pectinesterase, putative" -0.072181338 -0.659807685 3.557318825 -0.104567656 -1.536376488 -4.040064742 -0.291664608 2.00233393

At3g10710 pectinesterase family protein -0.917188553 1.518463629 0.452296626 -1.33732527 -0.274985985 0.295815031 0.218883026 0.410428736

At3g10700 GHMP kinase family protein 0.315552374 0.124198403 0.91570963 -0.028385486 0.199714271 -0.152401767 0.06964762 0.158958991

At3g10870 "ATMES17__MES17 (METHYL ESTERASE 17); hydrolase/ hydrolase, acting on ester bonds / methyl indole-3-acetate esterase" 1.38347698 -0.095953803 0.74718873 0.558034052 1.497011928 -0.162212207 -0.095483101 0.054755406

At3g10850 GLX2-2__GLY2; hydroxyacylglutathione hydrolase 0.457301694 -0.41222088 -0.187256384 -0.726861874 1.29837195 0.416129705 -0.705367569 0.259913563

At3g11750 "dihydroneopterin aldolase, putative" -1.127250946 -0.701857292 0.407834503 0.019736092 0.163648029 -0.239048484 0.090122258 -0.141019501

At3g04050 "pyruvate kinase, putative" -0.018391569 0.273966223 0.26033494 0.541274528 0.384342045 -0.511512652 0.176677057 -0.566264394

At3g04600 tRNA synthetase class I (W and Y) family protein 0.330503307 0.279631916 0.316196071 0.448938591 -0.083754537 0.390337295 -0.624582458 0.352101331

At3g07130 ATPAP15__PAP15 (PURPLE ACID PHOSPHATASE 15); acid phosphatase/ protein serine/threonine phosphatase -1.41718019 1.237894099 0.501824959 -1.591713706 -0.374409696 1.016485508 -0.045283617 -0.330448055

At3g07270 GTP cyclohydrolase I -1.128095096 0.597061227 1.253863507 0.730291268 -0.247433319 -0.378491745 0.450711967 0.856249478

At3g03100 NADH:ubiquinone oxidoreductase family protein 0.005211986 0.552768508 -0.094730709 -0.313974626 0.258434015 0.136902474 -0.019396251 0.08149726

At3g03250 UGP (UDP-glucose pyrophosphorylase); UTP:glucose-1-phosphate uridylyltransferase/ nucleotidyltransferase -0.995114598 0.504799652 0.358826431 0.219898617 -0.451106598 1.25246671 1.037112444 0.943020578

At3g03050 "ATCSLD3_KJK__CSLD3 (CELLULOSE SYNTHASE-LIKE D3); cellulose synthase/ transferase, transferring glycosyl groups" 0.162953046 0.913704001 0.749597863 0.195078916 -0.971496137 -1.162554514 -0.419769519 0.50354624

At3g03190 ATGSTF6__ATGSTF11 (GLUTATHIONE S-TRANSFERASE F11); glutathione transferase -3.033457269 0.661831847 1.522047441 -0.14541464 -0.030793926 0.761501463 0.170080217 0.703491407

At3g02100 UDP-glucoronosyl/UDP-glucosyl transferase family protein -0.134540838 0.542362754 0.340128419 0.404816834 0.519595797 -0.568035792 0.169622247 -0.724686576

At3g10050 OMR1 (L-O-METHYLTHREONINE RESISTANT 1); L-threonine ammonia-lyase 0.629289144 0.159869362 1.146833853 0.898203999 -0.871580532 0.231812763 -0.861976425 0.150276363

At3g05630 PDLZ2__PLDP2; phospholipase D -0.172663808 0.2196289 -2.151620735 0.407619111 -0.059473907 0.060449682 -0.134949995 -1.587367635

At3g05620 pectinesterase family protein -0.044811154 0.366238399 0.381275453 0.240973746 0.189532836 -0.403009854 0.486671643 -0.034930134

At3g05610 pectinesterase family protein -0.186008256 0.485397059 -0.053141782 0.097027424 0.153024958 -0.13470631 5.489519502 0.170643492

At3g06350 EMB3004__MEE32 (MATERNAL EFFECT EMBRYO ARREST 32); 3-dehydroquinate dehydratase/ NADP or NADPH binding / binding / catalytic/ shikimate 5-dehydrogenase 0.354529488 0.498178869 0.203111008 0.153220481 -0.845346667 0.209230876 -0.240742331 0.651836835

At3g10160 ATDFC__DFC (DHFS-FPGS HOMOLOG C); tetrahydrofolylpolyglutamate synthase -0.272738939 -0.549867756 0.831912801 -0.08154675 0.3160154 0.505791006 0.059143374 0.147846315

At3g09940 AtMDAR3__MDHAR (MONODEHYDROASCORBATE REDUCTASE); monodehydroascorbate reductase (NADH) -1.186605968 2.247415958 -0.154945793 1.727392638 -1.170453204 -2.638388927 -0.854627048 1.63172851

At3g10390 FLD (FLOWERING LOCUS D); amine oxidase/ electron carrier/ oxidoreductase 0.826761314 0.210455662 0.131687331 0.307962011 0.389348057 0.032979044 -0.1019658 -0.483413632

At3g01910 AT-SO_AtSO__SOX (SULFITE OXIDASE); sulfite oxidase -0.207845876 -0.079481052 -0.242323349 -0.35557727 0.148693997 0.438728025 0.265343154 0.218994119

At3g01440 oxygen evolving enhancer 3 (PsbQ) family protein -2.224047001 -1.703243671 1.454195468 1.405836389 0.937302025 1.183059223 0.600352665 -0.252851978

At3g01420 ALPHA-DOX1__DOX1; lipoxygenase -0.797213091 1.808934688 -3.166628103 -2.861099831 -1.414747955 -0.146754069 -0.635689311 -0.996638879

At3g02020 AK3 (ASPARTATE KINASE 3); aspartate kinase -1.625923396 -0.693526419 1.38388297 -0.577003632 -0.619165348 0.633446144 -0.649158742 0.62267941

At3g01820 adenylate kinase family protein -0.245277873 0.705468726 0.504625175 1.853407112 -0.612302206 -0.767466845 0.326177647 -0.15853711

At3g01850 "ribulose-phosphate 3-epimerase, cytosolic, putative / pentose-5-phosphate 3-epimerase, putative" 0.227661018 -0.13549459 -0.538805344 -0.425185432 0.944349322 -0.546846808 -0.347855852 -0.286760275

At3g07420 ATNS2_SYNC2_SYNC2_ARATH__NS2; asparagine-tRNA ligase -0.225844872 0.554280823 0.55824488 -0.222779461 0.432243402 -0.409584169 0.190382306 0.443836684

At3g03310 lecithin:cholesterol acyltransferase family protein / LACT family protein 0.765123031 0.521015208 -1.492848507 -0.140873062 0.059269356 -0.141834178 -1.069670605 -1.407203899

At3g11710 ATKRS-1 (ARABIDOPSIS THALIANA LYSYL-TRNA SYNTHETASE 1); ATP binding / aminoacyl-tRNA ligase/ lysine-tRNA ligase/ nucleic acid binding / nucleotide binding 1.014654752 0.281087549 0.566155437 0.596186885 -0.437969838 0.361144076 -1.025842427 0.296597789

At3g11670 "DGD1 (DIGALACTOSYL DIACYLGLYCEROL DEFICIENT 1); UDP-galactosyltransferase/ galactolipid galactosyltransferase/ transferase, transferring glycosyl groups" -0.028352805 -0.941542213 -0.384030494 0.016764764 -1.222697671 0.236617621 0.436794701 -0.717823398

At3g04870 "PDE181__ZDS (ZETA-CAROTENE DESATURASE); carotene 7,8-desaturase" -0.372094622 -1.234529955 -0.705089756 0.057752094 -0.87661044 0.447913252 -0.035893588 -0.284887998

At3g04940 ATCYSD1__CYSD1 (CYSTEINE SYNTHASE D1); cysteine synthase 0.251281513 -0.254841277 0.874178079 -0.897530372 0.170784038 0.345572289 -0.603708763 0.759567646

At3g04790 ribose 5-phosphate isomerase-related -1.152686571 -1.190308443 0.884270898 0.270642438 -0.295258746 0.732288619 -0.311575858 0.693803804

At3g10230 LYC (LYCOPENE CYCLASE); lycopene beta cyclase -0.859662409 -1.078878471 -0.031089003 -1.071852638 -0.51849033 0.209818418 0.179124546 0.034579173

At3g10370 SDP6 (SUGAR-DEPENDENT 6); glycerol-3-phosphate dehydrogenase 0.336086813 0.545280862 -0.42856301 0.763807781 0.487974057 0.310544837 0.795138392 0.072207998

At3g10340 PAL4 (Phenylalanine ammonia-lyase 4); ammonia ligase/ ammonia-lyase/ catalytic 0.600048229 0.519990111 -1.857730358 -3.579519738 -1.61340446 0.519580337 1.292441978 -1.707235194

At3g01500 ATBCA1_SABP3__CA1 (CARBONIC ANHYDRASE 1); carbonate dehydratase/ zinc ion binding -1.225600495 -4.881269335 0.556752292 1.022024783 -0.4038663 -0.643707934 0.298274002 1.579319018

At3g03520 phosphoesterase family protein -1.986389249 1.881163601 -0.625443252 -0.709976716 0.352627112 -0.487958129 -1.381418339 -0.822257489

At3g03630 CS26; cysteine synthase 0.349016224 -0.766293075 1.706078748 0.990071999 0.469972292 -0.151825656 -0.732140811 0.09197702

At3g03540 "NPC5 (NON-SPECIFIC PHOSPHOLIPASE C5); hydrolase, acting on ester bonds / phospholipase C" -0.19650912 0.970906952 -0.646870036 -0.36153737 -0.75346806 0.554703264 0.531583392 -1.883918519

At3g07630 ADT2 (arogenate dehydratase 2); arogenate dehydratase/ prephenate dehydratase 0.112374079 0.250068325 0.044780935 0.437891042 0.67352327 0.516316824 -0.616307744 -0.257711881

At3g11430 ATGPAT5__GPAT5 (GLYCEROL-3-PHOSPHATE ACYLTRANSFERASE 5); 1-acylglycerol-3-phosphate O-acyltransferase/ acyltransferase/ organic anion transmembrane transporter 0.133823974 1.349403884 -1.081725938 -1.746467529 -0.674761411 -0.041722483 1.587352405 -1.239458411

At3g11480 BSMT1; S-adenosylmethionine-dependent methyltransferase 2.352476701 -0.803030353 -2.277081218 0.860478872 -2.042882369 -0.373539664 2.288502958 1.749462879

At3g01040 "GAUT13 (Galacturonosyltransferase 13); polygalacturonate 4-alpha-galacturonosyltransferase/ transferase, transferring glycosyl groups / transferase, transferring hexosyl groups" -0.40312452 0.774859054 0.14893462 1.291271783 -0.464125901 -0.427839339 1.295458102 0.404731435

At3g03900 "adenylylsulfate kinase, putative" 0.089172578 0.597512393 0.630660708 1.005056477 -0.232981664 0.209775163 1.495018086 -0.982818867

At3g03780 AtMS2; 5-methyltetrahydropteroyltriglutamate-homocysteine S-methyltransferase/ methionine synthase -0.497741792 0.487619613 1.173509839 -0.331439105 -0.470651144 0.317076738 0.053812234 0.922819562

At3g03910 "GDH3 (GLUTAMATE DEHYDROGENASE 3); binding / catalytic/ oxidoreductase/ oxidoreductase, acting on the CH-NH2 group of donors, NAD or NADP as acceptor" -0.06697383 0.393974432 0.409764579 0.230917948 -0.740734777 0.119104449 1.29214735 0.096178797

At1g13440 GAPC-2__GAPC2 (GLYCERALDEHYDE-3-PHOSPHATE DEHYDROGENASE C2); NAD or NADH binding / binding / catalytic/ glyceraldehyde-3-phosphate dehydrogenase (phosphorylating)/ glyceraldehyde-3-phosphate dehydrogenase -0.125476012 -0.059221983 0.084400395 -0.672027737 -0.470557271 0.428179565 -0.512758212 0.612780414

At1g13280 AOC4 (ALLENE OXIDE CYCLASE 4); allene-oxide cyclase 1.723609769 -0.306386724 -0.039845961 -1.458122568 1.181381466 0.899301603 0.113836191 2.629220813

At3g16390 NSP3 (NITRILE SPECIFIER PROTEIN 3) -0.013820268 1.756856088 -0.062930824 -0.680636483 -0.427131232 -0.944716498 -3.449640277 1.853497054

At1g06350 fatty acid desaturase family protein 3.143350605 -1.495954151 2.629677444 0.552105266 -0.127856841 -0.41728961 -0.308411035 0.984337166

At1g17745 PGDH (3-PHOSPHOGLYCERATE DEHYDROGENASE); phosphoglycerate dehydrogenase 1.302264027 0.782731019 -3.499116964 0.78911691 0.003499051 2.890629347 -0.908804237 3.302986317

At1g02390 ATGPAT2__GPAT2 (GLYCEROL-3-PHOSPHATE ACYLTRANSFERASE 2); acyltransferase -0.36218664 -0.154800255 -1.633189021 0.657944654 -1.068153923 -0.205938648 0.164325869 -1.093462549

At1g06310 "ACX6 (ACYL-COA OXIDASE 6); FAD binding / acyl-CoA dehydrogenase/ acyl-CoA oxidase/ electron carrier/ oxidoreductase/ oxidoreductase, acting on the CH-CH group of donors" 0.192278901 0.446102469 0.432494976 0.300649278 0.185924921 -0.198970937 0.074854744 -0.332498049

At1g01600 CYP86A4; fatty acid (omega-1)-hydroxylase/ oxygen binding 2.53094084 -0.495168805 0.460820782 -0.755234733 -0.509644393 0.536405138 0.859721367 0.906813776

At1g01610 ATGPAT4__GPAT4 (GLYCEROL-3-PHOSPHATE ACYLTRANSFERASE 4); 1-acylglycerol-3-phosphate O-acyltransferase/ acyltransferase 0.889766813 -0.396551434 0.844259695 -1.22954183 -0.445996085 0.596425491 -0.912819024 -0.047612244

At1g01480 AT-ACC2__ACS2; 1-aminocyclopropane-1-carboxylate synthase 0.82668958 0.487586149 -1.866109764 1.981324811 -0.536522534 -1.696088869 -0.424265249 -1.742907156

At1g02400 ATGA2OX4_ATGA2OX6_DTA1__GA2OX6 (GIBBERELLIN 2-OXIDASE 6); gibberellin 2-beta-dioxygenase -0.336298655 0.977539327 -0.580565121 1.582627231 -1.416478067 -4.011372161 -0.349296935 -0.074517251

At1g44090 GA2OX5__ATGA20OX5 (ARABIDOPSIS THALIANA GIBBERELLIN 20-OXIDASE 5); gibberellin 20-oxidase -0.13491693 0.194084639 0.360689978 0.506302397 0.269909168 -0.654377196 0.39346158 -0.858750482

At1g15710 prephenate dehydrogenase family protein -0.09874545 0.295233028 0.91497375 0.604559479 -0.054713965 0.665985976 -0.135779565 -0.874189812

At1g15690 ATAVP3_AVP-3__AVP1; ATPase/ hydrogen-translocating pyrophosphatase 0.106485983 0.061019296 0.684211863 -0.239088143 0.408567888 0.473035011 -0.192453038 -0.364922623

At1g12520 ATCCS (COPPER CHAPERONE FOR SOD1); superoxide dismutase/ superoxide dismutase copper chaperone -0.134513818 -0.658334882 0.210912393 -0.349716978 0.259555387 -0.087757564 -0.189968128 0.385148881

At1g20630 CAT1 (CATALASE 1); catalase 0.999266812 -0.588448191 -1.557298389 -0.284753094 2.986533955 -0.782984749 1.631908867 -1.510256479

At1g20510 OPCL1 (OPC-8:0 COA LIGASE1); 4-coumarate-CoA ligase -0.314854896 -0.045462745 -0.038130066 2.248332898 -0.519439838 -2.840484629 -0.502511703 1.177159878

At5g02080 DNA/panthothenate metabolism flavoprotein family protein 0.2710817 0.158668011 -0.217561609 0.204642287 0.290307646 0.281199886 -0.103603602 0.144234646

At1g20575 "dolichyl-phosphate beta-D-mannosyltransferase, putative / dolichol-phosphate mannosyltransferase, putative / mannose-P-dolichol synthase, putative" 0.363674118 0.572013923 -0.2243008 -0.267300642 0.155056484 -0.1607603 -0.27228488 -0.107572736

At1g20620 SEN2__CAT3 (CATALASE 3); catalase -0.073236763 -0.81252291 -0.928785651 -0.394662458 2.835112633 -0.56371764 -2.174116226 -0.115754768

At1g28030 "oxidoreductase, 2OG-Fe(II) oxygenase family protein" -0.287557647 0.249491143 0.269253252 -0.076628529 -0.168169185 0.091393678 0.014822929 -2.830006808

At1g28130 GH3.17; indole-3-acetic acid amido synthetase 0.352093437 1.424984253 -0.681767289 -2.700402516 -1.650513571 -0.657224437 0.32174435 1.819022298

At1g56500 haloacid dehalogenase-like hydrolase family protein -1.62173873 -1.804834063 0.640942883 0.681779163 0.201412721 0.113706129 0.613193738 0.356270253

At1g47990 GA2OX4__ATGA2OX4 (ARABIDOPSIS THALIANA GIBBERELLIN 2-OXIDASE 4); gibberellin 2-beta-dioxygenase 0.28456654 0.39403648 0.43823291 0.624423227 0.398319105 -0.650048928 0.346295725 -0.77882783

At1g42970 GAPB (GLYCERALDEHYDE-3-PHOSPHATE DEHYDROGENASE B SUBUNIT); glyceraldehyde-3-phosphate dehydrogenase (NADP+)/ glyceraldehyde-3-phosphate dehydrogenase -0.730929459 -3.876594648 -0.09663783 0.174963013 -0.776382119 0.516064771 -0.189119431 0.548836952

At1g68890 2-oxoglutarate decarboxylase/ hydro-lyase/ magnesium ion binding / thiamin pyrophosphate binding -0.291559669 0.020788061 0.868242548 0.662512846 0.716887801 -0.17098894 0.219217526 -0.426453464

At1g55320 0.574053292 0.136424488 0.038790612 0.096584946 0.31931951 0.192092848 -0.090373858 -0.333185229

At1g55180 PLDALPHA4__PLDEPSILON (PHOSPHOLIPASE D ALPHA 4); phospholipase D -0.332447678 0.296327051 0.271056717 -0.121732126 0.112132322 -0.255779788 0.277310767 -0.271074713

At1g52340 ATABA2_GIN1_ISI4_SDR1_SIS4_SRE1__ABA2 (ABA DEFICIENT 2); alcohol dehydrogenase/ oxidoreductase/ xanthoxin dehydrogenase -0.084175427 -0.350197931 0.250111625 -1.54263633 -0.708515716 0.39458533 -0.391134956 -0.405840333

At1g77760 GNR1_NR1__NIA1 (NITRATE REDUCTASE 1); nitrate reductase -2.298749803 0.200994823 -0.032728779 0.431316908 0.258211808 -1.267921882 -0.655632325 0.702690935

At1g63050 membrane bound O-acyl transferase (MBOAT) family protein 0.435891333 0.561675136 1.010416954 -0.029281011 -0.066745696 1.140046078 -0.490656455 -0.719599293

At1g63180 UGE3 (UDP-D-glucose/UDP-D-galactose 4-epimerase 3); UDP-glucose 4-epimerase/ protein dimerization 0.178752024 0.011734636 -0.731424055 -1.253741318 1.350790646 -2.172344423 1.986987702 -0.715697054

At1g77420 "hydrolase, alpha/beta fold family protein" 0.171042594 0.268881564 -0.752477239 -0.153906087 0.296408837 0.004861842 -0.724026061 1.175883267

At1g60980 ATGA20OX4 (ARABIDOPSIS THALIANA GIBBERELLIN 20-OXIDASE 4); gibberellin 20-oxidase 0.061318748 0.306855899 0.247284354 0.399787142 0.168237499 -0.195297833 0.262830743 -0.607733977

At1g64390 "AtGH9C2 (Arabidopsis thaliana glycosyl hydrolase 9C2); carbohydrate binding / catalytic/ hydrolase, hydrolyzing O-glycosyl compounds" 0.218545215 0.50282009 1.367658916 -0.961400875 -0.503859117 2.168170792 0.409847857 1.014532543

At1g64400 "long-chain-fatty-acid--CoA ligase, putative / long-chain acyl-CoA synthetase, putative" -1.745641629 -0.389405508 -0.698953436 0.23616613 -0.328462571 0.156455736 -0.216079067 0.880191652

At1g71100 RSW10 (RADIAL SWELLING 10); ribose-5-phosphate isomerase 0.69100359 0.046653545 -0.310006018 1.434861732 -1.084352885 -1.422703892 -0.401860838 1.145054158

At1g77590 LACS9 (LONG CHAIN ACYL-COA SYNTHETASE 9); long-chain-fatty-acid-CoA ligase 0.653488265 -0.014841689 0.466434654 -0.749027421 -1.065782499 0.691794239 0.823065494 -0.220151192

At1g29410 PAI3 (phosphoribosylanthranilate isomerase 3); phosphoribosylanthranilate isomerase 0.70774161 0.301288224 0.412573972 0.552137323 -0.002019624 0.066851092 -0.667560334 0.217752868

At1g49820 ATMTK (ARABIDOPSIS THALIANA S-METHYL-5-THIORIBOSE KINASE); S-methyl-5-thioribose kinase -0.022954298 0.188636149 -1.005223621 -1.084299679 0.973819038 1.112920703 -0.906125855 1.427787794

At1g52230 PSAH-2_PSI-H__PSAH2 (PHOTOSYSTEM I SUBUNIT H2) 0.138252119 -3.649466809 0.526763423 -0.105099986 -0.317643874 -0.326058556 -0.544001093 0.184642408

At1g73600 methyltransferase/ phosphoethanolamine N-methyltransferase -4.461215654 -0.830413522 2.054025934 -0.275952389 -0.417835518 2.41950983 0.236266052 0.05852314

At1g72190 oxidoreductase family protein 0.135744416 -0.207088066 -0.399167473 0.046865548 1.188665364 0.602261207 -0.352834651 -0.866916111

At1g68420 asparaginyl-tRNA synthetase-related 0.152568473 0.134702766 0.165024696 0.161340194 0.116609229 -0.189449808 -0.012902007 -0.257505498

At1g71380 "ATGH9B3__ATCEL3 (ARABIDOPSIS THALIANA CELLULASE 3); catalytic/ hydrolase, hydrolyzing O-glycosyl compounds" -0.45462935 0.984138729 0.634914454 -0.034748062 0.185648349 -0.194884113 1.013312318 -0.551358834

At1g72680 "cinnamyl-alcohol dehydrogenase, putative" 0.847322145 0.435512267 -2.957036547 0.27805608 1.415940214 -0.226062699 -1.349431225 -0.935016728

At1g72550 tRNA synthetase beta subunit family protein 0.94282974 0.199818599 0.723245152 0.005413117 -0.009359465 0.352009831 -0.745364329 -0.216722808

At1g76550 "pyrophosphate--fructose-6-phosphate 1-phosphotransferase alpha subunit, putative / pyrophosphate-dependent 6-phosphofructose-1-kinase, putative" 1.03349748 0.663775693 0.68403609 -1.055263417 -0.861650588 0.489118705 -0.22416185 0.198830947

At1g76470 3-beta-hydroxy-delta5-steroid dehydrogenase/ binding / catalytic/ cinnamoyl-CoA reductase 1.330327178 -0.120711322 -1.432245178 -0.114814543 -1.186571941 0.568376397 -0.391978981 -0.686898041

At1g76490 HMGR1__HMG1 (HYDROXY METHYLGLUTARYL COA REDUCTASE 1); hydroxymethylglutaryl-CoA reductase 0.163444874 0.018059019 -0.048957083 -1.185340416 0.596970041 -1.256305203 0.750126377 0.63662379

At1g67990 "ATTSM1__TSM1; N1,N5,N10-tris-(5-hydroxyferuloyl)spermidine O-methyltransferase/ caffeoyl CoA:S-adenosyl-L-methionine O-methyltransferase/ caffeoyl-CoA O-methyltransferase/ tricaffeoyl spermidine:S-adenosyl-L-methionine O-methyltransferase/ trihydroxyferul" 2.09344659 -0.610326407 -0.00862724 -0.157408433 -0.37961 0.260299721 0.773787121 0.566552659

At1g68020 "TPS6__ATTPS6; alpha,alpha-trehalose-phosphate synthase (UDP-forming)/ transferase, transferring glycosyl groups / trehalose-phosphatase" 0.975920868 -0.472587472 -0.175284312 -0.738999517 0.570604488 -1.51347114 -0.173754019 -0.7294953

At1g68010 HPR; glycerate dehydrogenase/ poly(U) binding -0.624887332 -4.086382601 -0.553366763 -0.41624918 -0.703028279 0.23187135 -0.251019822 0.707283857

At1g67980 CCoAMT; caffeoyl-CoA O-methyltransferase 0.528382571 0.318028069 -2.223839992 1.414936444 0.261852751 -1.694963005 -1.054595586 0.724182377

At1g30040 ATGA2OX2 (GIBBERELLIN 2-OXIDASE); gibberellin 2-beta-dioxygenase 1.500758537 -0.390299758 -0.563597488 1.166961634 -1.115096756 -2.240719051 0.893504566 0.614423347

At1g68760 ATNUDT1__ATNUDX1 (ARABIDOPSIS THALIANA NUDIX HYDROLASE 1); dihydroneopterin triphosphate pyrophosphohydrolase/ hydrolase 0.447839605 0.569216458 0.892118951 0.005052296 -0.56288476 -0.087270373 -1.057337343 0.334435526

At1g78090 ATTPPB (TREHALOSE-6-PHOSPHATE PHOSPHATASE); trehalose-phosphatase -0.449092549 1.430723712 0.629004485 -1.732558827 -0.023607736 0.322266788 1.5599786 0.7500954

At1g73680 "pathogen-responsive alpha-dioxygenase, putative" -0.640660641 -0.870444649 -2.122202889 -0.938543786 0.012272116 1.075024608 0.755228166 -1.41907534

At1g63290 "ribulose-phosphate 3-epimerase, cytosolic, putative / pentose-5-phosphate 3-epimerase, putative" 0.596729331 0.217346995 -0.05974941 -0.594054412 0.164589862 0.481667726 -0.50472241 0.539613528

At1g73250 "ATFX__GER1 (GDP-4-KETO-6-DEOXYMANNOSE-3,5-EPIMERASE-4-REDUCTASE 1); GDP-L-fucose synthase" -0.481408778 0.974072866 0.824866338 0.381954208 0.149704996 -0.739875446 0.792995712 0.527763866

At1g66430 pfkB-type carbohydrate kinase family protein 0.879360606 -0.713615174 2.166892737 -0.054404299 0.164735837 0.615940538 -0.480611764 -0.239847691

At1g36370 SHM7 (serine hydroxymethyltransferase 7); catalytic/ glycine hydroxymethyltransferase/ pyridoxal phosphate binding -0.716141822 0.095926499 0.085124383 0.872165582 -1.742043194 -1.799015177 0.138536166 -0.484995429

At1g66390 ATMYB90_PAP2__MYB90 (MYB DOMAIN PROTEIN 90); DNA binding / transcription factor 0.089517071 0.222054297 -1.72203855 1.855719365 -0.179143218 1.092945017 -0.212392732 -0.005165733

At1g71920 "histidinol-phosphate aminotransferase, putative" 0.170516545 -0.09246431 0.878580351 -0.246321654 -0.061271514 0.691273574 -0.64147443 0.441675422

At1g72000 "beta-fructofuranosidase, putative / invertase, putative / saccharase, putative / beta-fructosidase, putative" -0.194425275 0.263147688 0.398750623 0.3545132 0.205324633 -0.6142653 0.226573462 -0.444734479

At1g70710 "CEL1__ATGH9B1 (ARABIDOPSIS THALIANA GLYCOSYL HYDROLASE 9B1); cellulase/ hydrolase, hydrolyzing O-glycosyl compounds" 3.535770002 0.316875125 1.893142147 -1.199591615 -0.176127576 0.343271714 -0.636185678 1.570301126

At1g67550 URE (UREASE); urease -0.389634044 0.490913833 -0.219919455 0.761852296 1.247738918 0.554947586 -0.141329825 0.551475735

At1g67560 lipoxygenase family protein 0.827600921 -0.035335535 -0.202545209 0.089271345 0.128655331 -0.734315622 0.657444552 1.431589091

At1g70730 "phosphoglucomutase, cytoplasmic, putative / glucose phosphomutase, putative" -1.258771097 -0.283715522 -0.229070091 0.55656844 -0.182808014 0.894546129 0.503859547 -0.438043161

At1g74470 geranylgeranyl reductase 0.699689258 -3.11014251 0.458800954 -1.521267133 -0.582232691 -0.109456669 -1.097002488 0.376559003

At1g68460 ATIPT1 (isopentenyltransferase 1); adenylate dimethylallyltransferase 0.246012299 0.155536553 0.218106647 0.188004249 0.066952603 -0.272915303 -0.007882677 -0.481265718

At1g63710 CYP86A7; fatty acid (omega-1)-hydroxylase/ oxygen binding 2.655214876 -0.7299333 0.433102019 -0.068690673 -0.101519293 0.442405502 1.185871683 0.801973138

At1g63650 ATMYC-2_EGL1__EGL3 (ENHANCER OF GLABRA 3); DNA binding / transcription factor 2.187692104 0.472677375 0.879865747 -0.08535558 -0.464074818 0.927268754 -0.524481007 0.23217962

At1g74320 "choline kinase, putative" 0.073428202 0.32712765 0.494193218 0.212486199 0.552117748 -0.349227346 1.244096309 -0.768062445

At1g74260 PUR4 (purine biosynthesis 4); ATP binding / catalytic/ phosphoribosylformylglycinamidine synthase -0.109365546 0.054735837 0.55216978 0.219816704 -0.071028128 0.767062786 -1.14456079 -0.161750812

At1g68530 "CER6_CUT1_G2_POP1__KCS6 (3-KETOACYL-COA SYNTHASE 6); catalytic/ transferase, transferring acyl groups other than amino-acyl groups" 1.311376444 -3.245160246 -1.511288855 -0.27985229 0.337260678 0.350095271 -0.011251626 -1.010755363

At1g80460 GLI1__NHO1 (nonhost resistance to P. s. phaseolicola 1); carbohydrate kinase/ glycerol kinase 0.183164386 0.797041294 -0.053550711 -0.858493902 0.129704723 -0.352755732 0.588463653 1.097143793

At1g80380 phosphoribulokinase/uridine kinase-related -1.1832454 -0.605548682 0.439326304 -0.749389464 0.447644735 -1.617398456 -0.445317049 -0.182610476

At1g80560 "3-isopropylmalate dehydrogenase, chloroplast, putative" 0.695037002 0.111195238 1.466060377 0.23723246 0.400561897 -0.199041978 -1.625230958 0.429241449

At1g80600 WIN1 (HOPW1-1-INTERACTING 1); N2-acetyl-L-ornithine:2-oxoglutarate 5-aminotransferase/ catalytic/ pyridoxal phosphate binding / transaminase -0.25636097 -0.498792026 -0.090689048 0.188051238 0.398926779 0.526351672 -0.673160156 0.112775535

At1g63660 "GMP synthase (glutamine-hydrolyzing), putative / glutamine amidotransferase, putative" 0.839976144 0.717202103 1.20668992 0.96679339 -0.126696713 0.677203273 -1.116896181 0.589025476

At1g80330 ATGA3OX4__GA3OX4 (GIBBERELLIN 3-OXIDASE 4); gibberellin 3-beta-dioxygenase -0.038604631 0.133815678 0.240617387 -0.091527636 -0.121312589 0.036986852 0.137686681 -1.599509898

At1g80340 ATGA3OX2_GA4H__GA3OX2 (GIBBERELLIN 3-OXIDASE 2); gibberellin 3-beta-dioxygenase -0.856484203 0.842541948 0.3989936 -0.017918775 -0.088101718 0.530232449 0.206388826 -0.236071286

At1g70580 GGT2__AOAT2 (ALANINE-2-OXOGLUTARATE AMINOTRANSFERASE 2); L-alanine:2-oxoglutarate aminotransferase/ glycine:2-oxoglutarate aminotransferase -1.837599792 -0.755562728 -0.405007159 0.005600718 -1.092781318 1.039889971 0.897642822 -1.928929163

At1g63970 "MECPS__ISPF; 2-C-methyl-D-erythritol 2,4-cyclodiphosphate synthase" -0.969430406 -1.298069215 -0.07677611 0.222531078 0.27769603 0.193551992 -0.16482262 -0.293728564

At1g70500 "polygalacturonase, putative / pectinase, putative" 0.145255536 0.404960486 -0.534984245 -0.58117332 -0.238508379 -0.224702596 0.133625175 0.570077521

At1g69190 2-amino-4-hydroxy-6-hydroxymethyldihydropteridine diphosphokinase/ dihydropteroate synthase -0.022274365 0.187758878 0.290185754 0.118928289 0.173644165 -0.184732348 0.048288498 -0.189161173

At1g69200 kinase 0.536839446 -0.963463044 1.883231079 1.227552306 0.097083346 0.503537981 -1.049750301 -0.669824164

At1g69370 cm-3__CM3 (chorismate mutase 3); chorismate mutase 0.767988259 -0.0103922 0.794068721 0.088971091 0.1547689 -1.104966854 0.934242461 0.436397941

At1g70560 WEI8__TAA1 (TRYPTOPHAN AMINOTRANSFERASE OF ARABIDOPSIS 1); L-alanine:2-oxoglutarate aminotransferase/ L-glutamine:2-oxoglutarate aminotransferase/ L-leucine:2-oxoglutarate aminotransferase/ L-methionine:2-oxoglutarate aminotransferase/ L-phenylalanine:2-o 2.540652954 0.200088306 0.81807369 -0.201091945 0.189196815 0.494703181 -0.728643425 -0.724023185

At1g69740 HEMB1; catalytic/ metal ion binding / porphobilinogen synthase 0.290005341 -0.921891169 0.875303255 0.087838175 0.436404016 -0.514612652 -0.843870833 0.069186835

At1g73980 phosphoribulokinase/uridine kinase family protein 0.411180563 -0.091798937 -1.550955931 -0.833475089 1.451775437 0.588281567 -0.2857565 -0.340281565

At1g73880 0.692843796 -0.15916841 -1.131815434 0.09915256 -0.371793367 0.003008729 0.330649255 -0.566437427

At1g74040 IPMS2_MAML-3__IMS1 (2-ISOPROPYLMALATE SYNTHASE 1); 2-isopropylmalate synthase -0.26488096 -0.487532882 0.759682763 0.451615832 0.124765561 -0.004570988 -0.076143426 -0.043673712

At1g74090 SOT18 (DESULFO-GLUCOSINOLATE SULFOTRANSFERASE 18); 3-methylthiopropyl-desulfoglucosinolate sulfotransferase/ 4-methylthiobutyl-desulfoglucosinolate sulfotransferase/ 5-methylthiopentyl-desulfoglucosinolate sulfotransferase/ 7-methylthioheptyl-desulfogluco -2.635745957 0.046266274 -0.109177016 -1.188303995 0.336977736 1.480551736 0.321015775 1.194442333

At1g74100 SOT16 (SULFOTRANSFERASE 16); desulfoglucosinolate sulfotransferase/ sulfotransferase -0.097003187 0.236461396 -0.337676275 0.301095277 -1.118551908 -1.323039375 -1.309862247 2.63849042

At1g74030 "enolase, putative" 0.934385002 1.581622636 0.970411295 -0.43157655 -1.233251324 1.434385212 -1.001084652 0.301159451

At1g72520 "lipoxygenase, putative" 0.138375647 -0.580736403 -0.179891618 4.32777127 -2.666732377 -5.045582631 0.335652051 0.932721146

At1g69830 ATAMY3__AMY3 (ALPHA-AMYLASE-LIKE 3); alpha-amylase 0.170867134 -0.897816785 0.45083905 0.513160102 3.931781203 -0.057717378 -0.263611611 0.378914453

At1g69640 SBH1 (SPHINGOID BASE HYDROXYLASE 1); catalytic/ sphingosine hydroxylase -0.138115982 0.309419238 -0.581116443 0.616370284 0.476687277 -0.041125454 -0.149765601 0.447237056

At1g11090 "hydrolase, alpha/beta fold family protein" 0.190914984 -0.069143649 -0.095721753 -0.247571125 -0.20756557 0.772506211 -0.413765092 -0.315833099

At1g51440 lipase class 3 family protein -0.115402441 -0.623400195 -0.982437581 -0.739263616 -0.501828683 0.749667412 1.999458525 0.453363123

At2g41850 ADPG2__PGAZAT (POLYGALACTURONASE ABSCISSION ZONE A. THALIANA); polygalacturonase 0.093142425 0.088359478 -0.402091641 0.154203176 0.040844828 -0.259198635 0.234678255 -0.181890875

At1g47260 APFI__GAMMA CA2 (GAMMA CARBONIC ANHYDRASE 2); carbonate dehydratase -0.021811012 0.356852547 0.563434401 -0.012775982 -0.159663773 0.472219872 -0.12639939 0.85040944

At1g51420 ATSPP1__SPP1 (SUCROSE-PHOSPHATASE 1); catalytic/ magnesium ion binding / phosphatase/ sucrose-phosphatase -0.889260765 1.780439848 0.283589928 -0.383046563 -0.225550585 -0.210220598 0.786542125 0.532318022

At1g51410 "cinnamyl-alcohol dehydrogenase, putative (CAD)" -0.200553322 0.877347664 0.655015099 0.938923722 0.447007491 -0.325745816 2.423482634 -0.437209149

At2g47240 long-chain-fatty-acid--CoA ligase family protein / long-chain acyl-CoA synthetase family protein 2.959575177 -1.717716578 0.165140933 0.17000031 0.913315265 0.774052546 0.762669718 -0.544013683

At2g43350 ATGPX3 (GLUTATHIONE PEROXIDASE 3); glutathione peroxidase -0.093735806 0.387834996 -0.37139216 -0.533745954 1.267529005 0.36735339 -0.555007244 0.90711962

At2g43360 BIOB__BIO2 (BIOTIN AUXOTROPH 2); biotin synthase 0.791430497 0.210169558 1.971787298 -0.972203822 0.331910883 1.142159939 -0.622845437 -0.277899527

At2g43430 GLY1__GLX2-1 (GLYOXALASE 2-1); hydroxyacylglutathione hydrolase 0.111929961 -0.197151939 -0.390892943 -0.839648702 0.117801072 -0.460977517 -0.145694881 0.257747572

At2g43860 "polygalacturonase, putative / pectinase, putative" -0.136939845 0.186260252 0.494646758 0.518842416 0.039031594 -0.367874596 0.413272363 -0.648268121

At2g43840 UGT74F1__UDP-glucoronosyl/UDP-glucosyl transferase family protein 0.428267288 0.492253108 0.107552196 0.179689942 -0.133317998 0.317568665 0.402608178 -0.079962554

At2g43750 ACS1_ATCS-B_CPACS1__OASB (O-ACETYLSERINE (THIOL) LYASE B); cysteine synthase -0.678507248 -0.579132696 0.794028819 -0.296331157 -0.136228324 -0.268937531 -0.70149125 -0.291306516

At2g43820 "GT__UGT74F2 (UDP-GLUCOSYLTRANSFERASE 74F2); UDP-glucose:4-aminobenzoate acylglucosyltransferase/ UDP-glucosyltransferase/ UDP-glycosyltransferase/ transferase, transferring glycosyl groups / transferase, transferring hexosyl groups" -0.854172077 -0.327892025 -2.4425925 -0.701825488 -0.219429292 -1.75932395 -1.65080336 -1.068777473

At2g43710 FAB2__SSI2; acyl-[acyl-carrier-protein] desaturase/ stearoyl-CoA 9-desaturase -0.255872508 -0.707192082 0.507655977 -0.119707863 -1.04001142 0.632089823 -0.52479783 -1.460483434

At2g43760 molybdopterin biosynthesis MoaE family protein -0.046202747 -0.120471579 -0.374089361 -0.918201434 0.352977013 -0.023999691 -1.101134691 -0.383786439

At2g47280 pectinesterase -0.055194594 0.263973268 0.335903727 0.337829386 0.14298684 -0.384159956 0.338531951 -0.639281625

At2g47380 cytochrome c oxidase subunit Vc family protein / COX5C family protein -0.019082236 0.591827649 -0.054458655 -0.306109195 0.056750676 0.201340539 -0.083280132 0.680249019

At1g55850 "CSLE1__ATCSLE1; cellulose synthase/ transferase, transferring glycosyl groups" -0.548797473 -0.079937113 -2.750512848 -0.382189718 1.389320987 0.753123339 -1.279720663 1.190297286

At1g55880 "pyridoxal-5'-phosphate-dependent enzyme, beta family protein" 0.409439723 0.386890554 0.43470444 -0.068207979 0.338835021 -0.224419276 -0.407644644 0.096871143

At1g55920 SAT1_SAT5__ATSERAT2;1 (SERINE ACETYLTRANSFERASE 2;1); serine O-acetyltransferase -1.723900174 0.197366919 -2.184062099 -0.657956596 -0.844778531 -1.710717465 -0.6190786 1.664487921

At1g53240 "malate dehydrogenase (NAD), mitochondrial" -0.240990814 0.175149195 1.045409753 -0.13048496 -0.382345368 0.825441912 -0.145867854 0.672372989

At1g08110 "lactoylglutathione lyase, putative / glyoxalase I, putative" 0.077642366 -0.387201039 -0.108891299 -0.871069846 0.384308813 0.115105965 -0.575372393 0.693804115

At1g08065 ATACA5__ACA5 (ALPHA CARBONIC ANHYDRASE 5); carbonate dehydratase/ zinc ion binding 0.28339939 0.265919855 0.431839383 0.290590844 0.146486537 -0.209149874 0.983777626 -0.244464917

At1g62430 ATCDS1; phosphatidate cytidylyltransferase 0.260899205 -0.231715914 -0.267677645 -0.123770046 2.682057112 0.422617752 -0.049780417 0.090599624

At1g62380 ATACO2__ACO2 (ACC OXIDASE 2); 1-aminocyclopropane-1-carboxylate oxidase -0.070743477 -0.737505668 -0.912908578 -3.121815684 -0.338765894 -1.020195661 -0.732800977 0.255029487

At1g08080 ATACA7__ACA7 (ALPHA CARBONIC ANHYDRASE 7); carbonate dehydratase/ zinc ion binding -0.130205642 0.154617432 0.414145069 0.364335496 0.283222094 -0.56638457 0.312302304 -0.491755537

At1g32440 PKp3 (plastidial pyruvate kinase 3); pyruvate kinase -1.260644135 0.156043653 0.522015803 0.241491862 -1.433104625 0.550232905 0.118887559 -0.121968077

At1g19440 "KCS4 (3-KETOACYL-COA SYNTHASE 4); acyltransferase/ catalytic/ transferase, transferring acyl groups other than amino-acyl groups" -0.713608609 0.845873803 1.732226321 0.715508715 -0.257774204 -0.403603938 0.031896935 -1.49943601

At1g32350 AOX1D (alternative oxidase 1D); alternative oxidase 1.07402059 -0.137639207 -3.620630493 2.7580081 -0.527161666 -1.507542837 -1.069899118 0.954379851

At1g48930 "AtGH9C1 (Arabidopsis thaliana glycosyl hydrolase 9C1); catalytic/ hydrolase, hydrolyzing O-glycosyl compounds" -1.382616503 1.605805252 0.300585453 -2.045991678 -0.577693559 1.015643734 -0.107039948 -1.241325542

At1g49140 NADH-ubiquinone oxidoreductase-related 0.093030584 0.372662417 0.360606326 -0.438154418 0.274870919 0.316507536 0.083742987 0.677719076

At1g78440 ATGA2OX1 (gibberellin 2-oxidase 1); gibberellin 2-beta-dioxygenase 0.613036669 0.122714076 0.357196751 0.101515714 -0.15613694 0.013991193 0.340393316 0.092001736

At1g14610 VALRS__TWN2 (TWIN 2); ATP binding / aminoacyl-tRNA ligase/ nucleotide binding / valine-tRNA ligase 0.792642639 0.411446476 0.573908528 0.146280027 -0.156358816 0.33298457 -0.673618327 0.118389109

At1g06290 ATACX3__ACX3 (ACYL-COA OXIDASE 3); acyl-CoA oxidase -0.024357857 0.398071586 -0.642445523 -0.165181652 -0.003571841 -0.115566985 0.37552027 -0.057127826

At1g06250 lipase class 3 family protein 0.715923968 0.034550951 0.294247652 0.178556659 0.028339116 0.026330737 1.411846881 0.114406123

At1g78390 ATNCED9__NCED9 (NINE-CIS-EPOXYCAROTENOID DIOXYGENASE 9); 9-cis-epoxycarotenoid dioxygenase -0.21506514 0.184936844 -0.162579949 -0.398114442 -0.237530198 0.303424929 -0.063949056 -2.899442985

At1g78270 "AtUGT85A4 (UDP-glucosyl transferase 85A4); UDP-glycosyltransferase/ glucuronosyltransferase/ transferase, transferring hexosyl groups" -0.987689987 -0.345934021 0.180788782 -0.046873239 1.811123319 -1.416748885 -0.821646637 -0.449959686

At1g43710 emb1075 (embryo defective 1075); carboxy-lyase/ catalytic/ pyridoxal phosphate binding -0.289334205 0.740629148 0.842968648 0.01168855 0.099029574 0.493675355 0.545185315 -0.020699402

At1g06780 "GAUT6 (Galacturonosyltransferase 6); polygalacturonate 4-alpha-galacturonosyltransferase/ transferase, transferring glycosyl groups" 0.216885206 0.906543749 -0.9588593 0.004883359 -0.073821763 0.249877622 0.149958466 -0.622508891

At1g06800 lipase class 3 family protein -0.002433117 0.39500536 -0.099092799 0.591194564 -0.076574205 -1.078160232 -0.497619961 -0.16873797

At1g06730 pfkB-type carbohydrate kinase family protein 0.289976821 0.261999757 0.772958441 0.579618883 0.112715425 0.267705484 0.002817591 0.037784608

At1g43670 "fructose-1,6-bisphosphatase, putative / D-fructose-1,6-bisphosphate 1-phosphohydrolase, putative / FBPase, putative" -0.927469682 -1.350667181 0.639832358 0.166763086 1.327638825 -0.533065958 0.924380305 -0.284505903

At1g17290 AlaAT1 (ALANINE AMINOTRANSFERAS); ATP binding / L-alanine:2-oxoglutarate aminotransferase -0.086152667 0.292705714 -0.214912365 1.138332414 1.043607306 -1.152231338 0.782096235 0.912123388

At1g21850 sks8 (SKU5 Similar 8); copper ion binding / oxidoreductase 0.442493869 0.174247316 0.174214172 0.964324811 0.053747176 -1.202525124 0.43232007 -0.470970445

At1g21860 sks7 (SKU5 Similar 7); copper ion binding / oxidoreductase -0.157170733 0.456621114 0.468013912 0.601213513 0.112677504 -0.45382436 0.711127197 -0.621840764

At1g43800 "acyl-(acyl-carrier-protein) desaturase, putative / stearoyl-ACP desaturase, putative" 3.415921567 0.888515313 1.324868853 0.412341341 -0.228153445 0.812556787 -2.757775565 -2.556804273

At1g21440 mutase family protein -0.37453655 -0.20041476 0.796063176 -1.138249458 0.024117013 1.504593328 -0.501398518 0.994376202

At1g02500 MAT1_SAM-1__SAM1 (S-ADENOSYLMETHIONINE SYNTHETASE 1); methionine adenosyltransferase 0.160899686 0.496312068 -0.428509421 -1.03019651 -0.315165753 0.191269528 -0.791653878 0.533141266

At1g06020 pfkB-type carbohydrate kinase family protein 0.0698573 0.146006484 0.253313741 0.095483101 0.110882321 -0.372850325 0.184655833 -0.458886947

At1g06100 fatty acid desaturase family protein -0.22273171 0.041132003 1.314540003 1.049363493 0.678012054 -0.251073334 -0.152397245 -0.578829563

At1g06120 fatty acid desaturase family protein -0.828882705 1.755911242 0.22490265 -1.693898494 0.050771169 -0.010110349 0.083803484 0.959520486

At1g06130 GLX2-4 (glyoxalase 2-4); hydrolase/ hydroxyacylglutathione hydrolase/ zinc ion binding 0.002418032 -0.005161152 -0.395422659 0.678491522 0.332631614 -0.315233113 -0.439469301 -0.620501056

At1g06000 UDP-glucoronosyl/UDP-glucosyl transferase family protein 1.656821076 0.696734226 0.559624963 2.479826257 -3.181555737 3.082856257 -1.750310208 -0.50987574

At1g06080 ADS1 (DELTA 9 DESATURASE 1); oxidoreductase 2.591078631 -0.369373467 1.843878451 -1.351889656 0.458328092 -0.95235719 -0.750346677 -0.496341107

At1g44980 pectinesterase family protein 0.091781057 0.274815525 0.180959068 0.251367454 0.188065197 -0.252322707 0.145682234 -0.490517473

At1g12240 "VAC-INV__ATBETAFRUCT4; beta-fructofuranosidase/ hydrolase, hydrolyzing O-glycosyl compounds" -0.928955574 -0.684566648 0.700040472 -0.949222694 -1.910753054 -1.497446267 2.027747518 1.491096081

At1g53520 chalcone-flavanone isomerase-related 0.565659249 -0.946538281 2.972619915 -0.58040491 -0.082957307 -0.156490197 -0.496605363 -1.241737501

At1g53500 "ATMUM4_ATRHM2_RHM2__MUM4 (MUCILAGE-MODIFIED 4); UDP-4-keto-6-deoxy-glucose-3,5-epimerase/ UDP-4-keto-rhamnose-4-keto-reductase/ UDP-L-rhamnose synthase/ UDP-glucose 4,6-dehydratase/ catalytic" -0.121292433 0.299681722 0.844499326 0.149526118 -0.946877033 0.562338711 1.185369894 -0.248540589

At1g53580 ETHE1_GLX2-3__GLY3 (GLYOXALASE II 3); hydrolase/ hydroxyacylglutathione hydrolase -0.262605226 0.106309555 -1.970962132 -1.733496157 -0.184761161 -0.671015502 -0.27018668 0.252410519

At1g12140 FMO GS-OX5 (FLAVIN-MONOOXYGENASE GLUCOSINOLATE S-OXYGENASE 5); 8-methylthiopropyl glucosinolate S-oxygenase/ flavin-containing monooxygenase/ monooxygenase -1.801352945 0.188305199 -0.98915501 -0.343404967 -0.134222466 0.660218646 -0.195751032 0.492929555

At1g26570 "ATUGD1__UGD1 (UDP-GLUCOSE DEHYDROGENASE 1); NAD or NADH binding / UDP-glucose 6-dehydrogenase/ binding / catalytic/ coenzyme binding / oxidoreductase/ oxidoreductase, acting on the CH-OH group of donors, NAD or NADP as acceptor" 0.956160688 -0.393612959 1.61188357 0.404945378 -0.291913935 -0.160681645 0.429459577 -1.037390786

At1g17410 nucleoside diphosphate kinase family protein 0.283445439 0.332447122 0.433906284 0.218164968 0.32478133 -0.741991061 0.064354419 -0.169810274

At1g17420 "LOX3; electron carrier/ iron ion binding / lipoxygenase/ metal ion binding / oxidoreductase, acting on single donors with incorporation of molecular oxygen, incorporation of two atoms of oxygen" 0.788216886 -1.085547104 -0.641102036 3.881512203 -1.388490394 -2.980528 0.134526409 0.226334024

At1g01290 CNX3 (COFACTOR OF NITRATE REDUCTASE AND XANTHINE DEHYDROGENASE 3); catalytic 0.508481351 0.003191858 -0.231917394 -0.216516712 -0.175257358 0.571106801 -0.445366521 0.296227449

At1g01390 UDP-glucoronosyl/UDP-glucosyl transferase family protein -1.562882902 -0.144588571 0.034990614 1.069437416 -0.157370176 0.977440599 0.360158485 -0.512852735

At1g01420 "UGT72B3 (UDP-GLUCOSYL TRANSFERASE 72B3); UDP-glycosyltransferase/ quercetin 3-O-glucosyltransferase/ transferase, transferring glycosyl groups" -1.11574846 0.193575609 -0.331778659 0.181700423 0.158194422 0.618891483 -0.265909872 -0.948889306

At1g62940 ACOS5 (ACYL-COA SYNTHETASE 5); 4-coumarate-CoA ligase/ long-chain-fatty-acid-CoA ligase/ medium-chain-fatty-acid-CoA ligase 1.312288743 -0.195325906 0.32491292 0.017259477 -0.298486173 0.054212287 0.335722915 -0.163017238

At1g63000 "UER1__NRS/ER (NUCLEOTIDE-RHAMNOSE SYNTHASE/EPIMERASE-REDUCTASE); UDP-4-keto-6-deoxy-glucose-3,5-epimerase/ UDP-4-keto-rhamnose-4-keto-reductase/ dTDP-4-dehydrorhamnose 3,5-epimerase/ dTDP-4-dehydrorhamnose reductase" -0.916979299 1.189586802 0.862723526 -0.341475023 -0.160307153 0.67084311 0.775580825 0.116551362

At1g62960 ACS10 (ACC SYNTHASE 10); 1-aminocyclopropane-1-carboxylate synthase/ L-aspartate:2-oxoglutarate aminotransferase/ aromatic-amino-acid:2-oxoglutarate aminotransferase -0.411863086 -0.611661056 0.500165224 -0.325694107 -1.248739544 -0.066264164 0.645638319 -0.042206584

At1g75450 ATCKX5_ATCKX6__CKX5 (CYTOKININ OXIDASE 5); cytokinin dehydrogenase 1.589878064 0.392405175 -0.36375572 0.832584692 -1.198528486 -0.860734612 0.334358845 -0.805466264

At1g75330 OTC (ORNITHINE CARBAMOYLTRANSFERASE); amino acid binding / carboxyl- or carbamoyltransferase/ ornithine carbamoyltransferase 0.297490865 0.008283365 0.457204023 0.290346384 0.076308894 0.445138912 -1.086113872 0.451103681

At1g19600 pfkB-type carbohydrate kinase family protein -0.224603337 0.693421099 0.537536766 -0.622966567 0.357652735 0.130260028 -0.798120041 0.52752671

At1g19580 GAMMA CA1 (GAMMA CARBONIC ANHYDRASE 1); carbonate dehydratase 0.797421767 0.287923833 0.673011172 0.607449168 -0.323316427 -0.133288737 -0.423923237 0.02436396

At1g19570 DHAR1 (dehydroascorbate reductase); glutathione dehydrogenase (ascorbate) 1.019962547 0.008406956 -0.991965883 -0.30778857 0.352899754 0.56885104 -0.876748179 0.396119664

At1g34430 EMB3003 (embryo defective 3003); acyltransferase/ dihydrolipoyllysine-residue acetyltransferase/ protein binding 0.338865117 -0.194819996 1.365568708 -1.228915899 -0.18876273 0.448085097 -0.299108778 -0.011872913

At1g04920 "ATSPS3F (sucrose phosphate synthase 3F); sucrose-phosphate synthase/ transferase, transferring glycosyl groups" 0.020726174 0.203039102 0.510719019 0.607371415 0.184093895 0.121570467 0.812437342 -2.670906933

At1g12900 GAPA-2 (GLYCERALDEHYDE 3-PHOSPHATE DEHYDROGENASE A SUBUNIT 2); NAD or NADH binding / binding / catalytic/ glyceraldehyde-3-phosphate dehydrogenase (phosphorylating)/ glyceraldehyde-3-phosphate dehydrogenase 0.527904925 -4.11915138 0.510827372 0.051177689 -0.504004267 -0.008811264 -0.263132054 0.175687145

At1g12780 UGE1 (UDP-D-glucose/UDP-D-galactose 4-epimerase 1); UDP-glucose 4-epimerase/ protein dimerization -0.248654996 -1.024882925 -1.419265083 -4.218755713 1.883581748 -1.467951119 1.427990557 -0.123322237

At1g33030 O-methyltransferase family 2 protein 0.119938625 0.437284564 -1.908192761 1.907724611 0.163256182 -1.00659828 -0.942191939 0.342137236

At1g05800 DGL (DONGLE); triacylglycerol lipase 0.055401457 0.215804766 0.227643766 0.291220408 0.051860994 -0.454477803 0.075033887 -0.451175039

At1g48470 GLN1;5 (glutamine synthetase 1;5); glutamate-ammonia ligase 0.29602322 0.66977261 0.37258213 0.145539843 -0.176577396 0.428828632 1.738009527 -4.242457935

At1g48520 "GATB (GLU-ADT SUBUNIT B); carbon-nitrogen ligase, with glutamine as amido-N-donor / glutaminyl-tRNA synthase (glutamine-hydrolyzing)/ ligase" -0.2623057 -0.90777954 1.306253934 0.521869487 0.522326076 -0.124757871 -0.547482998 0.427135491

At1g48600 "phosphoethanolamine N-methyltransferase 2, putative (NMT2)" -2.804943878 0.648839233 1.121474039 -0.313579931 -0.825431674 0.73316463 1.723123998 2.048831541

At1g79690 atnudt3 (Arabidopsis thaliana Nudix hydrolase homolog 3); hydrolase 1.04245372 0.14981994 -0.550192893 0.178007082 0.205047139 0.704886196 -0.530663824 -0.155249762

At1g41830 SKS6 (SKU5-SIMILAR 6); pectinesterase -1.040989365 0.315812666 1.432076336 -1.042897824 -0.755484963 0.250127439 0.004470353 0.509984016

At1g53000 cytidylyltransferase family 0.329448213 0.234016864 0.578205621 -0.594873709 1.185065715 0.573883253 -0.751501632 -0.587461676

At1g07890 CS1_MEE6__APX1 (ascorbate peroxidase 1); L-ascorbate peroxidase 0.342489859 0.150262805 -0.439133206 -0.053145994 -0.703581283 0.44696842 -0.2519484 1.923379869

At1g18870 ICS2 (ISOCHORISMATE SYNTHASE 2); isochorismate synthase -0.2999936 1.067011079 -0.945640276 -0.081853046 0.161722283 0.414567149 -0.629032293 0.5443065

At1g14520 -0.078923587 0.232843374 0.245386359 0.361158361 0.293852956 -0.297478296 0.260484354 -0.705710416

At1g14290 SBH2 (SPHINGOID BASE HYDROXYLASE 2); catalytic/ sphingosine hydroxylase -0.208927735 -0.008517433 2.218341683 -1.353965649 0.255099409 -2.326917987 2.182243307 0.978167273

At1g71697 CK__ATCK1 (CHOLINE KINASE 1); choline kinase -0.593695596 0.914831689 -0.783967065 0.697351598 -1.472908891 -2.643606955 2.020736724 0.781526521

At1g71750 phosphoribosyltransferase family protein 0.837690901 0.019822662 0.311220732 0.331142371 -0.162632849 0.515892142 -0.420162617 0.495686568

At1g01710 acyl-CoA thioesterase family protein 0.378268628 -0.170371106 -0.530687501 0.053173843 0.031734567 -0.17046317 -0.067985639 -0.306799679

At1g01120 KCS1 (3-KETOACYL-COA SYNTHASE 1); acyltransferase/ fatty acid elongase 0.324107313 -0.940221227 0.686952426 -1.719225037 -1.506292481 -1.79204913 -1.430753167 -0.804402631

At1g01050 AtPPa1 (Arabidopsis thaliana pyrophosphorylase 1); inorganic diphosphatase -0.469157455 0.274185856 0.415427707 -0.317679857 0.050295499 0.626734013 0.530291418 0.446967437

At1g01090 PDH-E1 ALPHA (PYRUVATE DEHYDROGENASE E1 ALPHA); pyruvate dehydrogenase (acetyl-transferring) 0.144387922 -0.164306943 0.64128714 -1.063236352 -0.414678398 0.203060588 0.058189915 -0.236466023

At1g02000 GAE2 (UDP-D-GLUCURONATE 4-EPIMERASE 2); UDP-glucuronate 4-epimerase/ catalytic -0.419834733 0.581209956 0.211095274 0.068360855 -0.296182488 -0.773626427 2.658978298 -0.516391905

At1g27680 APL2 (ADPGLC-PPASE LARGE SUBUNIT); glucose-1-phosphate adenylyltransferase 0.835789272 0.351685003 0.80672157 1.150157773 1.215632081 1.240670671 -0.340552118 -0.207643438

At1g18360 "hydrolase, alpha/beta fold family protein" 0.483748599 -0.981288632 0.963941638 -0.366940391 0.753984837 0.056382177 -0.145667754 -0.917684492

At1g18500 IPMS1__MAML-4 (METHYLTHIOALKYLMALATE SYNTHASE-LIKE 4); 2-isopropylmalate synthase 0.373428935 -0.089934441 0.333440612 0.363684655 0.564603425 -0.721305995 -1.502865855 -0.783599043

At1g18270 ketose-bisphosphate aldolase class-II family protein 0.502039555 0.562238572 -0.805207304 -1.567646013 0.483342255 -0.815520493 -0.031521459 0.359760432

At1g50090 aminotransferase class IV family protein -0.005223747 0.515664881 0.389170362 0.672671124 0.031930082 -0.031219486 0.061493872 -0.266856227

At1g08520 PDE166__CHLD; ATP binding / magnesium chelatase/ nucleoside-triphosphatase/ nucleotide binding -0.1494064 -1.70091262 0.825991137 -0.426030194 0.558352733 -0.434501624 -0.418774832 0.183451453

At1g32780 "alcohol dehydrogenase, putative" 0.457565198 -0.383417964 1.079082335 -0.025546423 -0.709062232 -0.378515969 1.900891343 0.061165257

At1g08480 unknown protein 0.308197121 0.620411516 -0.062911595 -0.160351327 0.536598156 0.744384096 -0.165862395 0.183509787

At1g08510 FATB (fatty acyl-ACP thioesterases B); acyl carrier/ acyl-[acyl-carrier-protein] hydrolase 0.50193634 -0.149845592 -0.083353696 -0.74463889 -0.189886757 -0.800470683 1.090692601 0.182482346

At1g76160 sks5 (SKU5 Similar 5); copper ion binding / oxidoreductase -0.20075266 -0.319658361 2.064229478 -0.220578801 -1.135319858 -1.983996732 -0.624871015 0.388953198

At1g47840 HXK3 (HEXOKINASE 3); ATP binding / fructokinase/ glucokinase/ hexokinase -0.655894529 1.189719715 0.569888569 -1.124482636 -0.162164439 0.730471078 -0.679233118 -0.651834572

At1g08490 ATCPNIFS_ATNFS2_ATSUFS_SUFS__CPNIFS (CHLOROPLASTIC NIFS-LIKE CYSTEINE DESULFURASE); cysteine desulfurase/ selenocysteine lyase/ transaminase -0.306085852 -0.377799208 -0.007595392 0.649306103 0.052443662 -0.190237036 -0.046039409 -0.517137183

At1g08380 PSAO (photosystem I subunit O) 0.558908987 -4.009307145 -0.12659825 -0.732326334 -0.243018418 -0.293998184 -0.919481379 0.226947226

At1g76130 ATAMY2__AMY2 (ALPHA-AMYLASE-LIKE 2); alpha-amylase/ calcium ion binding / catalytic/ cation binding -1.703443164 -0.5960131 -1.089095914 2.111751458 -1.620063294 -0.520274826 0.011763598 -1.082057105

At1g08250 ADT6 (arogenate dehydratase 6); arogenate dehydratase/ prephenate dehydratase 0.058698693 0.394173556 -1.045842017 0.19084007 -1.542513241 1.367653254 -0.528960873 -0.322296131

At1g15550 ATGA3OX1_GA4__GA3OX1 (GIBBERELLIN 3-OXIDASE 1); gibberellin 3-beta-dioxygenase/ transcription factor binding -1.05927304 0.340454985 1.013509629 -1.069860009 -1.009730648 -0.176963788 -0.076775126 -0.08331169

At1g76150 maoC-like dehydratase domain-containing protein 0.469723888 -0.006868864 -1.622893832 -0.26726451 0.972911585 -0.011608779 -0.316883557 0.379357023

At1g15950 ATCCR1_IRX4__CCR1 (CINNAMOYL COA REDUCTASE 1); cinnamoyl-CoA reductase -0.472008478 -0.30465598 -0.728407494 -1.411061755 -1.968189526 -0.039273074 -1.285249667 -0.77249736

At1g30520 AAE14 (Acyl-Activating Enzyme 14); AMP binding / o-succinylbenzoate-CoA ligase 0.419978803 -1.401331193 1.998584904 -0.022215694 0.244942901 -0.421093137 0.058399641 0.396139868

At1g30530 "UGT78D1 (UDP-GLUCOSYL TRANSFERASE 78D1); UDP-glycosyltransferase/ quercetin 3-O-glucosyltransferase/ transferase, transferring glycosyl groups" 0.977068138 -1.021111173 0.573808929 0.999266284 -1.993778787 3.306726553 -0.895306637 -0.635623342

At1g11580 ATPMEPCRA__PMEPCRA (METHYLESTERASE PCR A); enzyme inhibitor/ pectinesterase -1.793145917 1.445528313 0.611282361 -1.069361758 -2.265757401 -0.414822396 -0.808085378 2.217324677

At1g10670 ACLA-1; ATP citrate synthase 0.676722539 0.157103052 0.658145204 -0.719089928 -0.221497217 0.306646067 0.172896424 0.662171009

At1g11590 "pectin methylesterase, putative" 0.044284866 0.012759887 0.12740608 -0.233894497 -0.10646103 0.114633058 0.161602395 -0.854646215

At1g11370 pectinesterase family protein 0.090674268 0.175403628 0.181060392 0.167861063 0.135090631 -0.267247682 0.125939996 -0.450962434

At1g50460 HKL1 (HEXOKINASE-LIKE 1); ATP binding / fructokinase/ glucokinase/ hexokinase 0.503922909 -0.108287525 1.483732907 0.354792841 -1.030398119 -1.984806589 -0.319179753 -0.26634073

At1g50480 THFS (10-FORMYLTETRAHYDROFOLATE SYNTHETASE); ATP binding / copper ion binding / formate-tetrahydrofolate ligase -0.345609693 -0.323581727 -0.552344235 -0.961795682 1.341668715 -0.082838052 -0.575368111 0.736193963

At1g50580 glycosyltransferase family protein -0.375692741 1.036292844 0.286713075 -0.086897773 0.15496267 -0.047028842 0.125476577 0.095274624

At1g80820 CCR2 (CINNAMOYL COA REDUCTASE); cinnamoyl-CoA reductase 0.668877057 1.334350268 -0.520835167 1.557926348 -2.459012227 -2.938024146 -1.224607801 -0.325736571

At1g80860 PLMT (PHOSPHOLIPID N-METHYLTRANSFERASE); phosphatidyl-N-dimethylethanolamine N-methyltransferase/ phosphatidyl-N-methylethanolamine N-methyltransferase 0.764766507 0.280580722 0.539807618 -0.271188638 0.465104183 0.313553994 -0.260573987 0.060397586

At1g65060 4CL3; 4-coumarate-CoA ligase 0.770084379 -0.016778466 0.740123054 0.922414797 -2.729310824 3.507625766 0.271317596 0.754680526

At1g65860 FMO GS-OX1 (FLAVIN-MONOOXYGENASE GLUCOSINOLATE S-OXYGENASE 1); 3-methylthiopropyl glucosinolate S-oxygenase/ 4-methylthiopropyl glucosinolate S-oxygenase/ 5-methylthiopropyl glucosinolate S-oxygenase/ 6-methylthiopropyl glucosinolate S-oxygenase/ 7-methyl -3.102580695 -0.709401731 0.843775737 0.907106793 -0.094126607 -0.258568233 0.266647211 -0.480766538

At1g65880 BZO1 (BENZOYLOXYGLUCOSINOLATE 1); benzoate-CoA ligase 0.042457756 0.054760562 -0.067348727 -0.372417087 -0.252403569 0.411898263 -0.665984682 -3.013347171

At1g22380 "AtUGT85A3 (UDP-glucosyl transferase 85A3); glucuronosyltransferase/ transcription factor/ transferase, transferring glycosyl groups" 0.327735787 -0.184893975 -1.156048584 -0.131601796 0.561163592 -0.540069225 2.551537622 0.629720077

At1g22440 "alcohol dehydrogenase, putative" -1.023929809 2.429257441 -0.058278718 -2.316190171 -0.860770323 0.254195037 -0.982894763 0.944391999

At1g22430 "alcohol dehydrogenase, putative" -0.07064715 -1.268402876 1.038411835 -0.775311315 0.376808768 -0.179694386 -1.275677776 -1.827197249

At1g22410 "2-dehydro-3-deoxyphosphoheptonate aldolase, putative / 3-deoxy-D-arabino-heptulosonate 7-phosphate synthase, putative / DAHP synthetase, putative" 0.647519163 0.510852132 -0.674875534 -0.085049698 -0.93803718 0.000781976 -0.598793553 0.749918215

At1g22400 "ATUGT85A1__UGT85A1; UDP-glycosyltransferase/ cis-zeatin O-beta-D-glucosyltransferase/ glucuronosyltransferase/ trans-zeatin O-beta-D-glucosyltransferase/ transferase, transferring glycosyl groups / transferase, transferring hexosyl groups" -0.485663852 -0.399749017 -1.268277277 2.258651773 -1.189777536 -3.217429341 0.036592785 0.90208823

At1g22450 ATCOX6B2__COX6B (CYTOCHROME C OXIDASE 6B); cytochrome-c oxidase 0.128982349 0.532141744 0.557138815 0.080735754 -0.125260912 0.338147919 -0.003330649 0.448540969

At1g64440 REB1_UGE4__RHD1 (ROOT HAIR DEFECTIVE 1); UDP-glucose 4-epimerase/ protein dimerization 0.129244268 0.685349061 -0.100518815 0.214754367 -0.126771186 0.571663163 0.542421431 0.710087531

At1g64660 ATMGL (ARABIDOPSIS THALIANA METHIONINE GAMMA-LYASE); catalytic/ methionine gamma-lyase 1.464197237 -0.432120694 -3.045140698 -2.520666891 -0.555478808 -0.580402189 0.364991222 -1.675164452

At1g65960 GAD2 (GLUTAMATE DECARBOXYLASE 2); calmodulin binding / glutamate decarboxylase 0.033099525 -0.721618815 -0.139985306 -0.939729877 0.788361187 0.204899786 -0.306991097 3.97312613

At1g37130 B29_CHL3_NIA2-1_NR_NR2__NIA2 (NITRATE REDUCTASE 2); nitrate reductase (NADH)/ nitrate reductase -3.814956961 -0.858233842 -0.914749254 0.167533241 -0.836795035 -0.292286629 -1.019926746 2.522710195

At1g35580 CINV1 (cytosolic invertase 1); beta-fructofuranosidase 0.722348182 0.566252876 0.330109541 -1.19344346 -0.03588946 -1.603042293 -1.528643145 1.450258446

At1g80050 ATAPT2__APT2 (ADENINE PHOSPHORIBOSYL TRANSFERASE 2); adenine phosphoribosyltransferase/ phosphate transmembrane transporter 0.543931981 0.10515229 1.38221183 -1.339133493 0.315510615 1.322462141 -1.010316323 0.111520424

At1g80230 cytochrome c oxidase family protein -0.070164094 0.691820316 0.032581498 0.15748598 -0.12000789 0.413858451 0.389901878 0.586426444

At1g80170 "polygalacturonase, putative / pectinase, putative" 0.273994277 0.186002147 0.852861811 -1.058363068 0.50474371 -0.036261401 -0.240583656 0.484671301

At1g59500 GH3.4; indole-3-acetic acid amido synthetase 2.774824431 1.503991217 -1.044374694 1.919229018 0.482169808 -1.233878624 -1.969007919 -1.654559856

At1g02810 pectinesterase family protein 0.504114849 2.112188704 0.906251124 -1.558362228 -0.220435924 -0.161080165 0.050114242 0.790560529

At1g02730 "CSLD5__ATCSLD5; 1,4-beta-D-xylan synthase/ cellulose synthase" 2.189921228 0.499441195 2.656451397 0.021858028 0.380271414 0.73773438 -1.640473091 0.216258867

At1g02850 "BGLU11 (BETA GLUCOSIDASE 11); catalytic/ cation binding / hydrolase, hydrolyzing O-glycosyl compounds" 1.55587055 0.26761432 -4.004698363 0.779332637 0.091286502 0.768730429 -1.810707299 -1.418830654

At1g02800 "CEL2__ATCEL2; cellulase/ hydrolase, hydrolyzing O-glycosyl compounds" 3.992523178 -0.239721867 1.443016298 1.136599015 0.182000081 0.21911823 -0.771181486 -0.062663104

At1g02790 PGA4 (POLYGALACTURONASE 4); polygalacturonase 1.253240423 -0.305451901 -1.002251477 -0.93558856 -0.441293268 0.661163194 7.113880397 0.51830195

At1g52570 PLDALPHA2 (phosphlipase d alpha 2); phospholipase D -0.042571353 0.662392988 0.33016024 0.676352284 0.35496446 0.230293545 2.659610888 0.030730626

At1g75020 LPAT4 (LYSOPHOSPHATIDYL ACYLTRANSFERASE 4); acyltransferase -1.020829411 0.220387316 -0.491381896 0.691213603 -0.942576762 -1.414718406 -0.386450819 -0.410807298

At1g74920 ALDH10A8; 3-chloroallyl aldehyde dehydrogenase/ oxidoreductase 0.509991277 -0.231016464 -0.291535842 -0.810381098 0.567117731 0.243233358 -1.114890091 0.608915556

At1g74960 KAS2__FAB1 (FATTY ACID BIOSYNTHESIS 1); 3-oxoacyl-[acyl-carrier-protein] synthase/ fatty-acid synthase 0.485777859 -0.621282581 1.080203042 -0.554594121 -0.563724853 -0.128508861 -0.530780267 -0.547689258

At1g74710 EDS16_ICS1_SID2__isochorismate synthase 1 (ICS1) / isochorismate mutase -0.147414107 -0.228028396 -1.461800889 1.972200261 -0.489933431 -1.039900715 -1.19080524 0.640383283

At1g53830 ATPME2; pectinesterase -0.78285853 1.549172395 -0.055639052 -1.665517801 0.527037728 -0.421534421 0.258330296 0.705222422

At1g53840 ATPME1; pectinesterase 0.226140515 -0.325762681 0.991941004 -1.239661919 -0.053325166 -1.025966573 0.145054314 1.086931871

At1g68560 "ATXYL1__XYL1 (ALPHA-XYLOSIDASE 1); alpha-N-arabinofuranosidase/ hydrolase, hydrolyzing O-glycosyl compounds / xylan 1,4-beta-xylosidase" 1.320025624 -0.48402387 1.298753075 -1.581848649 1.991955497 0.804656031 -0.016784305 2.360405463

At1g48320 thioesterase family protein 0.263990855 0.050517367 -1.354181932 0.070234297 -1.768631621 -0.893175105 -0.745104127 -0.219090384

At1g70980 SYNC3; ATP binding / aminoacyl-tRNA ligase/ asparagine-tRNA ligase/ aspartate-tRNA ligase/ nucleic acid binding / nucleotide binding 0.683059169 0.516257777 0.267201727 0.47414153 0.242638283 0.402726319 -0.589239569 0.096378628

At1g70820 "phosphoglucomutase, putative / glucose phosphomutase, putative" -0.996670172 -2.210627747 0.649589026 0.28402853 4.559352602 -0.933443211 0.484863144 0.660837122

At1g64190 6-phosphogluconate dehydrogenase family protein -0.285727601 0.683363711 0.241880918 -0.958556267 -0.168275575 0.813537648 -0.665806706 0.97202808

At1g72970 EDA17__HTH (HOTHEAD); FAD binding / aldehyde-lyase/ mandelonitrile lyase 3.300355232 -1.336266251 2.715420449 0.65117663 -1.049136121 0.32517251 0.318057013 0.739048747

At1g72810 "threonine synthase, putative" 1.027240886 0.056389209 0.654843264 -0.935111093 0.179133582 1.306447352 -0.716088024 1.184254901

At1g49530 GGPS6 (geranylgeranyl pyrophosphate synthase 6); farnesyltranstransferase 0.361840129 0.156681398 0.219529637 0.50828471 0.194432911 -1.152357673 0.382082535 -0.154017893

At1g49430 LRD2_LRD2__LACS2 (LONG-CHAIN ACYL-COA SYNTHETASE 2); long-chain-fatty-acid-CoA ligase 1.998674757 -0.132572525 1.081702482 -1.023904874 -0.99541249 1.225384848 -0.468087594 -0.657884906

At1g49390 "oxidoreductase, 2OG-Fe(II) oxygenase family protein" -0.581933741 1.185380716 0.248976641 -0.44439748 0.167315553 -0.302193985 -0.232980181 -1.472375968

At1g50170 ATSIRB (ARABIDOPSIS THALIANA SIROHYDROCHLORIN FERROCHELATASE B); sirohydrochlorin ferrochelatase -0.702903092 -0.249149479 0.072829821 0.33904383 0.755928583 0.21149678 -0.06586955 -0.190723082

At1g47420 "FUNCTIONS IN: molecular_function unknown; INVOLVED IN: biological_process unknown; LOCATED IN: mitochondrion, nucleus; EXPRESSED IN: 26 plant structures; EXPRESSED DURING: 15 growth stages; BEST Arabidopsis thaliana protein match is: GAMMA CA2 (GAMMA CARB" 0.17028699 0.407554769 -0.058510997 -0.183806916 0.142799309 0.393508334 0.089359178 0.856165883

At1g50200 "ACD__ALATS (ALANYL-TRNA SYNTHETASE); ATP binding / alanine-tRNA ligase/ ligase, forming aminoacyl-tRNA and related compounds / nucleic acid binding / nucleotide binding" 0.53329724 -0.051926513 -0.152149172 -0.290482381 -0.074895207 0.254098429 -0.940405681 0.185484348

At1g17020 "ATSRG1__SRG1 (SENESCENCE-RELATED GENE 1); oxidoreductase, acting on diphenols and related substances as donors, oxygen as acceptor / oxidoreductase, acting on paired donors, with incorporation or reduction of molecular oxygen, 2-oxoglutarate as one donor," -0.15399702 0.648520814 -4.079181292 -0.894464761 -0.792078079 0.83654952 0.017987621 0.70097772

At1g21640 ATNADK2__NADK2; NAD+ kinase/ calmodulin binding -0.579225379 -0.720285957 -0.646734336 -0.601062984 0.135599949 -0.101555464 0.117794497 -0.219519538

At1g17050 SPS2 (Solanesyl diphosphate synthase 2); trans-octaprenyltranstransferase -1.713229013 -1.20189314 -0.475624099 -0.333141284 -3.490118377 0.9141602 -0.245930912 0.004963548

At1g17010 "oxidoreductase, 2OG-Fe(II) oxygenase family protein" -0.030392718 0.473846289 0.071292724 0.647785958 0.439711556 -0.492477853 -0.044981989 -2.353675244

At1g17260 "AHA10 (Autoinhibited H(+)-ATPase isoform 10); ATPase/ ATPase, coupled to transmembrane movement of ions, phosphorylative mechanism / cation-transporting ATPase" 0.317243124 0.120938481 0.30689368 -0.22502905 -0.164004772 0.151375075 0.643908797 -0.481358448

At1g17000 "TPS3__ATTPS3; alpha,alpha-trehalose-phosphate synthase (UDP-forming)" 0.048046238 0.24797036 0.155378919 0.242907782 0.247757397 -0.098176695 0.09743759 -0.444982839

At1g31330 PSAF (photosystem I subunit F) 0.593469335 -3.181781928 0.081244326 -0.670464183 -0.027884098 -0.33858319 -0.838058533 0.275100843

At1g15110 phosphatidyl serine synthase family protein 1.006686837 0.752329962 -0.903420404 -0.470217732 -0.268557739 -0.226171294 -0.370989948 -0.463400593

At1g15080 ATLPP2_ATPAP2__LPP2 (LIPID PHOSPHATE PHOSPHATASE 2); acid phosphatase/ phosphatidate phosphatase 0.347643186 0.18785613 0.855191592 0.410251167 0.376899875 0.103503868 -0.360700228 -0.110383599

At1g14120 "2-oxoglutarate-dependent dioxygenase, putative" -0.820226111 2.018092673 -0.269748468 -1.72462566 -1.335769414 -0.959112681 -1.700273677 1.583118869

At1g14150 oxygen evolving enhancer 3 (PsbQ) family protein -2.758263106 -3.249111963 1.067168584 0.616190269 0.419963455 0.079439743 0.725800353 0.529085952

At1g06550 enoyl-CoA hydratase/isomerase family protein 1.499682414 0.240623316 0.015347082 -0.972218138 -1.868433002 1.946423694 -0.782307027 1.437152351

At1g06520 ATGPAT1__GPAT1 (GLYCEROL-3-PHOSPHATE ACYLTRANSFERASE 1); 1-acylglycerol-3-phosphate O-acyltransferase/ acyltransferase 1.222068985 -0.310481688 -1.453348907 -1.541724612 -0.635925588 -0.378680618 1.737326146 -0.621094532

At1g06680 OEE2_PSII-P__PSBP-1 (PHOTOSYSTEM II SUBUNIT P-1); poly(U) binding 0.442935509 -3.307922666 -0.222036683 -0.740124103 -0.130562659 -0.056684916 -0.804454347 0.368052761

At1g06570 HPD__PDS1 (PHYTOENE DESATURATION 1); 4-hydroxyphenylpyruvate dioxygenase 0.748959408 -1.713088175 -2.991001706 -2.398817824 -2.354453867 -2.379681619 -0.568194674 -0.753062105

At1g62800 "ASP4 (ASPARTATE AMINOTRANSFERASE 4); catalytic/ pyridoxal phosphate binding / transaminase/ transferase, transferring nitrogenous groups" -2.717298844 0.986615265 -0.860214597 -3.124671921 -0.362861827 0.492189217 0.091220661 0.686490876

At1g14130 "2-oxoglutarate-dependent dioxygenase, putative" 0.885783287 0.460449764 -2.013303259 -0.148274452 -0.594346206 0.108131057 -0.476955735 -0.348152742

At1g62830 ATSWP1_SWP1__LDL1 (LSD1-LIKE1); amine oxidase/ electron carrier/ oxidoreductase 0.826632504 0.005133915 0.481580206 -0.275855122 0.240185803 0.925545834 -0.969021684 -0.447936663

At1g16460 ATMST2_MST2__ATRDH2 (ARABIDOPSIS THALIANA RHODANESE HOMOLOGUE 2); 3-mercaptopyruvate sulfurtransferase/ thiosulfate sulfurtransferase -0.06673704 -0.528426133 -0.195881615 -1.302852569 0.474495038 0.566247488 -0.6084862 0.209285785

At1g16410 "BUS1_SPS1__CYP79F1 (CYTOCHROME P450 79F1); oxidoreductase, acting on paired donors, with incorporation or reduction of molecular oxygen, NADH or NADPH as one donor, and incorporation of one atom of oxygen" -3.221499953 0.026362552 0.698023697 0.026552845 0.526755723 0.832314029 -0.054597711 -0.078883572

At1g16340 ATKSDA__ATKDSA2; 3-deoxy-8-phosphooctulonate synthase 0.651658921 0.191470558 1.211937592 -0.18531351 1.080308475 0.669731865 -0.441567504 0.499204661

At1g16350 "inosine-5'-monophosphate dehydrogenase, putative" 0.645318269 0.109060325 1.178534842 -0.528869181 -0.114782679 0.772492932 -1.56847381 0.233465607

At1g10700 ribose-phosphate pyrophosphokinase 3 / phosphoribosyl diphosphate synthetase 3 (PRS3) -0.102610683 0.622211106 0.348036399 1.354908797 0.288446546 -1.999871395 -0.229404382 0.519147113

At1g20950 pyrophosphate--fructose-6-phosphate 1-phosphotransferase-related / pyrophosphate-dependent 6-phosphofructose-1-kinase-related 0.497533675 0.55675703 1.49262553 -0.526952117 -0.995749894 1.194430059 -1.115546278 1.431445668

At1g11720 "ATSS3 (starch synthase 3); starch synthase/ transferase, transferring glycosyl groups" -0.150909912 -0.967627789 0.422177536 0.741432124 -0.529827798 0.316551506 -0.473645572 -1.515101362

At1g11790 ADT1 (arogenate dehydratase 1); arogenate dehydratase/ prephenate dehydratase -0.146156009 -0.017542644 0.926274517 0.889250143 -0.60424728 0.152593752 -0.728119112 -0.819882804

At1g14810 semialdehyde dehydrogenase family protein -0.21598465 0.289820039 1.488271777 0.904188248 -0.24563449 -0.085825775 -1.265346062 0.518988607

At1g64910 glycosyltransferase family protein -0.251904235 0.759516636 0.532892564 -0.06568877 0.116340888 0.143485122 0.044684668 -0.005662213

At1g64920 glycosyltransferase family protein -0.266919823 0.8515603 0.408558687 -0.099640058 0.02160537 0.040591769 0.016523673 -0.009201563

At1g64710 "alcohol dehydrogenase, putative" -0.510752313 -0.317536356 -0.488917754 1.607972457 0.970318042 -0.648789768 -1.3203388 -0.926379466

At1g64970 TMT1_VTE4__G-TMT (GAMMA-TOCOPHEROL METHYLTRANSFERASE); tocopherol O-methyltransferase 0.681680616 -1.745729331 -0.963148087 0.054665354 -2.155598333 1.36018421 0.977811935 0.907003312

At1g79460 ATKS_KS__GA2 (GA REQUIRING 2); ent-kaurene synthase 1.108211073 0.242654022 0.655435032 -0.047977225 2.005909757 0.377689763 -0.120577155 -1.013859502

At1g79440 SSADH_SSADH1__ALDH5F1; 3-chloroallyl aldehyde dehydrogenase/ NAD or NADH binding / succinate-semialdehyde dehydrogenase -0.572671453 0.998681451 -0.212831224 1.020108504 5.498826768 0.399341148 0.102755477 -0.123775172

At1g59900 "AT-E1 ALPHA; oxidoreductase, acting on the aldehyde or oxo group of donors, disulfide as acceptor / pyruvate dehydrogenase (acetyl-transferring)" 0.112296878 -0.384461218 -0.461222855 -0.928211821 0.80494546 -0.045358411 -0.290008968 0.197221715

At1g79500 AtkdsA1__2-dehydro-3-deoxyphosphooctonate aldolase / phospho-2-dehydro-3-deoxyoctonate aldolase / 3-deoxy-D-manno-octulosonic acid 8-phosphate synthetase (KDSA) 0.541767254 0.182317847 1.410409273 -0.354735825 2.02041569 0.149230287 -0.006815514 0.378426359

At1g65840 PAO4__ATPAO4 (ARABIDOPSIS THALIANA POLYAMINE OXIDASE 4); amine oxidase/ polyamine oxidase -0.711729221 1.515361168 0.794816845 0.932674165 -1.29759881 -0.408776765 0.69758382 -0.170273185

At1g79530 GAPCP-1 (GLYCERALDEHYDE-3-PHOSPHATE DEHYDROGENASE OF PLASTID 1); NAD or NADH binding / binding / catalytic/ glyceraldehyde-3-phosphate dehydrogenase (phosphorylating)/ glyceraldehyde-3-phosphate dehydrogenase 1.851114034 1.946248842 0.445410821 -0.214632928 -0.97443628 1.027803917 -0.848499868 1.609355232

At1g79470 inosine-5'-monophosphate dehydrogenase 0.316775037 1.45391524 0.708178603 0.687620118 -0.347613449 0.980352813 -0.607443055 -0.165131894

At1g79550 PGK (PHOSPHOGLYCERATE KINASE); phosphoglycerate kinase 0.082485849 0.603224857 -0.174591411 -0.071787874 -0.635380313 1.117305328 -0.356630395 0.334892305

At1g75790 sks18 (SKU5 Similar 18); copper ion binding / pectinesterase 1.240388862 -0.11234501 0.341193895 0.067377292 -0.232487838 0.131854501 1.163099784 0.290916159

At1g54220 "dihydrolipoamide S-acetyltransferase, putative" -1.2175873 0.177625056 0.552825734 0.598298591 -0.114927155 1.043548564 0.041811014 0.267871481

At1g75680 "AtGH9B7 (Arabidopsis thaliana glycosyl hydrolase 9B7); catalytic/ hydrolase, hydrolyzing O-glycosyl compounds" 0.268649066 -0.140038667 1.897227771 -1.865967487 -0.336865238 0.251528613 -0.730956056 -0.001140073

At1g23310 AOAT1__GGT1 (GLUTAMATE:GLYOXYLATE AMINOTRANSFERASE); L-alanine:2-oxoglutarate aminotransferase/ glycine:2-oxoglutarate aminotransferase -0.728206409 -2.450783199 -0.650821339 -0.333819166 -0.583777378 -0.12952538 -0.459980144 1.047305123

At1g23870 "TPS9__ATTPS9; transferase, transferring glycosyl groups / trehalose-phosphatase" 0.604474552 0.210906465 -0.075425444 -2.197906343 0.159930825 -3.132403903 -0.692225574 0.627526915

At1g23360 UbiE/COQ5 methyltransferase family protein 0.088701422 -0.849171455 0.662757167 0.324664153 0.513953506 -0.006032881 -0.263574122 0.615498858

At2g17630 "phosphoserine aminotransferase, putative" 0.329353873 1.255885191 1.863904411 0.375759667 0.156282133 0.748415852 -0.277368416 0.812607225

At1g03130 PSAD-2 (photosystem I subunit D-2) -0.283063338 -3.977001636 0.234657526 -1.154038437 0.203741709 -1.323195462 -0.460845873 -0.550474276

At1g03090 MCCA; methylcrotonoyl-CoA carboxylase 1.253467661 -0.701906493 -0.855911418 -3.150809396 1.381406766 -3.058920236 -0.529731502 0.554719538

At1g78510 SPS1 (solanesyl diphosphate synthase 1); trans-octaprenyltranstransferase -1.173650614 -1.125821205 -1.180204384 -0.157902127 -2.351871621 1.457781514 0.091640426 -0.262125212

At1g78500 "pentacyclic triterpene synthase, putative" -0.06220366 0.265433136 0.013655685 -0.240929387 -0.050309757 0.137913342 -0.78635899 -4.103269851

At1g78570 "ATRHM1_ROL1__RHM1 (RHAMNOSE BIOSYNTHESIS 1); UDP-L-rhamnose synthase/ UDP-glucose 4,6-dehydratase/ catalytic" 0.67194183 0.559209983 0.081178588 1.231820495 -2.273324002 2.391123022 -0.257533983 1.327460887

At1g78550 "oxidoreductase, 2OG-Fe(II) oxygenase family protein" 0.151957903 -0.10204784 -0.796024256 -0.706754901 0.678093324 0.014185222 -0.700298226 0.070680953

At1g78580 "TPS1__ATTPS1 (TREHALOSE-6-PHOSPHATE SYNTHASE); alpha,alpha-trehalose-phosphate synthase (UDP-forming)/ transferase, transferring glycosyl groups" 0.298735801 -0.016163817 0.880940574 0.002338725 -1.039987537 0.931975092 -0.169707032 -1.19996477

At1g78660 "ATGGH1__gamma-glutamyl hydrolase, putative / gamma-Glu-X carboxypeptidase, putative / conjugase, putative" 0.141015207 0.948263877 0.077052146 -0.529586705 0.709469686 -0.736054019 -0.871882868 2.130117481

At1g54100 ALDH7B4 (Aldehyde Dehydrogenase 7B4); 3-chloroallyl aldehyde dehydrogenase/ oxidoreductase 1.670653767 0.125880623 -3.133451101 -1.217155621 1.233467085 -0.542112682 -0.665072537 -0.634197934

At1g54040 ESR_TASTY__ESP (EPITHIOSPECIFIER PROTEIN); enzyme regulator 0.650168303 -1.816214313 0.547207209 2.461583942 0.730735175 0.968331026 0.211987745 0.479242351

At1g05530 "UGT2__UGT75B2 (UDP-GLUCOSYL TRANSFERASE 75B2); UDP-glucosyltransferase/ UDP-glycosyltransferase/ abscisic acid glucosyltransferase/ transferase, transferring glycosyl groups" -0.076675705 0.468422446 0.28244288 0.159791506 0.134756072 -0.161755219 0.162806542 -0.170404721

At1g05620 URH2 (URIDINE-RIBOHYDROLASE 2); hydrolase 0.669007927 1.059823442 0.055297614 -0.019845446 0.491139774 0.17097686 -0.010454208 0.65856477

At1g05560 "UGT1__UGT75B1 (UDP-GLUCOSYLTRANSFERASE 75B1); UDP-glucose:4-aminobenzoate acylglucosyltransferase/ UDP-glucosyltransferase/ UDP-glycosyltransferase/ abscisic acid glucosyltransferase/ transferase, transferring glycosyl groups" 0.294025772 -0.724300561 -3.335604281 1.051791058 0.741938421 0.277342992 -0.371450155 1.205664913

At1g36160 AT-ACC1_EMB22_GK_PAS3__ACC1 (ACETYL-COENZYME A CARBOXYLASE 1); acetyl-CoA carboxylase 1.163821342 -0.425870394 -0.493818397 -1.014879478 -0.97377714 1.149170886 -0.87080595 0.371350518

At1g05610 APS2 (ADP-glucose pyrophoshorylase small subunit 2); glucose-1-phosphate adenylyltransferase/ nucleotidyltransferase/ transferase 0.020190359 0.264786308 0.298410076 0.158038605 0.231252335 -0.426427042 0.213954668 -0.242794301

At1g30620 HSR8_UXE1__MUR4 (MURUS 4); UDP-arabinose 4-epimerase/ catalytic 1.791127202 1.036703991 -0.804459414 2.678184218 0.217928284 -0.979782329 -0.074871198 -0.014326551

At1g05650 "polygalacturonase, putative / pectinase, putative" -0.439371995 1.16453304 0.349118329 -0.626474889 0.129443022 -0.180437065 -0.099426545 0.327172654

At1g05670 UDP-glucoronosyl/UDP-glucosyl transferase family protein 0.670453767 0.257914166 0.072475834 1.00346734 0.187467896 -0.122675522 -0.25663161 -0.066988

At1g05680 UDP-glucoronosyl/UDP-glucosyl transferase family protein 2.61969219 -0.507498626 -4.390181529 0.874137052 -1.812416678 -1.310063676 -1.037480529 1.685508004

At2g16570 ATASE1 (GLN PHOSPHORIBOSYL PYROPHOSPHATE AMIDOTRANSFERASE 1); amidophosphoribosyltransferase 1.323707903 1.275491696 0.672826012 0.424560635 -0.237404105 0.325098286 -0.789379818 -0.845844332

At2g16500 ARGDC_ARGDC1_SPE1__ADC1 (ARGININE DECARBOXYLASE 1); arginine decarboxylase -0.659741512 0.25127214 0.373926642 1.17377995 -2.129339788 0.05847363 0.807659952 -0.28301378

At2g31350 GLX2-5 (GLYOXALASE 2-5); hydroxyacylglutathione hydrolase/ iron ion binding / zinc ion binding -0.494670763 0.727215309 -1.316177345 -0.471838794 0.399143149 -0.509237042 0.73991014 -0.942582508

At2g31360 ADS2 (16:0Delta9 Arabidopsis desaturase 2); oxidoreductase -0.00762457 0.743749497 1.351069583 0.74049931 3.280515937 -0.175236865 -0.314779439 -1.223950898

At2g31390 pfkB-type carbohydrate kinase family protein 0.70919152 1.487344744 -0.984914997 -0.511753783 -0.18863271 0.876419616 -0.48615027 0.929033904

At2g14170 ALDH6B2; 3-chloroallyl aldehyde dehydrogenase/ methylmalonate-semialdehyde dehydrogenase (acylating)/ oxidoreductase 0.855420077 0.07383234 -0.574618037 -1.719135713 2.152451479 0.013087056 -0.741365932 0.540130015

At2g47180 "AtGolS1 (Arabidopsis thaliana galactinol synthase 1); transferase, transferring glycosyl groups / transferase, transferring hexosyl groups" 0.014121453 0.047595168 -0.528379614 -1.678312509 -3.184943988 -3.275154155 -0.742778675 -1.601345352

At2g05710 "aconitate hydratase, cytoplasmic, putative / citrate hydro-lyase/aconitase, putative" 0.257553167 0.604661263 -1.182653091 0.393992779 0.186199497 0.998581972 -0.326565311 0.584922803

At2g13360 AGT1__AGT (ALANINE:GLYOXYLATE AMINOTRANSFERASE); alanine-glyoxylate transaminase/ serine-glyoxylate transaminase/ serine-pyruvate transaminase -1.689601668 -2.825570431 -0.9880167 -1.246807724 0.410574816 -0.513166082 0.155498042 0.49755468

At2g15230 ATLIP1 (Arabidopsis thaliana lipase 1); galactolipase/ hydrolase/ phospholipase/ triacylglycerol lipase -0.260236173 0.389458664 -0.535721185 -0.947190359 0.677502978 -0.041828759 -0.152986823 -0.295511545

At2g11810 "ATMGD3_MGD3__MGDC; 1,2-diacylglycerol 3-beta-galactosyltransferase" -0.620567804 0.225691817 -0.670010741 -0.290807153 0.438836592 -0.08194875 1.018227005 -0.154708478

At2g31490 unknown protein -0.16226285 0.405036419 0.484863811 -0.16955997 0.139840209 0.435016991 -0.009174104 0.136170008

At2g31570 ATGPX2 (GLUTATHIONE PEROXIDASE 2); glutathione peroxidase 0.672283843 -0.558902185 -1.706497439 -1.671304193 -1.19539047 0.38934135 -0.798215493 0.929110532

At2g22230 "beta-hydroxyacyl-ACP dehydratase, putative" -0.060744898 -0.013877944 2.18270024 -0.422040916 -0.18203765 0.592386064 -0.629156471 -0.415626376

At2g22240 ATMIPS2__MIPS2 (MYO-INOSITOL-1-PHOSTPATE SYNTHASE 2); binding / catalytic/ inositol-3-phosphate synthase 2.63468958 -1.223010745 -0.500662034 -1.049146489 -3.284059314 -0.022011934 -0.137071135 -2.442866419

At2g31690 lipase class 3 family protein 0.214006693 0.252671977 0.201446231 0.506072687 0.14062865 -0.591059261 0.00676008 -0.383519246

At2g22190 catalytic/ trehalose-phosphatase -1.104383776 0.046714713 0.184847634 0.689749274 1.175263849 0.499544218 0.504100571 0.169500535

At2g31750 "UGT74D1 (UDP-glucosyl transferase 74D1); UDP-glycosyltransferase/ abscisic acid glucosyltransferase/ transferase, transferring glycosyl groups / transferase, transferring hexosyl groups" -0.952925361 0.046763594 -0.049405176 -1.045099069 -2.032214209 0.860968604 0.377508762 1.957933811

At2g31790 UDP-glucoronosyl/UDP-glucosyl transferase family protein -1.518124018 -0.334397089 0.938147172 -0.090494914 -0.087261225 1.364207874 -0.096501501 1.329173133

At2g07687 cytochrome c oxidase subunit 3 0.838435705 0.155377736 -0.738390575 0.094558561 0.448311112 -0.772424375 -0.190564151 -0.730055742

At2g21610 pectinesterase family protein -0.016477499 0.476614406 0.379991371 0.239237611 0.209561597 -0.274189172 0.306024739 -0.475889372

At2g21590 APL4; glucose-1-phosphate adenylyltransferase 2.225607154 -0.301712422 -0.089049373 1.251512244 0.721971813 1.017961298 0.250984742 0.21385403

At2g21550 "bifunctional dihydrofolate reductase-thymidylate synthase, putative / DHFR-TS, putative" -0.356741011 1.174864078 0.330126079 -0.040965986 0.121209182 -0.15820343 -0.497982778 0.220044138

At2g27150 AAO3 (Abscisic ALDEHYDE OXIDASE 3); abscisic aldehyde oxidase/ aldehyde oxidase/ indole-3-acetaldehyde oxidase -0.126785793 -0.343044046 -2.306452542 0.258090665 -0.080116101 -0.448943218 -0.208348074 0.060178781

At2g17130 "IDH2 (ISOCITRATE DEHYDROGENASE SUBUNIT 2); isocitrate dehydrogenase (NAD+)/ oxidoreductase, acting on the CH-OH group of donors, NAD or NADP as acceptor" -0.63563179 0.77298125 -0.886640612 0.34335206 0.051626475 0.449427195 -0.248281841 1.787979243

At2g16370 THY-1 (THYMIDYLATE SYNTHASE 1); dihydrofolate reductase/ thymidylate synthase 0.508562073 0.433876815 1.057784062 0.096224659 0.54129481 0.740733641 -0.223103456 1.071128918

At2g16280 "KCS9 (3-KETOACYL-COA SYNTHASE 9); acyltransferase/ catalytic/ transferase, transferring acyl groups other than amino-acyl groups" -0.584719943 -0.798964213 1.042449159 -0.443656214 -0.347937986 -0.576832563 -0.569683899 -0.490395662

At1g04410 "malate dehydrogenase, cytosolic, putative" 0.061501824 -0.020694202 -0.432932861 -0.919061046 0.38480058 0.474747528 -0.890781138 1.241947398

At1g31220 phosphoribosylglycinamide formyltransferase -0.128232288 0.33912866 0.262984907 0.910810871 0.531277636 0.364266549 -0.353946328 -0.701452584

At1g31230 AK-HSDH I (ASPARTATE KINASE-HOMOSERINE DEHYDROGENASE I); aspartate kinase/ homoserine dehydrogenase -0.818045489 0.162892914 1.110130554 0.349174223 0.381028051 1.471908822 -0.782816659 -0.250578306

At1g31190 "IMPL1 (MYO-INOSITOL MONOPHOSPHATASE LIKE 1); 3'(2'),5'-bisphosphate nucleotidase/ inositol or phosphatidylinositol phosphatase/ inositol-1(or 4)-monophosphatase" -1.246818139 -0.969788526 0.440628234 0.56293407 -0.219091143 0.310094431 0.257026596 -0.282636501

At1g31180 "3-isopropylmalate dehydrogenase, chloroplast, putative" -2.322123946 0.169432743 1.548528115 -0.375904544 -0.249351701 0.951380839 -0.958608867 1.135597294

At2g20610 ALF1_HLS3_RTY_RTY1__SUR1 (SUPERROOT 1); S-alkylthiohydroximate lyase/ carbon-sulfur lyase/ transaminase -1.11255519 0.053483499 -0.126548585 0.234617152 -0.323975637 0.069216002 -1.173850694 1.52306846

At1g60090 "BGLU4 (BETA GLUCOSIDASE 4); catalytic/ cation binding / hydrolase, hydrolyzing O-glycosyl compounds" -0.240913629 0.156787169 0.573876376 0.240115613 0.034726199 -0.192774239 0.045835368 -0.413707318

At2g21330 "fructose-bisphosphate aldolase, putative" -0.636749106 -4.158003556 0.250796743 0.635610014 -1.304335593 0.434431265 -0.447064992 -0.460091076

At2g46390 unknown protein -0.030190303 0.565452392 -0.452597053 -0.118781588 0.363954646 0.644839577 0.222235573 0.533727945

At2g46370 FIN219__JAR1 (JASMONATE RESISTANT 1); ATP binding / adenylyltransferase/ catalytic/ jasmonate-amino synthetase -0.766161819 0.31653099 -0.492426815 -0.679696133 -0.160714559 0.242709277 -0.393476893 -0.551611443

At2g04400 indole-3-glycerol phosphate synthase (IGPS) 0.317505389 0.576793484 -0.891353794 2.218032598 -0.18131439 -0.504259241 -1.412468853 0.734894763

At2g04350 long-chain-fatty-acid--CoA ligase family protein / long-chain acyl-CoA synthetase family protein (LACS8) -0.359969216 -0.138274438 -1.958172709 0.526414809 -0.595150551 0.8318248 0.245595524 -0.069723155

At2g36880 MAT3 (methionine adenosyltransferase 3); methionine adenosyltransferase -0.067970238 0.855233446 0.929135144 -0.056632499 -1.52322373 0.63293721 0.101734703 1.06008031

At2g37040 pal1 (Phe ammonia lyase 1); phenylalanine ammonia-lyase 0.321615148 0.354461971 -0.5557047 -0.750721578 -4.111355754 0.178167257 -0.791604146 0.917344081

At2g36970 UDP-glucoronosyl/UDP-glucosyl transferase family protein -1.311124022 -0.593487183 -1.873343035 1.745991624 0.439639189 0.634990767 -0.053932591 -0.122614808

At2g04540 "3-oxoacyl-(acyl-carrier-protein) synthase II, putative" 0.210486776 0.125373797 0.391023142 0.061858293 0.639578243 -0.073396621 -0.293819961 -0.073289104

At2g21770 "CESA09__CESA9 (CELLULOSE SYNTHASE A9); cellulose synthase/ transferase, transferring glycosyl groups" -0.063295633 0.082411028 0.318889491 -0.102346374 0.160401825 0.058050344 0.592036443 -1.703683674

At2g21790 CLS8_R1__RNR1 (RIBONUCLEOTIDE REDUCTASE 1); ATP binding / protein binding / ribonucleoside-diphosphate reductase 1.36637824 0.63819485 1.774240321 1.393531812 0.472295172 1.290757043 -1.379618031 0.665989517

At2g21940 "shikimate kinase, putative" -0.105746779 0.193811503 -1.340390122 -0.651798048 0.626313982 0.227294557 0.384929266 -0.385998614

At2g36230 HISN3__APG10 (ALBINO AND PALE GREEN 10); 1-(5-phosphoribosyl)-5-[(5-phosphoribosylamino)methylideneamino]imidazole-4-carboxamide isomerase -0.041345326 -0.023653448 1.709284674 0.755923503 0.966290412 0.704082499 -0.690522499 0.009675181

At2g36390 "BE3__SBE2.1 (starch branching enzyme 2.1); 1,4-alpha-glucan branching enzyme" -0.197192481 0.03537965 -0.074149429 1.298247795 3.319846801 1.388752726 0.736895536 0.002078003

At2g36460 "fructose-bisphosphate aldolase, putative" 0.000623838 0.043399058 -1.188184731 0.010899571 1.645290494 0.545600735 -0.601208489 -0.518246883

At2g36580 "pyruvate kinase, putative" -0.017334116 0.785905545 -0.640907993 1.028119896 0.974529672 1.029150673 -0.484665246 0.737625121

At2g36530 LOS2; copper ion binding / phosphopyruvate hydratase 0.254058946 0.646639975 0.084294242 0.157265527 -0.320184404 0.757162951 -0.66407791 0.647063584

At2g21890 CAD3 (CINNAMYL ALCOHOL DEHYDROGENASE HOMOLOG 3); binding / catalytic/ oxidoreductase/ zinc ion binding 0.314402297 -0.003218705 0.480379323 0.486189122 0.082950737 0.156704431 1.227055 -0.019605641

At2g36310 URH1 (URIDINE-RIBOHYDROLASE 1); adenosine nucleosidase/ hydrolase/ inosine nucleosidase/ uridine nucleosidase 0.122135173 0.442205403 0.306602456 -0.822243068 -0.379826512 -1.178086789 0.157458134 0.693103434

At2g35840 sucrose-phosphatase 1 (SPP1) -0.919757107 -0.956723654 -0.460522465 0.077151941 -0.744740806 0.868641202 1.118650146 -0.031662292

At2g42790 CSY3 (citrate synthase 3); citrate (SI)-synthase -0.384968951 0.273805961 -2.881505492 -0.61378715 2.113956863 0.348518086 -0.638408351 -0.910107156

At2g42690 "lipase, putative" -1.590752633 -1.746743413 -0.158566054 -0.099591773 0.057419454 0.77153869 -0.617474852 1.531750004

At2g21170 TIM (TRIOSEPHOSPHATE ISOMERASE); catalytic/ triose-phosphate isomerase -0.18480492 -1.077716232 0.43984275 -0.81654977 -0.123429514 -0.007182006 -0.567425612 0.977401263

At2g22480 PFK5 (PHOSPHOFRUCTOKINASE 5); 6-phosphofructokinase -0.426552202 0.58512778 -0.726862904 -0.104014135 0.479438667 0.57559619 -0.067762599 1.241709062

At2g22450 "riboflavin biosynthesis protein, putative" 0.142784484 0.607118936 -0.301847043 0.773388145 6.183798818 0.69912027 -0.894336017 -0.62712038

At2g22330 CYP79B3; electron carrier/ heme binding / iron ion binding / monooxygenase/ oxygen binding -2.023725762 0.799694566 0.554003306 -0.004599389 -0.608277285 -2.045801589 -1.266153469 2.397284121

At2g31250 HEMA3; NADP or NADPH binding / binding / catalytic/ glutamyl-tRNA reductase/ shikimate 5-dehydrogenase -0.116790836 0.391840265 0.448855126 0.375430663 0.25558033 -0.520911909 0.622768109 -0.38310867

At1g79040 PSBR (photosystem II subunit R) 0.314823154 -2.671495429 -0.898773542 -1.002356596 -0.267891864 0.177490121 -0.735661519 0.046753116

At1g79230 ATMST1_ATRDH1_ST1_STR1__MST1 (MERCAPTOPYRUVATE SULFURTRANSFERASE 1); 3-mercaptopyruvate sulfurtransferase/ sulfurtransferase/ thiosulfate sulfurtransferase -0.192490609 0.178688824 -0.14906088 0.014248358 0.116138149 0.778821939 -0.205736992 -0.610633366

At1g79010 "NADH-ubiquinone oxidoreductase 23 kDa subunit, mitochondrial (TYKY)" -0.039372523 0.447722562 0.003264848 -0.189703457 0.274138638 0.48454574 -0.1424723 0.419538476

At1g78970 ATLUP1__LUP1 (LUPEOL SYNTHASE 1); beta-amyrin synthase/ lupeol synthase -0.585588803 -1.658915645 -0.57317935 -2.246151355 0.813863299 -0.064418474 2.212287598 1.009876827

At1g78960 ATLUP2; beta-amyrin synthase/ lupeol synthase 2.67570581 -0.935876979 -1.485958362 -0.903702053 -0.060999504 0.413524924 1.838955337 1.815957615

At1g78950 "beta-amyrin synthase, putative" 0.728568562 -0.368660549 -0.516122912 -2.038221289 -0.099284311 -0.064870516 1.794949412 0.781824152

At1g02205 CER1 (ECERIFERUM 1); octadecanal decarbonylase 3.536821568 -2.662425021 -0.998813976 -0.582998665 1.128899542 1.036954507 2.001901308 1.737606575

At1g02190 "CER1 protein, putative" 4.822696104 -0.621498385 0.030975892 0.258371859 0.007732558 0.490724424 1.142646955 1.455659444

At1g22650 "beta-fructofuranosidase, putative / invertase, putative / saccharase, putative / beta-fructosidase, putative" -0.788509 0.293433816 1.049496277 1.404338787 -0.643673096 -0.913296998 0.403414385 -0.132263621

At1g60420 DC1 domain-containing protein 0.6490572 0.394377812 -1.342017392 -0.418716854 0.642081069 0.488269267 -0.434278328 0.634443131

At1g60440 ATCOAA__ATPANK1 (PANTOTHENATE KINASE 1); pantothenate kinase 0.4499566 0.143541227 -0.525476034 0.376848536 0.141125785 -0.015924622 0.002750577 0.850424525

At1g60450 "AtGolS7 (Arabidopsis thaliana galactinol synthase 7); transferase, transferring glycosyl groups / transferase, transferring hexosyl groups" -0.12231765 0.298192252 0.419240894 0.482153077 0.233795349 -0.595532301 0.326686407 -0.666617923

At1g60140 "TPS10__ATTPS10 (trehalose phosphate synthase); transferase, transferring glycosyl groups / trehalose-phosphatase" 0.130706671 0.03114036 -1.65544211 -2.60038676 1.668555633 -2.30355373 -1.228387164 -0.422093494

At1g78920 AVPL1__AVP2 (ARABIDOPSIS VACUOLAR H+-PYROPHOSPHATASE 2); hydrogen-translocating pyrophosphatase 0.280298896 0.411822805 -0.34098145 -0.012437932 0.624343592 0.748158148 -0.499110315 -0.004920413

At1g78680 ATGGH2 (gamma-glutamyl hydrolase 2); omega peptidase 0.37839164 -0.621398628 -1.338904286 -1.295476794 1.580817862 0.666563841 0.267435072 1.269472718

At1g09240 ATNAS3__NAS3 (NICOTIANAMINE SYNTHASE 3); nicotianamine synthase -1.362531765 -0.81440092 -1.976542771 0.089402883 -0.21276577 0.241824799 0.825329191 0.333286895

At1g61850 galactolipase/ phospholipase -0.215702566 0.513097471 0.201347089 0.097284246 0.625141478 -0.312156757 0.874142265 -0.244295726

At1g70410 "ATBCA4_BCA4__carbonic anhydrase, putative / carbonate dehydratase, putative" -3.51026614 -0.642154392 0.797732991 -1.426028815 -0.893320972 -0.746878114 0.904481261 2.380551789

At1g70310 SPDS2 (spermidine synthase 2); spermidine synthase -0.07511864 0.436968301 1.249544236 -0.310017953 0.879021315 0.240576685 -1.285652758 -0.221732686

At1g12010 "1-aminocyclopropane-1-carboxylate oxidase, putative / ACC oxidase, putative" -1.999188517 1.243289631 -0.207804435 -3.078169829 0.844194315 -0.087593346 -1.326870234 -3.681417716

At1g11870 ATSRS_OVA7__SRS (SERYL-TRNA SYNTHETASE); serine-tRNA ligase -0.304594193 -0.393895045 1.177678933 0.589796408 0.061906189 -0.092429278 -0.16409578 -0.209658401

At1g11840 ATGLX1 (GLYOXALASE I HOMOLOG); lactoylglutathione lyase/ metal ion binding 0.494815696 0.567357273 -0.113305789 -0.194621909 -0.371035351 1.282162913 -0.29124397 -0.14693342

At2g25080 ATGPX1 (GLUTATHIONE PEROXIDASE 1); glutathione peroxidase -0.552654687 -2.119164763 0.023179025 -1.90645444 -0.0542475 -1.693161375 -0.168000682 -0.389623744

At1g12000 "pyrophosphate--fructose-6-phosphate 1-phosphotransferase beta subunit, putative / pyrophosphate-dependent 6-phosphofructose-1-kinase, putative" 1.165980754 0.729500027 1.040239135 -0.828181444 -0.91778352 1.092575674 -0.969053587 0.795745577

At1g11860 "aminomethyltransferase, putative" -1.049273679 -1.380685356 1.419143318 -0.051927112 -0.36810211 0.43784121 -0.225163759 0.122707257

At1g12050 "fumarylacetoacetase, putative" 0.616866402 -0.607870858 -1.186570504 -0.825697503 0.634789144 1.079221622 -0.309246716 0.51914733

At1g61720 BAN (BANYULS); oxidoreductase -0.077570617 -0.171039665 0.184440985 -0.937425013 -0.761929566 0.670315614 0.398481292 -2.968956219

At1g61680 ATTPS14__TPS14 (TERPENE SYNTHASE 14); S-linalool synthase 0.7792565 -0.592520308 -1.524007836 -1.190093191 -0.02705386 0.051018857 3.78492649 1.237832067

At1g27450 ATAPT1__APT1 (ADENINE PHOSPHORIBOSYL TRANSFERASE 1); adenine phosphoribosyltransferase 0.341910446 0.440084354 0.344309693 0.08375415 1.102774049 0.808931909 -0.582858469 1.531376654

At1g10400 "UDP-glycosyltransferase/ transferase, transferring glycosyl groups" -0.471486399 0.828558936 0.49195782 0.235959956 0.258160524 -0.238315898 0.138701892 -0.093269364

At1g67110 CYP735A2; electron carrier/ heme binding / iron ion binding / monooxygenase/ oxygen binding -0.727200125 1.557220476 0.917095378 -0.771655241 0.19449535 -0.24648969 0.07191706 0.460891904

At1g67090 RBCS1A (RIBULOSE BISPHOSPHATE CARBOXYLASE SMALL CHAIN 1A); copper ion binding / ribulose-bisphosphate carboxylase 0.784345697 -2.406662921 -0.370484899 -0.127436664 -0.183164707 0.292246828 -0.448344779 0.021639801

At1g09430 ACLA-3; ATP citrate synthase 0.210504441 -0.066587097 -1.102172797 -1.386629512 0.89211419 -0.473213654 -1.281316289 -0.076044663

At1g09350 "AtGolS3 (Arabidopsis thaliana galactinol synthase 3); transferase, transferring glycosyl groups / transferase, transferring hexosyl groups" -0.294541599 0.338964732 0.830579425 1.177941039 -0.423530382 -1.530704989 0.327040094 -1.057036103

At1g09500 cinnamyl-alcohol dehydrogenase family / CAD family 0.157982057 -0.983177974 -4.375026801 0.859739616 -0.829536326 1.083593197 -0.265134495 -2.275202563

At1g10070 ATBCAT-2 (ARABIDOPSIS THALIANA BRANCHED-CHAIN AMINO ACID TRANSAMINASE 2); branched-chain-amino-acid transaminase/ catalytic 0.475292117 -0.230974637 -2.851620544 -1.947328203 1.406212336 -2.729025807 0.727981806 -0.356256504

At1g10060 ATBCAT-1__branched-chain amino acid aminotransferase 1 / branched-chain amino acid transaminase 1 (BCAT1) 2.592091498 -1.283981927 -0.065548193 -0.848442114 1.113751867 0.004541231 1.028468376 -0.724569249

At1g30820 "CTP synthase, putative / UTP--ammonia ligase, putative" 3.056109157 0.416108886 1.343590796 -0.929835444 -0.071575182 -1.968527019 -0.777621529 0.843285064

At1g55740 "AtSIP1 (Arabidopsis thaliana seed imbibition 1); hydrolase, hydrolyzing O-glycosyl compounds" -0.501548232 1.066248484 -0.216165931 -0.888912073 -0.328235342 0.394311534 -0.165589371 -0.628570943

At1g55670 PSAG (PHOTOSYSTEM I SUBUNIT G) 0.652848473 -3.766152051 -0.245305361 -0.394136582 0.111313961 -0.235964596 -0.871718888 -0.321453677

At1g09480 cinnamyl-alcohol dehydrogenase family / CAD family 0.052093117 -0.393264135 -0.879888891 0.107212008 0.254572467 0.348761215 -0.391306624 -0.052356831

At1g09510 cinnamyl-alcohol dehydrogenase family / CAD family 0.016215258 0.083336062 -0.00712732 -0.014204151 -0.236937246 0.076336304 0.467789021 -1.360803497

At1g55810 "uracil phosphoribosyltransferase, putative / UMP pyrophosphorylase, putative / UPRTase, putative" 0.621098504 0.201497388 0.494757778 -1.778407405 -0.026972933 -0.72635411 -0.312576679 0.065818527

At1g05310 pectinesterase family protein -1.286097687 0.208789383 0.643528327 -0.89591771 0.482688944 -0.649152818 0.37934294 0.108970826

At1g05160 ATKAO1_KAO1__CYP88A3 (CYTOCHROME P450 88 A3); ent-kaurenoate oxidase/ oxygen binding 0.62917083 -0.149806703 -0.031116865 -0.337533994 0.226077225 0.599867515 -0.320398306 -1.807193416

At2g17640 SAT-106__ATSERAT3;1; acetyltransferase/ serine O-acetyltransferase 0.051265474 -0.354997164 -1.023633235 -1.357231218 0.296592482 0.370732034 -0.306045763 -0.555751249

At1g04710 KAT1__PKT4 (PEROXISOMAL 3-KETOACYL-COA THIOLASE 4); acetyl-CoA C-acyltransferase/ catalytic -0.163641394 -0.114987178 0.317532531 0.846734583 -0.078633242 0.738951926 0.706088857 -0.318793635

At1g04580 ATAO-4_ATAO2__AAO4 (ARABIDOPSIS ALDEHYDE OXIDASE 4); aldehyde oxidase/ aryl-aldehyde oxidase 0.182377257 -0.012616121 -0.304009342 -0.283900586 -0.370725136 0.475346473 0.828141703 -1.787068604

At1g65520 "ATECI1_ECHIC_PEC11__ECI1 (DELTA(3), DELTA(2)-ENOYL COA ISOMERASE 1); carnitine racemase/ catalytic/ dodecenoyl-CoA delta-isomerase" 0.02013264 0.591256027 -0.512045379 0.074529109 0.197862281 -0.416789333 -0.080334074 -0.140929384

At1g08980 "ATAMI1_ATTOC64-I__AMI1 (AMIDASE 1); amidase/ hydrolase, acting on carbon-nitrogen (but not peptide) bonds / indoleacetamide hydrolase" 0.218659411 -1.149037143 0.167488073 -0.321299891 2.975122747 -0.375179325 -0.700036211 -0.180036958

At1g09940 HEMA2; glutamyl-tRNA reductase -0.135355329 0.724056876 0.630524357 1.97122652 -0.324645478 -2.95673632 0.863796466 -0.200079361

At1g09780 "2,3-biphosphoglycerate-independent phosphoglycerate mutase, putative / phosphoglyceromutase, putative" -0.327623853 0.855285697 0.543336978 0.565024196 -0.177651649 0.812917447 -0.370028464 2.149121534

At1g09795 HISN1B__ATATP-PRT2 (ATP PHOSPHORIBOSYL TRANSFERASE 2); ATP phosphoribosyltransferase 0.048619497 -1.091596349 0.222917132 1.844578527 1.992573842 0.771695699 -0.041875453 -1.441313131

At1g09830 phosphoribosylamine--glycine ligase (PUR2) 0.069764213 0.023674338 0.659282185 0.703051502 -0.244282519 0.968299813 -0.552360867 0.691973877

At1g65570 "polygalacturonase, putative / pectinase, putative" -0.470474393 0.746655043 0.328168816 -0.515402008 0.200632857 -0.261330744 0.283120476 -0.035240661

At1g65610 "ATGH9A2__KOR2; catalytic/ hydrolase, hydrolyzing O-glycosyl compounds" -0.415075409 1.159116697 -0.377645932 -0.13962205 -0.289794674 -0.708086636 -0.302337141 0.460772585

At1g09620 ATP binding / aminoacyl-tRNA ligase/ leucine-tRNA ligase/ nucleotide binding 0.678535006 0.108950306 0.405250709 -0.044109938 0.126460851 0.364339066 -1.186172924 -0.296102534

At1g62180 APSR_ATAPR2_PRH_PRH43__APR2 (5'ADENYLYLPHOSPHOSULFATE REDUCTASE 2); adenylyl-sulfate reductase/ phosphoadenylyl-sulfate reductase (thioredoxin) -1.722762509 -0.61903761 0.032070868 1.039627782 -2.66023746 0.342074465 -1.130563496 -0.773099436

At1g23020 ATFRO3__FRO3; ferric-chelate reductase -2.562530811 0.46184542 -0.739406642 -0.761223634 1.610381944 -0.345636786 0.249609902 1.409325295

At1g22940 TH-1_THIE__TH1 (THIAMINE REQUIRING 1); hydroxymethylpyrimidine kinase/ phosphomethylpyrimidine kinase/ thiamin-phosphate diphosphorylase -0.200128445 -0.149714787 0.097214924 0.089931246 0.50454493 0.033585325 -0.260025589 -0.366010153

At1g22880 "ATCEL5_ATGH9B4__CEL5 (CELLULASE 5); catalytic/ hydrolase, hydrolyzing O-glycosyl compounds" -0.931053069 1.081890752 0.940099027 -0.704700791 0.178144708 -0.135880489 0.451440667 -0.305536058

At1g08630 THA1 (Threonine Aldolase 1); aldehyde-lyase/ threonine aldolase 2.671252572 -0.803708091 -1.513649008 -1.060006443 -0.351421818 -0.783742489 -0.664006456 -1.570823155

At1g08550 AVDE1__NPQ1 (NON-PHOTOCHEMICAL QUENCHING 1); violaxanthin de-epoxidase -0.993309731 -2.06120316 0.161211788 0.242761532 3.534785571 -0.098710761 0.053316443 -0.670901408

At1g08830 CSD1 (COPPER/ZINC SUPEROXIDE DISMUTASE 1); superoxide dismutase 1.088458533 0.048871371 -0.132385437 0.412160436 -0.24768474 0.070054057 -0.266552146 1.22989001

At1g03475 ATCPO-I_HEMF1__LIN2 (LESION INITIATION 2); coproporphyrinogen oxidase -0.44777194 -0.875089975 0.879463388 0.781350343 1.396816156 0.182639102 -0.413573372 0.152188616

At1g03630 PORC__POR C (PROTOCHLOROPHYLLIDE OXIDOREDUCTASE); NADPH dehydrogenase/ oxidoreductase/ protochlorophyllide reductase -0.387746052 -3.158051619 1.106939934 0.410890771 -0.040716699 0.435022286 0.021745383 0.581786935

At2g17265 HSK (HOMOSERINE KINASE); homoserine kinase -0.209983535 0.267272471 -0.131362429 -0.379203985 0.703367383 1.077140571 -0.275373711 0.073763695

At1g24280 G6PD3 (GLUCOSE-6-PHOSPHATE DEHYDROGENASE 3); glucose-6-phosphate dehydrogenase -1.460483961 1.759045629 0.804993911 0.702396075 0.308927871 1.851218394 -0.542551328 -0.168593303

At1g24180 "IAR4; oxidoreductase, acting on the aldehyde or oxo group of donors, disulfide as acceptor / pyruvate dehydrogenase (acetyl-transferring)" -0.085702226 1.324983734 -0.074143081 0.289761985 -0.334520097 -0.068736506 0.576267574 1.148348693

At1g24100 "UGT74B1 (UDP-glucosyl transferase 74B1); UDP-glycosyltransferase/ thiohydroximate beta-D-glucosyltransferase/ transferase, transferring glycosyl groups" -0.533117357 0.329529783 -0.231051418 0.304810221 -0.149719848 0.01104616 -1.456951549 1.469709115

At1g23200 pectinesterase family protein -0.422527707 -0.289294465 -1.292380073 -0.638064371 -1.035617869 0.440128232 0.975315022 -3.280173296

At1g23210 "AtGH9B6 (Arabidopsis thaliana glycosyl hydrolase 9B6); catalytic/ hydrolase, hydrolyzing O-glycosyl compounds" -0.356621837 0.917199928 0.685776832 0.48933719 0.304702482 -0.308987075 1.532444864 -0.286612618

At1g23190 "phosphoglucomutase, cytoplasmic, putative / glucose phosphomutase, putative" -0.428471005 0.285600208 -0.670020901 -0.023330253 -0.332243413 1.260603571 0.762887449 0.907958195

At2g17420 ATNTRA_NTR2__NTRA (NADPH-DEPENDENT THIOREDOXIN REDUCTASE A); thioredoxin-disulfide reductase -0.035605276 0.979020078 -0.135512422 0.362365702 -0.024554084 0.187249925 -0.200481131 1.518145524

At1g60810 ACLA-2; ATP citrate synthase 1.31369365 0.576992698 1.141184456 -1.536368216 -0.276478449 0.92272933 -0.15283548 0.713338552

At1g60550 DHNS__ECHID (ENOYL-COA HYDRATASE/ISOMERASE D); catalytic/ naphthoate synthase -0.392952091 -1.843078516 1.222269785 -0.693362992 -0.426240591 -0.057788763 -0.317129438 -0.257893531

At1g60470 "AtGolS4 (Arabidopsis thaliana galactinol synthase 4); transferase, transferring glycosyl groups / transferase, transferring hexosyl groups" -0.140810891 0.796574827 -0.274212557 0.680716959 -0.214938637 -0.786636239 -0.223871871 -0.444820694

At1g77120 ATADH__ADH1 (ALCOHOL DEHYDROGENASE 1); alcohol dehydrogenase 1.720981993 0.666296619 -1.904990695 1.066823181 1.228939771 1.079092511 -1.907596155 -3.003190421

At1g60600 "ABC4 (ABERRANT CHLOROPLAST DEVELOPMENT 4); 1,4-dihydroxy-2-naphthoate octaprenyltransferase/ prenyltransferase" -0.137511479 -1.243236848 1.473803823 0.482901685 0.771455264 0.194091067 0.015209068 0.100229646

At1g67280 "lactoylglutathione lyase, putative / glyoxalase I, putative" -0.90227044 -0.926315721 -0.372338881 0.258292278 0.841884511 -0.090632525 -0.164784939 -0.256653917

At1g24360 "3-oxoacyl-(acyl-carrier protein) reductase, chloroplast / 3-ketoacyl-acyl carrier protein reductase" 0.920768265 0.224867731 0.728464792 -0.268330649 -0.443196046 0.866198839 -0.932623237 0.16724339

At1g55570 sks12 (SKU5 Similar 12); copper ion binding / oxidoreductase 0.573249491 0.328555366 -0.034055162 0.019650742 0.005398969 0.111576085 6.402843579 0.372229982

At1g03495 "transferase/ transferase, transferring acyl groups other than amino-acyl groups" -0.683689987 0.218420131 -0.7080848 3.228998219 -1.416021525 1.761234674 -1.011641355 -0.352598575

At1g31070 UDP-N-acetylglucosamine pyrophosphorylase-related -0.373085065 1.009260995 0.956718214 -0.23554915 0.200238346 0.247278617 0.513583954 0.361332972

At1g62570 FMO GS-OX4 (FLAVIN-MONOOXYGENASE GLUCOSINOLATE S-OXYGENASE 4); 4-methylthiopropyl glucosinolate S-oxygenase/ 8-methylthiopropyl glucosinolate S-oxygenase/ flavin-containing monooxygenase/ monooxygenase -0.296823438 1.064936901 -3.183552291 -0.010051751 -0.876263319 1.015597154 -0.409968334 0.778705284

At1g62640 "KAS III (3-KETOACYL-ACYL CARRIER PROTEIN SYNTHASE III); 3-oxoacyl-[acyl-carrier-protein] synthase/ catalytic/ transferase, transferring acyl groups other than amino-acyl groups" 0.322426479 0.258113996 1.172744886 -0.705363583 0.041247392 0.532211898 -0.218873712 -0.299008405

At1g62560 FMO GS-OX3 (FLAVIN-MONOOXYGENASE GLUCOSINOLATE S-OXYGENASE 3); 3-methylthiopropyl glucosinolate S-oxygenase/ 4-methylthiopropyl glucosinolate S-oxygenase/ 5-methylthiopropyl glucosinolate S-oxygenase/ 6-methylthiopropyl glucosinolate S-oxygenase/ 7-methyl -3.112079235 -0.760173926 -0.001078949 0.589567765 -0.173802826 2.075693742 -0.410487719 0.091965833

At1g62540 FMO GS-OX2 (FLAVIN-MONOOXYGENASE GLUCOSINOLATE S-OXYGENASE 2); 3-methylthiopropyl glucosinolate S-oxygenase/ 4-methylthiopropyl glucosinolate S-oxygenase/ 5-methylthiopropyl glucosinolate S-oxygenase/ 7-methylthiopropyl glucosinolate S-oxygenase/ 8-methyl 0.939194585 -0.167133416 -0.827013627 0.958596896 -0.335611273 1.153686094 -0.343247151 0.781033304

At1g55560 sks14 (SKU5 Similar 14); copper ion binding / oxidoreductase 0.622007571 0.214648801 -0.313612075 -0.278141031 -0.177188555 0.276759127 6.04777441 0.264953192

At1g51260 LPAT3; 1-acylglycerol-3-phosphate O-acyltransferase -0.353010662 0.987759119 0.753816509 0.522560425 0.278197295 -0.501001835 2.314677608 -0.321855362

At1g23730 ATBCA3__BCA3 (BETA CARBONIC ANHYDRASE 4); carbonate dehydratase/ zinc ion binding -0.768110044 0.175136744 -1.030620573 0.398281022 -0.001420211 -0.026565919 2.417945954 0.890043619

At1g23820 SPDS1 (spermidine synthase 1); spermidine synthase 0.277967553 0.42718013 0.11414624 0.188653516 1.161516476 1.013513267 -0.31118415 0.168956881

At1g23800 ALDH2B7; 3-chloroallyl aldehyde dehydrogenase/ aldehyde dehydrogenase (NAD) -0.943029975 1.89185588 -0.247059534 0.465019507 0.441191123 -0.198449675 1.249370774 0.040420355

At1g05010 ACO4_EAT1__EFE (ETHYLENE-FORMING ENZYME); 1-aminocyclopropane-1-carboxylate oxidase -2.160292167 -0.938948268 -1.341350955 1.0721067 0.326865521 -1.79541207 -1.113445267 2.299689683

At2g36750 "UGT73C1 (UDP-GLUCOSYL TRANSFERASE 73C1); UDP-glucosyltransferase/ UDP-glycosyltransferase/ cis-zeatin O-beta-D-glucosyltransferase/ trans-zeatin O-beta-D-glucosyltransferase/ transferase, transferring glycosyl groups" 0.363736477 -0.108250662 -1.055863982 0.864509224 -0.068043495 -0.112678336 0.045054191 0.035290721

At2g36760 "UGT73C2 (UDP-glucosyl transferase 73C2); UDP-glycosyltransferase/ transferase, transferring glycosyl groups / transferase, transferring hexosyl groups" -0.04480224 0.030013895 0.108362723 -0.096568014 -0.014178349 0.03643226 0.029566895 -0.975399697

At2g36770 UDP-glucoronosyl/UDP-glucosyl transferase family protein 0.37367025 0.026355289 -1.912983942 1.325170388 0.081208034 -0.94093601 0.007282272 -1.346086005

At2g36790 "UGT73C6 (UDP-glucosyl transferase 73C6); UDP-glucosyltransferase/ UDP-glycosyltransferase/ quercetin 3-O-glucosyltransferase/ quercetin 4'-O-glucosyltransferase/ quercetin 7-O-glucosyltransferase/ transferase, transferring glycosyl groups" 0.14822881 -0.821533788 -2.470001292 1.399872199 -0.79542586 -0.507773946 0.579361448 0.9716356

At2g36690 "oxidoreductase, 2OG-Fe(II) oxygenase family protein" -0.2620451 1.010448073 0.032029234 -0.868111126 -0.502568051 -1.848248357 -1.060651025 0.368428864

At2g02050 "NADH-ubiquinone oxidoreductase B18 subunit, putative" 0.274837035 0.465192017 0.340207022 -0.289515356 0.232284437 0.351763904 -0.067047611 0.135271346

At2g36700 pectinesterase family protein 0.098918757 0.20943961 0.216625354 0.161456135 0.237391688 -0.265268948 0.151965398 -0.510683209

At2g36710 pectinesterase family protein -0.184347624 0.08480839 0.084003654 -0.209465052 -0.166143125 0.181725611 0.153545604 -0.713959831

At2g43020 ATPAO2 (Polyamine oxidase 2); amine oxidase/ electron carrier/ oxidoreductase 0.543620479 0.145763459 -0.658927642 0.467324331 0.807732009 0.02089626 0.836097701 -0.758388563

At2g43050 ATPMEPCRD; enzyme inhibitor/ pectinesterase -1.19142349 0.320635834 -0.584372026 -3.27203852 -0.380815228 0.370033327 0.367950318 -1.3217393

At2g20420 "succinyl-CoA ligase (GDP-forming) beta-chain, mitochondrial, putative / succinyl-CoA synthetase, beta chain, putative / SCS-beta, putative" 0.348269274 0.56985647 -0.144683605 0.468047251 0.086019917 0.881742952 -0.304277774 1.120762301

At2g42910 ribose-phosphate pyrophosphokinase 4 / phosphoribosyl diphosphate synthetase 4 (PRS4) 0.436077535 0.408366732 0.546473943 -0.045997549 -0.325422433 0.691952899 -0.604512236 0.762688035

At2g20370 "KAM1__MUR3 (MURUS 3); catalytic/ transferase, transferring glycosyl groups" -0.236239269 0.558540308 0.353708394 0.725809053 -0.432070402 0.081350153 -0.66560059 -0.683552908

At2g20360 binding / catalytic/ coenzyme binding 0.399826782 0.365856332 0.366127175 -0.191764196 0.118625749 0.419347522 -0.038741828 0.681763242

At2g20260 PSAE-2 (photosystem I subunit E-2); catalytic 0.187196792 -3.354530412 0.137280495 -0.622318464 1.091636976 -0.722151944 -0.574917226 -0.212751643

At2g22590 "transferase, transferring glycosyl groups" -0.661573976 1.285615483 -0.212357231 0.119628156 -0.578846434 0.818247721 -0.128595999 -0.057324462

At2g20340 "tyrosine decarboxylase, putative" -0.342186072 -0.436124804 -0.08478162 1.52469645 -0.550153872 0.372274536 0.07918961 0.672795484

At2g18250 ATCOAD (4-phosphopantetheine adenylyltransferase); nucleotidyltransferase/ pantetheine-phosphate adenylyltransferase -0.195606485 0.044028442 -0.496836434 -0.300335244 0.704244315 -0.474387715 -0.619316765 -0.315651175

At2g18450 SDH1-2; succinate dehydrogenase -0.324457142 0.762451809 0.515411148 -0.175572234 -0.142653815 0.022488735 -0.011895458 -0.013126517

At2g18230 AtPPa2 (Arabidopsis thaliana pyrophosphorylase 2); inorganic diphosphatase/ pyrophosphatase 0.201630424 -0.23281534 -0.621997188 0.911049898 -1.695984121 2.969877464 0.66020342 0.845321112

At2g22570 ATNIC1__NIC1 (NICOTINAMIDASE 1); catalytic/ nicotinamidase -0.331008232 0.604070278 -0.437500443 -0.507203741 1.426775741 0.226038226 0.125670507 0.036953789

At2g06520 PSBX (photosystem II subunit X) 0.449162446 -3.046266135 -0.059077581 -0.725827041 -0.31081974 0.238367401 -1.000852984 0.501936005

At2g20690 lumazine-binding family protein -0.439669058 -0.468992331 0.327583151 0.729151907 0.693986948 0.429440293 -0.35705562 0.733949174

At2g20810 "LGT4__GAUT10 (GALACTURONOSYLTRANSFERASE 10); polygalacturonate 4-alpha-galacturonosyltransferase/ transferase, transferring glycosyl groups / transferase, transferring hexosyl groups" 0.277815485 0.272227862 0.507896726 -0.026273427 -0.192909785 0.068666417 -0.192243049 -0.377738702

At2g20860 LIP1 (LIPOIC ACID SYNTHASE 1); lipoic acid synthase -0.616874186 -0.322464048 0.25542747 0.1955523 0.309465448 -0.211918857 0.680607321 -0.542158647

At2g46505 SDH4; succinate dehydrogenase (ubiquinone) -0.071357195 0.593620419 0.192068244 -0.076985073 0.25275113 0.119782092 -0.063151201 -0.119825282

At2g37090 "IRX9 (IRREGULAR XYLEM 9); transferase, transferring glycosyl groups / xylosyltransferase" -0.844203364 0.496595924 -0.133334062 -2.355042092 -0.695808812 0.669732897 0.568592233 -0.17483488

At2g15620 ATHNIR__NIR1 (NITRITE REDUCTASE 1); ferredoxin-nitrate reductase/ nitrite reductase (NO-forming) -3.102544029 0.106755257 0.720503181 0.333118432 -0.504198287 0.339371385 -0.550390865 0.649662945

At2g46480 "LGT2__GAUT2; polygalacturonate 4-alpha-galacturonosyltransferase/ transferase, transferring glycosyl groups" 0.101246517 0.449834237 0.600062044 0.447016108 0.185177885 -0.352251551 0.973507975 -0.385520432

At2g15480 "UGT73B5 (UDP-glucosyl transferase 73B5); UDP-glucosyltransferase/ UDP-glycosyltransferase/ quercetin 3-O-glucosyltransferase/ transferase, transferring glycosyl groups" 0.027614186 1.35789769 -2.30216666 1.75550395 -0.835583121 -1.795947043 -1.028319541 1.370807612

At2g15490 "UGT73B4 (UDP-GLYCOSYLTRANSFERASE 73B4); UDP-glucosyltransferase/ UDP-glycosyltransferase/ quercetin 3-O-glucosyltransferase/ quercetin 7-O-glucosyltransferase/ transferase, transferring glycosyl groups" 0.190267197 1.562346962 -1.264118667 0.981037997 -0.751098545 -1.152573966 -1.237120424 1.067018777

At2g06050 OPR3 (OPDA-REDUCTASE 3); 12-oxophytodienoate reductase 0.690924997 -0.380436882 -0.304855526 2.743607607 -0.136908831 -2.05457043 -0.799693518 2.66103934

At2g28210 -0.061528976 0.552400253 0.141058378 2.711445983 -0.030525857 -1.628630697 -0.708881999 -0.375139581

At2g25540 "CESA10 (CELLULOSE SYNTHASE 10); cellulose synthase/ transferase, transferring glycosyl groups" -0.202015465 0.054230866 0.067083497 -0.665091275 -0.590708616 0.561003387 0.39004925 -2.754589377

At2g25450 "2-oxoglutarate-dependent dioxygenase, putative" -4.550479592 -0.330711833 -2.533574338 -1.897495193 0.434361139 -0.096723135 0.07037652 -0.597072638

At2g27490 ATCOAE; ATP binding / dephospho-CoA kinase 0.495105888 0.335232401 -0.484499143 0.152150471 -0.242789032 -0.367235179 -0.029046746 -0.579460176

At2g27450 "ATNLP1_CPA__NLP1 (NITRILASE-LIKE PROTEIN 1); N-carbamoylputrescine amidase/ hydrolase, acting on carbon-nitrogen (but not peptide) bonds" -0.256423849 -0.018092581 -0.322203669 -0.464441899 -0.038623704 0.22946399 -0.309109018 0.941690101

At2g31955 CNX2 (COFACTOR OF NITRATE REDUCTASE AND XANTHINE DEHYDROGENASE 2); catalytic 0.061717399 0.19182188 -0.041807232 0.428112191 0.323218186 -0.239054027 0.090599985 -0.238963989

At2g01140 "fructose-bisphosphate aldolase, putative" 0.625002143 0.833431263 -0.126785707 -0.488047323 -0.594471497 0.950588024 -0.683597209 0.410620824

At2g01180 ATLPP1_LPP1_PAP1__ATPAP1 (PHOSPHATIDIC ACID PHOSPHATASE 1); phosphatidate phosphatase -0.833222002 0.25678521 0.244965011 0.682837396 -0.848591767 -2.449528886 -0.198602527 0.638767449

At2g01350 QPT (QUINOLINATE PHOSHORIBOSYLTRANSFERASE); nicotinate-nucleotide diphosphorylase (carboxylating) -0.115646765 -0.092827622 -0.917583442 -0.066267528 0.372757744 0.624000506 -0.603136668 0.441836036

At2g01290 ribose-5-phosphate isomerase -0.766678583 -0.607482309 0.322563007 1.362580003 -0.125226011 1.846113869 0.418499663 -0.343271779

At2g35690 "ACX5 (ACYL-COA OXIDASE 5); FAD binding / acyl-CoA dehydrogenase/ acyl-CoA oxidase/ electron carrier/ oxidoreductase/ oxidoreductase, acting on the CH-CH group of donors" -0.291340539 -0.151818021 -0.359435408 -0.588259033 0.40383634 -0.152092535 1.101110335 -0.303205407

At2g42310 unknown protein 0.551319882 0.616310407 0.040394159 -0.227163033 1.230404919 0.389431811 -0.394616707 0.166857977

At2g15090 "KCS8 (3-KETOACYL-COA SYNTHASE 8); acyltransferase/ catalytic/ transferase, transferring acyl groups other than amino-acyl groups" 1.314670157 -1.514687831 2.376004593 0.178011712 2.035536254 -1.206502311 -1.323026133 -1.508214016

At2g18620 "geranylgeranyl pyrophosphate synthase, putative / GGPP synthetase, putative / farnesyltranstransferase, putative" -0.053705221 0.449279137 0.300320644 0.050318054 0.170922767 -0.137798708 -0.04902378 -0.249791575

At2g18570 UDP-glucoronosyl/UDP-glucosyl transferase family protein -1.696804826 0.157800967 -0.106244367 -2.180905052 0.239689703 -0.138513843 0.248539298 -1.416933879

At2g19570 AT-CDA1_DESZ__CDA1 (CYTIDINE DEAMINASE 1); cytidine deaminase 0.117812877 0.487184732 -2.455790907 -0.975208824 -0.664753028 0.223993967 0.246548618 -1.257944973

At2g19500 ATCKX2__CKX2 (CYTOKININ OXIDASE 2); amine oxidase/ cytokinin dehydrogenase -0.10261665 0.240709029 0.109783974 0.22778217 0.040610037 -0.43171384 1.589369349 -0.715932517

At2g19590 ACO1 (ACC OXIDASE 1); 1-aminocyclopropane-1-carboxylate oxidase -0.847109926 1.249408991 0.854478134 -0.768648941 -0.066638932 -0.009814109 -0.433812555 -0.489046906

At2g37260 ATWRKY44_DSL1_WRKY44__TTG2 (TRANSPARENT TESTA GLABRA 2); transcription factor 0.194716302 0.173423307 0.282900459 0.161812215 -0.218010121 0.285268322 -0.565026831 -1.361412397

At2g37250 ATPADK1__ADK (ADENOSINE KINASE); adenylate kinase/ nucleotide kinase -0.437316543 0.784615423 0.085101791 0.923874609 -0.726922212 0.451397659 -0.095557885 0.462102969

At2g24210 TPS10 (terpene synthase 10); (E)-beta-ocimene synthase/ myrcene synthase 1.545387656 -0.175126916 -1.158308828 -0.104262579 0.306678733 -0.387033397 2.823059933 0.76883762

At2g24270 ALDH11A3; 3-chloroallyl aldehyde dehydrogenase/ glyceraldehyde-3-phosphate dehydrogenase (NADP+) 1.497558719 -3.454783945 0.097281764 -1.05380681 -1.021235277 0.197149711 -0.50768954 -0.096227062

At2g05990 ENR1__MOD1 (MOSAIC DEATH 1); enoyl-[acyl-carrier-protein] reductase (NADH)/ enoyl-[acyl-carrier-protein] reductase/ oxidoreductase 0.947739789 0.110885962 2.332428486 -1.156729801 -0.456297012 0.340407146 -0.21039283 -0.357723525

At2g07727 cytochrome b (MTCYB) (COB) (CYTB) 1.113159405 0.429634867 -0.477097286 -0.041563918 0.743049028 -0.582606152 -0.277038945 -0.877016312

At2g18640 GGPS4 (GERANYLGERANYL PYROPHOSPHATE SYNTHASE 4); farnesyltranstransferase -0.010292146 0.28203201 0.247121635 -0.008430422 0.353601471 -0.276368274 -0.08334067 -0.543461679

At2g18700 "ATTPSB_TPS11__ATTPS11; transferase, transferring glycosyl groups" 0.965803708 -1.442273045 -0.384242425 -2.458202702 -0.488511545 -5.744091873 -0.48263349 -0.523858794

At2g38050 DWF6__DET2 (DE-ETIOLATED 2); sterol 5-alpha reductase 0.371461638 0.36706125 0.094277028 -0.703938584 0.649627919 -0.447836451 -0.549873781 0.219907504

At2g38040 CAC3; acetyl-CoA carboxylase 0.210608865 -0.385241958 0.704574928 -1.038252336 0.029457968 -0.101813775 -0.404958011 -0.212179242

At2g45150 phosphatidate cytidylyltransferase family protein 0.089606011 -0.084926433 0.561566298 0.641836734 0.519814616 0.518772893 -0.448494603 -1.242951924

At2g44990 MAX3__CCD7 (CAROTENOID CLEAVAGE DIOXYGENASE 7); 9-cis-epoxycarotenoid dioxygenase -0.209682085 0.299842105 0.145749371 0.478931771 0.476867953 -0.57346605 0.020407033 -1.641268518

At2g28190 CZSOD2__CSD2 (COPPER/ZINC SUPEROXIDE DISMUTASE 2); superoxide dismutase 0.159021568 -0.697228039 1.315667797 0.660938819 -0.565500357 0.488443607 -0.302428963 0.494933336

At2g28080 glycosyltransferase family protein 0.188039492 0.720038013 1.055648506 1.063265233 0.259636461 0.113895321 0.495555468 0.736911502

At2g27860 AXS1 (UDP-D-apiose/UDP-D-xylose synthase 1); NAD or NADH binding / UDP-glucuronate decarboxylase 0.020209046 0.129680613 0.667729382 -0.129970803 0.303963946 -0.657293994 0.302552866 1.15351536

At2g27730 unknown protein 0.391296615 0.73559415 -0.14047903 0.287472295 0.055449325 0.675806284 -0.076782688 0.090475647

At2g27760 IPPT__ATIPT2 (TRNA ISOPENTENYLTRANSFERASE 2); adenylate dimethylallyltransferase/ tRNA isopentenyltransferase 0.787080068 0.329408599 0.378571451 0.656731015 0.19715818 -0.149836816 -0.373671617 -0.305946676

At2g28880 emb1997 (embryo defective 1997); anthranilate synthase/ catalytic/ oxo-acid-lyase -0.245048135 0.203447899 0.6566634 0.805008404 -0.627946557 -0.023737413 0.255230841 -0.119295595

At2g28760 UXS6__NAD-dependent epimerase/dehydratase family protein -1.094334833 0.459300868 -0.145965805 -0.965293006 -0.549618816 0.252666889 0.431187827 0.802719438

At2g27690 CYP94C1; fatty acid (omega-1)-hydroxylase/ oxygen binding -0.871811189 -0.023511742 0.945829948 1.632157962 -1.799239336 -4.195489031 -0.197013779 -1.563102161

At2g27820 ADT3__PD1 (PREPHENATE DEHYDRATASE 1); arogenate dehydratase/ prephenate dehydratase 0.249999442 0.074354726 0.494281303 -0.26220855 -0.571248249 0.575037334 -0.441330794 -0.006840157

At2g29560 "enolase, putative" 0.254211311 0.512420484 0.577017351 -0.014117734 0.137713882 0.381586465 -0.24386251 0.417889457

At2g46720 "HIC__KCS13 (3-KETOACYL-COA SYNTHASE 13); acyltransferase/ catalytic/ transferase, transferring acyl groups other than amino-acyl groups" 2.30423002 -1.292048982 0.433948601 0.234079594 -0.249533328 -0.124943824 0.456713279 -0.109718101

At2g32440 CYP88A4__KAO2 (ENT-KAURENOIC ACID HYDROXYLASE 2); ent-kaurenoate oxidase/ oxygen binding 0.631114273 -0.532997865 0.893239939 0.057256291 0.639796799 0.196703975 0.531582352 -0.493369149

At2g32290 BMY5__BAM6 (BETA-AMYLASE 6); beta-amylase/ catalytic/ cation binding -2.229642534 -0.401574154 -0.535341989 1.256325625 0.644123716 1.868449419 0.703806465 -0.210898373

At2g32260 "cholinephosphate cytidylyltransferase, putative / phosphorylcholine transferase, putative / CTP:phosphocholine cytidylyltransferase, putative" 0.014545224 0.445520211 -0.60371474 -0.390130723 0.674785162 -0.006425858 -0.036641974 0.752503117

At2g41220 GLU2 (GLUTAMATE SYNTHASE 2); glutamate synthase (ferredoxin) 0.748052938 0.851941223 -1.151874285 -0.650750645 0.167370115 1.337052336 -0.673256604 0.933030018

At2g38670 PECT1 (PHOSPHORYLETHANOLAMINE CYTIDYLYLTRANSFERASE 1); ethanolamine-phosphate cytidylyltransferase 0.765420573 0.46021505 0.085652835 0.545992309 2.237432644 0.956016566 -0.028382506 -0.198823571

At2g38660 "tetrahydrofolate dehydrogenase/cyclohydrolase, putative" 0.776923295 0.222504959 -0.20130442 -0.119703149 0.594092066 0.649863467 -0.579445656 -0.044546799

At2g38740 haloacid dehalogenase-like hydrolase family protein -0.260391946 -0.047241581 -1.020656067 0.029453069 -1.948669536 0.5340908 0.951844776 1.198405211

At2g38700 ATMVD1__MVD1 (MEVALONATE DIPHOSPHATE DECARBOXYLASE 1); diphosphomevalonate decarboxylase/ protein homodimerization 0.538471565 0.648068308 0.460715281 -1.229923417 0.254333535 0.301560099 0.259347681 1.14907388

At2g38650 "LGT7__GAUT7 (GALACTURONOSYLTRANSFERASE 7); polygalacturonate 4-alpha-galacturonosyltransferase/ transferase, transferring glycosyl groups" 0.223917137 0.326929615 0.774736107 -0.065291306 0.333745529 0.533393497 0.168739432 0.550888472

At2g43180 catalytic -0.464712927 -0.288970177 -0.252648612 0.138774529 0.70439796 -0.216205549 0.090096235 -0.543580229

At2g22780 "PMDH1 (PEROXISOMAL NAD-MALATE DEHYDROGENASE 1); L-malate dehydrogenase/ binding / catalytic/ malate dehydrogenase/ oxidoreductase/ oxidoreductase, acting on the CH-OH group of donors, NAD or NADP as acceptor" -0.293962346 0.7087515 -0.482780364 0.487744086 0.138443119 0.370966528 0.13020677 -0.199059056

At2g31100 triacylglycerol lipase 0.170891784 0.293113307 0.309490734 0.267385634 0.19139348 -0.314123879 0.74939778 -0.504348268

At2g31170 SYCO ARATH; ATP binding / aminoacyl-tRNA ligase/ cysteine-tRNA ligase/ nucleotide binding -0.439533638 -0.421592164 1.517058058 1.062258143 0.308621518 0.303142179 -0.22954059 -0.389918168

At2g47630 esterase/lipase/thioesterase family protein 1.528164494 0.307293192 1.087111631 -0.606890157 -1.148872764 0.734772991 -1.62098732 -0.582744582

At2g07050 CAS1 (cycloartenol synthase 1); cycloartenol synthase 0.057571464 -0.050159982 0.776457547 -0.958813502 -0.24306875 -0.114158525 -0.870802829 -0.015588246

At2g06960 "oxidoreductase, 2OG-Fe(II) oxygenase family protein" -0.057286432 0.269123828 0.397182634 0.337307331 0.52109011 -0.354144119 0.290906836 -0.458200809

At2g06925 ATSPLA2-ALPHA__PLA2-ALPHA; phospholipase A2 1.401771643 -0.341139527 1.378726758 -0.872950665 3.711993171 0.048501717 -1.149881338 0.307400236

At2g47690 NADH-ubiquinone oxidoreductase-related 0.132664093 0.508307743 0.491456458 -0.350692926 0.357499579 0.133028556 -0.016939656 0.479709794

At2g35120 "glycine cleavage system H protein, mitochondrial, putative" 0.827331426 1.462073485 0.868541705 -1.304883814 -0.18461363 0.713889904 -0.164500744 -0.209664211

At2g16890 UDP-glucoronosyl/UDP-glucosyl transferase family protein 0.387446086 0.431822363 -1.705769094 -0.123395227 -0.662107934 1.244690697 1.298002494 -0.692324831

At2g23910 cinnamoyl-CoA reductase-related 0.273819438 1.153288199 -1.32249973 1.988649942 0.446636756 4.392857098 -0.416588184 0.090959177

At2g46110 PANB1__KPHMT1 (KETOPANTOATE HYDROXYMETHYLTRANSFERASE 1); 3-methyl-2-oxobutanoate hydroxymethyltransferase 0.172761205 -0.071363075 0.999197801 0.474019165 1.185397925 0.848013633 -0.528805166 -0.896527516

At2g35390 ribose-phosphate pyrophosphokinase 1 / phosphoribosyl diphosphate synthetase 1 (PRSI) -0.188251318 0.18282981 0.45649438 1.591151926 -0.466032606 -0.274647268 -0.0303158 -0.991143937

At2g35370 GDCH; glycine dehydrogenase (decarboxylating) -0.453239258 -3.316311969 0.265882208 0.889548879 0.225949874 0.657200906 -0.024242441 1.39664691

At2g29730 "UGT71D1 (UDP-GLUCOSYL TRANSFERASE 71D1); UDP-glycosyltransferase/ quercetin 3-O-glucosyltransferase/ transferase, transferring glycosyl groups" 0.297217492 0.613761495 0.680354284 -1.312310743 0.023644595 -0.318735996 0.315779092 1.895427735

At2g25840 OVA4 (ovule abortion 4); ATP binding / aminoacyl-tRNA ligase/ nucleotide binding / tryptophan-tRNA ligase -0.215780916 -0.652205076 1.662536501 0.535027402 0.149589169 -0.105546843 -0.171489847 -0.17785839

At2g29750 "UGT71C1 (UDP-GLUCOSYL TRANSFERASE 71C1); UDP-glycosyltransferase/ quercetin 3'-O-glucosyltransferase/ quercetin 7-O-glucosyltransferase/ transferase, transferring glycosyl groups" -1.009937097 2.258741671 0.324012527 -2.019306645 -0.280555714 0.120322261 -0.251134162 1.417718397

At2g29740 "UGT71C2 (UDP-GLUCOSYL TRANSFERASE 71C2); UDP-glycosyltransferase/ quercetin 3'-O-glucosyltransferase/ quercetin 3-O-glucosyltransferase/ quercetin 7-O-glucosyltransferase/ transferase, transferring glycosyl groups" -0.396807074 1.187102319 0.128853079 -0.77806623 -0.028507895 -0.919317515 -0.302819657 0.550910261

At2g29690 ATHANSYNAB__ASA2 (ANTHRANILATE SYNTHASE 2); anthranilate synthase -0.011442352 0.039312692 0.948565452 0.28258413 0.622269779 0.196697311 -0.271365309 -0.076878005

At2g29630 THIC (ThiaminC); ADP-ribose pyrophosphohydrolase/ catalytic/ iron-sulfur cluster binding -1.673388188 -1.910617312 -0.768968608 1.351974448 3.693384116 1.851628072 0.056196593 0.351288823

At2g25870 haloacid dehalogenase-like hydrolase family protein -0.566267984 0.285580485 1.138015097 1.083919351 0.136626235 0.049882077 0.700216429 0.574937454

At2g19800 MIOX2 (MYO-INOSITOL OXYGENASE 2); inositol oxygenase 2.472464605 0.856270529 1.469603173 -2.702160046 -0.807252449 -5.40374358 0.095692379 0.971615864

At2g19860 ATHXK2__HXK2 (HEXOKINASE 2); ATP binding / fructokinase/ glucokinase/ hexokinase 0.052073705 0.130100005 -0.326160968 0.791463094 0.975106107 1.228965162 -0.083958653 -0.800051583

At2g19940 "N-acetyl-gamma-glutamyl-phosphate reductase/ NAD or NADH binding / binding / catalytic/ oxidoreductase, acting on the aldehyde or oxo group of donors, NAD or NADP as acceptor / protein dimerization" -0.287041781 -0.304927687 1.232028246 0.148036513 0.073934697 -0.061914331 -0.30527291 0.176197641

At2g46820 PSAP_PTAC8_TMP14__PSI-P (PHOTOSYSTEM I P SUBUNIT); DNA binding -0.203473015 -3.20001271 0.083095578 -0.402743235 -0.016209444 -0.352154915 -0.665623405 0.156264419

At2g03220 "ATFT1_ATFUT1_MUR2__FT1 (FUCOSYLTRANSFERASE 1); fucosyltransferase/ transferase, transferring glycosyl groups" 1.022889044 0.425494257 0.151090155 -0.398772343 0.285218163 0.970075177 -0.658084671 0.599566891

At2g47040 VGD1 (VANGUARD1); enzyme inhibitor/ pectinesterase 1.386764373 -0.565394695 -1.318316443 -1.242616081 -0.641890249 0.891861458 7.01781963 -0.208024345

At2g46860 AtPPa3 (Arabidopsis thaliana pyrophosphorylase 3); inorganic diphosphatase/ pyrophosphatase -0.359721478 1.255667896 0.418075514 -0.53910377 -0.309241801 0.23031949 5.354520992 0.945455598

At2g22910 GCN5-related N-acetyltransferase (GNAT) family protein / amino acid kinase family protein 0.157330879 0.145322537 -0.160338002 1.320254277 0.043263021 -0.126279698 -0.710463561 -0.890067211

At2g22930 glycosyltransferase family protein -0.562529187 1.230544282 0.221336556 -0.488804116 0.035245883 -0.050681422 -0.091433981 0.411645279

At2g22810 ACC4__ACS4 (1-AMINOCYCLOPROPANE-1-CARBOXYLATE SYNTHASE 4); 1-aminocyclopropane-1-carboxylate synthase -0.570991812 -0.087170065 0.930227824 0.148677621 0.085575133 -1.088887943 0.29549615 -0.500533483

At2g22830 SQE2 (squalene epoxidase 2); FAD binding / oxidoreductase/ squalene monooxygenase 0.63115458 -0.118610085 0.772952769 -0.327301426 1.42032243 -0.613309198 0.445047519 0.064039105

At2g26870 phosphoesterase family protein 0.392929692 -0.253906376 0.771241693 0.530697692 0.642901413 0.094812739 0.247034308 -0.491287425

At2g26830 "emb1187 (embryo defective 1187); kinase/ phosphotransferase, alcohol group as acceptor" 0.13633573 0.762329466 -0.123813404 -0.045178683 0.489547171 0.04692885 0.373316538 0.191107571

At2g26800 "hydroxymethylglutaryl-CoA lyase, putative / 3-hydroxy-3-methylglutarate-CoA lyase, putative / HMG-CoA lyase, putative" -0.323937616 -0.243237707 -0.420752607 -0.875849218 -1.302352022 -0.528775069 0.853709225 -0.004723588

At2g29980 FAD3 (FATTY ACID DESATURASE 3); omega-3 fatty acid desaturase 0.21862724 0.507579022 1.6495885 -0.295268983 1.157864921 0.551937 0.108133234 -1.334175333

At2g44800 "oxidoreductase, 2OG-Fe(II) oxygenase family protein" 0.048021695 0.455771337 0.32231453 0.647777679 0.306105767 -0.184242182 0.216140361 -0.74801

At2g44570 "AtGH9B12 (Arabidopsis thaliana glycosyl hydrolase 9B12); catalytic/ hydrolase, hydrolyzing O-glycosyl compounds" -0.027188949 0.330171514 0.510976206 0.404886175 0.457836406 -0.531031783 0.586201565 -0.417363019

At2g44810 DAD1 (DEFECTIVE ANTHER DEHISCENCE 1); phospholipase A1/ triacylglycerol lipase -0.161160601 0.331822626 0.351930294 0.448237724 0.101121735 -0.677008015 -0.021803122 -1.079223677

At2g44750 TPK2 (Thiamin pyrophosphokinase 2); thiamin diphosphokinase 0.452236415 0.595003283 -0.849424059 0.876460627 3.91E-05 0.157900021 -0.498343142 -0.041962472

At2g26080 AtGLDP2 (Arabidopsis thaliana glycine decarboxylase P-protein 2); ATP binding / glycine dehydrogenase (decarboxylating) -0.659998577 -0.770742496 0.689647895 -0.336181321 -1.935250342 0.875507993 -0.105336899 1.019616384

At2g45880 BMY4__BAM7 (BETA-AMYLASE 7); beta-amylase/ catalytic/ cation binding 0.540327885 0.079078197 0.299172891 0.214722191 -0.019440609 -0.136586221 -0.122976106 -0.166433358

At2g45970 LCR__CYP86A8; fatty acid (omega-1)-hydroxylase/ oxygen binding 1.399674305 -0.462268729 1.561016875 -0.644560632 -0.536072079 -0.378371998 -0.25425152 -0.467405598

At2g45790 ATPMM__PMM (PHOSPHOMANNOMUTASE); phosphomannomutase -0.468775502 0.308361223 0.176903883 -0.202095909 0.925985441 1.161291716 -0.749549181 2.003727543

At2g18950 ATHPT_TPT1_VTE2__HPT1 (HOMOGENTISATE PHYTYLTRANSFERASE 1); homogentisate phytyltransferase/ prenyltransferase 1.158520704 -0.599081169 -1.707152217 1.001655864 1.306623672 1.579806094 -0.926767848 0.214024663

At2g34555 ATGA2OX3 (gibberellin 2-oxidase 3); gibberellin 2-beta-dioxygenase 0.173280476 0.214433184 0.410197989 0.418196328 0.016048645 -0.367738865 0.384131022 -0.412858389

At2g34630 "GPPS_GPS1__geranyl diphosphate synthase, putative / GPPS, putative / dimethylallyltransferase, putative / prenyl transferase, putative" 0.446633613 0.502781561 -0.404445341 0.990805575 -1.247135786 0.596505457 -0.522884601 -0.410509593

At2g39400 "hydrolase, alpha/beta fold family protein" -0.527818963 -1.389117405 -1.779003552 -0.517292386 0.700440071 -4.426929763 -1.464503518 -1.559136208

At2g39290 PGPS1_PGS1__PGP1 (PHOSPHATIDYLGLYCEROLPHOSPHATE SYNTHASE 1); CDP-alcohol phosphatidyltransferase/ CDP-diacylglycerol-glycerol-3-phosphate 3-phosphatidyltransferase 0.30856772 -0.179031273 0.739648496 0.12873685 0.312582836 0.248932914 -0.256877251 0.117670893

At2g32620 "ATCSLB2_CSLB02__ATCSLB02; cellulose synthase/ transferase/ transferase, transferring glycosyl groups" -0.549169793 0.863953435 0.43474077 -0.370802575 0.072430997 0.026988529 0.194178664 -0.0466901

At2g38280 ATAMPD__FAC1 (EMBRYONIC FACTOR1); AMP deaminase 0.496086107 0.374313965 0.075583644 0.294416129 -0.119110314 0.601178794 -0.92835621 0.756361935

At2g41490 GPT (UDP-GLCNAC%3ADOLICHOL+PHOSPHATE+GLCNAC-1-P+TRANSFERASE); UDP-N-acetylglucosamine-dolichyl-phosphate N-acetylglucosaminephosphotransferase -0.496748483 0.55789962 -0.280697419 0.100669487 0.76256653 0.066257573 0.265496249 0.360683163

At2g14750 "AKN1_ATAKN1__APK (APS KINASE); ATP binding / adenylylsulfate kinase/ kinase/ transferase, transferring phosphorus-containing groups" -1.691854859 0.68378419 0.155014781 0.657511304 -0.564152382 -0.520337439 0.828576778 0.906881344

At2g32540 "ATCSLB4_CSLB04__ATCSLB04; cellulose synthase/ transferase/ transferase, transferring glycosyl groups" -1.361503743 -1.825176625 -1.459288996 -0.494046187 -2.604424112 1.156471672 1.350514082 0.236443999

At2g32610 "ATCSLB1_CSLB01__ATCSLB01; cellulose synthase/ transferase/ transferase, transferring glycosyl groups" -0.39603058 0.979339962 0.203819249 -0.459614678 0.227790553 -0.221454667 0.037235987 0.25609437

At2g23560 "ATMES7__MES7 (METHYL ESTERASE 7); hydrolase/ hydrolase, acting on ester bonds / methyl indole-3-acetate esterase/ methyl salicylate esterase" -1.617940992 0.874793692 0.964355773 0.326959869 -0.067333258 -0.13005869 0.2742131 -0.093683755

At2g23610 "ATMES3__MES3 (METHYL ESTERASE 3); hydrolase, acting on ester bonds / methyl indole-3-acetate esterase/ methyl jasmonate esterase" -2.48058192 -0.258455768 0.355271885 -1.046568121 -0.362896188 -0.306637123 -1.816459788 -0.125356508

At2g23620 "ATMES1__MES1 (METHYL ESTERASE 1); hydrolase, acting on ester bonds / methyl indole-3-acetate esterase/ methyl jasmonate esterase/ methyl salicylate esterase" -0.867571435 1.067615334 -0.087930483 -1.2008894 -0.38517254 -0.452402118 -0.010708695 0.038733752

At2g23420 NAPRT2 (NICOTINATE PHOSPHORIBOSYLTRANSFERASE 2); nicotinate phosphoribosyltransferase -0.574502591 -0.444167294 -0.372585179 1.281406964 -1.840733924 -0.183017464 0.253642592 0.794991276

At2g38110 ATGPAT6__GPAT6 (GLYCEROL-3-PHOSPHATE ACYLTRANSFERASE 6); 1-acylglycerol-3-phosphate O-acyltransferase/ acyltransferase 2.605391459 -0.155138313 -0.870372622 -0.924862658 -0.279446851 0.442903865 2.243023742 1.344711661

At2g38240 "oxidoreductase, 2OG-Fe(II) oxygenase family protein" 0.461862971 0.244221914 -1.159143864 2.531845245 0.363761815 -2.953297305 1.004176496 -0.451191779

At2g30970 ASP1 (ASPARTATE AMINOTRANSFERASE 1); L-aspartate:2-oxoglutarate aminotransferase -0.891542222 0.556061891 0.568707748 0.547698206 -0.317607209 0.848331201 0.78613326 0.7191554

At2g30860 ATGSTF7_GLUTTR__ATGSTF9 (GLUTATHIONE S-TRANSFERASE PHI 9); glutathione peroxidase/ glutathione transferase -0.060450278 0.380407176 -0.642118931 0.128537971 1.273567418 0.655941979 -1.615960724 2.036716111

At2g30870 ATGSTF10_ATGSTF4_ERD13__GSTF10 (HALIANA GLUTATHIONE S-TRANSFERASE PHI 10); copper ion binding / glutathione binding / glutathione transferase -1.13635487 0.899327122 -0.438982517 0.530675308 1.135685595 -0.49233432 -0.88469977 3.546987655

At2g37690 "phosphoribosylaminoimidazole carboxylase, putative / AIR carboxylase, putative" 0.989287462 0.891327399 0.693526854 1.027538941 -0.178875856 0.295980078 -1.43786693 0.486140987

At2g37700 catalytic/ iron ion binding / oxidoreductase -0.442251323 0.524284483 0.61481988 -0.092751699 0.071181335 -0.283297748 0.383429903 -0.43991641

At2g44160 MTHFR2 (METHYLENETETRAHYDROFOLATE REDUCTASE 2); methylenetetrahydrofolate reductase (NADPH) -0.213881326 0.094087065 1.406038028 -0.522850876 -0.71022279 0.182241662 -0.035012167 1.120563707

At2g44050 "COS1 (COI1 SUPPRESSOR1); 6,7-dimethyl-8-ribityllumazine synthase" 0.114947475 -0.096345479 1.223536002 0.130197158 -0.088678014 0.199628558 -0.872459964 0.037365431

At2g30920 COQ3_EMB3002__ATCOQ3 (ARABDIOPSIS THALIANA COENZYME Q 3); hexaprenyldihydroxybenzoate methyltransferase/ polyprenyldihydroxybenzoate methyltransferase 0.304336776 0.419087941 0.33942652 -0.073886729 0.226467291 0.714799346 -0.425168241 -0.510834886

At2g02500 ATMEPCT_MCT__ISPD; 2-C-methyl-D-erythritol 4-phosphate cytidylyltransferase -0.025783948 -0.829555452 1.733071496 0.415109684 0.553564073 0.125604723 -0.134044669 0.304955789

At2g43880 "polygalacturonase, putative / pectinase, putative" -0.44344719 1.298499517 0.355263175 -0.422767128 -0.097477604 0.003954057 -0.097009997 -0.469978504

At2g44040 dihydrodipicolinate reductase family protein 0.298605672 0.197367084 2.481298522 0.717941232 0.111142315 -0.062013682 -0.456542384 -0.385560443

At2g23000 scpl10 (serine carboxypeptidase-like 10); serine-type carboxypeptidase -0.302727779 -1.293221385 0.261848026 0.862136693 -2.501590106 0.875636061 0.226731146 0.355227953

At2g22990 SCPL8__SNG1 (SINAPOYLGLUCOSE 1); serine-type carboxypeptidase/ sinapoylglucose-malate O-sinapoyltransferase -2.001511376 -4.177559703 -0.785750716 -2.92501076 0.344461832 -2.289680106 -0.337195469 -1.690117784

At2g19450 ABX45_AS11_ATDGAT_DGAT1_RDS1__TAG1 (TRIACYLGLYCEROL BIOSYNTHESIS DEFECT 1); diacylglycerol O-acyltransferase -0.608250987 1.530953799 -0.558046387 1.53762506 4.271692161 0.512637357 0.831196249 -1.672515732

At2g23630 sks16 (SKU5 Similar 16); copper ion binding / pectinesterase -0.967893032 1.551218794 0.838511479 -0.635838387 0.145911612 0.224410212 0.285970445 0.140171993

At2g23800 GGPS5__GGPS2 (GERANYLGERANYL PYROPHOSPHATE SYNTHASE 2); farnesyltranstransferase 0.95236423 0.030931731 0.225037098 -0.08731669 -0.128847926 0.083556993 2.554366072 -0.688570669

At2g30150 UDP-glucoronosyl/UDP-glucosyl transferase family protein 0.305218664 -0.87840064 0.578542794 -0.036279762 -0.205898006 0.433754879 0.271474733 1.55400578

At2g30140 UDP-glucoronosyl/UDP-glucosyl transferase family protein -1.541614261 -0.146614341 -3.865886719 2.21156566 -0.657903739 -0.805909426 -0.799845785 0.100515443

At2g30200 binding / catalytic/ transferase 0.354968309 0.054085392 1.694049713 -0.266036556 -0.225603584 0.855904208 -0.49294287 -0.069671406

At2g34770 FAH1 (FATTY ACID HYDROXYLASE 1); catalytic/ fatty acid alpha-hydroxylase -0.702178559 0.033455843 1.632500512 0.548048353 0.594000564 -2.31025676 1.810868914 0.084128956

At2g44520 COX10 (cytochrome c oxidase 10); prenyltransferase/ protoheme IX farnesyltransferase -0.054528384 0.444804142 -0.44656798 0.609215444 -0.064626284 0.217977579 -0.229504705 0.161046331

At2g44530 "ribose-phosphate pyrophosphokinase, putative / phosphoribosyl diphosphate synthetase, putative" 0.53546507 -0.113887569 -0.353775212 0.137874899 0.553754358 0.060845145 -0.748782038 0.064559245

At2g44350 CSY4__ATCS; ATP binding / ATP citrate synthase/ citrate (SI)-synthase -0.351161079 0.898717269 -0.737833117 0.385713586 -0.038366987 0.588621472 0.215400629 0.069182058

At2g26230 "uricase / urate oxidase / nodulin 35, putative" -0.317816601 0.473110232 -1.594823292 -0.471056357 0.929347938 0.52554658 0.352390698 0.360554474

At2g44490 "BGLU26__PEN2 (PENETRATION 2); hydrolase, hydrolyzing O-glycosyl compounds / thioglucosidase" -2.316651802 -0.569905839 -0.851729662 -0.218491728 0.796138807 -1.978352444 -1.089848545 2.533911538

At2g44540 "AtGH9B9 (Arabidopsis thaliana glycosyl hydrolase 9B9); catalytic/ hydrolase, hydrolyzing O-glycosyl compounds" 0.052925645 0.302971557 0.47040277 0.27548352 0.180917021 -0.350689803 0.7252797 -0.508994164

At2g44560 "AtGH9B11 (Arabidopsis thaliana glycosyl hydrolase 9B11); catalytic/ hydrolase, hydrolyzing O-glycosyl compounds" -0.362069355 0.874025874 0.676286558 0.667167247 0.285834898 -0.471990683 3.43461192 -0.227913189

At2g34890 "CTP synthase, putative / UTP--ammonia ligase, putative" 0.052589278 0.147656758 0.472341289 0.00672086 0.221845294 -0.485046182 0.153989311 -0.643761402

At2g35040 AICARFT/IMPCHase bienzyme family protein 0.198070776 0.021680433 1.182694029 0.674949327 -0.316302048 0.377589644 -0.816301663 -0.10398697

At2g34850 MEE25 (maternal effect embryo arrest 25); UDP-glucose 4-epimerase/ binding / catalytic/ coenzyme binding 0.725677531 0.412437025 -0.056954574 1.410275871 0.054755513 -0.240924467 -0.552552019 -1.340607728

At2g35020 UTP--glucose-1-phosphate uridylyltransferase family protein 0.074059051 0.102488854 0.415207289 -0.155619491 0.30753485 0.672909562 -0.529894226 0.346325709

At2g19150 pectinesterase family protein -0.149988645 0.636358334 0.213392558 -0.052255135 0.12096895 -0.178086653 -0.040769023 -0.450765661

At2g30490 ATC4H_CYP73A5__C4H (CINNAMATE-4-HYDROXYLASE); trans-cinnamate 4-monooxygenase 1.331813156 0.411358243 -0.364673536 -0.583086207 -2.871397795 0.017250843 -1.896442092 0.744452617

At2g30390 FC2 (FERROCHELATASE 2); ferrochelatase -0.434111364 -1.38810261 0.281192613 0.634875903 0.375378403 0.998596836 0.035246557 0.232390614

At2g30550 lipase class 3 family protein -0.304651555 -0.345220076 -2.499061231 0.620641657 -0.346977548 -0.900006292 -0.336511719 0.244541761

At2g45560 CYP76C1; electron carrier/ heme binding / iron ion binding / monooxygenase -1.203077981 -0.629744479 -0.390162895 -0.189815334 3.787565995 0.410381696 1.867400026 -1.386852315

At2g30570 PSBW (PHOTOSYSTEM II REACTION CENTER W) 0.763479707 -3.348853371 -0.609364649 -0.535654773 -0.09278076 -0.053914891 -0.853936373 -0.011790861

At2g42010 PLDBETA1 (PHOSPHOLIPASE D BETA 1); phospholipase D -0.348032485 0.371292941 -0.498121796 0.267278783 -0.243378996 -0.786304362 1.063185187 -0.140353914

At2g41880 AGK1__GK-1 (GUANYLATE KINASE 1); guanylate kinase 0.06228342 0.265721603 0.244185652 1.6005229 -0.85479929 -1.827994245 0.215943181 -1.003954053

At2g32770 ATPAP13_PAP13__purple acid phosphatase (PAP13) 0.855074627 0.008437661 0.655442365 0.2913165 0.06543938 -0.144400088 0.034615455 -0.203390761

At2g30770 "CYP71A13 (cytochrome P450, family 71, subfamily A, polypeptide 13); indoleacetaldoxime dehydratase/ oxygen binding" 0.819055369 0.370775312 -2.714647956 3.263020117 0.196506004 -0.813455203 -1.403092707 0.824948455

At2g32990 "AtGH9B8 (Arabidopsis thaliana glycosyl hydrolase 9B8); catalytic/ hydrolase, hydrolyzing O-glycosyl compounds" -0.452051489 0.761423901 1.108802148 -0.946729765 -2.385216197 1.858232084 -0.216737007 0.116096765

At2g26640 "KCS11 (3-KETOACYL-COA SYNTHASE 11); acyltransferase/ catalytic/ transferase, transferring acyl groups other than amino-acyl groups " -0.472045201 0.211723897 1.449202696 -1.42838853 0.679580687 0.232314845 -0.100462026 -0.373894378
