# Supplementary material for: Two glycosyltransferases involved in anthocyanin modification delineated by transcriptome independent component analysis in Arabidopsis thaliana
Source: Plant J. 2011 Oct 14;69(1):154–67. doi: 10.1111/j.1365-313X.2011.04779.x (PMC3507004; doi:10.1111/j.1365-313X.2011.04779.x)
Supplement: Supplementary file 8 [file tpj0069-0154-SD5.doc]

**Supplemental Data Set 2.** The Alignment Used for Construction of the Phylogenetic Tree Shown in Figure 3.

#PhA3G6RhaT ----NEMKHS ----NDALHV VMFP-FFAFG HISPFVQLAN KLSSYGVKVS FFTASGNASR

#BpA3G2GlcAT ------.DSK ID--SKTFR. ..L.-WL.YS ...R.LVF.K R.TNHNFHIY ICSSQT.MQY

#IpA3G2GT ------.GSQ ----ATTY.M A.Y.-W.GV. .LTG.FR... ..AGK.HRI. .LIPKNTQ.K

#CmF7G2RhaT ------.DTK HQ--DKPS-I L.L.-WL.H. ..A.HLE..K ...QKNFHIY .CSTPN.LQS

#AcA3Ga2XylT ------.G-- ----APTF.I A.Y.-W..L. .LT..LH.S. ..ARK.H.I. .LIPTKTQQK

#UGT79B1 ------.GVF GSNESSSMSI ..Y.-WL... .MT..LH.S. ..AEK.H.IV .LLPKKALNQ

#UGT78D1 -------MTK FSEPIRDS.. AVLAF.PVGA .AG.LLAVTR R.A-AASP-. TIFSFF.TA.

#UGT78D2 -------MTK PSDPTRDS.. AVLA-.PFGT .AA.LLTVTR R.A-SASP-. TVFSFF.TAQ

#UGT78D3 -------MAK PSQPTRDS.. AVLV-.PFGT .AA.LLAVTC R.A-TAAP-. TVFSFFSTA.

#Vv3GlcT ---------- ----TTNP.. AVLA-.PFST .AA.LLAVVR R.A-AAAP-H AVFSFFST.Q

#Ph3GalT ---------- ----MSNY.. AVLA-.PFAT .AGLLLG.VQ R.A-NALP-N VTFTFF.T.K

#Ph3GlcT ---------- --MTTSQ..I ALLA-.PFGS .AA.LLT.VQ ...-PFLPSD TIFSFF.T.Q

#Pf3GlcT ---------- ---MGFE..I GVLA-.PFGT .AP.LLA.VR R.A-ASSP-G TLFSFL.SAE

#Hv3GlcT ------.AP- -----PPP.I AVVA-.PFSS .AAVLFSF.R A.AAAAP-AG TSLSFLTTAD

#Zm3GlcT ------.APA DGESSPPP.. AVVA-.PFSS .AAVLLSI.R A.AAAAAPSG ATLSFLSTAS

#At5GlcT ------.AT. VNGSHRRP.Y LLVT-.P.Q. ..N.AL.... R.IHH.AT.T YS..VSAHR.

#Pf5GlcT ---------- ----MVRRR. LLAT-.P.Q. ..N.AL.F.K R.LKA.TD.T ...SVYAWR.

#Vh5GlcT ---------- ----MSRA.. LLAT-.P.Q. ..N.AL.F.K R.ANADIQ.T ...SVYAWR.

#Ph5GlcT ---------- ----MVQP.. ILTT-.P.Q. ..N.AL.F.K N.VKM.IE.T .S.SIYAQ..

#At7GlcT ------.AFE KNNEPFP..F .L..-.M.Q. .MI.M.DI.R L.AQR..LIT IV.TPH..A.

#At7RhaT ---------M TTTTTKKP.. LVI.-.PQS. .MV.HLD.TH QILLR.AT.T VLVTPK.S.Y

#DbB5GlcT --------MG THSTAPD... .F..-.L.H. .MI.SLDI.K LFAAR...TT II.TPL...M

#NtIS5a ---------- ----MGQ..F FF..-VM.H. .MI.TLDM.K LFA.R...AT II.TPL.EFV

#Gt3GlcT ---------- ----M.Q... FF..-.L.N. ..L.TIDM.K LF..R...AT LI.THN.SAI

#UGT84A2 ------.ELE SSP-PLPP.. MLVS-.PGQ. .VN.LLR.GK L.A.K.LLIT .V.TESWGKK

#UGT84A1 MGSIS..VFE TCPSPNPI.. MLVS-.QGQ. .VN.LLR.GK LIA.K.LL.T .V.TELWGKK

#PhA3G6RhaT VKS--MLNSA --------PT THIVPLTLPH VE-GLPPGAE STAELTPASA ELLKVALDLM

#BpA3G2GlcAT L.NNLTSQYS --------KS IQLIE.N..S SS-E..LQYH T.HG.P.HLT KT.SDDYQKS

#IpA3G2GT LE.--FNLHP --------HL ISF..IVV.S IP-....... T.SDVPFP.T H..ME.M.KT

#CmF7G2RhaT FGRNVEK.FS --------SS IQLIE.Q..N TFPE..SQNQ T.KN.P.HLI YT.VG.FEDA

#AcA3Ga2XylT LEP--FNLHP --------DL ITFI.V.V.. .D-...L... T.SDVPYPLQ T..MT.M.RT

#UGT79B1 LEP--LNLYP --------NL ITFHTISI.Q .K-....... TNSDVPFFLT H..A..M.QT

#UGT78D1 SNASLFSS-- -----DHPEN IKVHDVSDGV P.G-TML.NP LEM-VELFLE AAPRI-FRSE

#UGT78D2 SN.SLFSSGD EA---DRPAN IRVYDIADGV P.GYVFS.RP QE.-IELFLQ AAPEN-FRRE

#UGT78D3 SN.SLLSS-- -----DIPTN IRVHNVDDGV P.GFVLT.NP QH.-VELFLE AAPEI-FRRE

#Vv3GlcT SNASIFHD.- MH---TMQCN IKSYDISDGV P.GYVFA.RP QED-IELFTR AAPES-FRQG

#Ph3GalT SN.SLFTTP- ------HDNN IKPFNISDGV P.GYVVGKGG IE.LIGLFFK SAKEN-IQNA

#Ph3GlcT SNTSIFSEGS K------PDN IKVYNVWDGV T.TNGNKPVG LE.-IKLFIQ ATPTN-FEKV

#Pf3GlcT SNAALFNERT -------YDN IRAFDVWDGT P.GRIFT.TH FE.-VGLFLK ASPGN-F.KV

#Hv3GlcT NAAQLRKAG- -----ALPGN LRF.EVPDGV PPGETSCLSP PRR-MDLFM. AAEAGGVRVG

#Zm3GlcT SLAQLRKA.S ASAGHGLPGN LRF.EVPDGA PAAEETVPVP -RQ-MQLFME AAEAGGVKAW

#At5GlcT MGEPPS-TKG ---------- LSFAWF.DGF DDGLKS-FED QKIYMSELKR CGSNALR.II

#Pf5GlcT MANTASAAAG NPP------G LDF.AFSDGY DDGLK.-CGD GKRYMSEMK. RGSEALRN.L

#Vh5GlcT MSRTAAGSNG ---------L INF.SFSDGY DDGLQ.-.DD GKNYMSEMKS RGI.ALS.TL

#Ph5GlcT MDEKSI..AP --K------G LNFI.FSDGF D.GFDH-SKD PVFYMSQLRK CGSETVKKII

#At7GlcT F.NVLNRA-I ESGLPINLVQ VKFPYQEAGL Q.GQENMDLL T.M.QITSFF KAVNLLKEPV

#At7RhaT LDALRS.H-- -SPEHFKTLI LPFPS-HPCI PSGVESLQQL P-L.AIVHMF DA.SRLH.PL

#DbB5GlcT FTKAIEKTRK NTETQMEIEV FSFPSEEAGL PLGCENLEQA MAIGANNEFF NAANLLKEQL

#NtIS5a FSKAIQR.-K HLGIEIEIRL IKFPAVENGL P.ECERLDQI PSD.KL.NFF KAVAMMQEPL

#Gt3GlcT FLKAINRS-K ILGFDISVL. IKFPSAEFGL P.GYETADQA RSIDMMDEFF RACILLQEPL

#UGT84A2 MRISNKIQDR VLKP-VGKGY LRYDFFDDGL P.DDEASRTN L.ILRPHLEL VGKREIKN.V

#UGT84A1 MRQANKIVDG ELKP-VGSGS IRFEFFDEEW A.DDDR-R.D FSLYIAHLES VGIREVSK.V

#PhA3G6RhaT QPQIKTLLSH LKPHFVLFDF AQEWLPK-MA NGLGIKTVYY SVVVALSTAF LTCP--ARVL

#BpA3G2GlcAT G.DFE.I.IK .N..L.IY.. N.L.A.E-V. ST.H.PSIQL LSGCVALY.L DAHL------

#IpA3G2GT .ND.EII.KD ..VDV.FY.. T-H...S-L. RKI...S.F. .TISP.MHGY ALS.--E.RV

#CmF7G2RhaT K.AFCNI.ET ...TL.MY.L F.PMAAE-A. YQYD.AAILF LPLS.VACS. .LHN------

#AcA3Ga2XylT EKYVEDV.LG ..VDV.F... T-H.M.S-V. KR....S.N. CIISPATIGY TMS.--..Q.

#UGT79B1 R.EVE.IFRT I..DL.FY.S .-H.I.E-I. KPI.A...CF NI.S.A.I.L SLV.SAE.EV

#UGT78D1 IAAAEIEVG- K.VTCM.T.A FFWFAAD-I. AE.NATW.AF WAGG.N.LCA HLYTDLI.ET

#UGT78D2 IAKAE.EVG- TEVKCLMT.A FFWFAAD-.. TEINASWIAF WTAG.N.LSA HLYTDLI.ET

#UGT78D3 IKAAE.EVG- R.FKCI.T.A FLWLAAETA. AEMKASW.A. YGGG.T.LTA HLYTDAI.EN

#Vv3GlcT MVMAVAETG- RPVSCLVA.A FIWFAAD-.. AEM.VAWLPF WTAGPN.LST HVYIDEI.EK

#Ph3GalT MAAAVEESG- K.ITC.MA.A FMWFSGE-I. EE.SVGWIPL WTSA.G.LSV HVYTDLI.EN

#Ph3GlcT MKEAEEETG- V.FSCIFS.A FLWFSY.-L. EKINVPWIAF WTAASG.LSV HLYTDFI.--

#Pf3GlcT IEEAEPKTG- ..ICCLIT.A FLWFACD-.. QKR.LPW.PF WTAASC.LSS HLYTDQIVKA

#Hv3GlcT LEAACASAGG ARVSC.VG.A FVW-TAD-A. SAA.APW.AV WTAASCALLA HLRTDAL.RD

#Zm3GlcT LEAARAAAGG ARVTC.VG.A FVWPAAD-A. ASA.APW.PV WTAASCALLA HIRTDSL.ED

#At5GlcT KANLDATTET EPITG.IYSV LVP.VST-V. REFHLP.TLL WIEP.TVLDI YYYYFNTSYK

#Pf5GlcT LNN------- HDVT..VYSH LFA.AAE-V. RESQVPSALL W.EP.TVLCI YYFYFNG-YA

#Vh5GlcT AANNVD-QKS S.IT..VYSH LFA.AA.-V. REFHLRSALL WIEP.TVLDI FYFYFNG-YS

#Ph5GlcT LTCS---ENG QPITCL.YSI FLP.AAE-V. REVH.PSALL WSQP.TILDI YYFNFHG-YE

#At7GlcT .NL.EEMSP- -R.SCLIS.M CLS-YTSEI. KKFK.PKILF HGMGCFCLLC VNVLRKN.EI

#At7RhaT VDFLSRQPPS DL.DAI.GSS FLSPWINKV. DAFS..SISF LPIN.H.ISV MWAQEDRS--

#DbB5GlcT ENFLVKT--- -R.NCLVA.M FFT-WAADST AKFN.P.LVF HGFSFFAQCA KEVMWRYK-P

#NtIS5a EQL.EEC--- -R.DCLIS.M FLP-WTTDT. AKFN.PRIVF HGTSFFALCV ENSVRLNK-P

#Gt3GlcT EELL.EH--- -R.QALVA.L FFY-WANDA. AKF..PRLLF HGSSSFAMIA AESVRRNK-P

#UGT84A2 KRYK--EVTK QPVTCLINNP FVS.VCD-V. ED.Q.PCAVL W.QSCACL.A YYYYHHNL.G

#UGT84A1 RRYE--EAN- EPVSCLINNP FIP.VCH-V. EEFN.PCAVL W.QSCACFSA YYHYQDGS.S

#PhA3G6RhaT EPKKYPSLED MKKPPLGFPQ TSVTSVRTFE ARDFLYVFKS FHNGP---TL YDRIQSGLR-

#BpA3G2GlcAT ---------- YT..-.DENL AKFP-FPEIY PKNR----DI PKG.S---KY IE.FVDCM.R

#IpA3G2GT V-G.QLTEA. .M.A.AS..D P.IK-LHAH. ..--GFTART VMKFGGDI.F F...FTAVS-

#CmF7G2RhaT ---------- IVN.S.KY.F FESD-YQDR. SKNIN.FLHL TA..T---LN K..FLKAFEL

#AcA3Ga2XylT Q-GRELTEA. LMV..I.Y.D FLIR-L..H. ..--AFAARR VMKFGGDTRF C..NFISFS-

#UGT79B1 IDG.EM.G.E LA.T...Y.S SK.V-L.PH. .KSLSF.WRK HEAIG---SF F.GKVTAM.-

#UGT78D1 IGL.DV--SM EETLGFIPGM ENYR-.KDIP EEVVFEDLD. VFPKALYQ-- ---MSLA.PR

#UGT78D2 IGV.EVGERM EETIGVISGM EKIR-.KDTP EGVVFGNLD. VFSKMLHQ-- ---MGLA.PR

#UGT78D3 VGV.EVGERM EETIGFISGM EKIR-.KDTQ EGVVFGNLD. VFSKTLHQ-- ---MGLA.PR

#Vv3GlcT IGVSGIQGRE DELLNFIPGM SK.R-F.DLQ EGIVFGNLN. LFSRMLHR-- ---MGQV.PK

#Ph3GalT VEAQGIAGRE DEILTFIPGF AELR-LGSLP SGVVSGDLE. PFSVMLHK-- ---MGKTIGK

#Ph3GlcT -------SN. ETSLN-IPGF S.TLKISDMP PEVMAENLDL PMPSMLYN-- ---MALN.HK

#Pf3GlcT G-----TANQ EQNLSFIPGL EMA.-LTDLP PEV..DNSP. PLAITINK-- ---MVEK.PK

#Hv3GlcT VG-DQAASRA DELLVAHAGL GGYR-..DLP DGVVSGD.NY VISLLVHR-- ---QAQR.PK

#Zm3GlcT VG-DQAANRV DEPLISHPGL A.YR-..DLP DGVVSGD.NY VISLLVHR-- ---MGQC.PR

#At5GlcT HLFDVEP--- ---IK.PK-L PLI.-TGDLP SFLQPSKALP SALVTL---R EH-.EALETE

#Pf5GlcT DEIDAG.D.- ---IQ.PR-L PPLE-Q.SLP TFLLPETPER -FRLMM---K EK-LETLDGE

#Vh5GlcT DEIDAG.DA- ---IH.PGGL PVLA-Q.DLP SFLLPSTHER -FRSLM---K EK-LETLEGE

#Ph5GlcT KAMANE.NDP NWSIQ.PG-L PLLE-T.DLP SFLLP.GA.G SLRVALPPFK EL-.DTLDAE

#At7GlcT LDNLKSDK.Y FIV.YFPDRV EFTR--PQVP VETYVPAG-- -WKEILEDMV ------EADK

#At7RhaT ---------- ---------- ---------- ---------- ----FFNDLE ------TATT

#DbB5GlcT YKAVSSDT.V FSL.F.PHEV KMTR--LQVP ESMRKGEE-T HFTKRTERIR ------E.ER

#NtIS5a FKNVSSDS.T FVV.D.PHEI KLTR--TQVS PFERSGEE-T AMTRMIKTVR ------ESDS

#Gt3GlcT YKNLSSDSDP FVV.DIPDKI ILTK--SQVP TP.ETEENNT HITEMWKNIS ------ESEN

#UGT84A2 F.T.TEPEI. ---VQISG-M PLLK-HDEIP SFIHPSSPH. ALREVIID-- ----.IKRLH

#UGT84A1 F.TETEPEL. ---VK.PC-V PVLK-NDEIP SFLHPSSRFT GFRQAILG-- ----.FKNLS

#PhA3G6RhaT GCSAILAKTC SQMEGPYIKY VEAQFNK--P VFLIGPVVP- -DPPSGKLE- ----------

#BpA3G2GlcAT S.EI..VRST MEL..K..D. LSKTLG.--K .LPV..L.Q- EASLLQDDH- ----------

#IpA3G2GT ESDGLAYS.. REI..QFCD. I.T..Q.--. .L.A..AL.- -V.SKSTM.- ----------

#CmF7G2RhaT S.KFVFI..S REI.SK.LD. FPSLMGN--E IIPV..LIQ- EPTFKVDD-- ----------

#AcA3Ga2XylT E.D.MGF... REI....CD. L.S..G.--. .L.S...I.- -E..TSP..- ----------

#UGT79B1 N.D..AIR.. RET..KFCD. ISR.YS.--. .Y.T...L.G SQ.NQPS.D- ----------

#UGT78D1 -A..VFISSF EEL.PT-LN. NLRSKL.--R FLN.A.LTLL SSTSEK--.- -------MRD

#UGT78D2 -AT.VFINSF EDLDPT-LTN NLRSRF.--R YLN...LGLL SSTLQQ--L- -------VQD

#UGT78D3 -AT.VFINSF EELDPT-FTN DFRSEF.--R YLN...LALL SS.SQTSTL- -------VHD

#Vv3GlcT -AT.VFINSF EELDDS-LTN DLKSKL.--T YLN...FNLI TP..VVP--- ---------N

#Ph3GalT -AT.LPVNSF EELDP.-.VE DLKSKFN--N FLNV..FNLT TP...AN--- -------ITD

#Ph3GlcT -AA.VVLNSF EELDPT-.NK DLKVKLQ--K .LN...L.LQ PTS.KKV.D- -------ACD

#Pf3GlcT -ST.VVLNSF EEIDPI-.TD DLKTKF.--N FLNV..SILA SP-.QATPD- ---------D

#Hv3GlcT AAT.VALN.F PGLDP.DLIA AL.AELP--N CLPL..YHLL PGAEPTADTN -----EAPAD

#Zm3GlcT SAA.VALN.F PGLDP.DVTA AL.EILP--N CVPF..YHLL L-AEDDADT- -----AAPAD

#At5GlcT SNPK..VN.F .AL.HDALTS ..KLKMI--. IGPLVSSS-- EGK------- -TDLFKS--S

#Pf5GlcT EKAKV.VN.F DAL.PDALTA IDRYELI--G IGPLI.SAFL DGGDPSETSY GGDLFEK-SE

#Vh5GlcT EKPKV.VNSF DAL.PDAL.A IDKYEMI--A IGPLI.SAFL DGKDPSDRSF GGDLFEKGSN

#Ph5GlcT TTPK..VN.F DEL.PEALNA I.GYKFY--G IGPLI.SAFL GGNDPLDASF GGDLFQN---

#At7GlcT TSYGVIVNSF QEL.PA.A.D FKEARS--GK AWT....SLC NKVGVD.A.R GN---KSDID

#At7RhaT ESYGLVINSF YDL.PEFVET .KTR.LNHHR IWTV..LL.F KAG----VDR GG---QSSIP

#DbB5GlcT KSYGVIVNSF YEL.PD.ADF LRKELG--RR AWH....SLC NRSIED.AQR GR---QTSID

#NtIS5a KSYGVVFNSF YEL.TD.VEH YTKVLG--RR AWA...LSMC NRDIED.A.R GK---KSSID

#Gt3GlcT D.YGVIVNSF YEL.PD.VD. CKNVLG--RR AWH...LSLC NNEGEDVA.R GK---KSDID

#UGT84A2 KTFS.FID.F NSL.KDI.DH MSTLSLP--G .IRPLGPLYK MAKTVAYDVV KVNISEP---

#UGT84A1 KSFCV.IDSF DSL.REV.D. MSSLCP---- -VKTVGPLFK VARTVTSDVS G-DICKS---

#PhA3G6RhaT -EKWATWLNK FEG-GTVIYC SFGSETFL-T DDQVKELALG LEQTGLPFFL VLNFPANVDV

#BpA3G2GlcAT -IWIMK..D. K.E-SS.VFV C....YI.-S .NEIEDI.Y. ..LSQVS.VW AIRAKT----

#IpA3G2GT -Q..SD..G. .KE-.S.... A....CT.-R K.KFQ..LW. ..L..M...A A.KP.FETES

#CmF7G2RhaT -T.IMD..SQ K.P-RS.V.A .....Y.P-S T.EIHDI.I. .LL.EVN.IW AFRLHPDEKM

#AcA3Ga2XylT -.I..K..GG .RA-.S.... A....CT.-K MN.FQ..L.. .VL..M..LA ..KP.IGAKS

#UGT79B1 -PQ..E..A. .NH-.S.VF. A...QPVVNK I..FQ..C.. ..S..F..LV AIKP.SG.ST

#UGT78D1 PHGCFA.MG. RSA-AS.A.I ...TVME-PP PEELVAI.Q. ..SSKV..VW S.KEKNM.H-

#UGT78D2 PHGCLA.ME. RSS-.S.A.I ...TVMT-PP PGELAAI.E. ..SSKV..VW S.KEKSL.Q-

#UGT78D3 PHGCLA.IE. RST-AS.A.I A..RVAT-PP PVELVAI.Q. ..SSKV..VW S.QEMKMTH-

#Vv3GlcT TTGCLQ..KE RKP-TS.V.I ...TV.T-PP PAE.VA.SEA ..ASRV..IW S.RDK.R.H-

#Ph3GalT EYGCIA..D. Q.P-.S.A.I G..TAAT-PP PNEP.AM.EA ..ESKT..LW S.KDLFKSF-

#Ph3GlcT ERGCII..E. QKE-ES.V.L ...TV.T-LP PNEIVAV.EA ..AKKF..IW S.KDNGIKN-

#Pf3GlcT ETGCLS..AD QTSPKS.V.I ...TVIT-PP ENELAA..DA ..ICRF..LW S.KDY.VKS-

#Hv3GlcT PHGCLA..DR RPA-RS.A.V ...TNAT-AR P.ELQ...A. ..AS.A..LW S.R-GVVAA-

#Zm3GlcT PHGCLA..GR QPA-RG.A.V ...TVAC-PR P.ELR...A. ..ASAA..LW S.REDSWTL-

#At5GlcT D.DYTK..DS KLE-RS...I .L.THADDLP EKHMEA.TH. VLA.NR..LW IVREK-----

#Pf5GlcT ENNCVE..DT KPK-SS.V.V ....VLR-FP KA.ME.IGK. .LAC.R..LW MIREQK.D.G

#Vh5GlcT DDDCLE..ST NPR-SS.V.V ....FVN-T. KS.ME.I.R. .LDC.R..LW .VRVN-----

#Ph5GlcT SNDYME...S KPN-SS.V.I ....LMN-PS IS.ME.ISK. .IDI.R..LW .IKENEKGK-

#At7GlcT QDECLE..DS K-EP.S.L.V CL..ICN-LP LS.LL..G.. ..ESQR..IW .IRGWEKYKE

#At7RhaT PA.VSA..DS CPEDNS.V.V G...QIR-L. AE.TAA..AA ..KSSVR.IW AVRDA.KKVN

#DbB5GlcT EDECLK...S K-KPDS...I C...TGH-LI AP.LH.I.TA ..AS.QD.IW AVRGDHGQGN

#NtIS5a KHECLK..DS K-KPSS.V.V C...VAN-F. AS.LH...M. I.AS.QE.IW .VRTELDNED

#Gt3GlcT AHECLN..DS K-NPDS.V.V C...MAN-FN AA.LH...M. ..ES.QE.IW .VRTCVDEED

#UGT84A2 TDPCME..DS QPV-SS.V.I ...TVAY-LK QE.ID.I.Y. VLNADVT.LW .IRQQELGFN

#UGT84A1 TD.CLE..DS RPK-SS.V.I ...TVAY-LK QE.IE.I.H. VLKS..S.LW .IRP.PHDLK

#PhA3G6RhaT SAE----LNR ALPEGFLERV K--DKGIIHS GWVQQQHILA HSSVGCYVCH AGFSSVIEAL

#BpA3G2GlcAT ---------- SALN..ID.. G--...LVID K..P.AN..S ...T.GFIS. C.W..TM.SI

#IpA3G2GT -------VEA .I..ELK.KI Q--GR..V.G E.....LF.Q .P....F.S. C.WA.LS...

#CmF7G2RhaT T------IEE ...Q..A.EI ERNN..M.VQ ...P.AK..R .G.I.GFLS. C.WG..V.GM

#AcA3Ga2XylT -------VEE ....K.ETG. E--GR.VV.E ......L..E .P....FIT. C.SG.LS...

#UGT79B1 -------VEE ......K... Q--GR.VVFG ..I..PLV.N .P....F.S. C..G.MW.S.

#UGT78D1 ---------- -..K...D.T REQ--..VVP -.AP.VEL.K .EAM.VN.T. C.WN..L.SV

#UGT78D2 ---------- -..K...D.T REQ--..VVP -.AP.VEL.K .EAT.VF.T. C.WN..L.SV

#UGT78D3 ---------- -......D.T REQ--.MVVP -.AP.VEL.N .EAM.VF.S. G.WN..L.SV

#Vv3GlcT ---------- -.......KT RGY--.MVVP -.AP.AEV.. .EA..AF.T. C.WN.LW.SV

#Ph3GalT ---------- -F.......T SEY--.K.V. -.AP.VQV.S .G...VFIN. C.WN..L.SI

#Ph3GlcT ---------- -..T.....T GQF--.K.V. -.AP.LE..N ..A..VF.T. C.WN.IL.GI

#Pf3GlcT ---------- -..D...D.T .GF--.K.VA -.AP..QV.. .RN..VF.T. C.WN.IL.SI

#Hv3GlcT ---------- -A.R.....A P----.LVVP -.AP.VGV.R .AA..AF.T. ..WA..M.GV

#Zm3GlcT ---------- -..P...D.A AGTGS.LVVP -.AP.VAV.R .P...AF.T. ..WA..L.GV

#At5GlcT NP.------E KKKNR...LI RGS.R.LVVG -.CS.TAV.. .CA...F.T. C.WN.TL.S.

#Pf5GlcT EE.------E EELSCIG.LK .---M.K.V. -.CS.LEV.. .PAL..F.T. C.WN.AV.S.

#Vh5GlcT EG.------E V.ISCME.LK R---V.K.V. -.CS.LEV.T .P.L..F.T. C.WN.TL.SI

#Ph5GlcT EE.------N KKLGCIE.LE .---I.K.VP -.CS.LEV.K .P.L..F.S. C.WN.AL.S.

#At7GlcT LV.------W FSES..ED.I QD--R.LLIK ..SP.ML..S .P...GFLT. C.WN.TL.GI

#At7RhaT .SDNSVEEDV IPAG-.E... .E--..LVIR ..AP.TM..E .RA..S.LT. L.WG..L.GM

#DbB5GlcT .E.------W LP.G-YEH.L QG--..L.IR ..AP.VL..E .EAT.GFLT. C.WN.AL.GI

#NtIS5a ---------W LPEG-.E..T .E--..L.IR ..AP.VL..D .E...AF.T. C.WN.TL.GV

#Gt3GlcT ESK------W FPDG-.EK.. QENN..L.IK ..AP.VL..E .EA..AF.S. C.WN.TL.GI

#UGT84A2 KEK------H V...EVK--- ---G..K.VE -.CS.EKV.S .P..A.F.T. C.WN.TM..V

#UGT84A1 VET------H V..QELK.SS A-KG..M.VD -.CP.EQV.S .P..A.F.T. C.WN.TM.S.

#PhA3G6RhaT VNDCQVVMLP QKGDQILNAK LVSGDMEAGV EINRR----- ----DEDGYF GKEDIKEAVE

#BpA3G2GlcAT RYGVPIIAM. MQF..PY..R .MET-VG..I .VG.D----- ----G.-.RL KR.E.AAV.R

#IpA3G2GT .....I.L.. .V....I..R IM.VSLKV.. .VEKG----- ----E...V. SR.SVCK..K

#CmF7G2RhaT .FGVPIIGV. MAYE.PS... V.VD-NGM.M VVP.D----- ----KINQRL .G.EVARVIK

#AcA3Ga2XylT F.K..L.L.. NV....I..R MM.QNLKV.. .VEKG----- ----E...L. TG.SVCR..R

#UGT79B1 MS...I.LV. .H.E.....R .MTEE..VA. .VE.------ ----EKK.W. SRQSLEN..K

#UGT78D1 SAGVPMIGR. ILA.NR..GR A.EVVWKV.. MMDNG----- ----VFTKEG ----FEKCLN

#UGT78D2 SGGVPMICR. FF...R..GR A.EVVW.I.M T.ING----- ----VFTKDG ----FEKCLD

#UGT78D3 SAGVPMICR. IF..HAI..R S.EAVW.I.. T.SSG----- ----VFTKDG ----FE.SLD

#Vv3GlcT AGGVPLICR. FF...R..GR M.EDVL.I.. R.EGG----- ----VFTKSG ----LMSCFD

#Ph3GalT AAGVP.ICR. FF..HQ...W M.EKVWKI.. K.EGG----- ----VFTKDG ----TML.LD

#Ph3GlcT SCGVPMICR. FF...K..SR M.ESVWQI.L Q.EGG----- ----SFTKIG ----TIS.LD

#Pf3GlcT SSCVPLICR. FF...K..SR M.QDSWKI.. RVEGG----- ----VFTKNE ----AV.SLK

#Hv3GlcT SSGVPMACR. FF...TM..R S.ASVWGF.T AFDG------ ----PMTRGA ----VAN..A

#Zm3GlcT SSGVPMACR. FF...RM..R S.AHVWGF.A AFEG------ ----AMTSAG ----VAA...

#At5GlcT ESGVP..AF. .FA..CTT.. ..EDTWRI.. KVKVG----- ----E-E.DV DG.E.RRCL.

#Pf5GlcT SCGVP..AV. .WF..TT... .IEDAWGT.. RVRMN----- ----E-G.GV DGSE.ERC..

#Vh5GlcT SFGVPM.AF. .WF..GT... .MEDVWRT.. RVRAN----- ----E.GSVV DGDE.RRCI.

#Ph5GlcT ACGVP..AF. .WT..MT... Q.EDVWKS.. RVRIN----- ----E-..VV ES.E..RCI.

#At7GlcT TAGLPMLTW. LFA..FC.E. ..VQILKV.. SAEVKEVMKW GEEEKIGVLV D..GV.K...

#At7RhaT .GGVMLLAW. MQA.HFF.TT .IVDKLR.A. RVGE------ -------NRD SVP.SDKLAR

#DbB5GlcT SAGVPM.TW. TFAE.FH.EQ .LTQILKV.. AVGS---KKW TLKPSIEDVI KA...EK..R

#NtIS5a SGGVPM.TW. VFAE.FF.E. ..TEVLKT.A GVGS---IQW KRSAS.G--V KR.A.AK.IK

#Gt3GlcT CGGVAM.TW. LFAE.FY.E. .MTDILRT.. SVGS---LQW SRVTTSAVVV KR.S.SK..R

#UGT84A2 SSGVPT.CF. .W...VTD.V YMIDVWKT.. RLS.G----- ----EAEERL VPREEVAERL

#UGT84A1 SSGVP..CC. .W...VTD.V YLIDVFKT.. RLG.G----- ----ATEERV VPREEVAEKL

#PhA3G6RhaT KVMVDVEKEP GKLIRENQKK WKEFLLNKDI QSKYIGNLVN EMTAMAKVST T*--------

#BpA3G2GlcAT ..V.EDS--- .ES...KA.E LG.IMK-.NM EAEVD.IVIE NLVKLCEMNN *---------

#IpA3G2GT A..DEKS-.I .REV.G.HD. LRG..M.A.L D...MDSFNQ KLQDLLG*-- ----------

#CmF7G2RhaT H.VLQE.--- A.Q..RKANE IS.SMK-.IG DAQMS-VV.E KLLQLV.K.E *---------

#AcA3Ga2XylT DA.EEGS-.V A.EV.D.HA. MR.......L E.S..D.FNK KLQDLLG*-- ----------

#UGT79B1 S..EEGS-.I .EKV.K.HD. .RCV.TDSGF SDG..DKFEQ NLIELV.S*- ----------

#UGT78D1 D.F.HDD--- ..TMKA.A.. L..K.QEDFS MKGSSLENFK ILLDEIVKV* ----------

#UGT78D2 ..L.QDD--- ..KMKC.A.. L..LAYEAVS SKGRSSENFR GLLDAVVNII *---------

#UGT78D3 R.L.QDD--- ..KMKV.A.. LE.LAQEAVS TKGSSFENFG GLLDEVVNFG *---------

#Vv3GlcT QILSQEK--- ..KL...LRA LR.TADRAVG PKGSSTENFI TLVDLVSKPK DV*-------

#Ph3GalT L.LSKDKR-- NTELKQQIGM Y..LA..AVG P.GSSAENFK KLVDIITSCN *---------

#Ph3GlcT TFFSEEK--- ..VL...V.G L..RA.EAVK PDGSSSKNFK DLVELV.CHK LT*-------

#Pf3GlcT .L.ATEA--- .MK....VSL LR.KATAAVK PEGSSSQNFK KLLEIIGAAE SS*-------

#Hv3GlcT TLLRGED--- .ERM.AKAQE LQAMVGKAFE PDGGCRKNFD .FVEIVCRV* ----------

#Zm3GlcT ELLRGE.--- .AGM.ARA.E LQALVAEAFG PGGECRKNFD RFVEIVCRA* ----------

#At5GlcT ...SGG.E-- AEEM...AE. ..AMAVDAAA EGGPSDLNLK GFVDEDE*-- ----------

#Pf5GlcT M..DGG..-- S..V...AI. ..TLAREAMG EDGSSLKNL. AFLHQVARA* ----------

#Vh5GlcT E..DGG..-- SRKL..SAG. ..DLARKAME EDGSSV.NLK VFLDEVVGI* ----------

#Ph5GlcT L..DGG..-- .EEL.K.A.. ...LAREAVK EGGSSHKNLK AFIDDVAKGF *---------

#At7GlcT EL.GESDD-- A.ER.RRA.E LG.SAHKAVE EGGSSHSNIT FLLQDIMQLA QSNN*-----

#At7RhaT ILAESARE-- DLPE.VTLM. LR.KAMEAIK EGGSSYKNLD .LV.EMCL*- ----------

#DbB5GlcT E...G-.E-- .EER.RRA.. L..MAWRAIE EGGSSYSDLS ALIEEL.GYH .SEKE*----

#NtIS5a R...S-.E-- ADGF.NRA.A Y..MARKAIE EGGSSYTGLT TLLEDISTYS STGH------

#Gt3GlcT RL.AE-.E-- .VD..NRA.A L..KAKKAVE GGGSSYSDLS ALLVELSSYP HN*-------

#UGT84A2 REVTKG..-- AIELKK.AL. ...EAEAAVA RGGSSDRNLE KFVEKLGAKP VGKVQNGSHN

#UGT84A1 LEATVG..-- AEEL.K.AL. ..AEAEAAVA PGGSSDKNFR .FVEKLGAG- VTKTKDNGY*

#PhA3G6RhaT ---------- -

#BpA3G2GlcAT ---------- -

#IpA3G2GT ---------- -

#CmF7G2RhaT ---------- -

#AcA3Ga2XylT ---------- -

#UGT79B1 ---------- -

#UGT78D1 ---------- -

#UGT78D2 ---------- -

#UGT78D3 ---------- -

#Vv3GlcT ---------- -

#Ph3GalT ---------- -

#Ph3GlcT ---------- -

#Pf3GlcT ---------- -

#Hv3GlcT ---------- -

#Zm3GlcT ---------- -

#At5GlcT ---------- -

#Pf5GlcT ---------- -

#Vh5GlcT ---------- -

#Ph5GlcT ---------- -

#At7GlcT ---------- -

#At7RhaT ---------- -

#DbB5GlcT ---------- -

#NtIS5a ---------- -

#Gt3GlcT ---------- -

#UGT84A2 HVLAGSIKSF *

#UGT84A1 ---------- -
