# Supplementary material for: Two glycosyltransferases involved in anthocyanin modification delineated by transcriptome independent component analysis in Arabidopsis thaliana
Source: Plant J. 2011 Oct 14;69(1):154–67. doi: 10.1111/j.1365-313X.2011.04779.x (PMC3507004; doi:10.1111/j.1365-313X.2011.04779.x)
Supplement: Supplementary file 9 [file tpj0069-0154-SD6.doc]

**SUPPORTING INFORMATION**

Additional Supporting Information may be found in the online version of this article;

**Figure S1.** A Schematic ICA Model of Gene Expression Data as a Strategy for Gene Discovery.

The ICA model was framed in black. An expression data matrix can be denoted as an *m* × *n* matrix *X* with rows and columns representing *m* genes and *n* samples, respectively, and may be considered to be a linear combination of independent components (*i.e.,* source signal *S,* *m* × *k* gene signature matrix). If *A* denotes latent mixing matrix (*k* × *n* latent vectors of the gene expression data), we can describe *X* = *SA* (black box). *k* represents the number of components to be extracted. Note that the rows of *S* (*i.e.,* ICs) are statistically independent from each other in ICA. The scheme of the strategy for gene discovery was shown in pink. HCA, hierarchical cluster analysis; IC, independent component.

**Figure S2.** Phylogenetic Tree and Multiple Alignment of Flavonoid UGTs Catalyzing Glycosyl Transfer to a Sugar Moiety of Flavonoid Glycosides.

(A) A phylogenetic tree of flavonoid UGTs catalyzing glycosyl transfer to a sugar moiety of flavonoid glycosides (GGTs). Prospective substrates of ancestral GGTs are shown in red. Bar = 0.2 amino acid substitutions per site. A3G2”, C-2” position of anthocyanin 3-*O*-glucoside; A3G6”, C-6” position of anthocyanin 3-*O*-glucoside; A3Ga2”, C-2” position of anthocyanin 3-*O*-galactoside; F7G2”, C-2” position of flavanone 7-*O*-glucoside; Glc, UDP-glucose; GlcA, UDP-glucuronic acid; Rha, UDP-rhamnose; Xyl, UDP-xylose; A3G2”GlcAT, anthocyanidin 3-*O*-glucoside 2”-*O*-glucuronosyltransferase; A3G2”GlcT, anthocyanidin 3-*O*-glucoside 2”-*O*-glucosyltransferase; A3G6”RhaT, anthocyanidin 3-*O*-glucoside 6”-*O*-rhamnosyltransferase; A3Ga2”XylT, anthocyanidin 3-*O*-galactoside 2”-*O*-xylosyltransferase; F7G2”RhaT, flavanone 7-*O*-glucoside 2”-*O*- rhamnosyltransferase; Abbreviations for species: Ac, *Actinidia chinensis*; At, *Arabidopsis thaliana*; Bp, *Bellis perennis*; Cm, *Citrus maxima*; Ip, *Ipomoea purpurea*; Ph, *Petunia hybrida.*

(B) Multiple alignment of deduced amino acid sequences of flavonoid GGTs was generated by a CLUSTALW program (http://www.clustal.org/). The PSPG box was surrounded by a box. Amino acid residues conserved in flavonoid GGTs are shown in white on a black background. An amino acid residue specifically conserved in flavonoid GGTs is indicated by a black triangle. Possible amino acid residues involved in the recognition of UDP-xylose and anthocyanin 3-*O*-glucoside are indicated with red and gray backgrounds, respectively, based on the assumption described in the text. Common ancestral sequences were calculated by Phylogenetic Analysis by Maximum Likelihood (PAML, ver.4.3).

**Table S1.** Primers used in this study

**Data S1.** Complete hierarchical clustering data of a gene signature matrix with 1877 metabolism-related genes based on 8 ICs. Java Treeview ([http://jtreeview.sourceforge.net](http://jtreeview.sourceforge.net/)) is required for visualization.

**Data S2.** The Alignment Used for Construction of the Phylogenetic Tree Shown in Figure 2.
